# Pre- and post-therapy functional MRI connectivity in severe acute brain injury with suppression of consciousness: a comparative analysis to epilepsy features

**DOI:** 10.3389/fnimg.2024.1445952

**Published:** 2024-10-01

**Authors:** Emilio G. Cediel, Erika A. Duran, Jeffrey Laux, William Reuther, Olivia Leggio, Belfin Robinson, Varina L. Boerwinkle

**Affiliations:** ^1^Clinical Resting State fMRI Service, Department of Neurology, University of North Carolina at Chapel Hill, Chapel Hill, NC, United States; ^2^North Carolina Translational and Clinical Sciences Institute, School of Medicine, University of North Carolina at Chapel Hill, Chapel Hill, NC, United States

**Keywords:** functional neuroimaging, connectome, seizures, brain injury, coma, consciousness disorders, epilepsy, antiseizure medication

## Abstract

Severe acute brain injury (SABI) with suppressed consciousness is a major societal burden, with early prognosis being crucial for life-and-death treatment decisions. Resting-state functional MRI (rs-fMRI) is promising for prognosis and identifying epileptogenic activity in SABI. While established for SABI prognosis and seizure networks (SzNET) identification in epilepsy, the rs-fMRI use for SzNET detection in SABI is limited. This study compared evolution of SzNET and resting-state networks (RSN) pre-to-post treatment in SABI and epilepsy, hypothesizing that changes would align with clinical evolution. Therapies included epilepsy surgery for the epilepsy group and antiseizure medication for the SABI group. Independent component analysis (ICA) was used to identify SzNET and RSNs in all rs-fMRI. High-frequency BOLD (HF-BOLD), an ICA power spectrum-based index, quantified RSN and SzNET changes by the patient. Confidence intervals measured HF-BOLD changes pre-to-post-therapy. Baseline HF-BOLD and HF-BOLD changes were compared using linear-mixed models and interaction tests. Five SABI and ten epilepsy patients were included. SzNET were identified in all SABI's pre-therapy rs-fMRI. The clinical changes in SABI and epilepsy were consistent with rs-fMRI findings across groups. HF-BOLD reduced in the epilepsy group RSN post-therapy (−0.78, 95% CI −3.42 to −0.33), but the evidence was insufficient to determine an HF-BOLD reduction in SABI patients or SzNET. The HF-BOLD change trend in pre-to-post epilepsy surgery scans paralleled the clinical improvement, suggesting that the power spectrum may quantify the degree of abnormality on ICA-derived networks. Despite limitations such as small sample sizes, this exploratory study provides valuable insights into network dysfunction in SABI and epilepsy.

## 1 Introduction

Severe acute brain injury (SABI) resulting in consciousness suppression poses a significant societal burden (Collaborators and Spinal Cord, 2019; Rivara et al., 2012; Rochmah et al., 2021; Schneier et al., 2006). Early prognosis in SABI is pivotal for treatment decisions, including the consideration of withdrawal of life-sustaining therapy (WLST), and relies on neurological reactivity assessments conducted through multiple modalities, in addition to clinical examination (Boerwinkle et al., 2023a; Edlow et al., 2017, 2021). However, the complexity of SABI involves various contributing factors to consciousness suppression, making it challenging to identify potentially reversible cases. Brain network dysfunction, such as epileptogenic activity, contributes to secondary brain injury and complicates prognosis assessment by masking neurological reactivity (Boerwinkle et al., 2022; La Rocca et al., 2023).

Resting-state functional MRI (rs-fMRI) is a valuable tool for both functional prognosis in SABI and detection of epileptogenic activity in epilepsy. Recent meta-analyses have highlighted the predictive value of rs-fMRI in SABI outcomes, as similarly observed across other neurological disorders (Hannawi et al., 2015; Lee et al., 2014; Sole-Padulles et al., 2017; Zhou et al., 2010; Zhou, 2021). Consciousness is fundamentally tied to the interconnection and integrated activity of multiple cortical and subcortical regions that can be understood as networks (Boly et al., 2008; Panda et al., 2023). Rs-fMRI can identify resting-state networks (RSNs) that play central integrative roles in supporting consciousness—including the default mode network (DMN), subcortical networks, and frontoparietal networks; differences in these networks clearly distinguish SABI patients from healthy controls (Edlow et al., 2021; O'Neill et al., 2017; Panda et al., 2022; Threlkeld et al., 2018). In particular, disruptions within these networks consistently correlate with the degree of consciousness suppression (Vanhaudenhuyse et al., 2010).

Similar to SABI, connectivity studies in epilepsy have shown relatively established impacts on RSN functioning, particularly the DMN (Luo et al., 2011; Zhang et al., 2010), which may have cross-informing attributes for SABI where seizures are not uncommon. In epilepsy, rs-fMRI detects networks with abnormal features associated with the seizure onset zones, termed seizure networks (SzNET) (Boerwinkle et al., 2017; Chakraborty et al., 2020). Intriguingly, rs-fMRI in SABI patients has detected abnormal brain networks resembling SzNET, raising the question of whether these networks could exert effects similar to those observed in epilepsy. The application of rs-fMRI in SABI for identifying SzNET during early injury stages is not well established beyond case reports (Boerwinkle et al., 2023b, 2019b), and there is limited understanding of significance of those networks in the context of SABI.

The altered cerebral physiology during the early phases of brain injury poses additional challenges to understanding the driving factors of SzNET in SABI. Epileptogenic activity disrupts normal brain connectivity, but other factors such as increased intracranial pressure, cerebral edema, hemorrhage, hydrocephalus, or cortical spreading depolarizations may also contribute (Andrew et al., 2022; Edlow et al., 2021). These factors lead to functional connectivity changes in traumatic brain injury (TBI) and ischemic injuries.

The application of rs-fMRI in SABI is filled with uncertainties. However, observing the alterations in connectivity features over time could offer preliminary insights into their importance in this field. In this paper, we provide a comparative study of the clinical and functional imaging progression in all the SABI cases where pre-and-post-therapy rs-fMRI scans were available. The objective was to evaluate the alignment between clinical and functional imaging progression. In addition, we incorporated high-frequency BOLD (HF-BOLD), which is an index derived from the power spectrum of the blood oxygen-dependent signal, for a quantitative analysis of progression of these networks. Considering the limited data on the evolution of RSN and SzNET during SABI, and to improve understanding, we juxtapose the HF-BOLD analysis with a consistent subgroup from an epilepsy surgery cohort, hypothesizing a decrease in HF-BOLD in both groups following their respective antiseizure treatments. By comparing SABI networks with established SzNET from epilepsy, we aimed to gain a deeper understanding of the features of SABI networks through their similarities and differences with the more extensively researched epilepsy network pathology. In summary, pathological network activity of SzNET is hypothesized to contribute to the suppression of consciousness observed in SABI, given the frequent observation of epileptic activity in these patients. This pathological activity could disrupt RSNs that support consciousness, impairing their normal function and potentially leading to consciousness impairment.

Observing the evolution of RSN and SzNET in SABI, along with their relationship to seizures and networks supporting consciousness, can significantly enhance the interpretability of early rs-fMRI findings. This enhanced understanding can, in turn, facilitate the classification of Disorders of Consciousness (DOC) within the endotypes approach advocated by the Curing Coma campaign (Boerwinkle et al., 2023a; Kondziella et al., 2021; Mainali et al., 2022; Provencio et al., 2020).

## 2 Methods and materials

### 2.1 Data collection and ethics

The local Institutional Review Boards approved the retrospective review of SABI cases with rs-fMRI results. This report includes data from two separate cohorts. The SABI group includes all the patients hospitalized in pediatric or neonatal intensive care units from May 2018 to June 2023 who received at least two rs-fMRI, determined to be clinically indicated by their care team and interpreted by the last author. At least one of the rs-fMRI must have been while in the ICU, and in every case, the first and the last rs-fMRI scans were used for the quantitative analysis. Of this group, patient number 3 and patient number 4 were included in a descriptive case series (Boerwinkle et al., 2023b). The scans were obtained when it was considered safe by the care team. Anatomical MRI sequences were acquired during the same scanning session.

The epilepsy cohort comprised all the patients with epilepsy attributed to malformations of cortical development, from a prior published article of rs-fMRI on epilepsy surgery (Boerwinkle et al., 2019a). This subgroup was selected due to its homogeneous etiology, and all of them had reduction of seizures >50% at the time of the follow-up scan. Additionally, their age range closely aligned with that of the SABI cohort, facilitating comparisons between the two groups and aiding in the interpretation of HF-BOLD findings in SABI.

Of the five SABI patients, who were 5–17 years old, four were male. The etiologies were as follows: two severe traumatic brain injury (TBI), one cardiac arrest, one autoimmune with supratentorial multifocal intraparenchymal hemorrhage, and one infratentorial secondary spontaneous hemorrhage. The first rs-fMRI (rs-fMRI#1) occurred on respective hospital days 2, 8, 15, 17, and 20; rs-fMRI#2 occurred on days 10, 13, 31, 55, and 90+; and rs-fMRI#3, which occurred in two patients, was on days 63 and 238. Herein called SzNET were identified in all the initial rs-fMRIs. Antiseizure medication (ASM) was given to all five SABI patients.

### 2.2 Clinical description

#### 2.2.1 Case 1

A 17-year-old man with muscular dystrophy, who was 1 year post-heart transplant, was hospitalized for transplant rejection. Subsequently, he experienced a cardiac arrest. The EEG showed no seizures but did show generalized periodic discharges. However, he was given ASM for subcortical myoclonus. The initial MRI showed diffusion restriction largely isolated to the basal ganglia. He was in a coma, and on day 4, he worsened as his pupils became non-reactive. However, repeated head imaging did not show a mass effect to explain the deterioration, and EEG also remained without seizures. Still comatose, his day 15 rs-fMRI#1 showed relatively typical RSN and temporal-opercular SzNET. Thus, ASM was escalated, but he did not improve. The day 31 rs-fMRI#2 showed a worsening of SzNET and RSN. Thus, EEG was restarted, with new frequent seizures, which resolved with further ASM escalation. By day 63, the rs-fMRI#3 showed reduced SzNET and improved RSN, and he was tracking without language, thus minimally conscious state (MCS) minus. Despite this neurological improvement, due to systemic complications the family then opted for WLST.

#### 2.2.2 Case 2

A 5-year-old healthy male was found at home posturing with an initial Glasgow Coma Score (GCS) of 4. The CT showed large bilateral intraparenchymal hemorrhages and normal angiography. He received an intracranial pressure (ICP) bolt, extra-ventricular drainage, and a left decompressive hemicraniectomy. However, the ICP remained high and thus had a day 4 right decompressive hemicraniectomy. The brain biopsy showed acute hemorrhagic leukoencephalitis. With continued high ICP and coma, the day 14 MRI showed progressive edema and hemorrhages. The ongoing EEG through this time showed generalized periodic discharges and ASM was empirically given. On day 20 only, he had electrographic seizures that resolved with further ASM escalation, but by day 30, he continued to oscillate between coma to periods of simple command following, congruent with MCS plus. His rs-fMRI#1 at that time showed SzNET in the temporal region, and atypical diencephalon region RSN, for which he was already on multiple high-dose ASM, which were maintained. His examination continued to improve with a resolution of unresponsive periods. On day 55, rs-fMRI#2 showed improved RSN and improved but still present SzNET. Then, one of three ASMs was weaned, and by 90 days, he remained on MCS but hemiplegia was present. However, by day 110, he was having a return of episodic reductions of consciousness with non-ictal EEG. The rs-fMRI#3 showed RSN and continued bitemporal SzNET. ASM maintenance was temporarily escalated again with improvement.

#### 2.2.3 Case 3

A healthy 11-year-old male presented with a sudden headache and a GCS of 7. His CT showed compressive cerebellar hematoma. He was taken for an emergency extra-ventricular drainage and hematoma evacuation. The day 2 angiogram showed an arteriovenous malformation. He remained without arousal, thus requiring day#14 tracheostomy. EEG taken in these early days had brief seizures that subsided with ASM loading dose alone. Moreover, through day 17, the EEG continued to show delta slowing without a seizure. The day 17 MRI showed late posterior fossa infarcts and rs-fMRI#1 showed normal RSN, but largely frontal SzNET. This was treated with ASM. By day 53, he followed simple commands consistently (MCS plus) and had quadriplegia. A 5-month rs-fMRI#2 showed the resolution of SzNET and normal RSN. Five years after injury, he had normal cognition and was independent in all daily activities.

#### 2.2.4 Case 4

A 6-year-old male had severe TBI, cervical spine injury, and coma. The day 2 EEG showed slowing without seizures and MRI showed evidence of diffuse axonal injury and severe brainstem and upper spinal cord injury. The day 2 rs-fMRI#1 showed only the visual RSN and SzNET. ASM was then given. The coma persisted and day 10 rs-fMRI#2 showed the typical RSN, and nearly resolved SzNET. On the 10^th^ day, the task-based fMRI demonstrated covert consciousness through brain activation in response to commands. At 18 months was performing age-level school academics, though quadriplegic.

#### 2.2.5 Case 5

An 8-year-old healthy female had severe TBI and a GCS of 6. ASM was initiated for posturing. The CT showed subarachnoid hemorrhage, and an MRI informed diffuse axonal injury. The 1^st^ week of EEG was non-ictal with slowing and generalized sharp waves. The day 8 rs-fMRI#1 showed the RSNs but with fronto-temporal SzNETs. Thus, ASM was given. Still in a coma, the day 13 rs-fMRI#2 showed substantial resolution of SzNETs, and task-fMRI showed covert consciousness. By day 25, she was following commands during the examination. At 4 months, she was independent in daily living activities for age.

### 2.3 Rs-fMRI analysis

#### 2.3.1 Data acquisition

Images were acquired with subjects' eyes closed, using minimal or no sedation, on 3 Tesla scanners (ingenuity; Philips Medical Systems and MAGNETOM Prisma; Siemens Healthineers). The rs-fMRI consisted of echo-planar images (TR = 2020 ms, TE = 32 ms, matrix size 80 × 80, flip angle 80°, number of slices 46, slice thickness 4 mm, no gap), totaling 600 volumes acquired over two 10-min runs (the second run performed approx. 5 min after the first run). Additionally, a T1-weighted image was obtained for registration (TR 1.9 ms, TE 2.5 ms, flip angle 9°, slice thickness 1 mm, in-plane resolution 0.9 × 0.9 mm).

#### 2.3.2 Preprocessing

Standard preprocessing steps were applied to the rs-fMRI data, including removal of non-brain structures, deletion of the first five volumes, motion correction using FSL's MCFLIRT (Jenkinson et al., 2002), inter-leaved slice time correction, high-pass filtering at 100 s, and non-smoothed. In our study, all subjects exhibited head displacement of < 1 mm in any direction. Individual functional scans were then linearly registered to each subject's high-resolution T1 scan using linear registration (Jenkinson et al., 2002), and optimization was achieved through boundary-based registration (Greve and Fischl, 2009).

### 2.4 HF-BOLD quantification

#### 2.4.1 Defining SzNET

**Figure 1:**
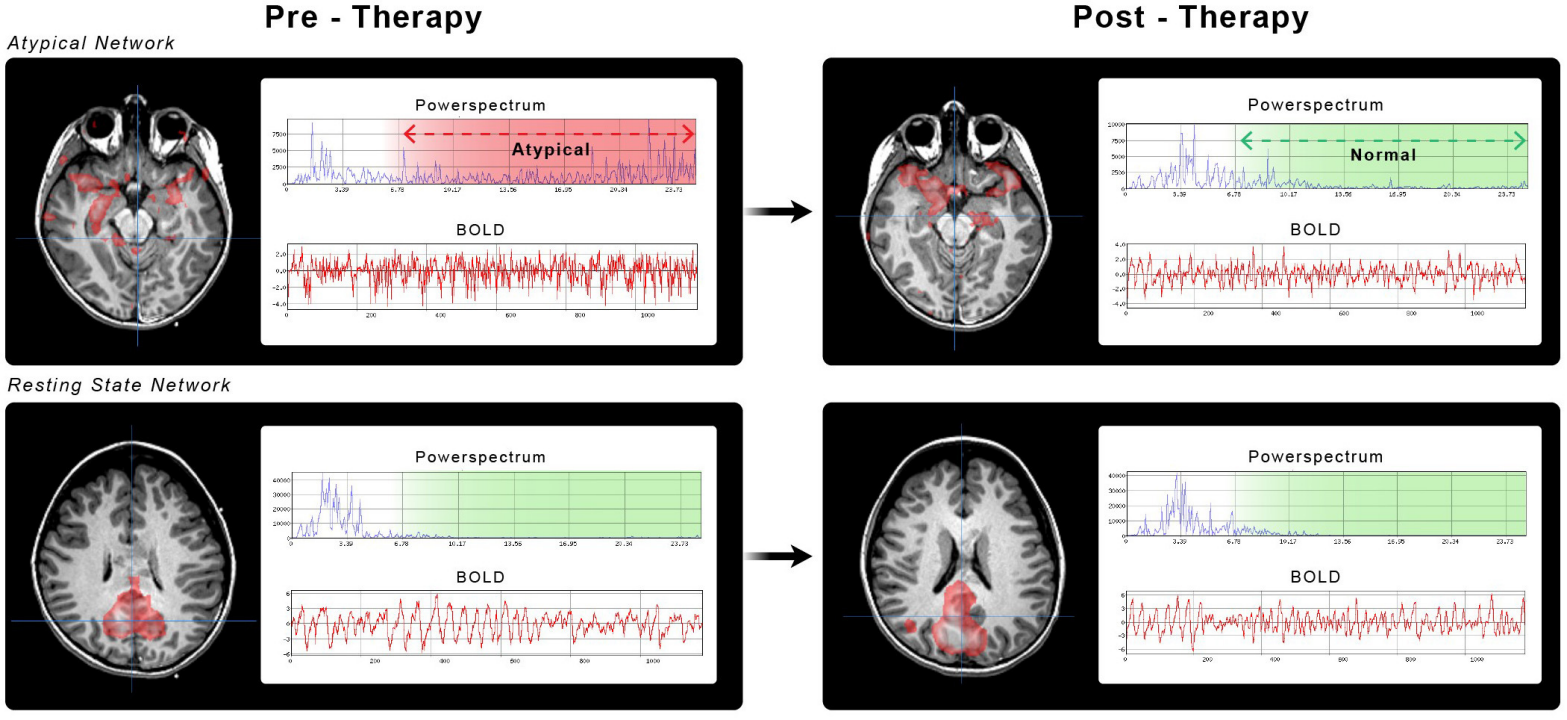
Brain networks from case number 5. Pediatric ICU patient with TBI GCS of 6 and ictal-negative EEG. All the images show ICA-identified brain networks with their corresponding power spectrum (blue) and BOLD signals (red). **(A)** SzNET with its characteristic atypical power spectrum over high frequencies. **(B)** Post-ASM SzNET in homologous localization with comparatively reduced power spectrum over the frequencies of interest. **(C, D)** Depict the default mode network before and after ASM administration, respectively. A better spatial symmetry and smother BOLD oscillation can be observed compared to the SzNET. We can observe a greater change in the power spectrum of SzNET pre-to-post compared to the relatively normal default mode network. **(A–D)** Are positioned in a left to right top up to bottom order.

Rs-fMRI distinguishes individuals with epilepsy from normal controls (Jiang et al., 2022). The subclasses of signals resulting from independent component analysis (ICA), we utilize from our prior study, are RSN, SzNET, and noise. SzNET correlate with seizure location as determined by intracranial EEG (Boerwinkle et al., 2017; Chakraborty et al., 2020; Hunyadi et al., 2013), and targeting them in epilepsy surgery planning has been associated with better seizure outcomes in epilepsy surgery (Boerwinkle et al., 2019a). SzNET are distinguished from RSN and artifacts by differentiating spatial and temporal features observed in epilepsy and have been successfully identified by machine learning strategies (Bharath et al., 2019). Networks exhibiting similar features are also observed in SABI, as shown in Figure 1. Therefore, networks with such atypical features will be referred to as SzNET in SABI patients as well, by study definition, despite the lack of firm establishment. The RSN were further classified into the following categories: motor, frontal, parietal, language, vision, temporal, subcortical/infratentorial, default mode network, and long-range association network.

#### 2.4.2 SzNET identification

ICA was performed using MELODIC of the FSL Toolbox (Jenkinson et al., 2012) with the total number of independent components determined by automated Bayesian dimensionality (Beckmann et al., 2005). A standard local false discovery rate threshold of *p* < 0.05 was employed for IC detection (Beckmann et al., 2005). Only visually inspected high-quality data were used, and expert personnel conducted manual assessments to categorize the components as noise, RSN, and SzNETs, based on spatial and temporal features in epilepsy to replicate our previously validated method (Boerwinkle et al., 2017), supported on previous studies (Griffanti et al., 2017; Hunyadi et al., 2015). Henceforth, non-noise ICs will be referred to as networks.

#### 2.4.3 Quantifying HF-BOLD

To quantify the burden of atypical power spectrum of each network, an index here called *HF-BOLD* was computed based on spatial and temporal features. For each network, it is higher than 0.06 Hz power spectrum ratio was multiplied by its percentual volume from the volume of total networks in the same scan, as shown by Equation 1.


(1) $$\begin{array}{l} F_{i}=\frac{V_{i}H_{i}}{V_{t}P_{i}} \end{array}$$

where *F*_*i*_ represents the *HF-BOLD* of a specific network *i, V*_*i*_ denotes the number of voxels within that network, while *V*_*t*_ represents the total number of voxels across all networks in the scan. Additionally, *H*_*i*_ is the area under the curve of the power spectrum function above 6 Hz/100s, and *P*_*i*_ denotes the power spectrum's total area under the curve of the same network. For calculation script details, further information is available in the Appendix.

### 2.5 Statistical methods

The patients are grouped into two pathologies: epilepsy and severe acute brain injury. We characterize the demographics of our patients using descriptive statistics both overall and stratified by group. We used Hedges' g, the bias-corrected standardized mean difference with both conventional and bootstrapped 95% confidence intervals, to measure the change in HF-BOLD in SzNET or RSN corresponding to the intervention. These were computed for both epilepsy and SABI patients. To compare the changes in HF-BOLD from before to after, we retain the HF-BOLD from each category of network (e.g., visual or motor) within each patient from rs-fMRI scans #1 and #2. The differences are simply #2 minus #1. These are modeled with a linear mixed-effects model with the group as the only fixed effect and random effects for patient and network category. To determine whether there are differences in baseline HF-BOLD values between the categories that differ between groups, we subsequently fit a linear mixed model (LMM) with network category also as a fixed effect and an interaction between group and category. The test of the interaction assesses whether the pattern of baseline HF-BOLD varies by category. The same LMM was applied to the changes across the categories between the groups. Finally, we assess whether there are different patterns of changes from #1 to #2 between the groups in networks that are classified as SzNET or RSN. Here, we model the differences again with an LMM that includes an interaction between group and network type. All our hypotheses are tested using likelihood ratio tests, and an alpha of 0.05 is used as the threshold for significance.

## 3 Results

The demographic characteristics of both patient groups are summarized in Table 1. Table 2 provides a qualitative summary of networks and neurological reactivity in SABI patients, with additional case details in Section 2.2. The mean age at the first rs-fMRI scan was 10.1 years for the epilepsy group and 9.4 years for the SABI group. Among the epilepsy group, 40% had developmental delay, and all had normal consciousness before the scan. In contrast, all SABI patients had typical neurological development and consciousness before hospitalization, with a GCS < 9 at the time of the first rs-fMRI. The mean number of ASM at the time of the first rs-fMRI was two for the epilepsy group and one for the SABI group, with no significant difference between the groups.

**Table 1:** Patient characteristics.

**Characteristics**	**Overall**	**Epilepsy**	**SABI**	***P***
No. of subjects	15	10	5	
Seizures onset age	51.8 (59.9)	19.1 (23.0)	117.2 (57.8)	< 0.001^*^
Age^†^	118.9 (61.4)	119.8 (66.2)	117.2 (57.8)	0.943
Female sex (%)	6 (40.0)	5 (50.0)	1 (20.0)	0.58
**Handedness (%)**				0.006^*^
Left	3 (20.0)	3 (30.0)	0 (0.0)	
Right	8 (53.3)	7 (70.0)	1 (20.0)	
Unknown	4 (26.7)	0 (0.0)	4 (80.0)	
Developmental delay—no. (%)	4 (26.7)	4 (40.0)	0 (0.0)	0.231
**Neurological deficit—no. (%)**				< 0.001^*^
Language only	1 (6.7)	1 (10.0)	0 (0.0)	
Multiple	5 (33.3)	0 (0.0)	5 (100.0)	
None	9 (60.0)	9 (90.0)	0 (0.0)	
**Admission GCS (%)**				< 0.001^*^
13–15	10 (66.7)	10 (100.0)	0 (0.0)	
3–8	5 (33.3)	0 (0.0)	5 (100.0)	
**Main seizure semiology (%)**				0.001^*^
Focal	6 (40.0)	6 (60.0)	0 (0.0)	
Generalized	1 (6.7)	0 (0.0)	1 (20.0)	
Second. Generalized	4 (26.7)	4 (40.0)	0 (0.0)	
Not documented	4 (26.7)	0 (0.0)	4 (80.0)	
**Number of ASM**^†^ **(%)**				0.615
0–1	6 (40.0)	3 (30.0)	3 (60.0)	
2–3	7 (46.7)	5 (50.0)	2 (40.0)	
4+	2 (13.3)	2 (20.0)	0 (0.0)	
Previous epilepsy surgery (%)	3 (20.0)	3 (30.0)	0 (0.0)	0.505
**Etiology (%)**				0.001^*^
Focal cortical dysplasia	9 (60.0)	9 (90.0)	0 (0.0)	
Heterotopia	1 (6.7)	1 (10.0)	0 (0.0)	
Hypoxic ischemic	1 (6.7)	0 (0.0)	1 (20.0)	
ICH	3 (20.0)	0 (0.0)	3 (60.0)	
TBI	1 (6.7)	0 (0.0)	1 (20.0)	

Age is in months. Continuous variables (e.g., age) are summarized with means and SDs in parentheses. Categorical variables (e.g., female) are described with counts and percentages in parentheses. ^†^At First rs-fMRI, ^*^significant *p*-value. GCS, Glasgow Coma Scale; ASM, antiseizure medication; ICH, intracranial Hemorrhage; TBI, traumatic brain injury.

**Table 2:** Qualitative network and examination change with antiseizure medication.

**Case**	**EEG seizures**	**ASM**	**Rs-fMRI#1**		**ASM**	**Examination**	**Rs-fMRI#2 change**		**EEG seizures**	**ASM**	**Examination**	**Rs-fMRI#3 change**		**Summary and interpretation**
			**RSN**	**SzNET**			**RSN**	**SzNET**				**RSN**	**SzNET**	
**1**	GPDS	**+**	**+**	**+**	**↑**	**=**	**+**	**+↑**	**+**	**↑**	**↑**	**+↑**	**+↓**	Initial prophylactic ASM. The worsening of SzNET on rs-fMRI#2 was consistent with the transition to electrographic seizures. After ASM escalation due to seizures, Rs-fMRI#3, EEG, and neurological examination improved.
**2**	+	**+**	**+**	**+**	**=**	**↑**	**-**	**+↑**	**-**	**↓**	**↓**	**+ = **	**+↑**	No ASM changes after rs-fMRI#1 and scan #2 worsened despite clinical improvement. After ASM reduction, Rs-fMRI#3 worsened, and neurological symptoms reappeared.
**3**	+	**+**	**+**	**+**	**↑**	**↑**	**+**	**+↓**			**↑**			Rs-fMRI#2 improved, and ASM was increased prior.
**4**	-	**-**	^*^	**+**	**↑**	**=**	**+↑**	**+↓**			**↑**			Rs-fMRI#2 improved, and ASM increased prior. Task-fMRI at the time of Rs-fMRI#2, with command response or covert consciousness.
**5**	GSW	**+**	**+**	**+**	**↑**	**=**	**+**	**+↓**			**↑**			Rs-fMRI#2 improved, and ASM increased prior, in covert consciousness by task-fMRI

EEG seizures, EEG with electrographic seizure or epileptiform activity as specified; Examination, neurological examination referring to the degree of reactivity to external stimuli; Strong green background means consistency, and light red background means inconsistency between the clinical changes and the rs-fMRI changes. Arrow symbol meanings are compared to the previous observation on the same modality: ↓, decrease; ↑, increase; =, no change. Other symbols: +, present; -, absent; ^*^, only 1 RSN detected. ASM, antiseizure medications; GPDS, generalized periodic discharges; GSW, generalized sharp waves.

In the SABI cohort, following treatment including ASM, neurological improvement was observed between rs-fMRI#1 and #2 in patient 2 and patient 3. Additionally, after rs-fMRI #2, neurological improvement was noted in all patients except patient 2. After ASM reduction, patient 2 developed new neurological symptoms and also exhibited an increase in SzNET in his third rs-fMRI. In comparison with rs-fMRI#1, the SzNET improved in all patients except patient 1. In this case, the rs-fMRI findings were congruent with no initial clinical improvement, and epileptogenic activity reappeared on EEG. Subsequent adjustments to ASM were made, resulting in both clinical and connectivity improvements.

**Figure 2:**
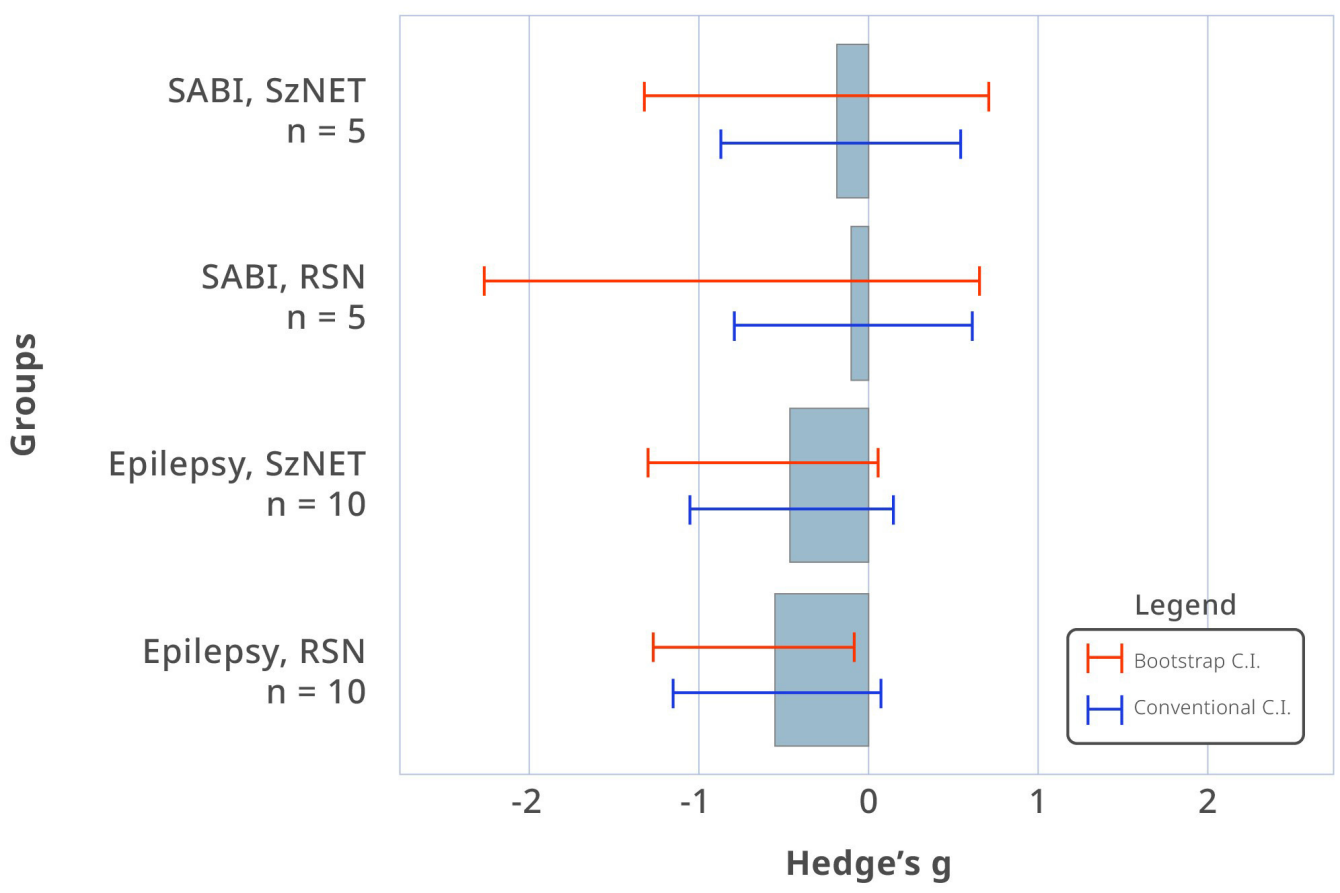
Bias-corrected standardized mean differences in HF-BOLD (post–pre) for SzNET and RSN in SABI and epilepsy patients. The red lines are bootstrapped confidence intervals (CI), whereas the blue lines are conventional CI assuming normality. Confidence intervals that do not include 0 are significant by that method. Only epilepsy RSN shows a statistically significant HF-BOLD reduction, and only when assuming normality.

Comparison of the baseline HF-BOLD pattern across different categories did not show sufficient evidence to observe a difference between the etiologies (χ^2^ = 11.0, df = 11, *p* = 0.44). In the epilepsy group, the median HF-BOLD changes were −0.032 for SzNET and −0.047 for RSN. For the SABI group, the median HF-BOLD changes were −0.054 for SzNET and −0.037 for RSN. We quantified how the HF-BOLD of networks changed from the earliest to the latest rs-fMRI using bias-corrected standardized mean differences. The evolutive change in the SZNET's HF-BOLD was −0.52 (95% CI −6.14 to 0.34) for the epilepsy group and −0.17 (95% CI −1.31 to 0.72) for the SABI group. Similarly, RSN's HF-BOLD evolutive change was −0.78 (95% CI −3.42 to −0.33) for the epilepsy group and −0.09 (95% CI −2.26 to 0.67) for the SABI group, as shown in Figure 2. When observing this HF-BOLD evolution by network category, the mean HF-BOLD differences decreased after the intervention across all network types in both etiology groups, except for the atypical RSN category in the epilepsy group, which had a least square mean of 0.01. In the multivariate analysis of HF-BOLD change after treatment, no statistical differences were found between the etiologies overall (χ^2^ = 2.12, df = 1, *p* = 0.15), by the pattern of different network types (χ^2^ = 6.53, df = 10, *p* = 0.77), or by subgroups of SzNET and RSN (χ^2^ = 0.68, df = 1, *p* = 0.41).

## 4 Discussion

The study presents rs-fMRI data from two time points, pre-and-post-therapy in the medical evolution of SABI and epilepsy patients. Given the tendency of SzNET in epilepsy patients to exhibit a higher power spectrum and improve consistently with seizure frequency, we utilized a power spectrum-based measure called HF-BOLD. Despite observing HF-BOLD reduction in over 50% of cases, this exploratory study only found a statistically significant reduction in the HF-BOLD of grouped RSN from epilepsy patients. Neither the grouped SzNET of epilepsy patients nor any network group from SABI patients showed statistically significant HF-BOLD reduction, possibly due to the small cohort sizes or pathological heterogeneity.

Across the literature, abnormalities in SABI rs-fMRI are broad, ranging from increased functional connectivity to generalized or focal dysconnectivity. These differences have been attributed to the heterogeneity of lesions and the methods used (Caeyenberghs et al., 2024; O'Neill et al., 2017; Sair et al., 2018). Interhemispheric dysconnectivity has been recurrently reported in TBI, potentially leading to asymmetry in ICA-identified networks (Ordóñez-Rubiano et al., 2024; Tang, 2012). Therefore, we considered exploring methods to reduce the dimensionality of rs-fMRI data to an index suitable for small sample sizes, while still including network volume in this index.

The study provides insight into the evolution of networks observed in rs-fMRIs of SABI patients, offering new elements for understanding functional connectivity findings and their implications for brain abnormal network activity. Quantitative and reproducible measures that detect changes in function of networks could prove invaluable in this regard. However, it is currently uncommon to acquire a second rs-fMRI in SABI patients, thereby limiting the validation of its results. While seed-based analysis and network theory measures have been informative in predicting consciousness recovery, an ICA approach quantifying SzNET features may offer enhanced precision-based detection of network dysfunction across the whole brain, aiding therapeutic interventions.

Disease variability inherent to each etiology, such as differences in GCS at admission, were noted between the cohorts. However, there was no significant difference in baseline HF-BOLD patterns between the two groups, although this may again be attributed to the small group sizes. We acknowledge that the anatomical distribution of brain lesions in SABI varies according to the injury mechanism (Zhou, 2021). For instance, traumatic contusions are predominantly located near the frontal and temporal poles, while hypoxic–ischemic lesions are primarily found in transition areas between major vascular territories and specific neuronal populations, such as Purkinje cells (Busl and Greer, 2010; La Rocca et al., 2021). In contrast, epilepsy more frequently affects a different set of regions, with a high prevalence of mesial temporal compromise and anatomical regions involved with the DMN, the frontoparietal network, and the ventral attention network where lesion emergence has a higher likelihood of causing seizures (Mansouri et al., 2020). Larger sample sizes could potentially reveal baseline differences by network type between the SABI and epilepsy groups, particularly in relation to the specific RSNs that overlap with these distinct regions. Multivariate analysis did not yield sufficient evidence to conclude differences in HF-BOLD evolution by network categories or grouped categories between the two etiologies, again suspected to be due to low statistical power. These findings may be due also to factors such as multifactorial SABI network dysfunction worse than epileptogenic activity alone or variations in the injury evolution times.

In conclusion, gaining insight into the evolution of networks observed in rs-fMRIs of SABI patients holds promise for elucidating connectivity findings that could inform therapeutic interventions. While the study highlights interesting trends related to the power spectrum of networks, limitations stemming from small sample sizes and heterogeneity of events between initial and follow-up scans underscore the need for larger, more comprehensive studies to confirm and further explore these findings. In the absence of large datasets to perform such studies, qualitative rs-fMRI analysis can provide insight into the evolution of network dysfunction in the individualized clinical context.

## Data Availability

The original contributions presented in the study are included in the article/Supplementary material, further inquiries can be directed to the corresponding author.
