# Supplementary material for: Pre- and post-therapy functional MRI connectivity in severe acute brain injury with suppression of consciousness: a comparative analysis to epilepsy features
Source: Front Neuroimaging. 2024 Oct 1;3:1445952. doi: 10.3389/fnimg.2024.1445952 (PMC11473429; doi:10.3389/fnimg.2024.1445952)
Supplement: Supplementary file 3 [file Table_3.DOCX]

Resting State functional MRI Whole Brain Map

| **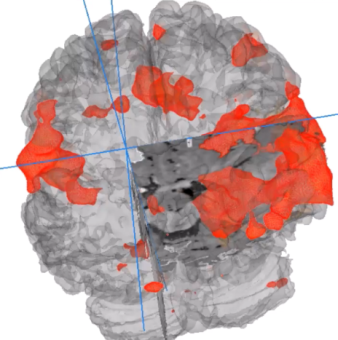** |  |  |
| --- | --- | --- |

PP2 MRI#2

Medications:

Anatomical MRI - see separate radiological report,

Technique and analysis Methods 3T MRI, whole brain BOLD ICA

Data Quality Analysis 20 min; Head motion < 1 mm, no interfering artifacts detected

Abbreviation index list at end of rs-report

Impression:

| 1. **Rs-fMRI seizure onset zone**: | Bilateral mesial temporal |
| --- | --- |
| 1. **Language**: | Split dominance with R dominant for expressive-frontal and left dominant for receptive-temporal; note BOTH L and R have STG-IFG in same unilateral network |
| 1. **Whole brain networks:** | Well detected |

| Rs-fMRI Informed Neuro-Prognostication | | |
| --- | --- | --- |
| Consciousness | Alertness and Awareness  Equivalent of 0=Coma, 1=Normal Consciousness, 2=MCS, 3=VS/UWS | 1 |
| Developmental Stream | 0=very poor, 1=Normal, 2=Atypical but not very poor, 3=Very poor, 4=Indeterminate | |
| Motor | Walking | 1-R; 3-L |
|  | Gross motor body movement | On R 1, L-3 |
|  | L - arm/hand fine motor | 1 |
|  | R- arm/hand fine motor | 3 |
|  | face/mouth motor coordination | 1-R; 3-L |
| Motor tone | R appendicular tone | 1-2 |
|  | L appendicular tone | 1 |
|  | Central tone | 1 |
| Language | Understanding words | 1 |
|  | Speaking in Words | 1-2 |
| Vision | R field primary visual reception | 1 |
|  | R field higher level visual stimuli interpretation | 1 |
|  | L field primary visual reception | 1 |
|  | L field higher level visual stimuli interpretation | 1 |
| Sensory | Touch/Sense of Spatial/complex relationship | 1-2 |
| Cognition/Learning/Memory | equivalent IQ >70 | 1-2 |
|  | equivalent IQ < 70 |  |
|  | Profound Intellectual Disability Equivalent IQ < 35 |  |

* Scoring unless otherwise specified is shown as the possible highest score expected or range

| Network Characterization | | | | |
| --- | --- | --- | --- | --- |
| 1=yes, 0=no | Detected | Normal | Atypical | Comment |
| Motor | 1 |  | 1 | Normal temporal and spatial except leg is unilateral L and R  Not detected: L arm and L face region motor networks |
| Language | 1 | 1* |  | Bilateral and IFG-STG in same signal networks  (24) inc freq |
| Parietal | 1 | 1 |  |  |
| Frontal | 1 | 1* |  | Reduced L PFC activity compared to R |
| Temporal | 1 | 1 |  | Except as in atypical areas below |
| Vision | 1 | 1* |  | Except unilateral only |
| Deep grey | 1 | 1* |  | R > L, but bilateral which is typical |
| Modulating | 1 | 1 |  |  |
| Association | 1 | 1* |  | See atypical – clear LR-FTP found with some spatial and temporal irregular features |
| Atypical/possible pathological | Summary: 3 B T SOZ; RSN with greated disruption in temporal > DMN > frontal networks, which is an indirectly supportive and localizing RSN feature to the temporal SOZ location.  (17) B MT-AT deactivation, high freq  (22) MT-AT high freq  (73) R > L AT inc freq  RSN with downstream connectivity disruption  (11) B DMN – deactivation high freq  (39) B LR-FTP deactivation with high freq  (24) B Language – high freq  (30) B lateral temporal high freq  (36) B parietal mod high freq  (57) B STG mod high freq  (75) B R > L PFC mild to mod inc freq  (74) R PFC mild to mod inc freq | | | |

**Prior Resting State Findings:**

**10/21**

1. Rs-fMRI seizure onset zone: No clear SOZ. The atypical networks located in the midbrain and thalamus can prevent expression of consciousness. The atypical network in the R mesial temporal regions indicates irritzation in this region but it is not to the extent causing multiple overlapping abnormal SOZ-suspicious networks at this time.
2. Language: The left superior temporal gyrus is in the same network as the left inferior frontal gyrus, despite the local space occupying lesion between them, which is remarkable. This means that it may be possible that this network could survive yet still and support language function. The right language networks does not currently have both location in one network, but the right side maintains its long-range fronto-parietal network, so there is still good A-P direction connection there.
3. Whole brain networks: The most likely deficits anticipated by the rs-fMRI are R arm relative weakness. Partial visual field deficit in L field. The language function may return to a significant but unknown proportion as prior with comprehension more assuredly recovering and to some extent still possible to have expressive language if no further damage occurs. Expect full return of consciousness or even possibly to some degree covertly conscious now.

**11/15**

1. Rs-fMRI seizure onset zone: possible frontal-insular-temporal
2. Language: well detected, L dominant likely
3. Whole brain networks: relatively well detected and consistent with prior rs-fMRI

| **Atypical Networks** |
| --- |

| 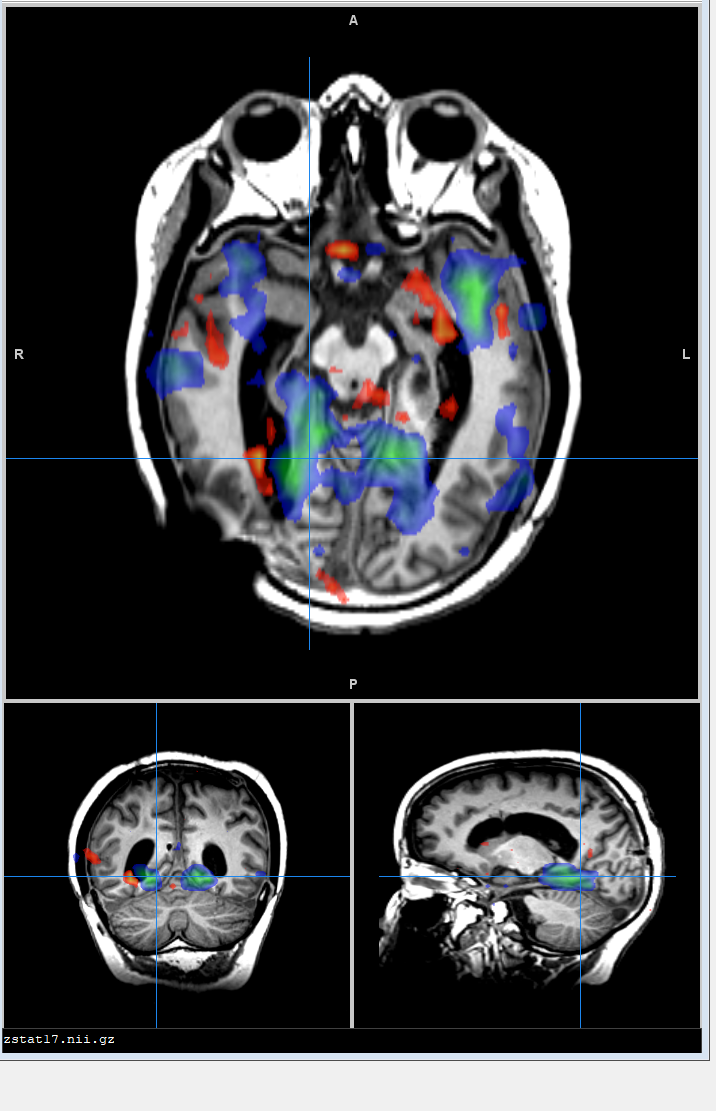 zstat17 | 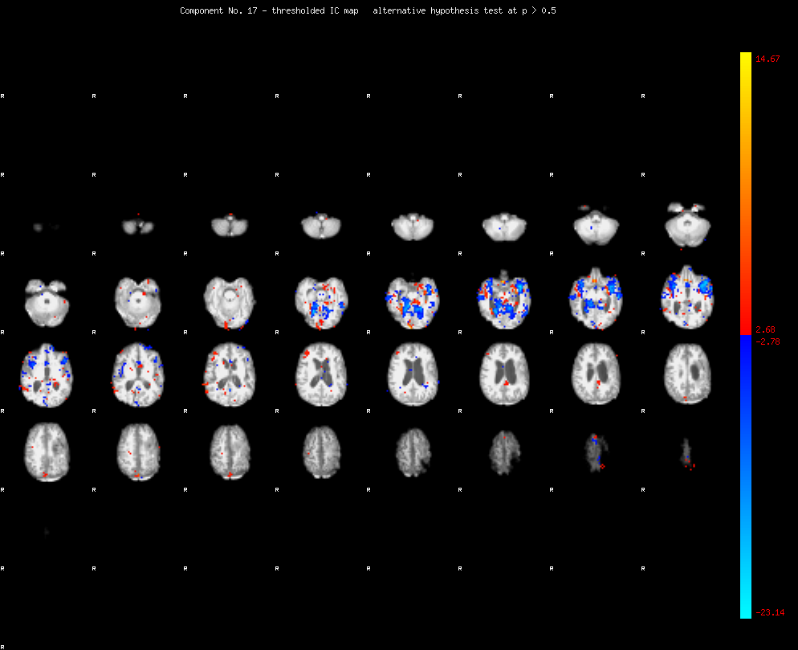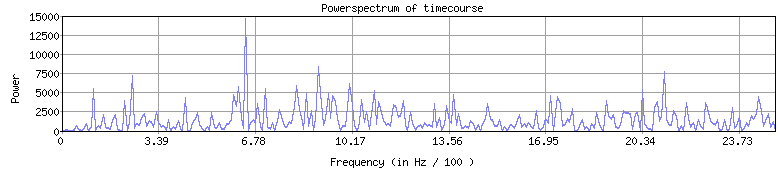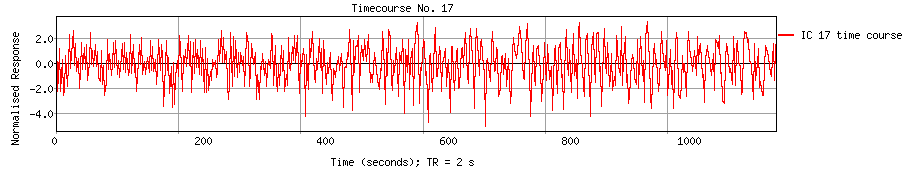 |
| --- | --- |
| 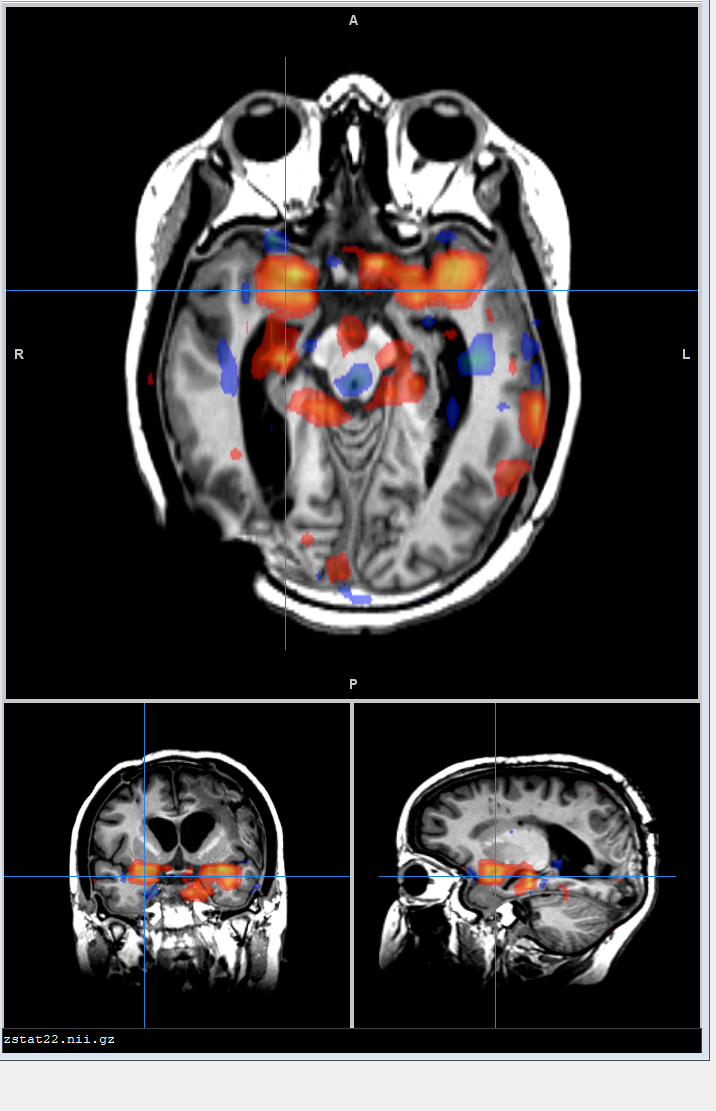 zstat22 | 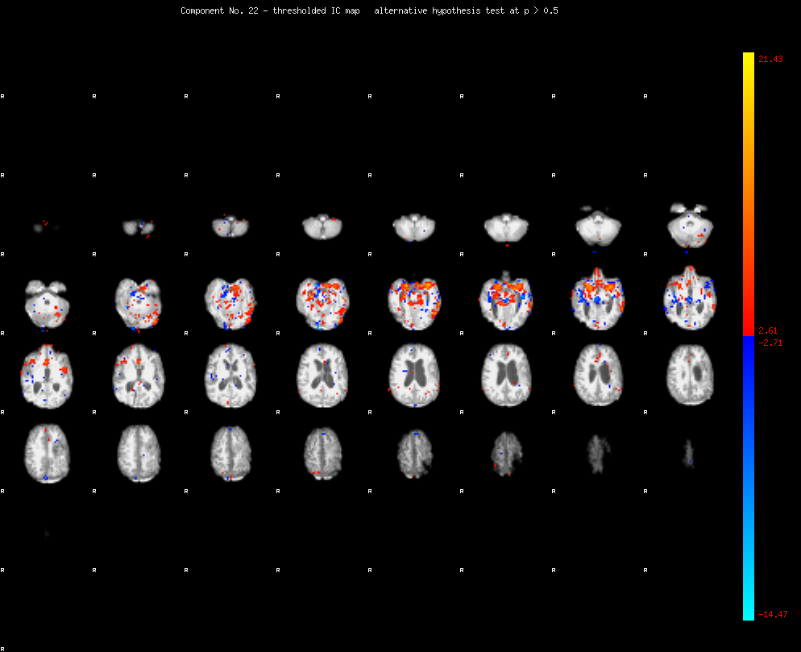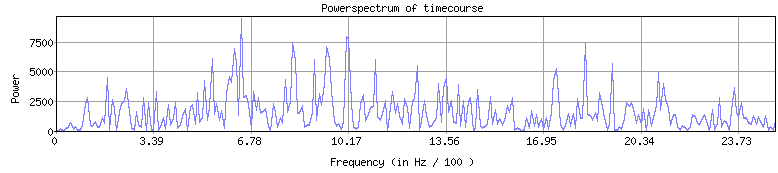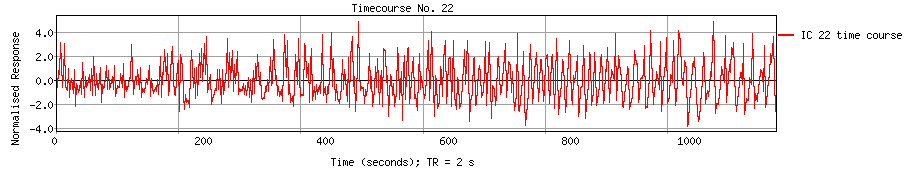 |
| 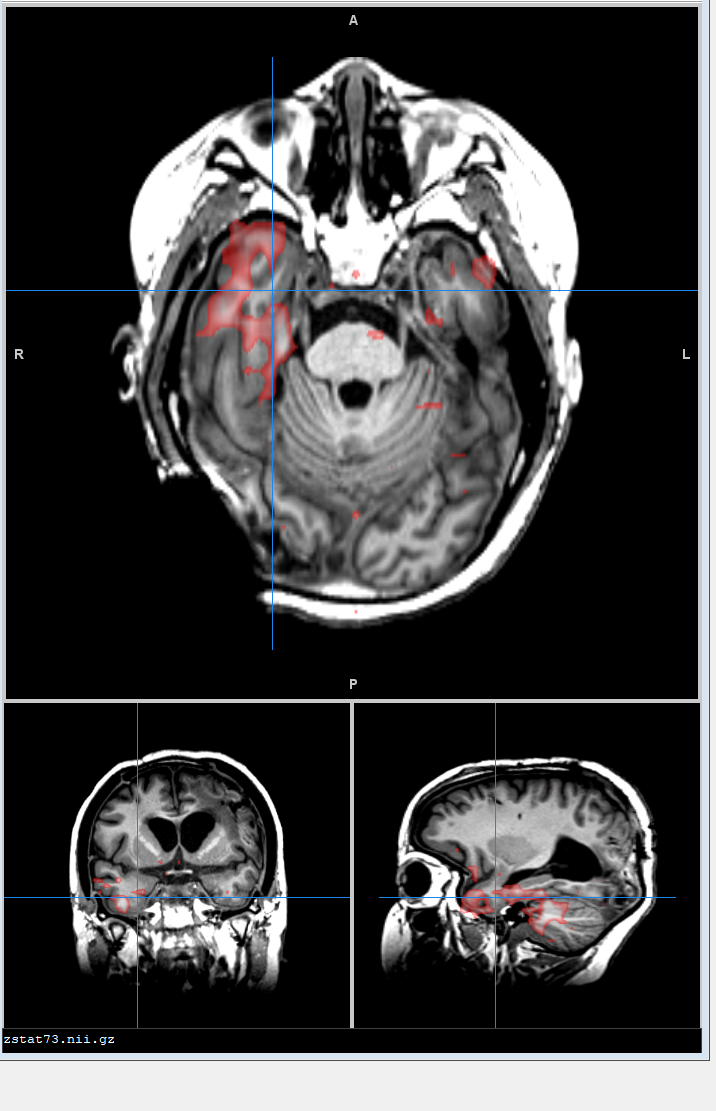 zstat73 | 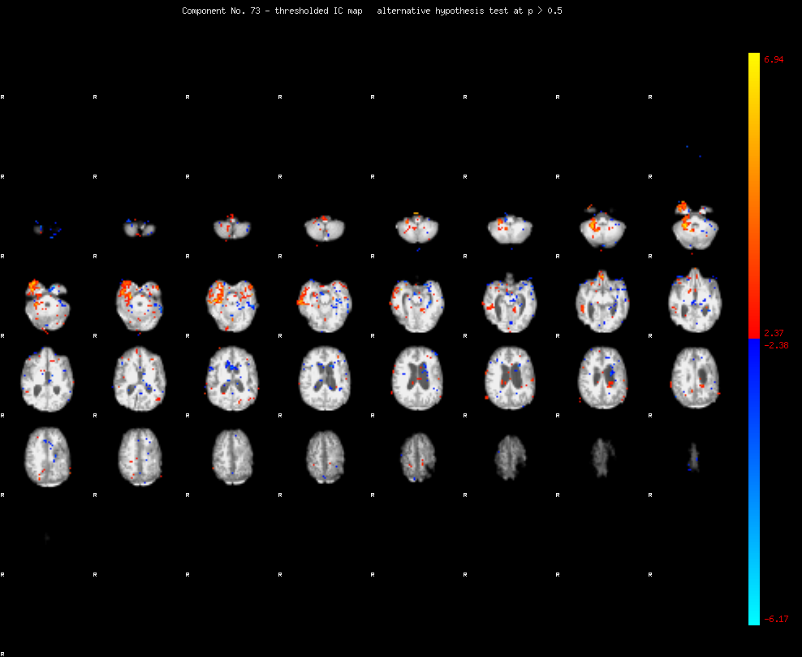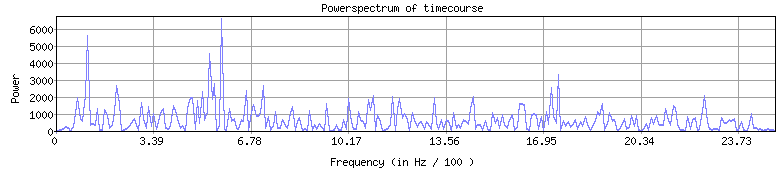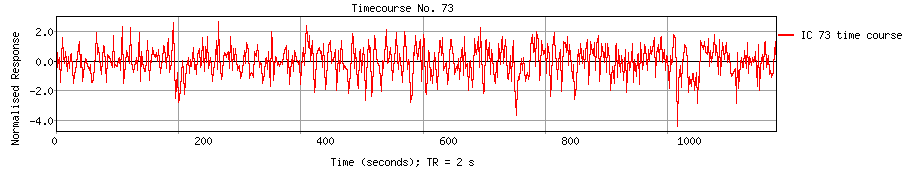 |

| 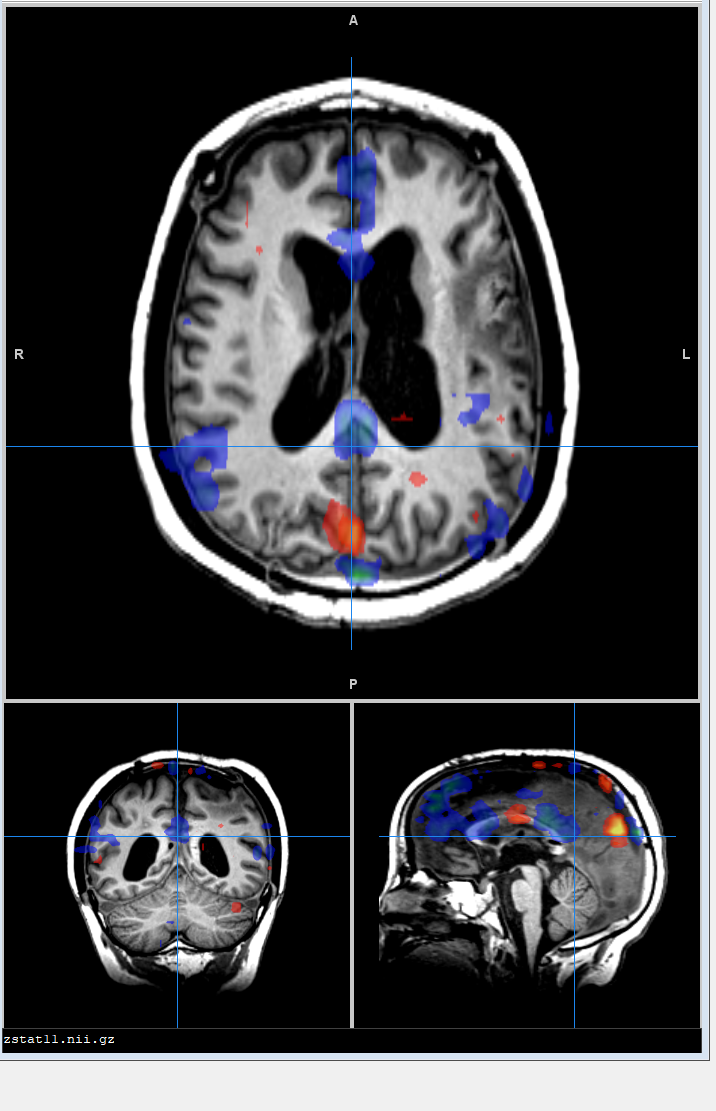 zstat11 | 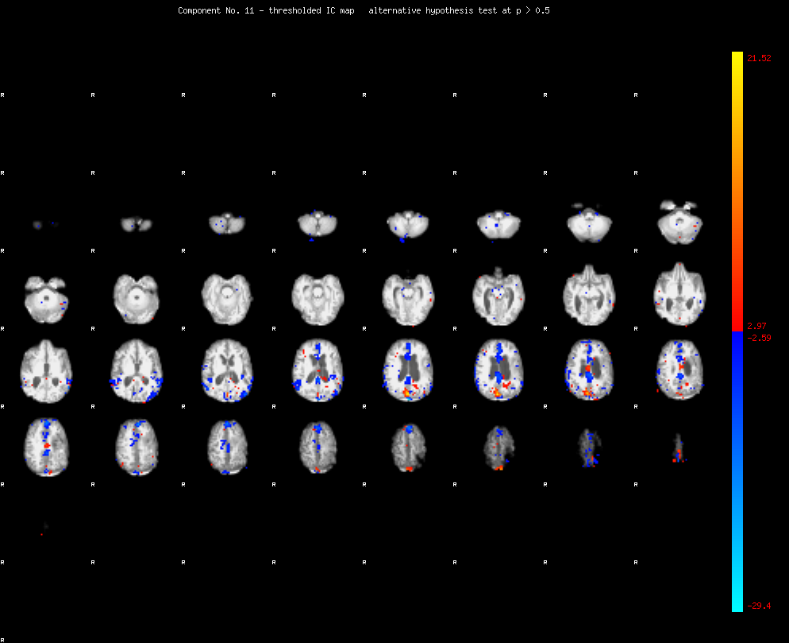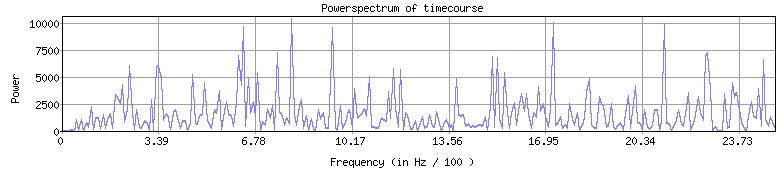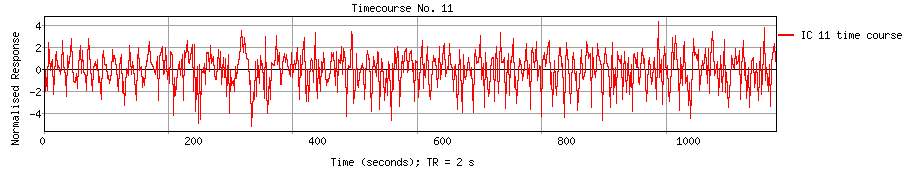 |
| --- | --- |

| 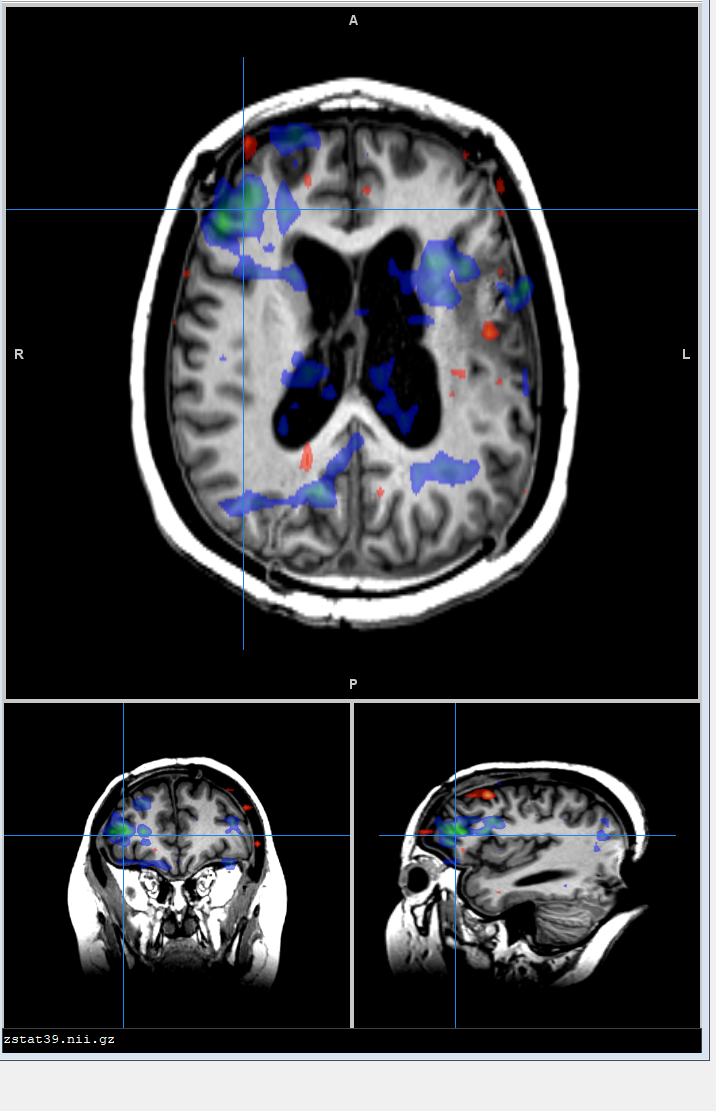 zstat39 | 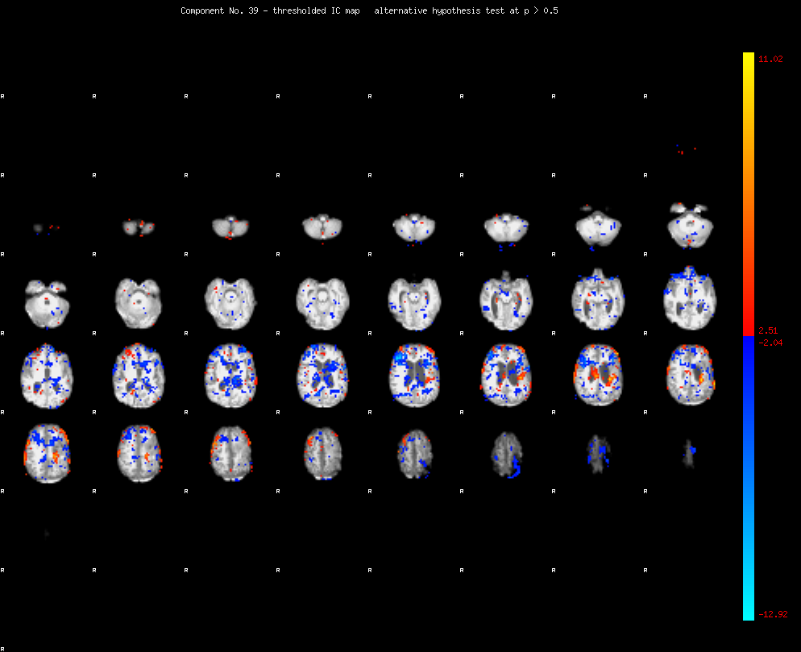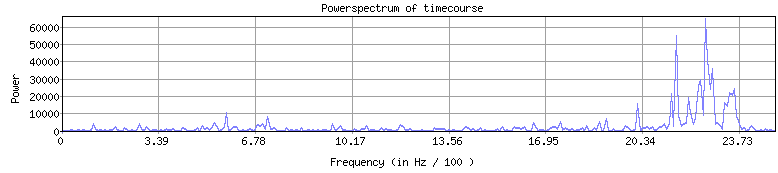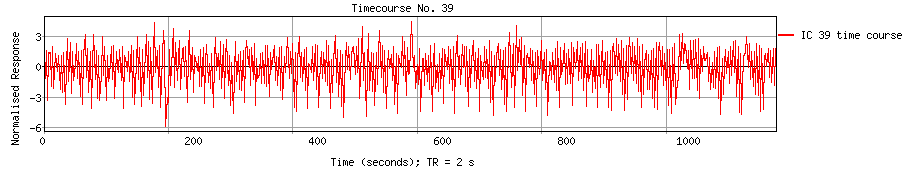 |
| --- | --- |
| 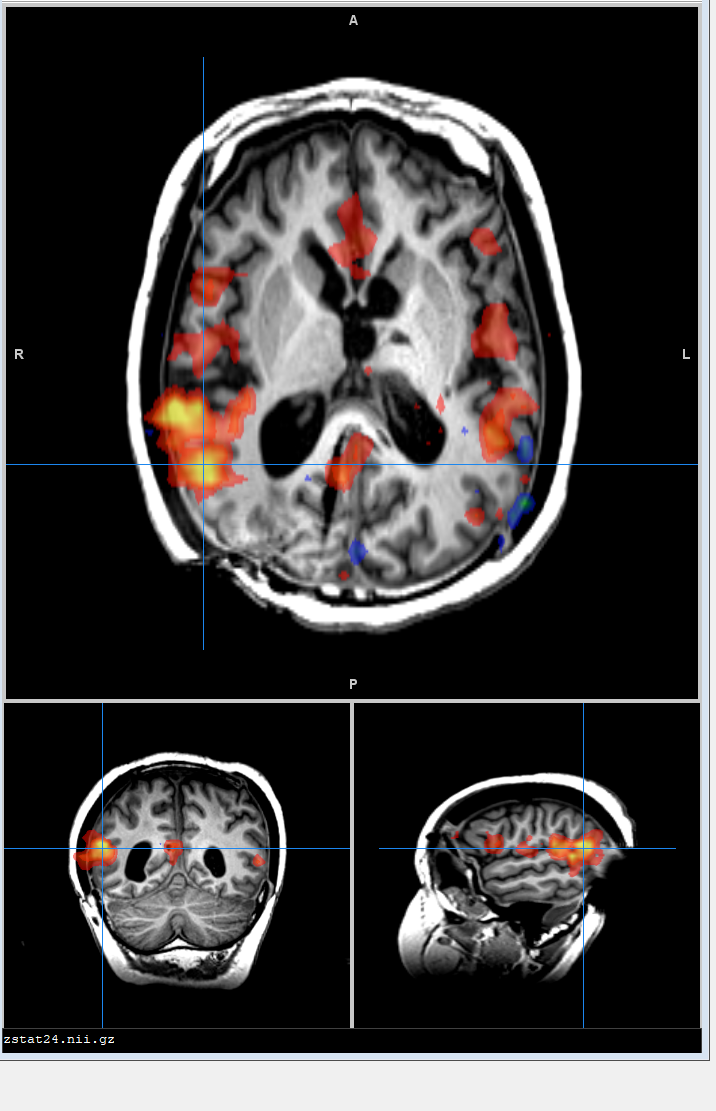 zstat24 | 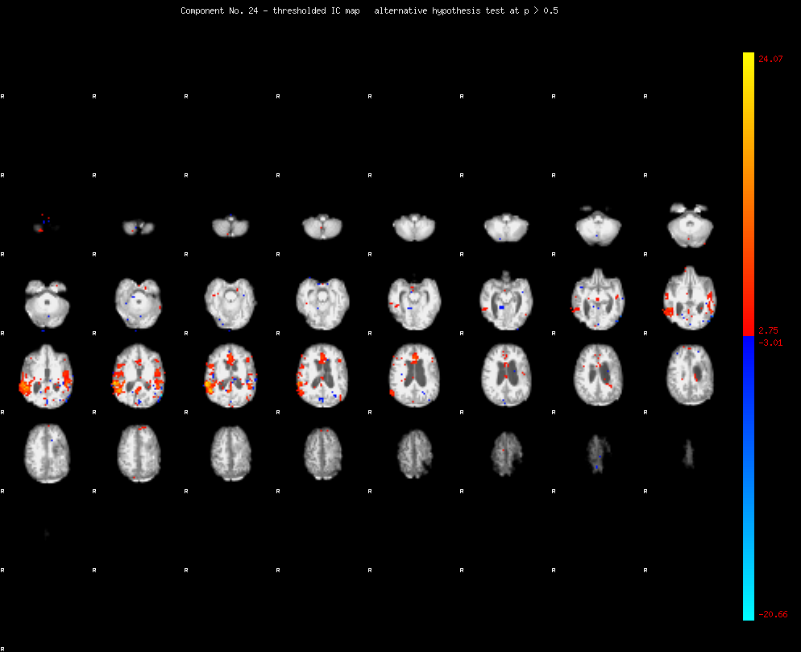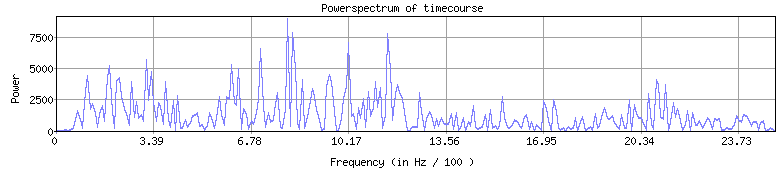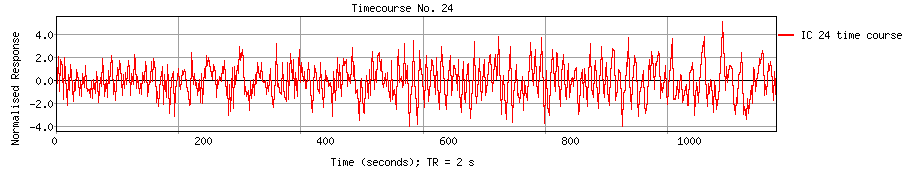 |
| 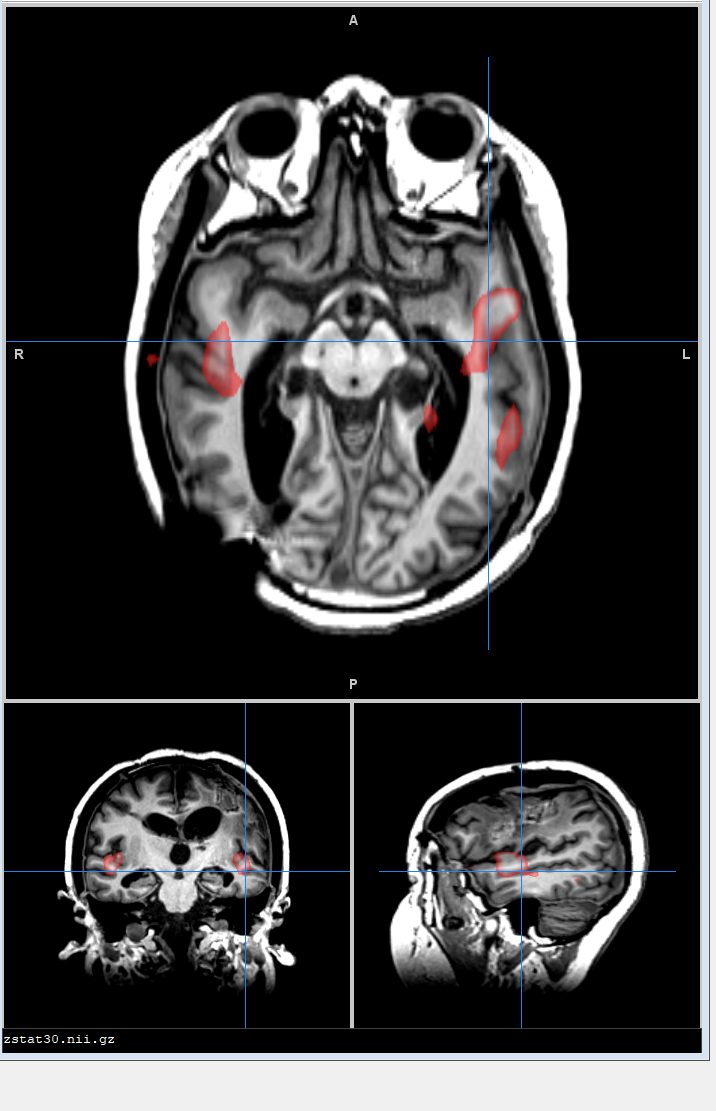 zstat30 | 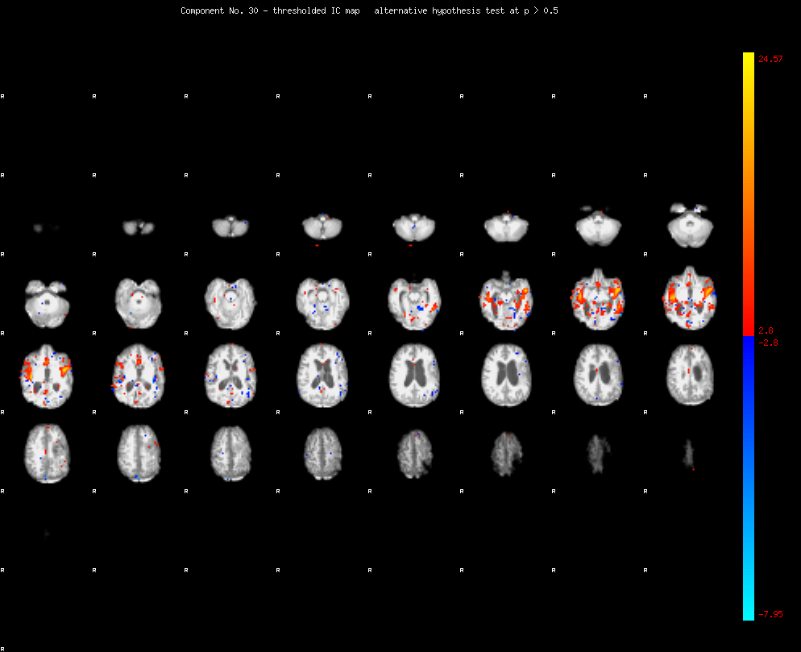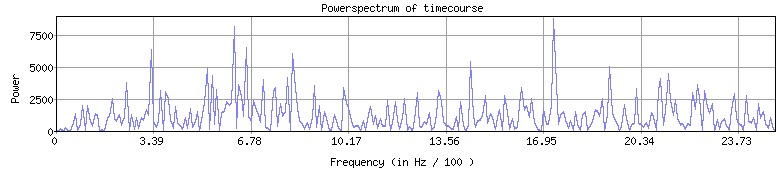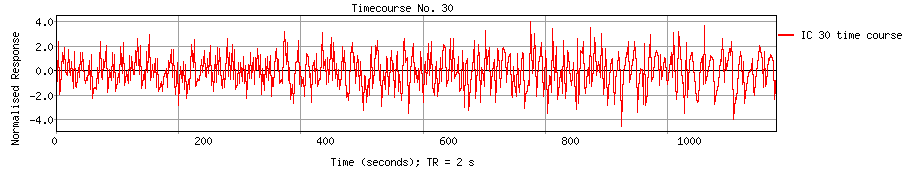 |
| 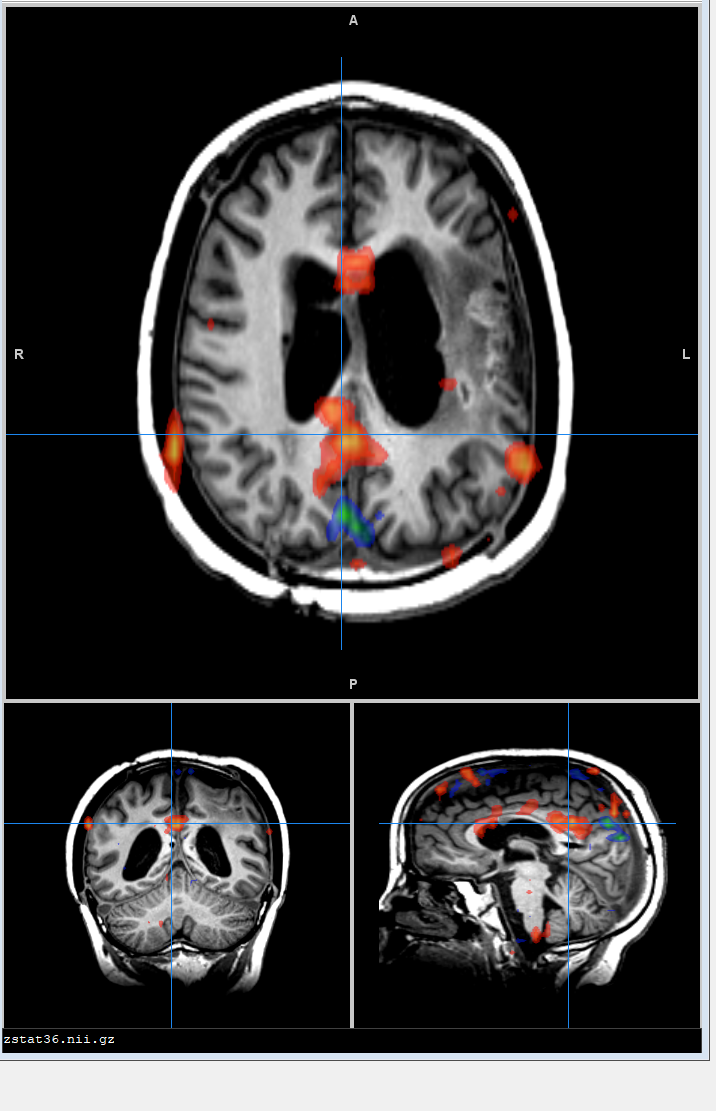 zstat36 | 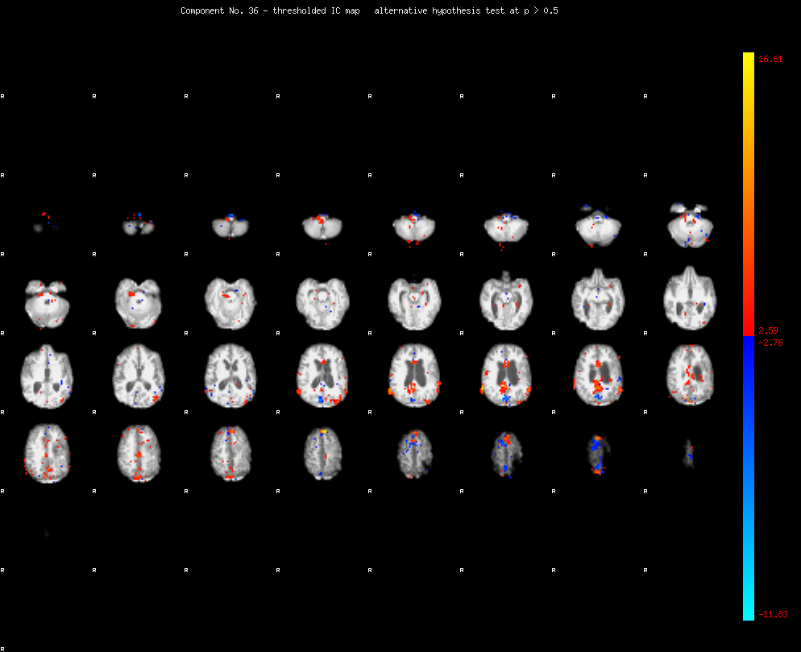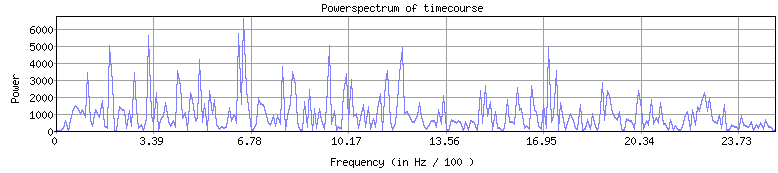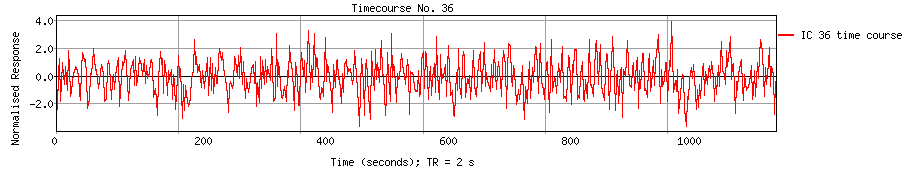 |
|  |  |
| 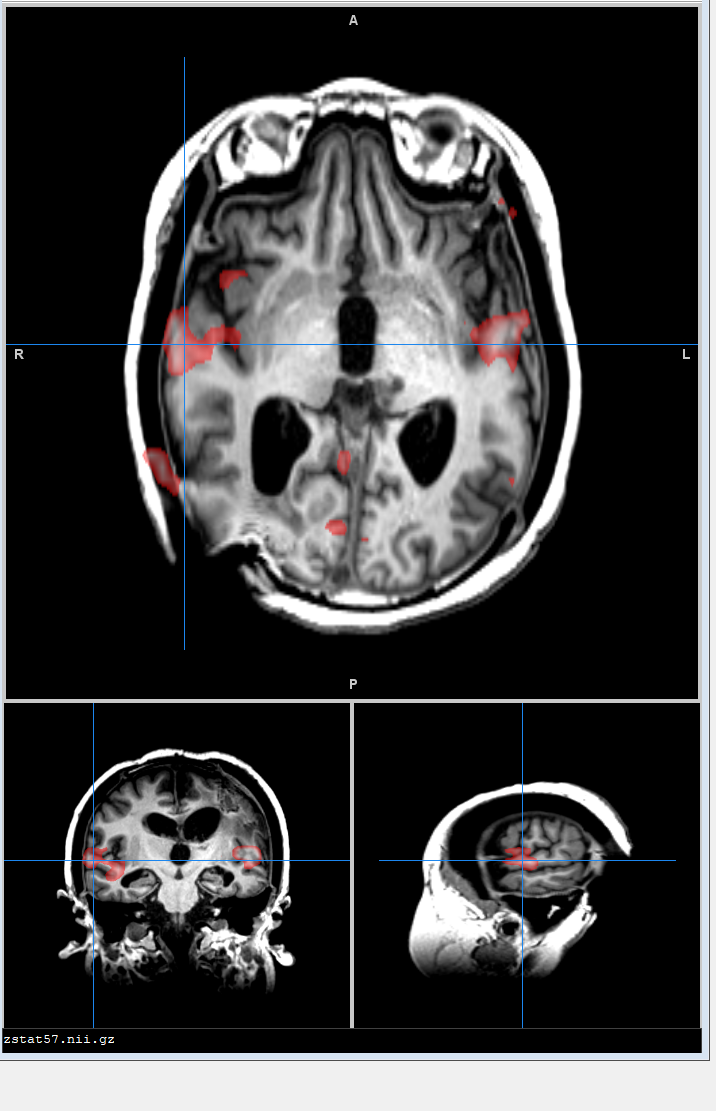 zstat57 | 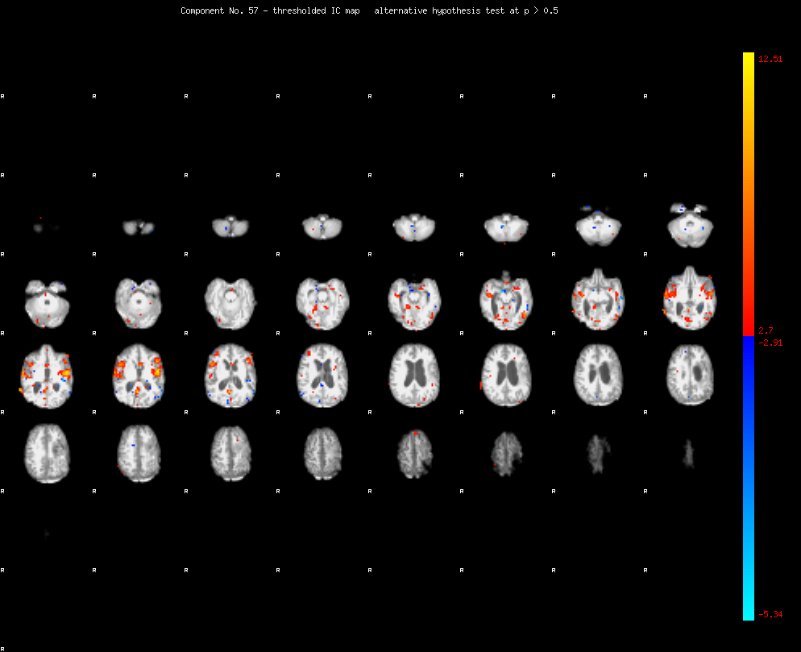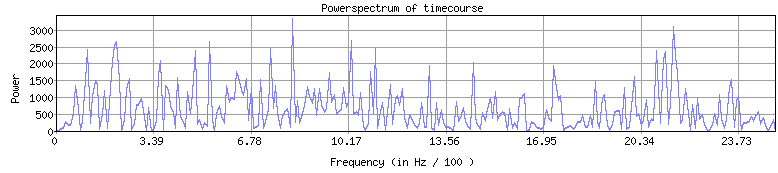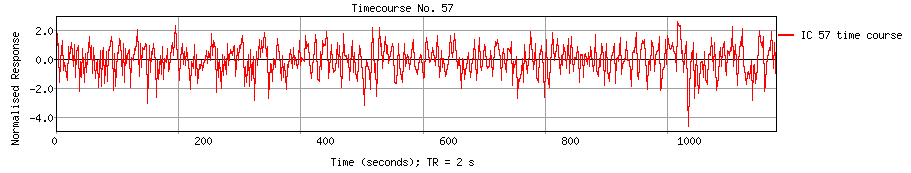 |
|  |  |
|  |  |
| 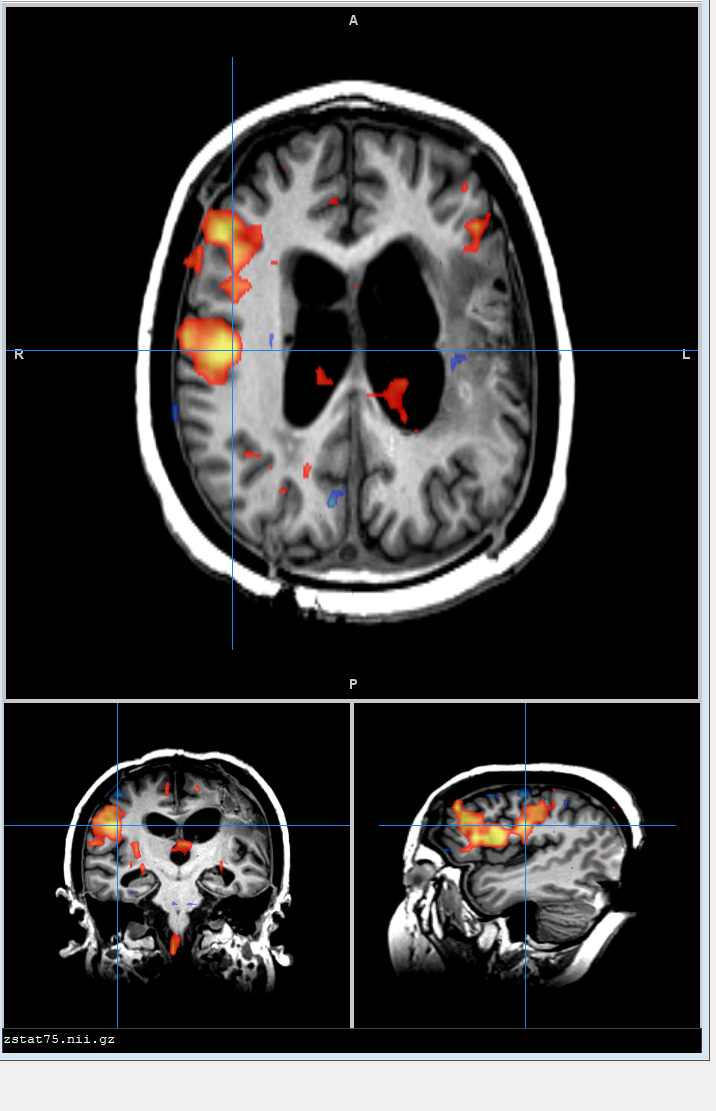 zstat75 | 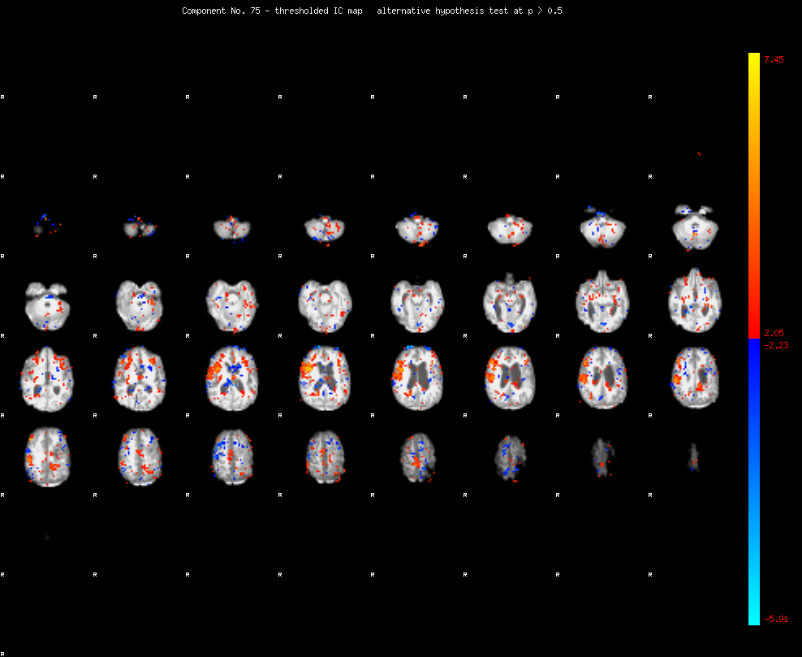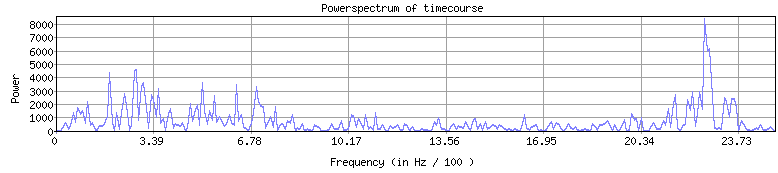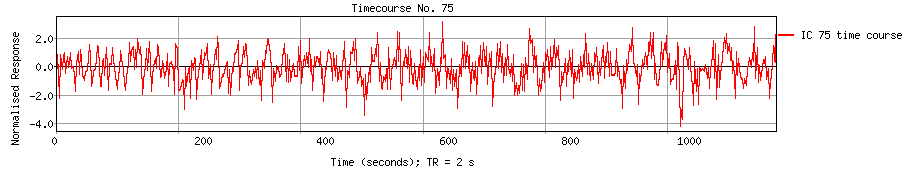 |
| 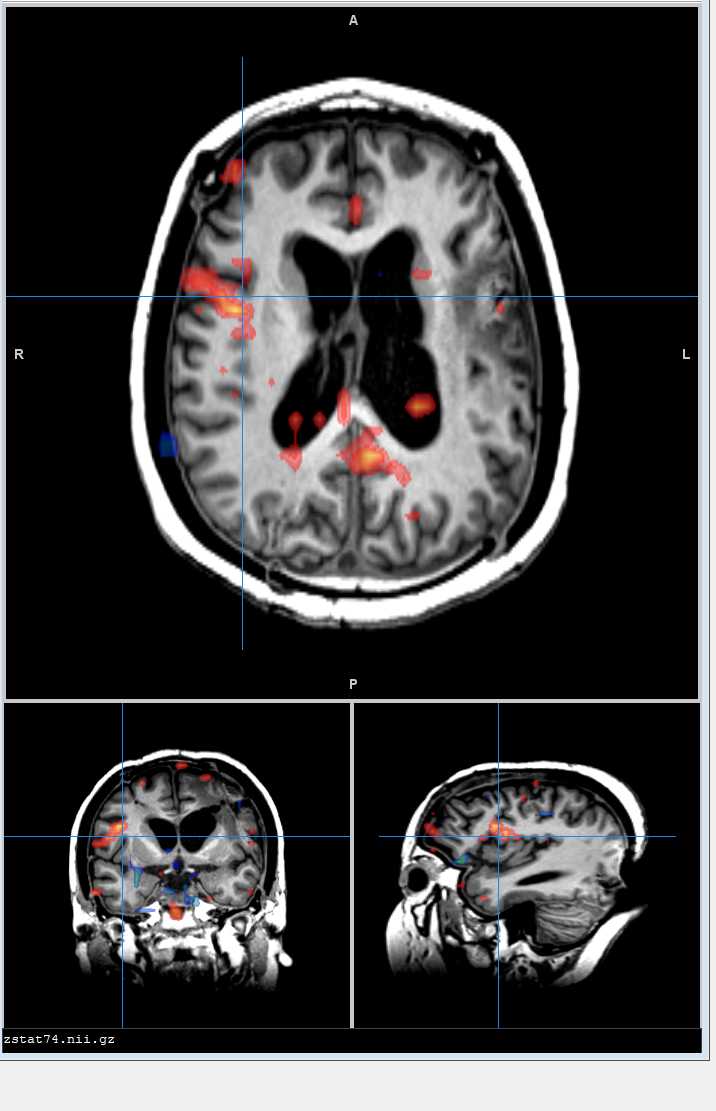 zstat74 | 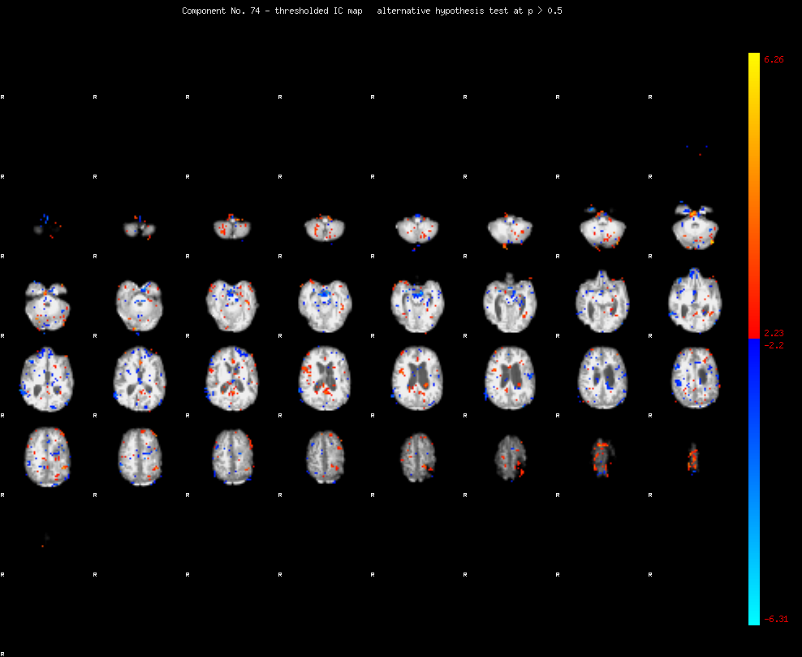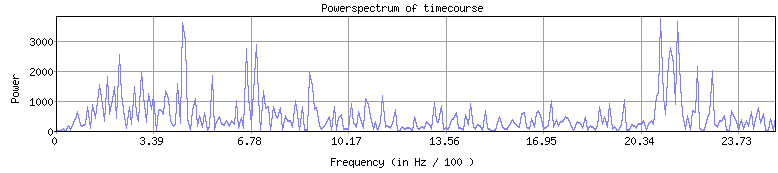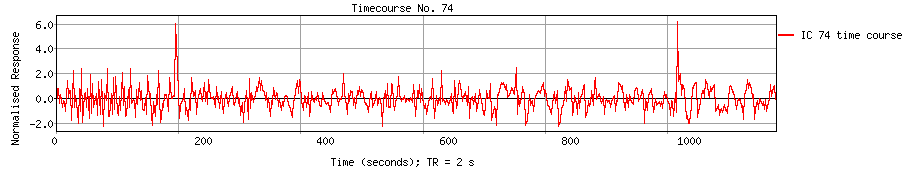 |

| **Atypical RSN** |
| --- |

| 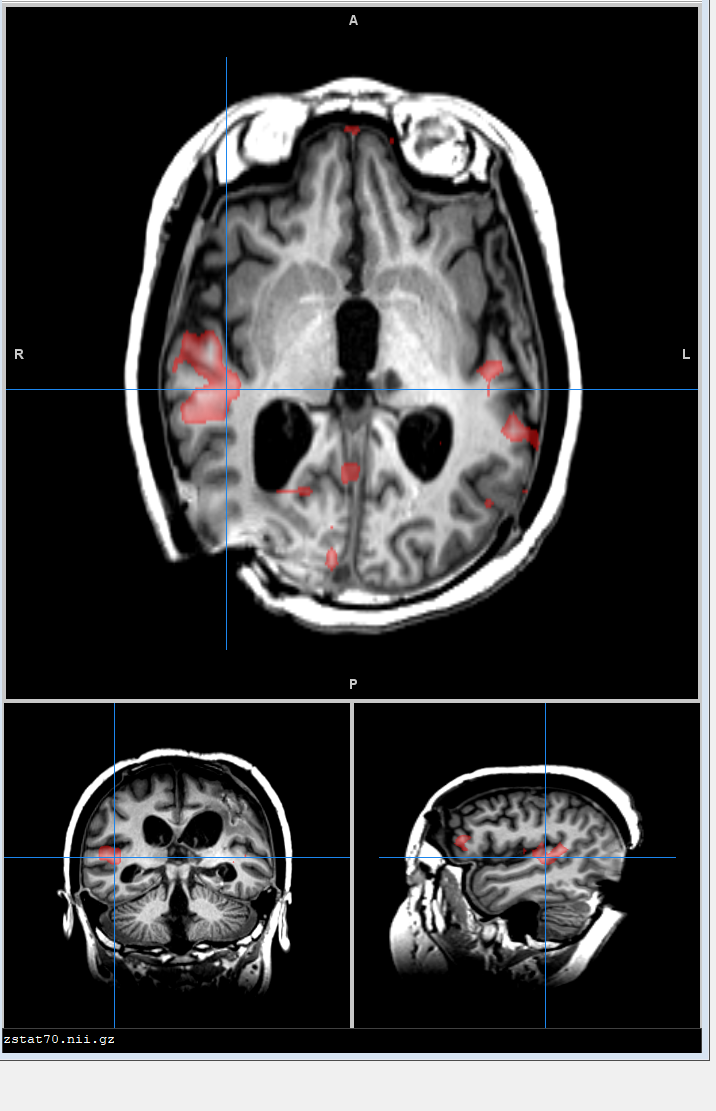 zstat70 | | 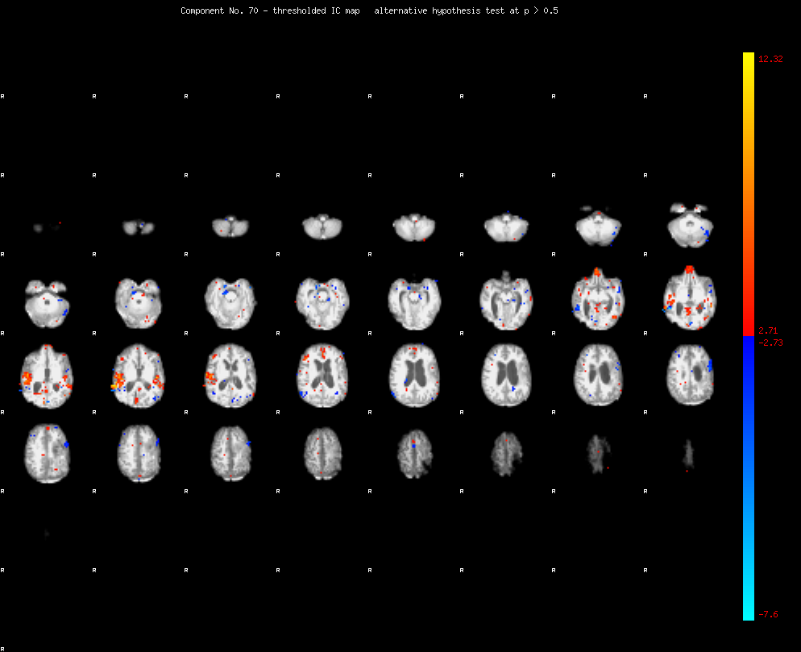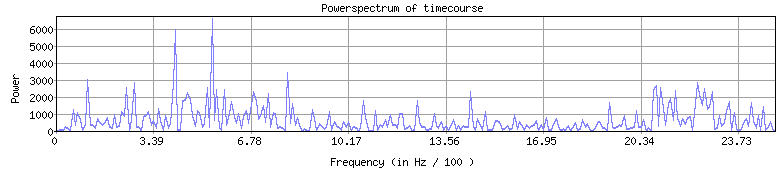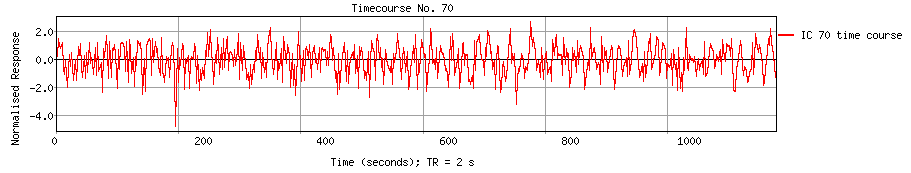 |
| --- | --- | --- |
| 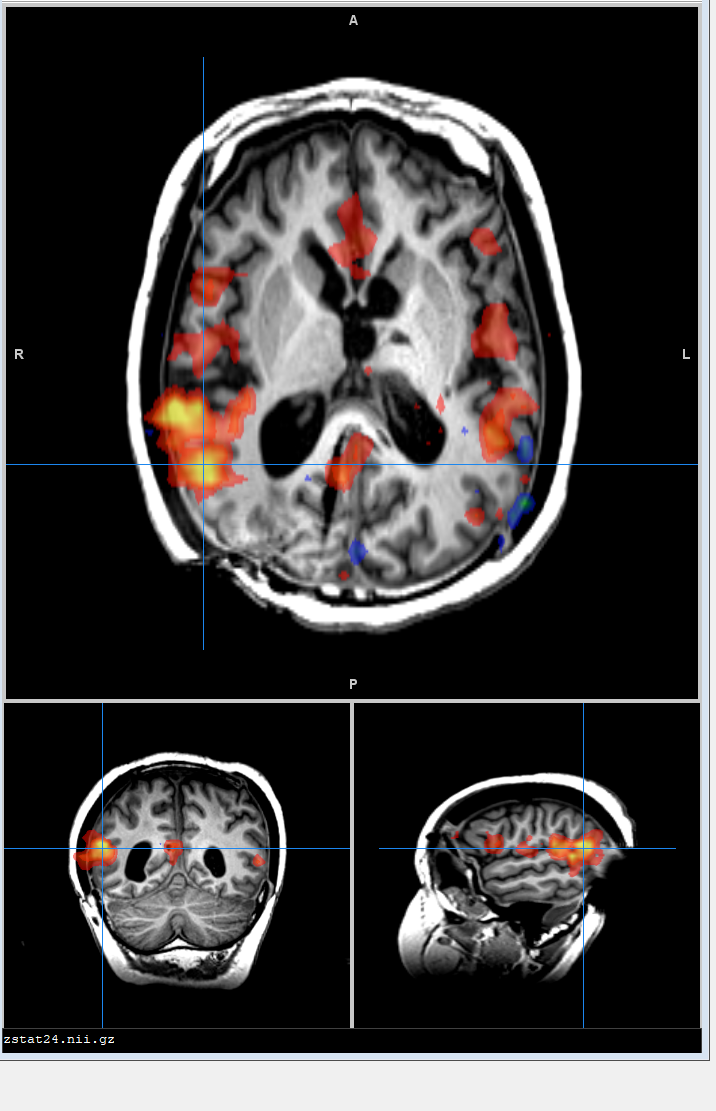 zstat24 | | 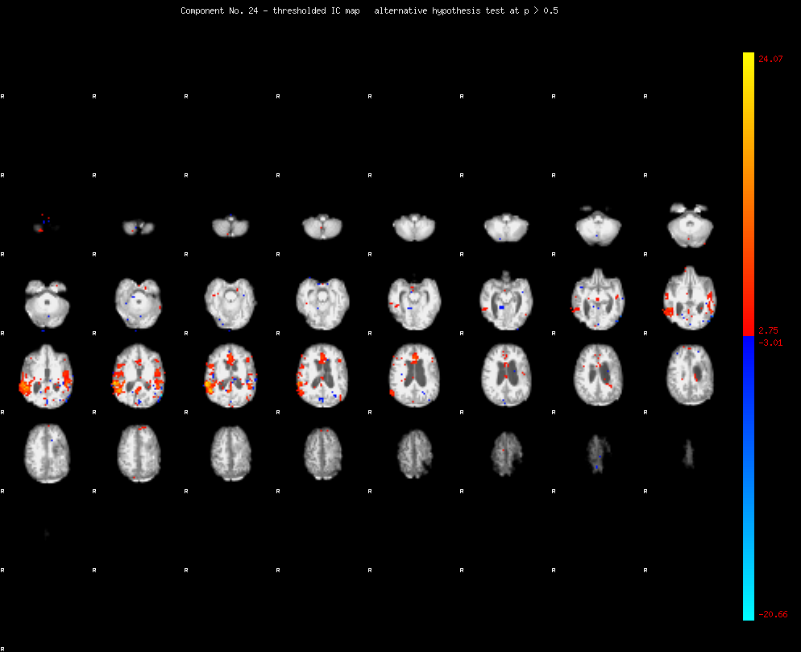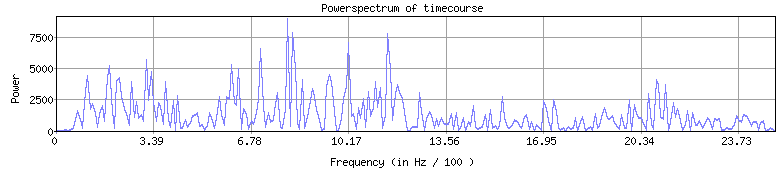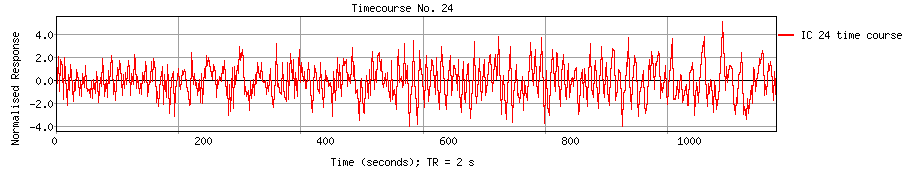 |
|  |  | |
|  |  | |

| **Motor** |
| --- |

| 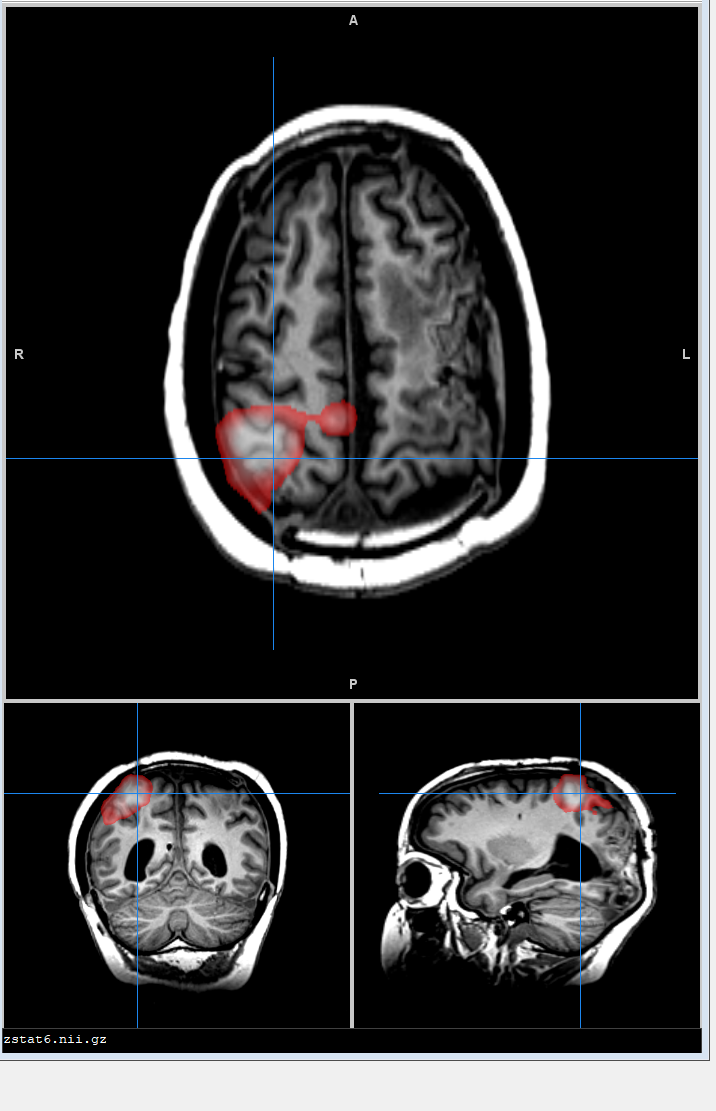 zstat6 | 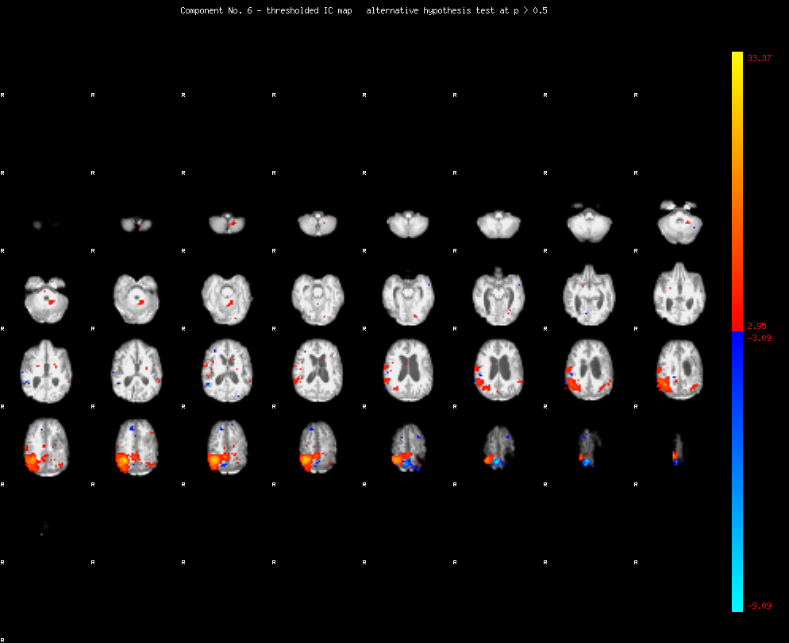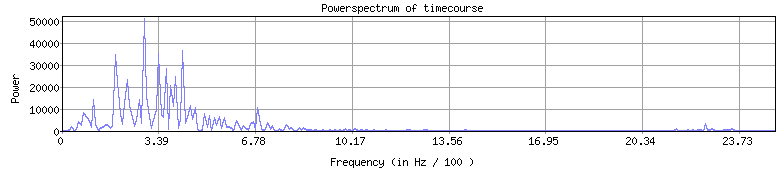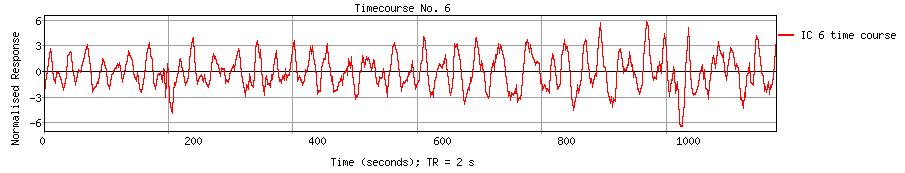 |
| --- | --- |
| 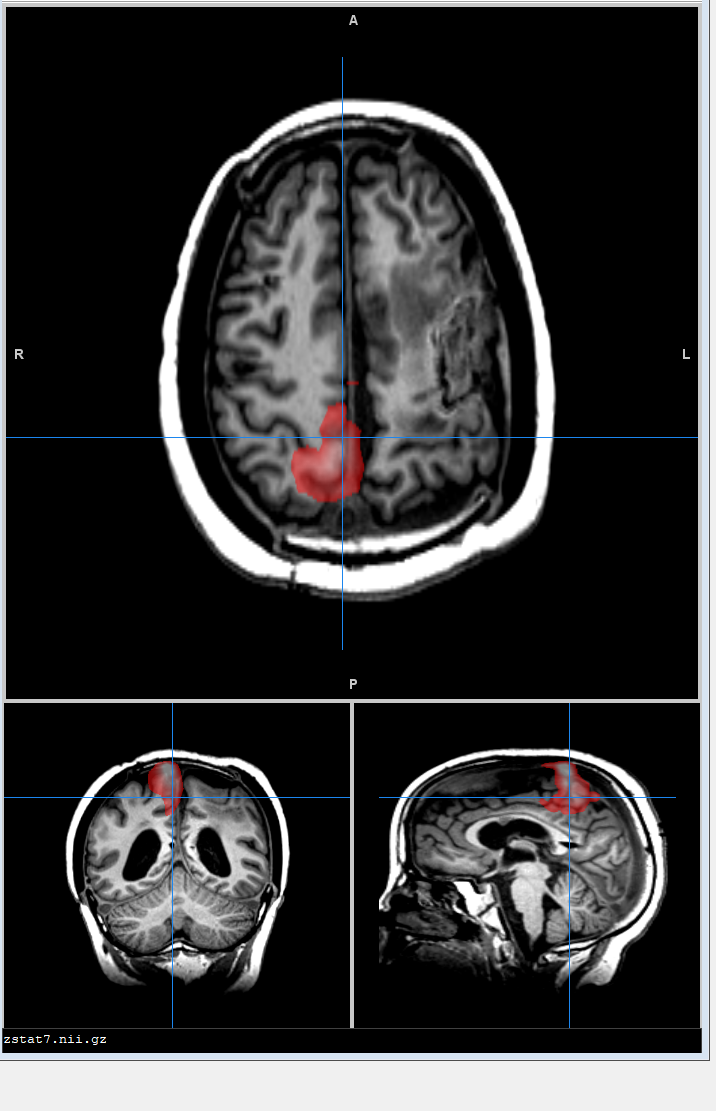 zstat7 | 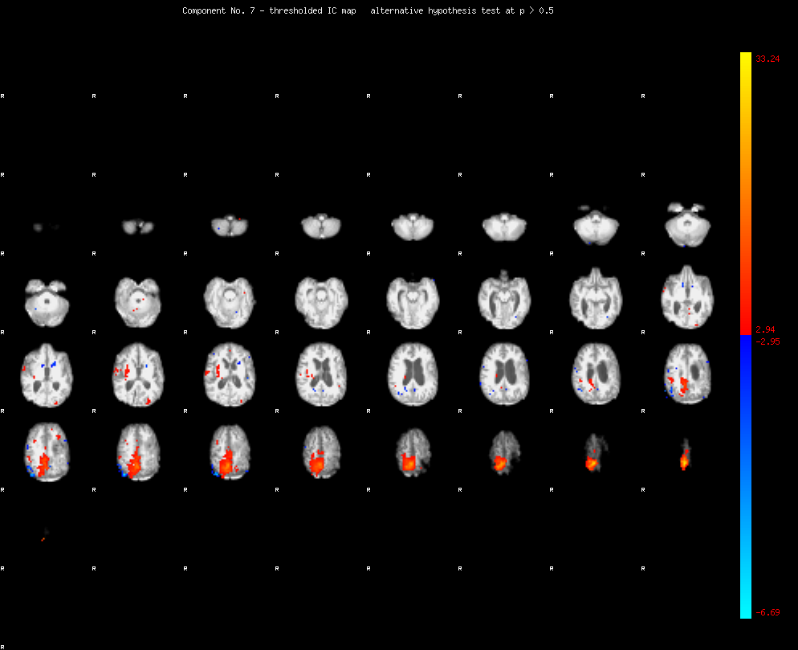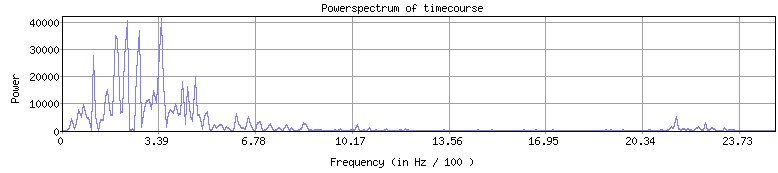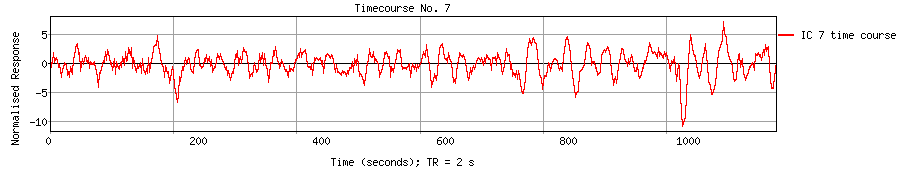 |
| 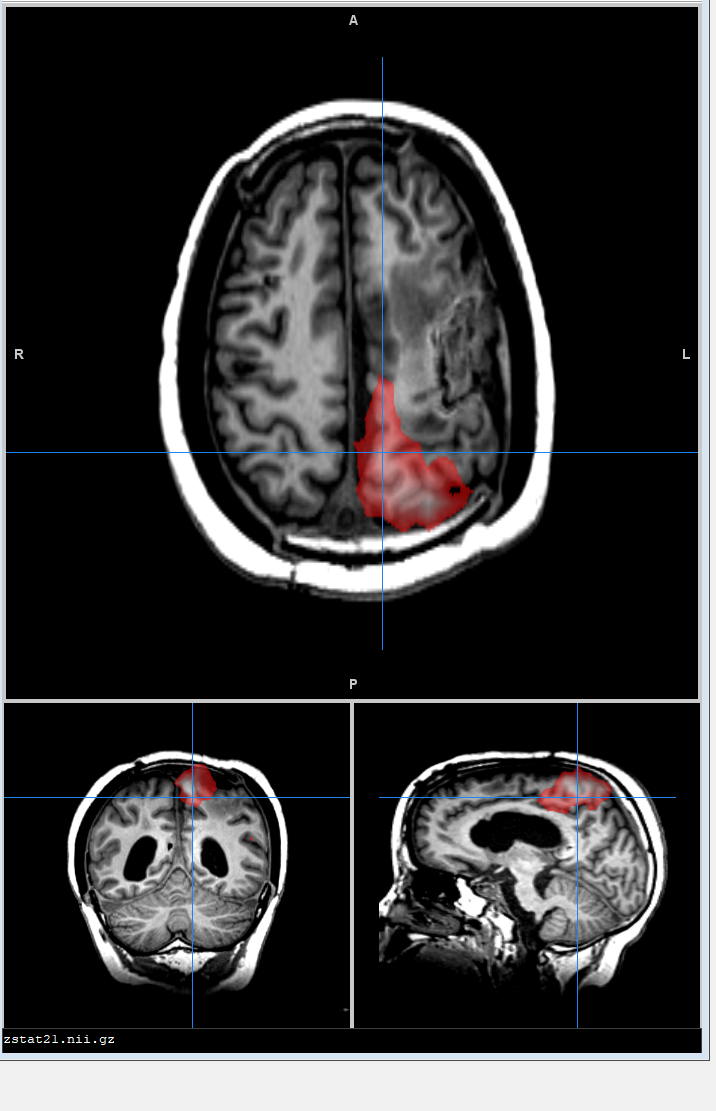 zstat21 | 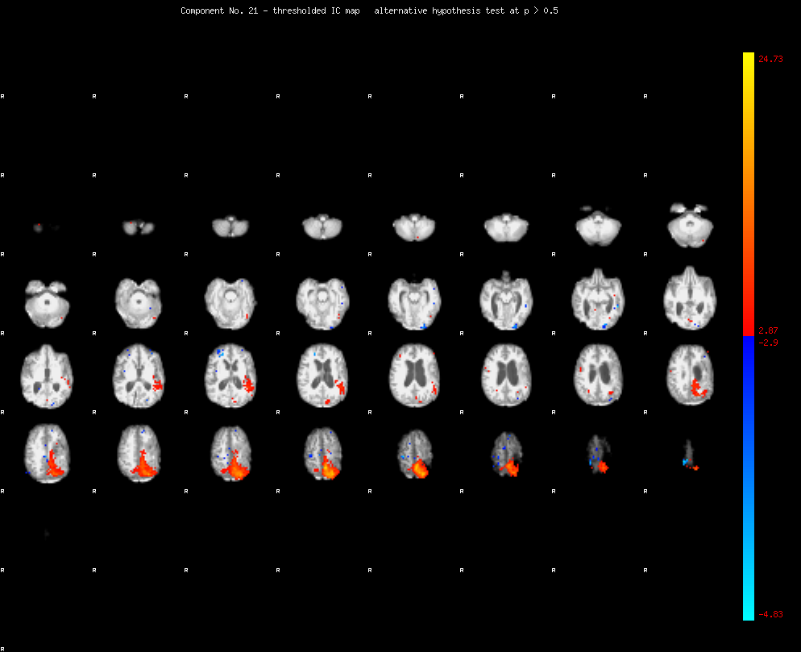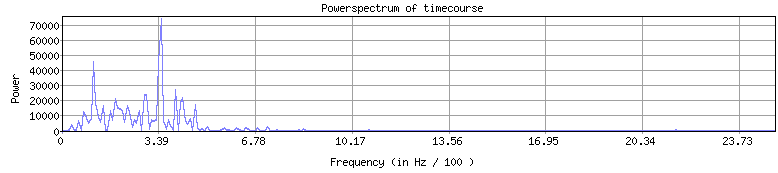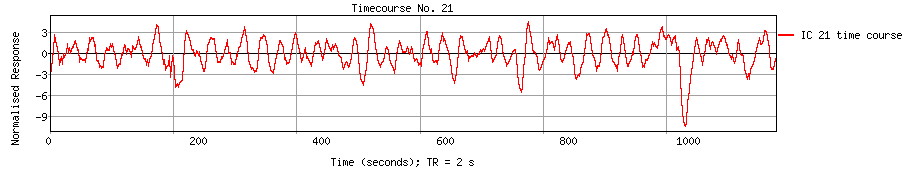 |
| 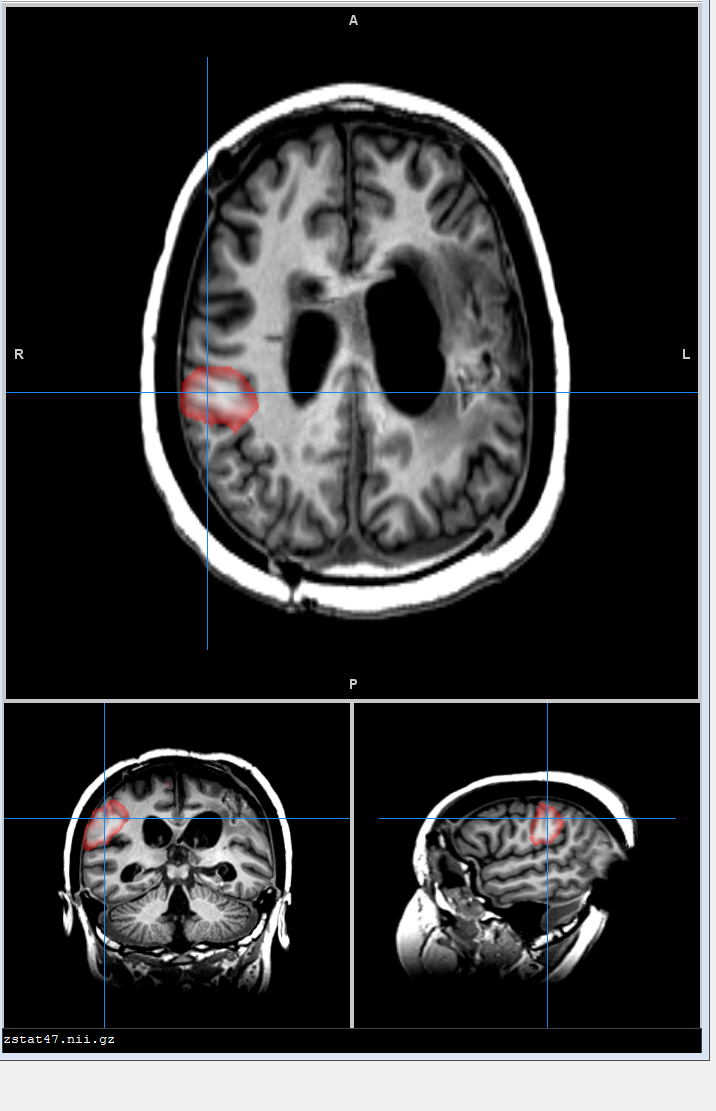 zstat47 | 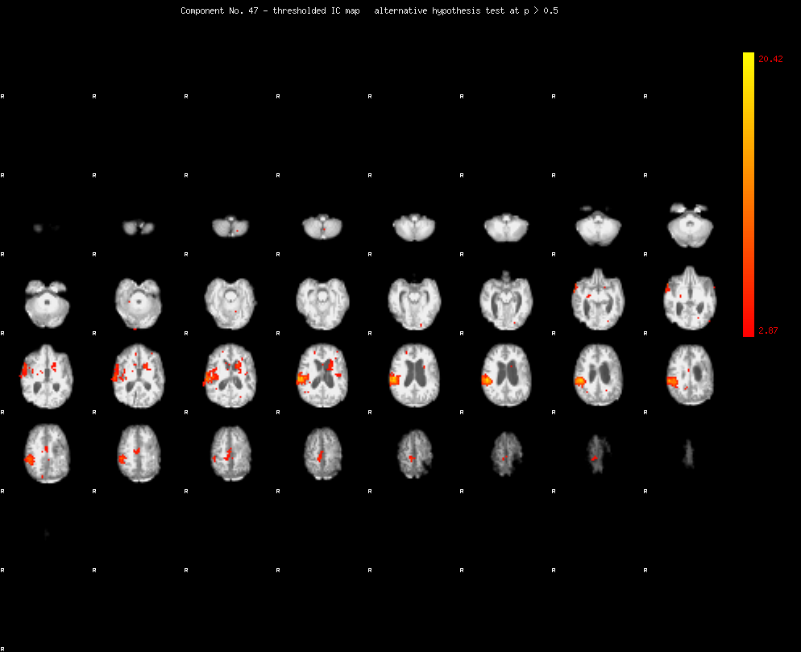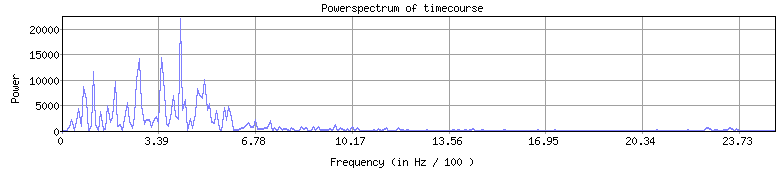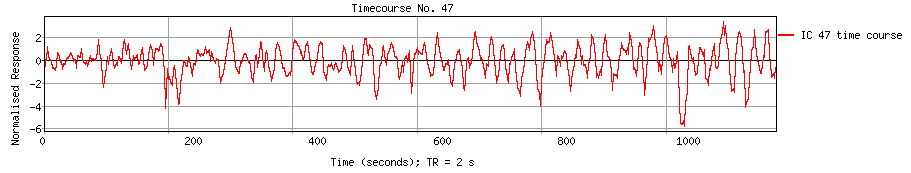 |
| 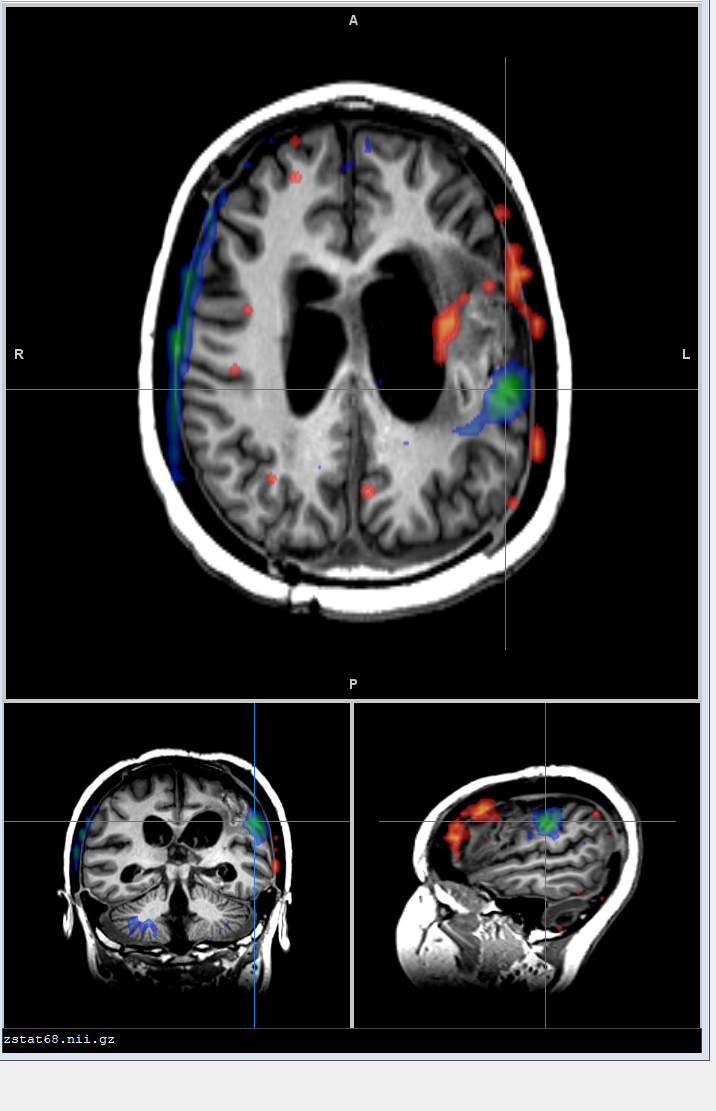 zstat68 | 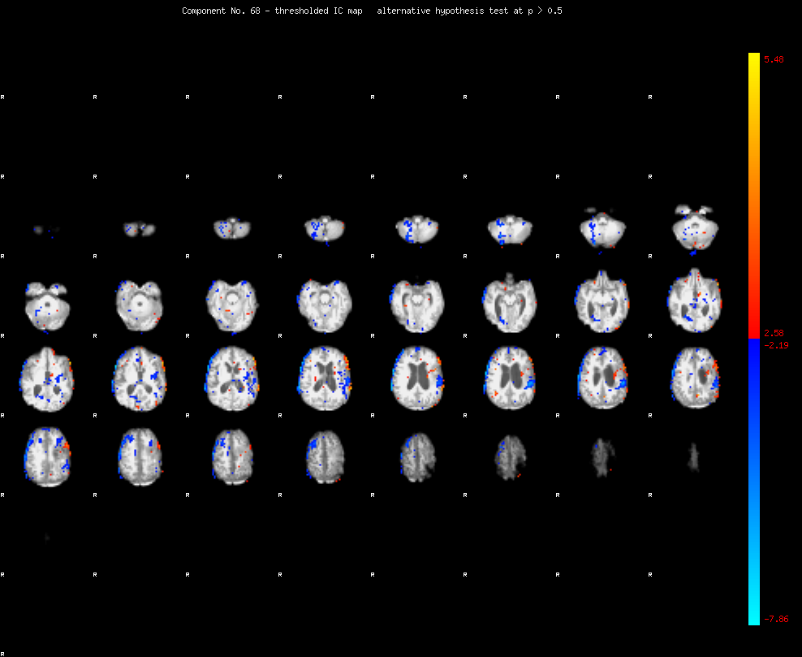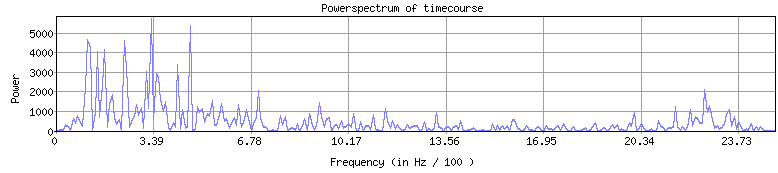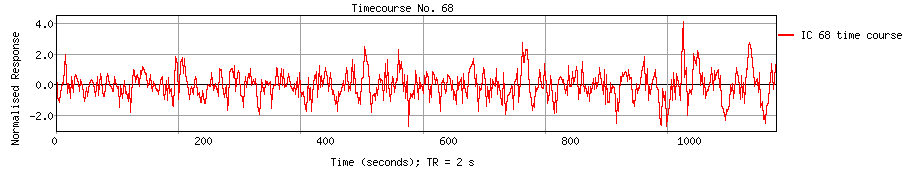 |
| 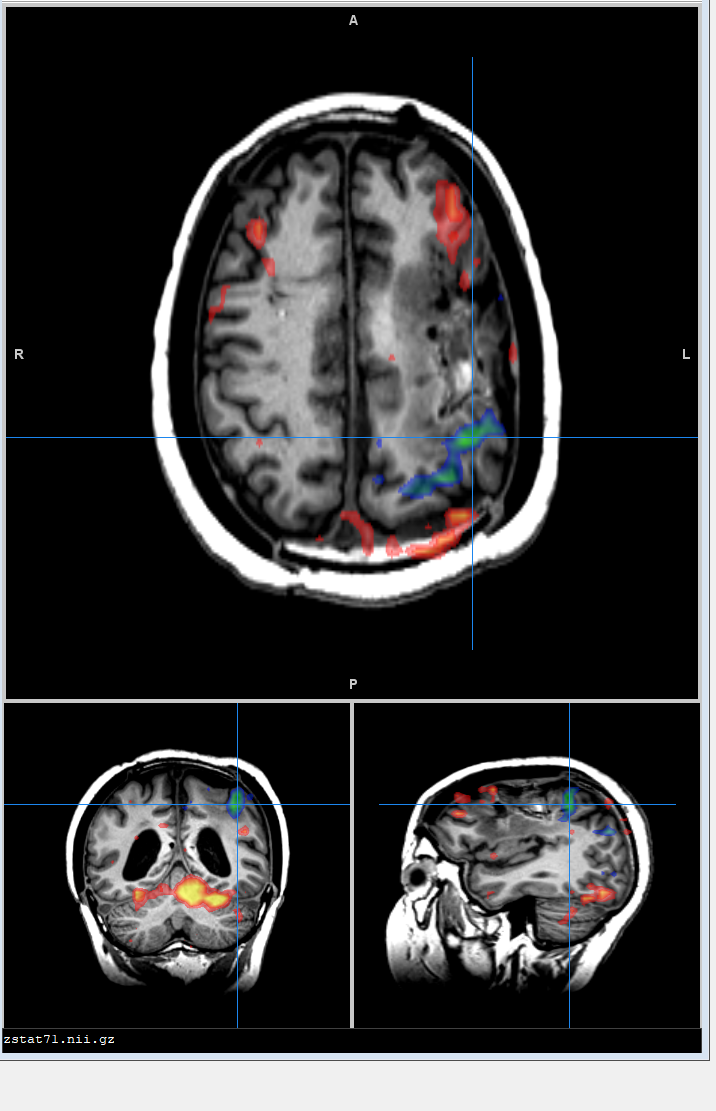 zstat71 | 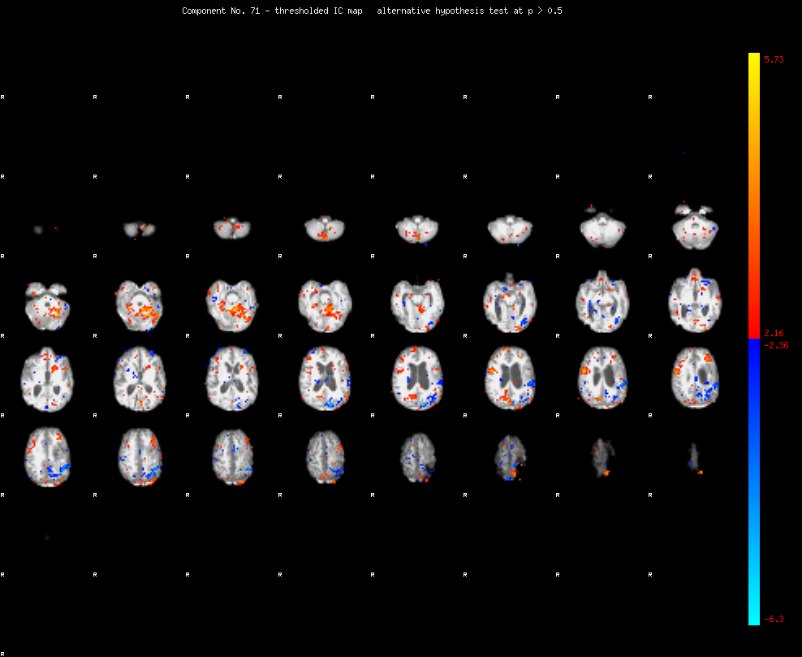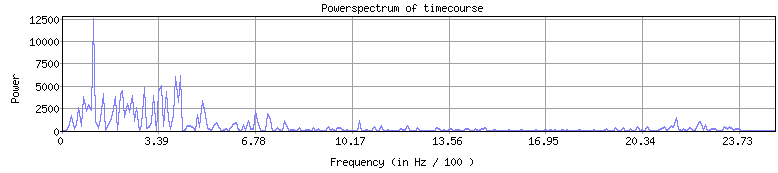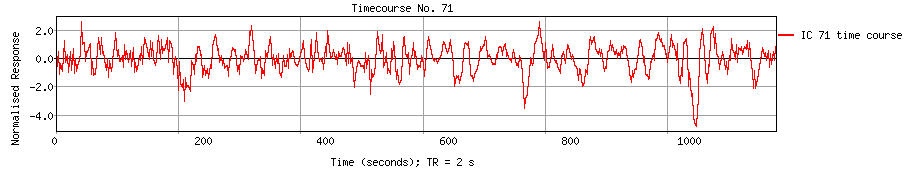 |
|  |  |

| **Language** |
| --- |

| Left language summary image | Right language summary image |
| --- | --- |
| 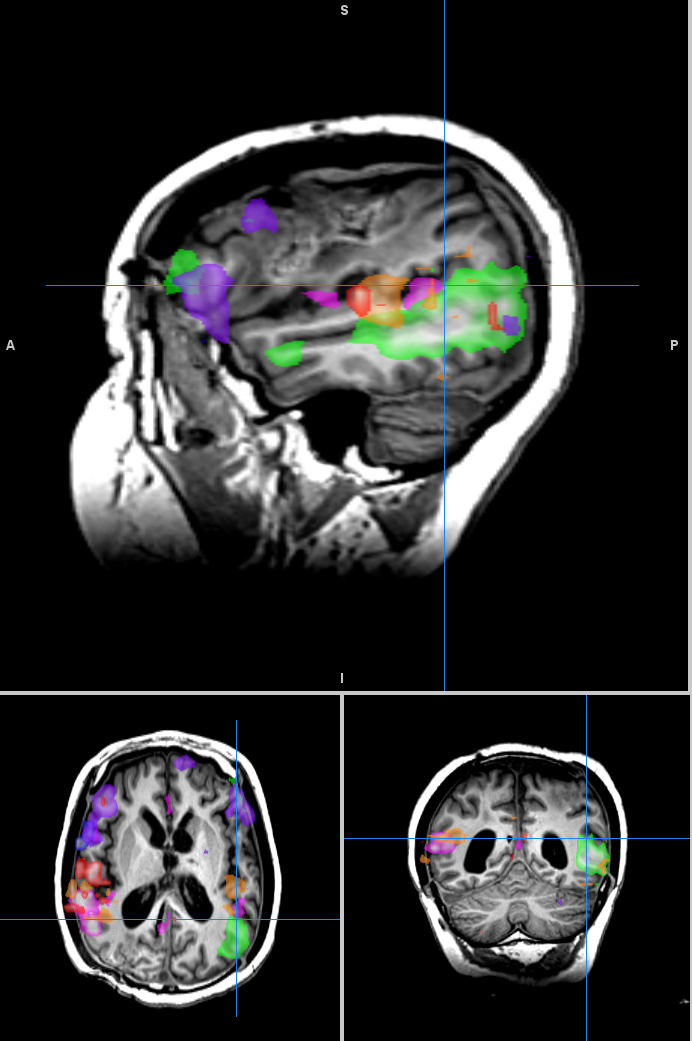 | 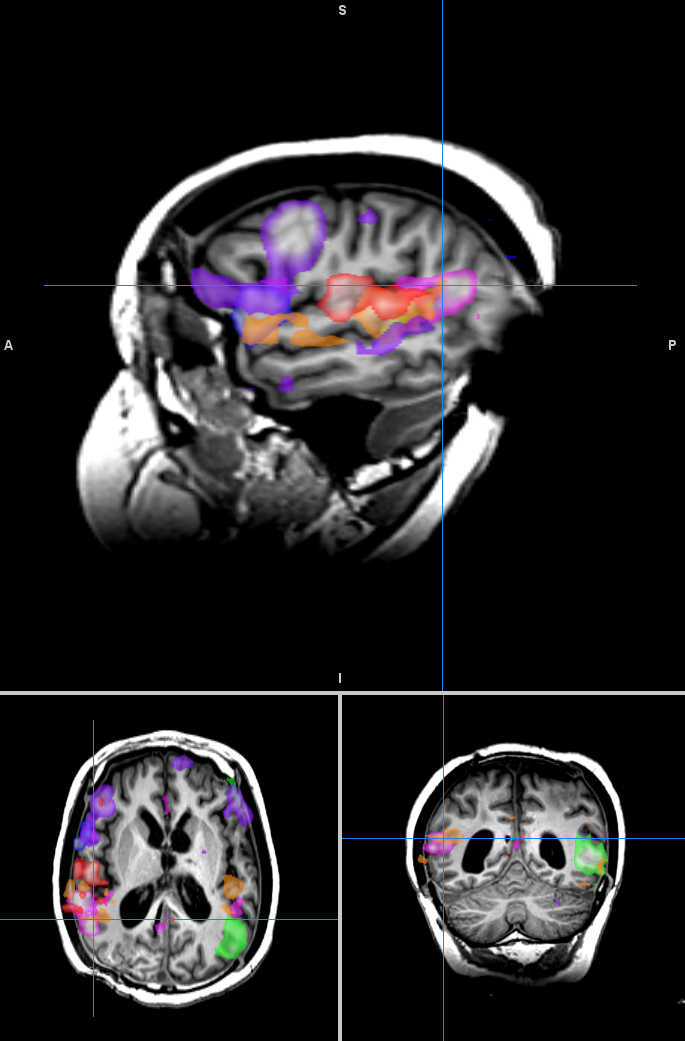 |
| 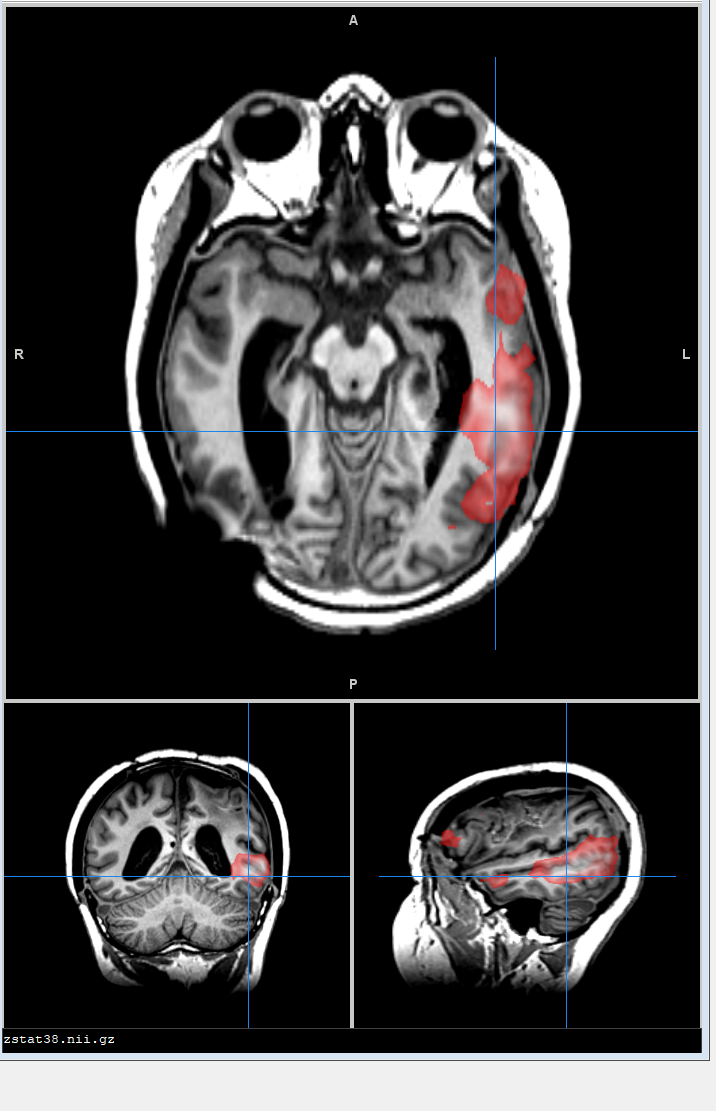 zstat38 | 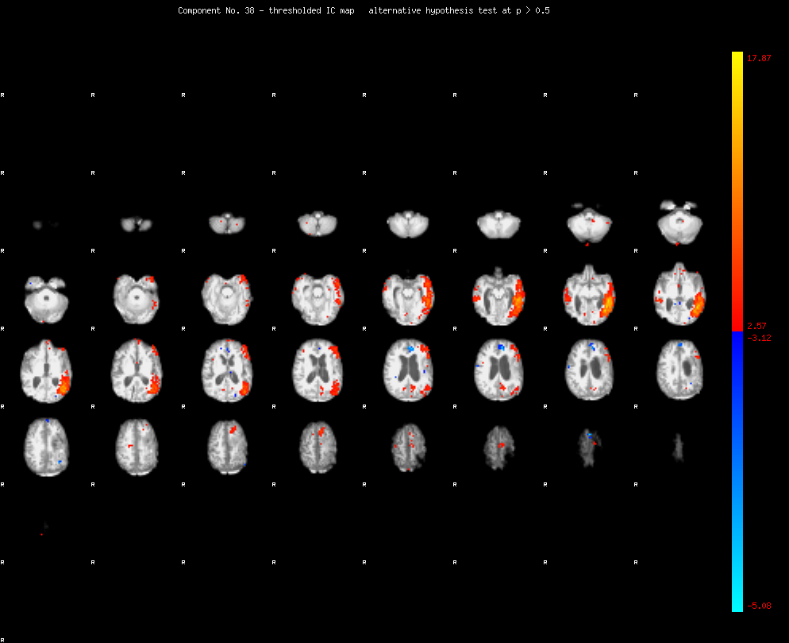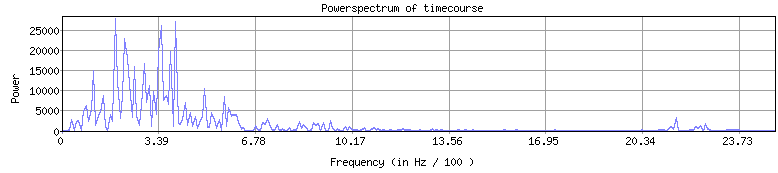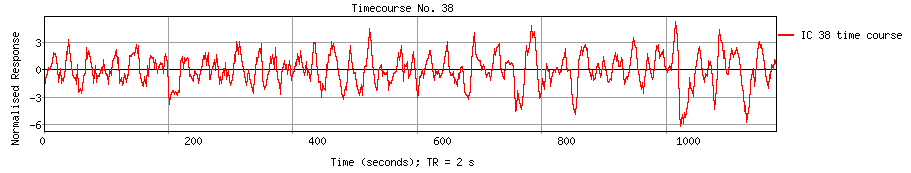 |
| 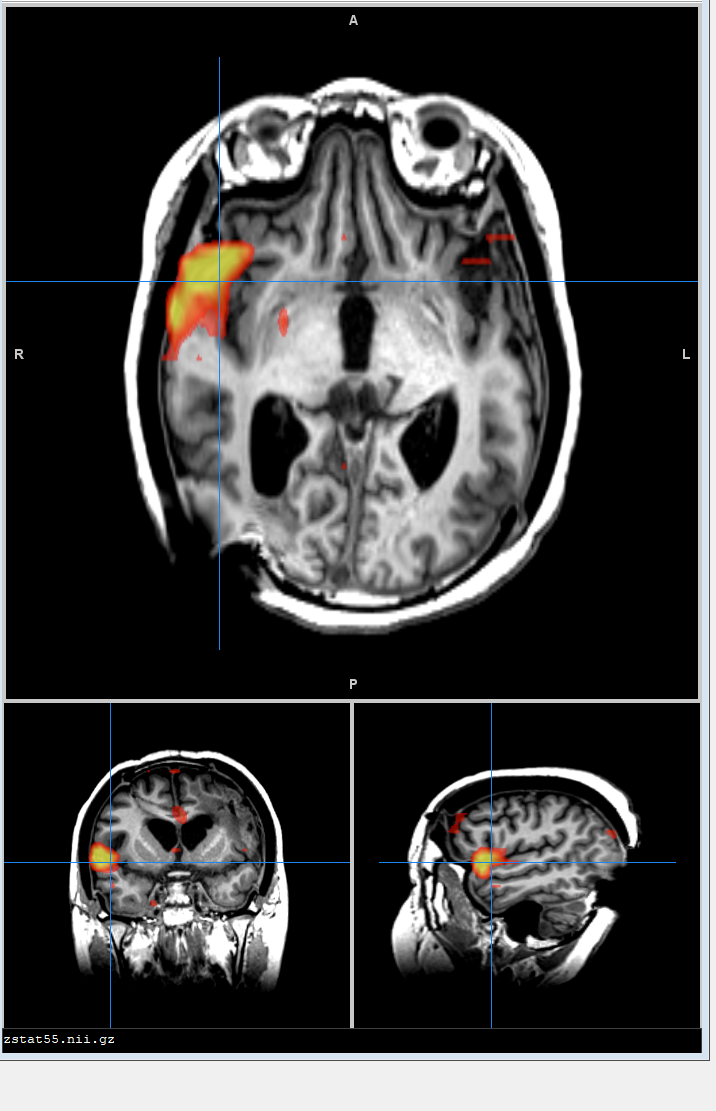 zstat55 | 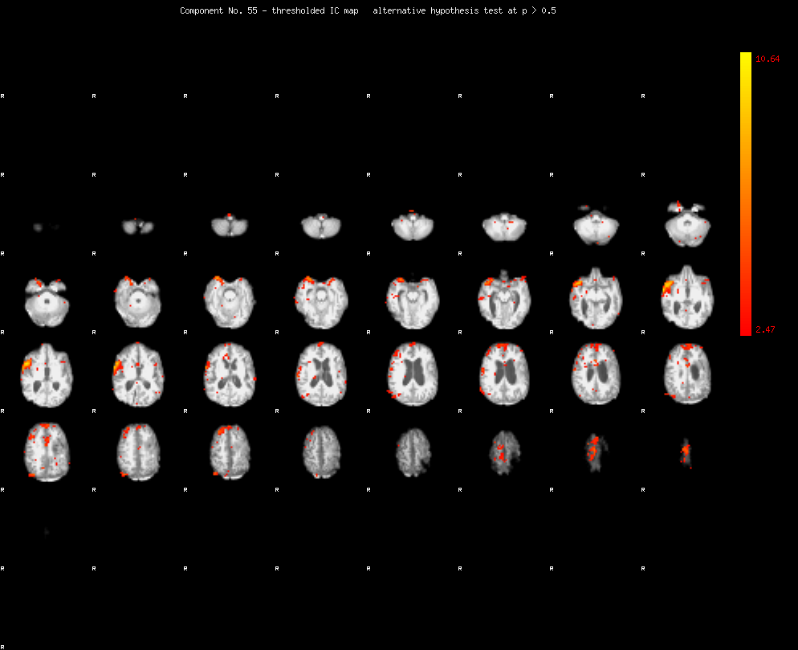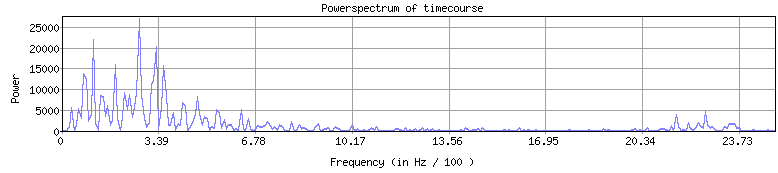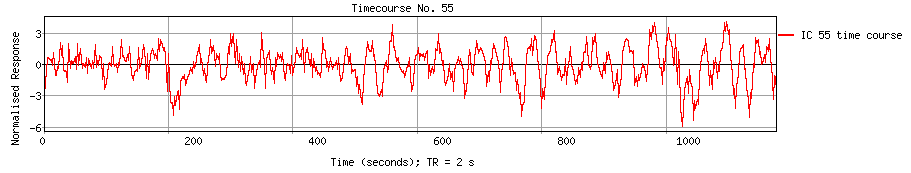 |
| 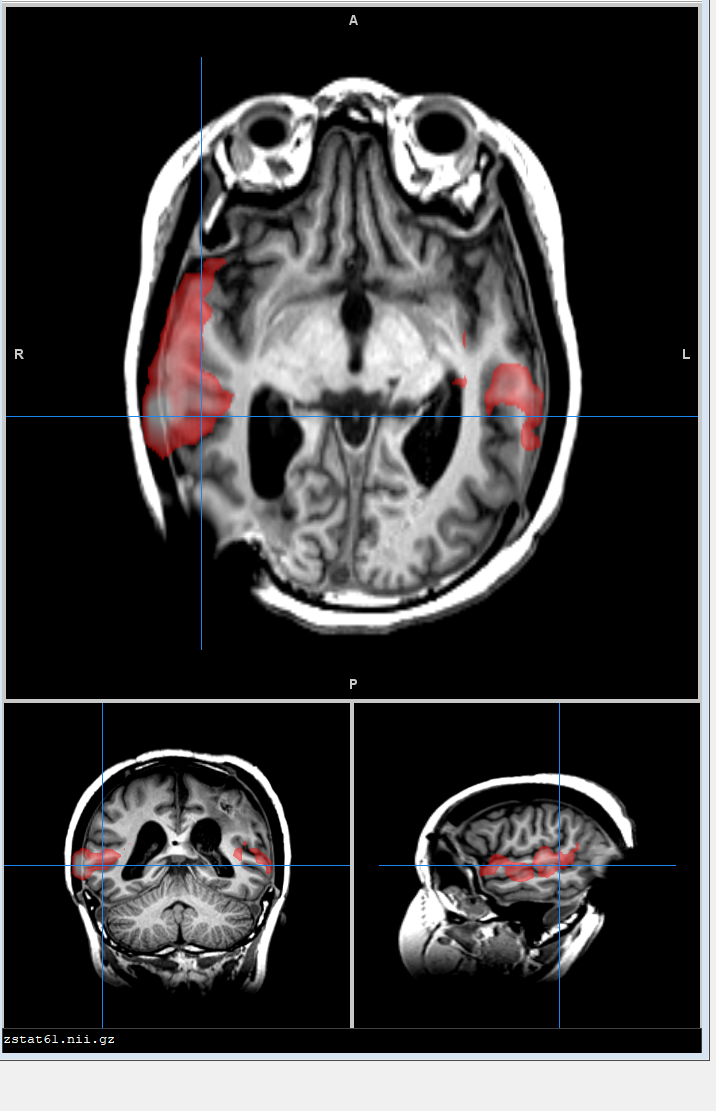 zstat61 | 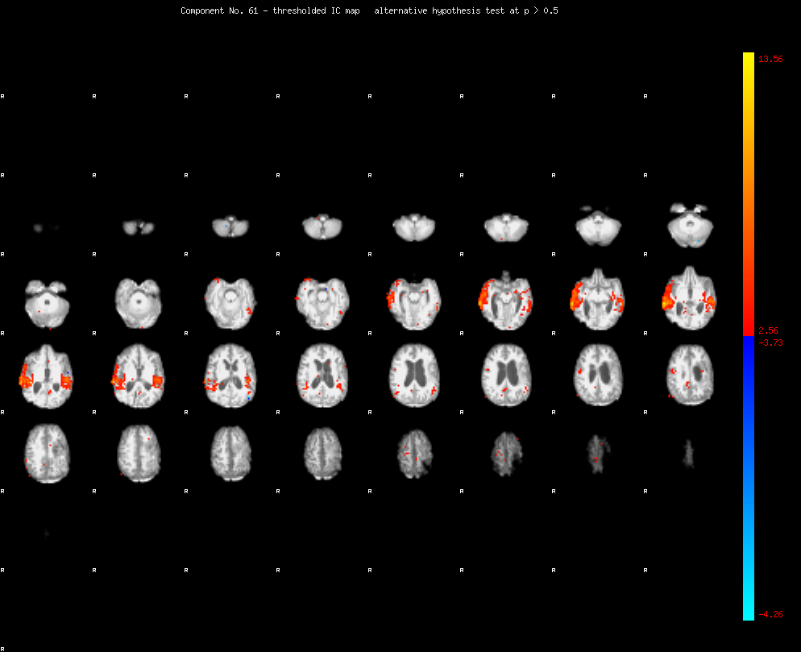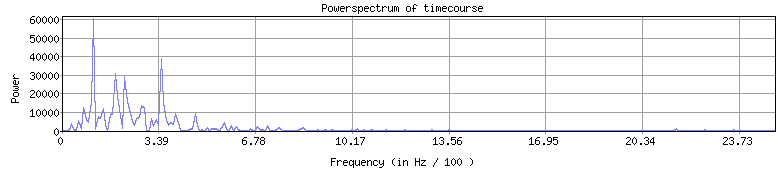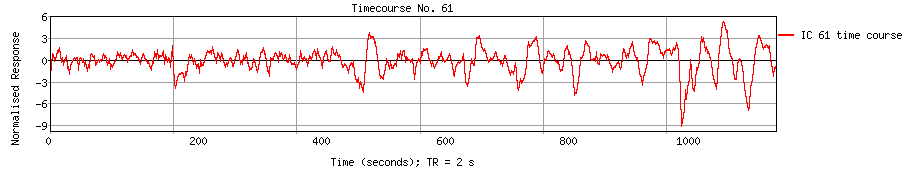 |
|  |  |
| 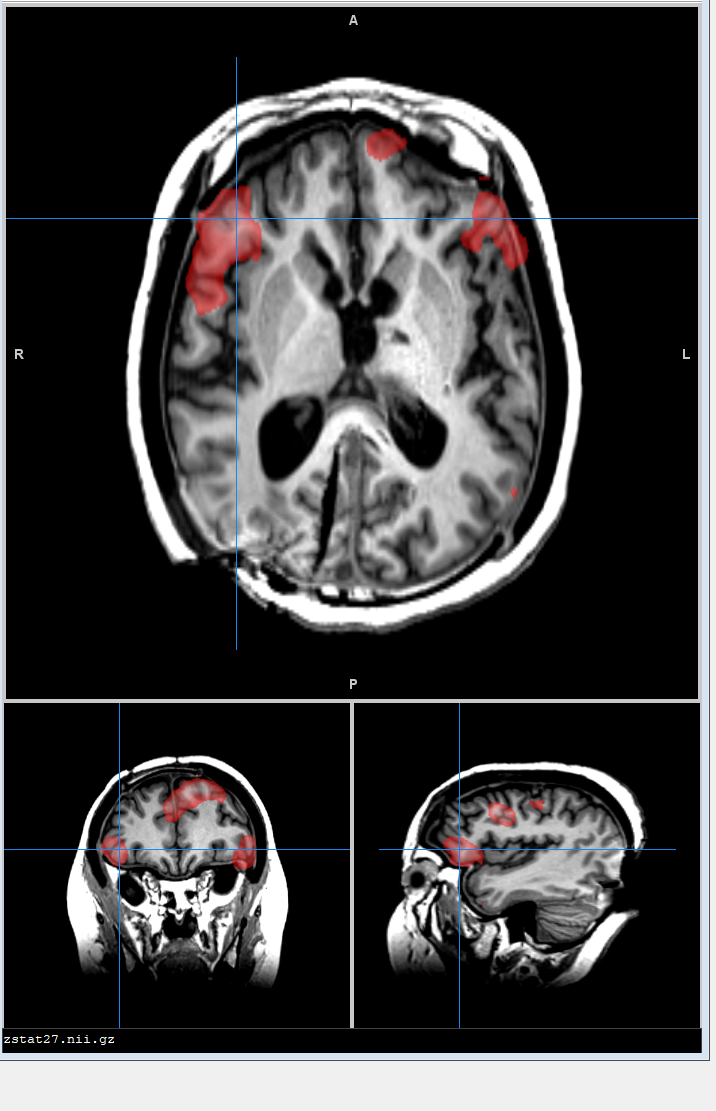 zstat27 | 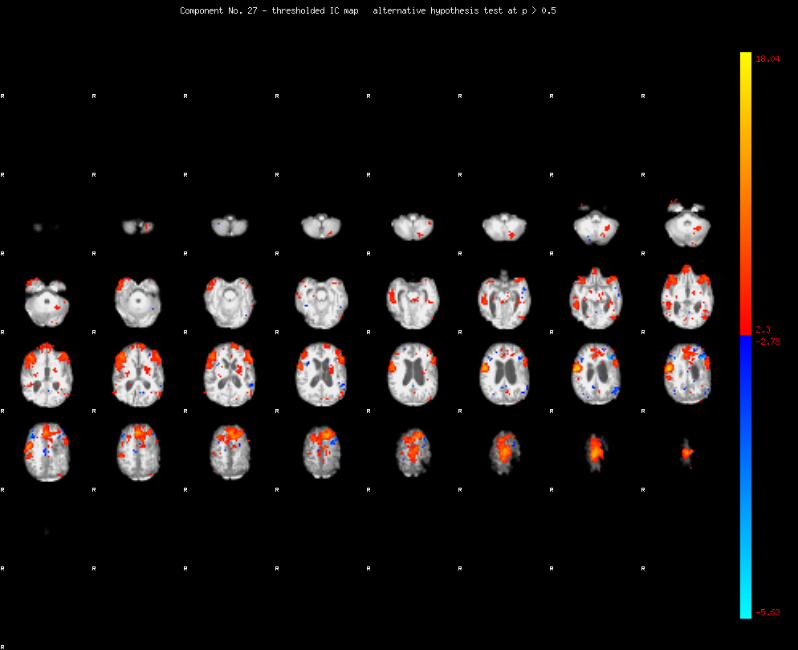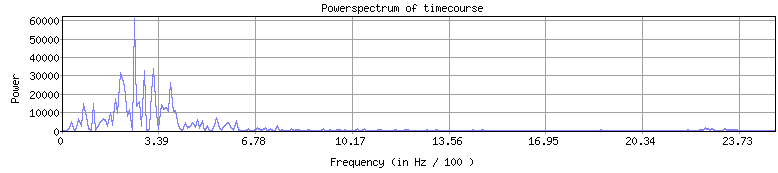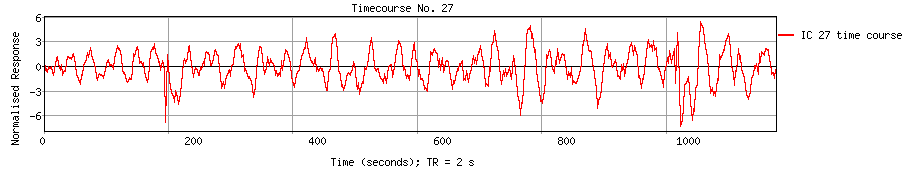 |

| **Parietal** |
| --- |

|  |  |
| --- | --- |
| 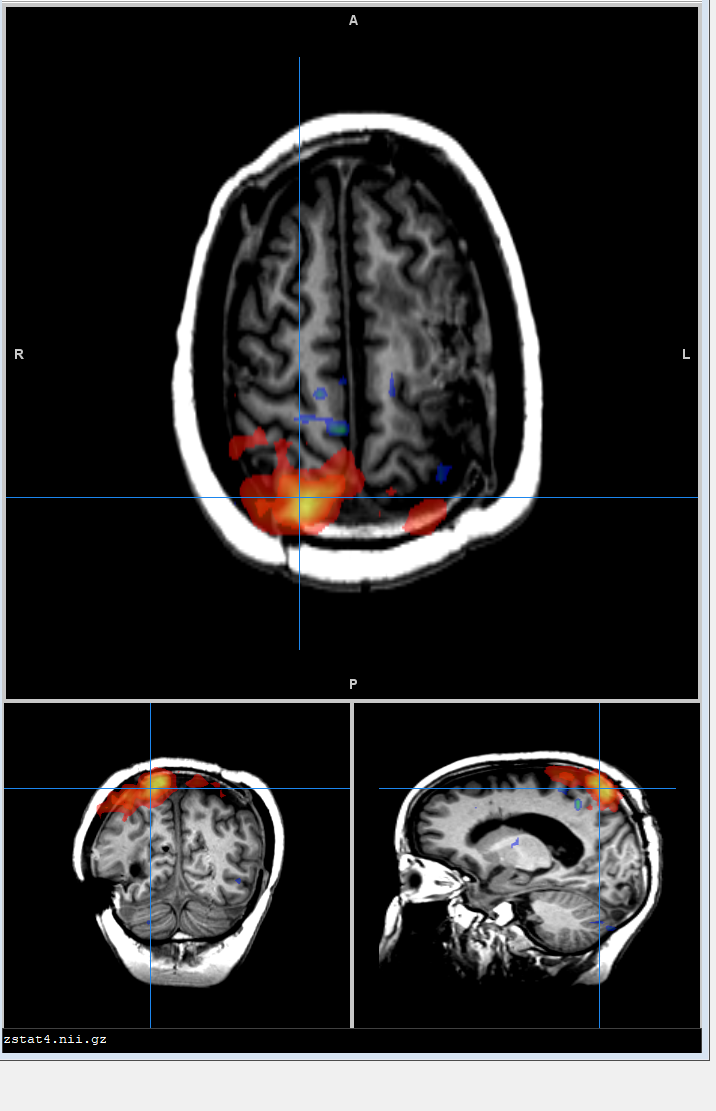 zstat4 | 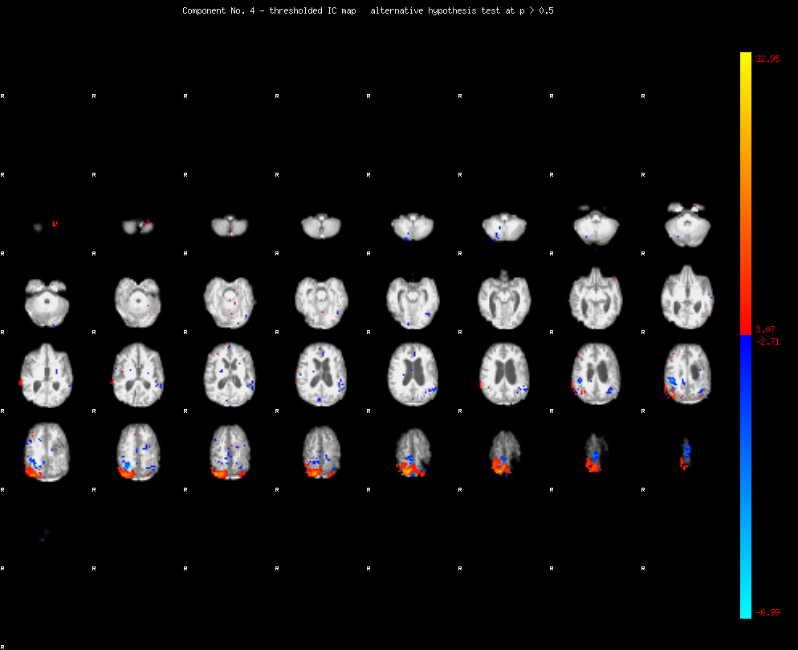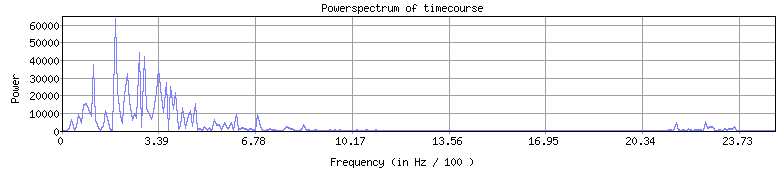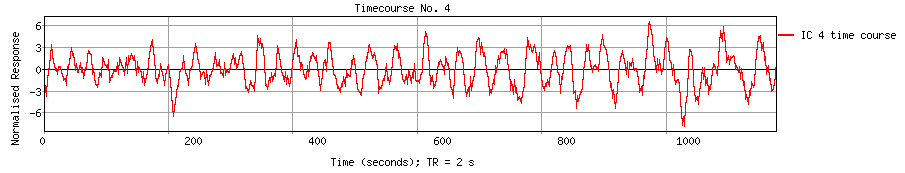 |
| 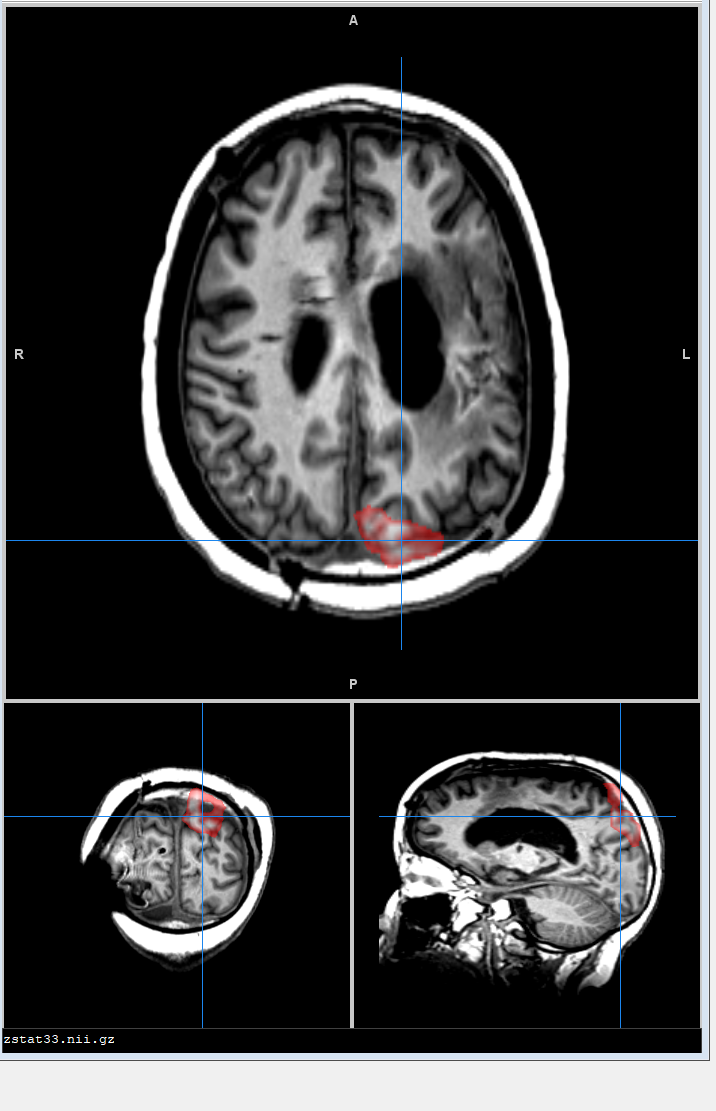 zstat33 |  |
| zstat37 |  |
| zstat63 |  |
|  |  |

| **Frontal** |
| --- |

| zstat9 |  |
| --- | --- |
|  |  |
| zstat35 |  |
| zstat40 |  |
| zstat62 |  |
| zstat72 |  |
|  |  |

| **Temporal** |
| --- |

| zstat66 |  |
| --- | --- |
| zstat69 |  |
|  |  |

| **Vision** |
| --- |

| zstat3 |  |
| --- | --- |
| zstat8 |  |
| zstat13 |  |
| zstat14 |  |
| zstat28 |  |
| zstat44 |  |
| zstat45 |  |
| zstat49 |  |
|  |  |

| **Deep Grey** |
| --- |

| zstat16 |  |
| --- | --- |
| zstat51 |  |
|  |  |

| **Modulating** |
| --- |

| zstat10 |  |
| --- | --- |
|  |  |

| **Association: Long-range Fronto-Parietal** |
| --- |

|  |  |
| --- | --- |
|  |  |

**Abbreviation Guide**

ACG Anterior Cingulate Gyrus

APF Anterior PreFrontal

AT anterior Temporal

B Bilateral

BG Basal Ganglia

DMN Default Mode Network

IFG Inferior Frontal Gyrus

IFS Inferior Frontal Sulcus

IPL Inferior Parietal Lobule

ITG Inferior Temporal Gyrus

L Left

LR-FTP Long Range Fronto-to-Parietal association network, anterior prefrontal IFG, MFG, posterior lateral parietal S2

LS2 – lateral Secondary Sensory area

MFG Middle Frontal Gyrus

MT mesial Temporal

MTG Middle Temporal Gyrus

mS2 – medial Secondary Sensory area

OPC- Operculum

OTG Occipito-temporal gyrus

PCG Posterior Cingulate Gyrus

PFC Prefrontal Cortex

PMC Premotor Cortex

PHG Parahippocampal Gyrus

POS Parietal Occipital Sulcus

R Right

Rs-fMRI Resting state functional MRI

RS Resting State

RSN Resting state network – an expected brain network

S1 Primary somatosensory cortex

S2 Secondary somatosensory cortex or secondary sensory association area

SFG Superior Frontal Gyrus

SFS Superior Frontal Sulcus

SMA Supplementary Motor Association area

SMG Supramarginal Gyrus

SOZ Seizure onset zone

SPL Superior Parietal Lobule

STG Superior Temporal Gyrus

STS Superior Temporal Sulcus

TOJ – Temporal Occipital Junction network

V1 Primary visual cortex associated network

V2 Secondary visual cortex associated network

vmPFC ventral medial prefrontal cortex
