# Supplementary material for: Pre- and post-therapy functional MRI connectivity in severe acute brain injury with suppression of consciousness: a comparative analysis to epilepsy features
Source: Front Neuroimaging. 2024 Oct 1;3:1445952. doi: 10.3389/fnimg.2024.1445952 (PMC11473429; doi:10.3389/fnimg.2024.1445952)
Supplement: Supplementary file 4 [file Table_4.DOCX]

**
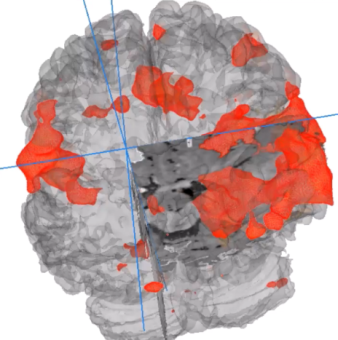
**

Resting State Connectivity Whole Brain Map

PP3_1

Anatomical MRI see separate radiological report

Technique and analysis Methods 3T MRI, whole brain BOLD ICA

Data Quality Analysis 20 min; Head motion < 1 mm, no interfering artifacts detected

Abbreviation index list at end of rs-report

| **Interpretation** |
| --- |
| **Motor**  Normal |
| **Language**  Nearly equal, left dominate |
| **Parietal**  Normal |
| **Frontal**  Normal |
| **Temporal**  Normal  **Vision**  Normal  **Deep Grey**  Normal  **Modulating**  Normal spatial, faster erratic temporal coarse |
| **Association**  Normal |
| **Atypical Signal Sources**  **2 vm-PFC deactivation; 8 brainstem, deep grey & Frontal - broad cortical activation**  (9) vm-PFC deactivation, brainstem & deep grey activation ** most abnormal  (44) vm-PFC deactivation, deep grey PFC activation ** most abnormal  **Broader and less localizing – though deep grey B heavily involved**  (73) deep grey – Opc activation  (44) deep grey – Opc-PFC activation  (74) B AT-MT activation  (86) PFC deactivation  (41) P deactivation  (37) P deactivation  **Impression** |

Deactivation of the vm-PFC is highly atypical, and in signal source 9 is found with alternating activation-deactivation pattern emanating from the brainstem through the deep grey. The other patterns emulate the effect of such disruptive signal source with broad atypical cortical and subcortical connectivity. Notably ALL of the expected normal networks were detected, including the modulating network (though with faster than typical temporal features), portending a potentially excellent cognitive recovery is possible.

Given the location and pattern two unconvential therapies for such an atypical injury pattern are suggested:

1. Attempt empiric treatment for subcortical epilepsy – load 30 mg/kg fos, if no significant improvement in 6-12 hours, load 30 mg/kg phenobarbital. If no improvement in 1-2 days, this makes this diagnosis much less likely.
2. Consider brain stimulation to bypass or modulate the atypical brainstem to vm-PFC connectivity. Considerations include VNS or DBS. Of course, discussion with team and neurosurgery indicated, as this would be a less convential therapy.

Atypical

| 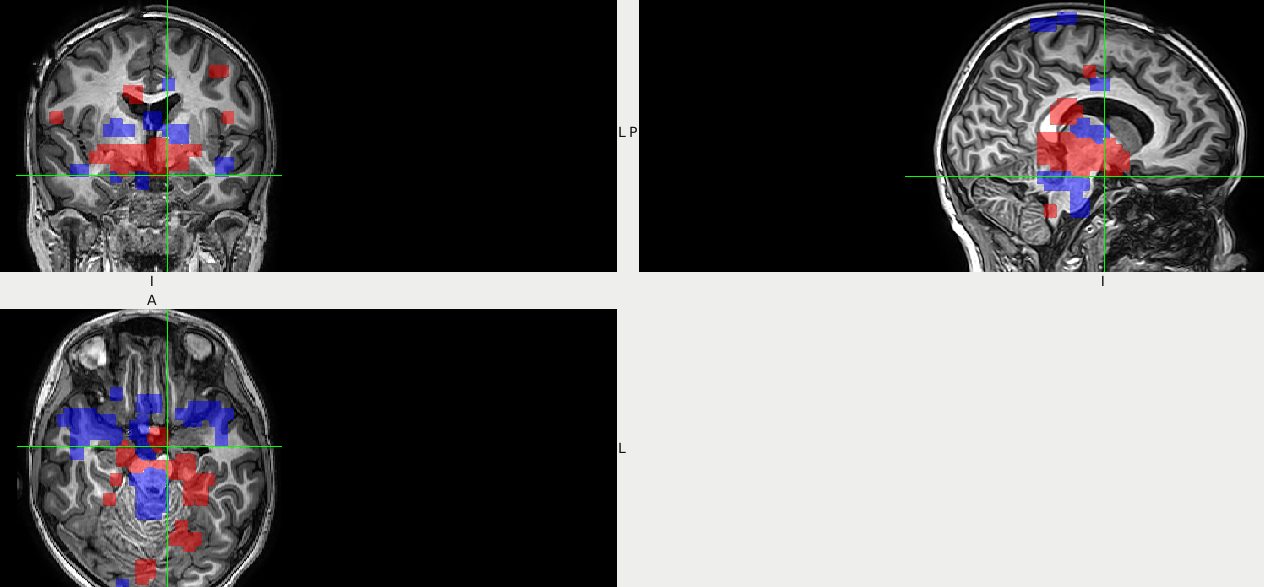  zstat 9 | 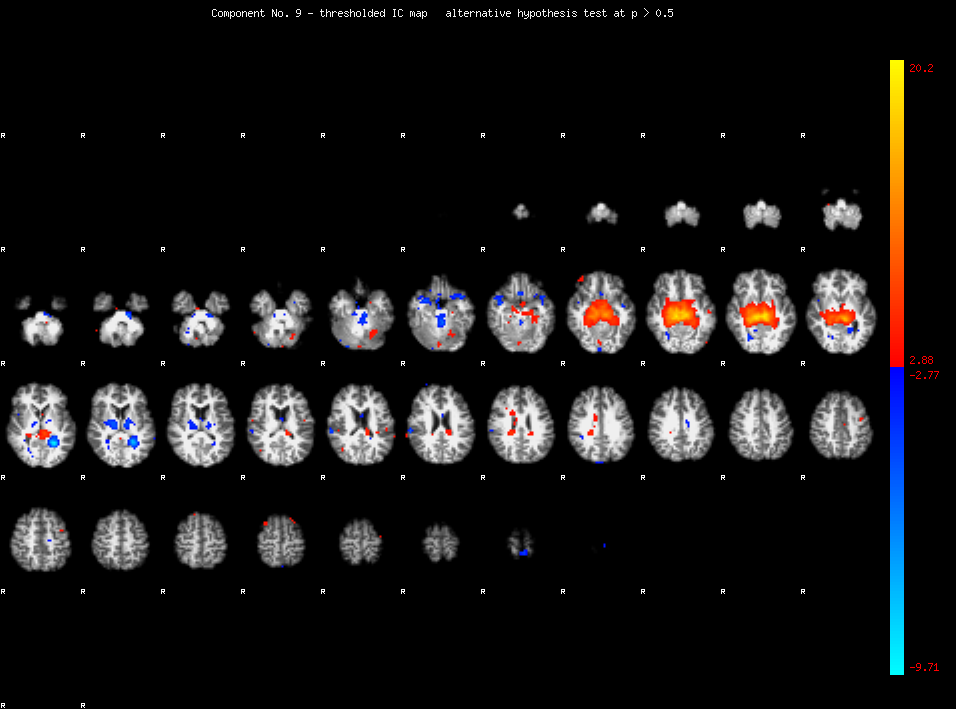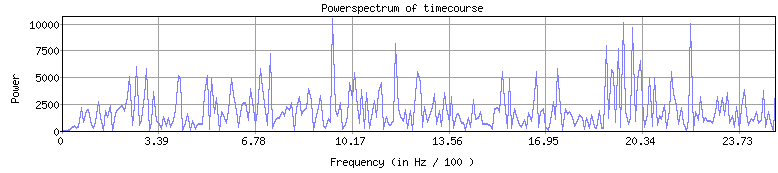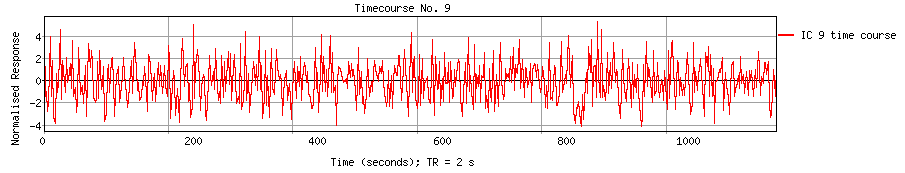 |
| --- | --- |
| 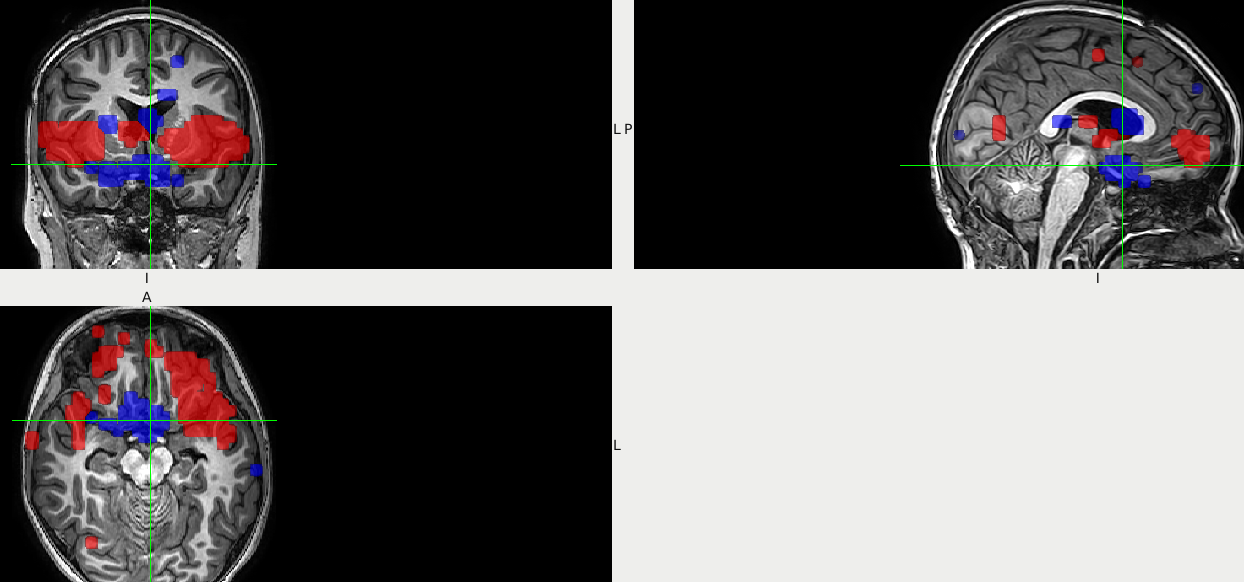  zstat 44 | 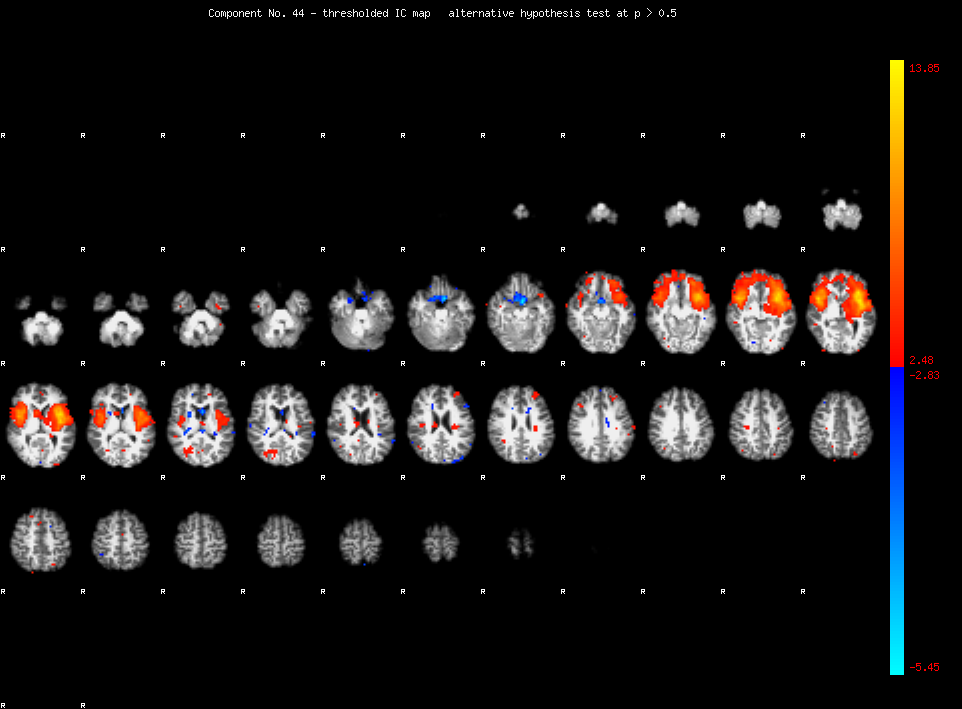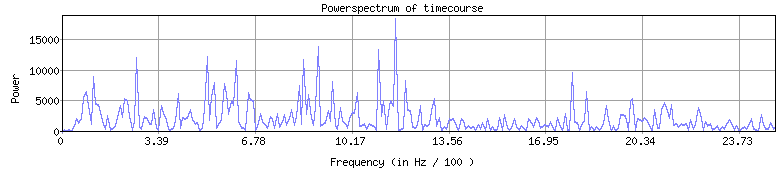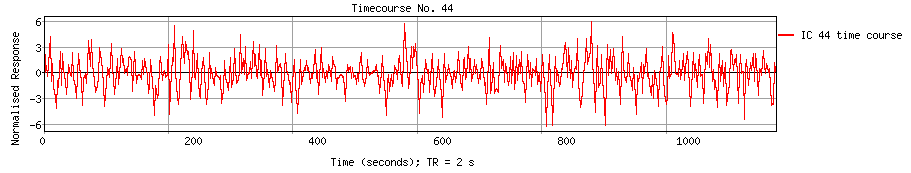 |
| 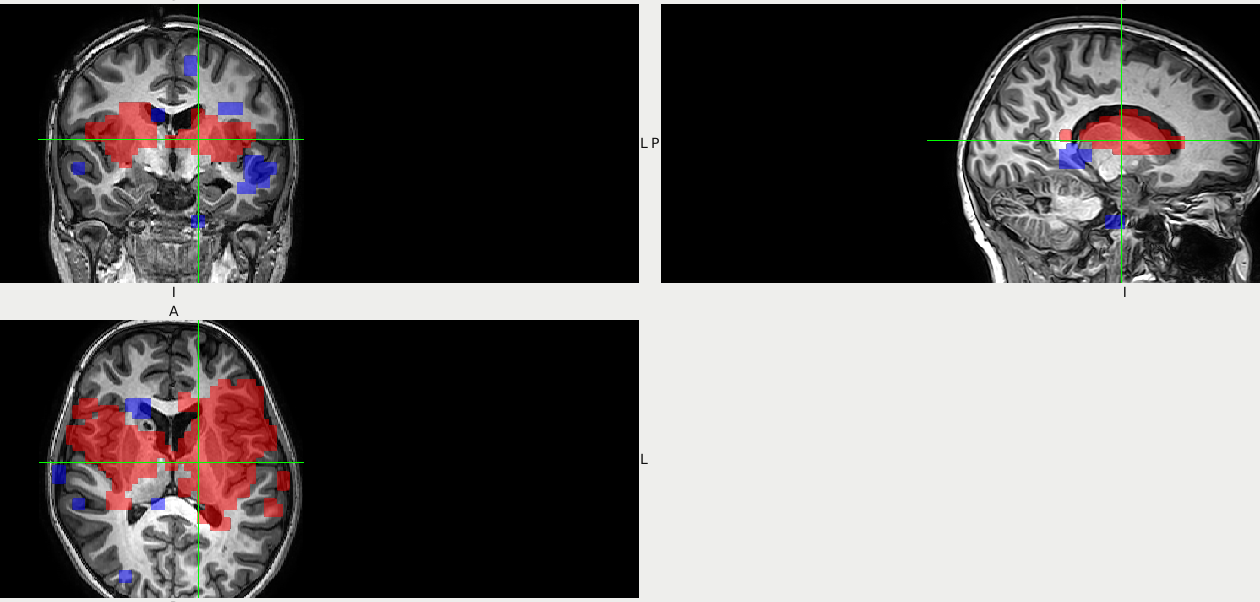  zstat 73 | 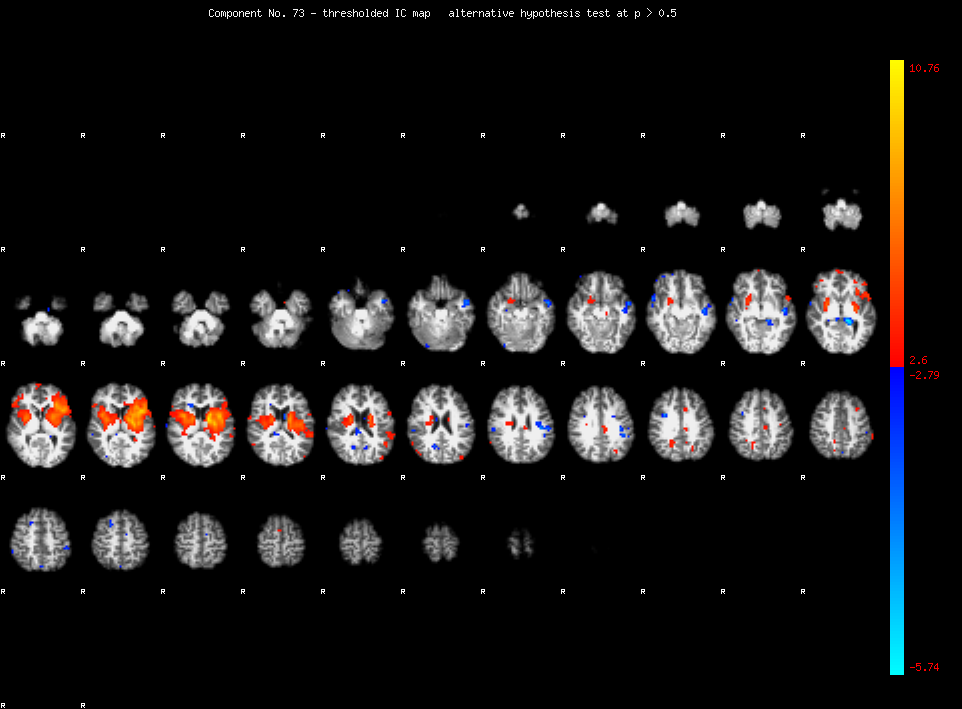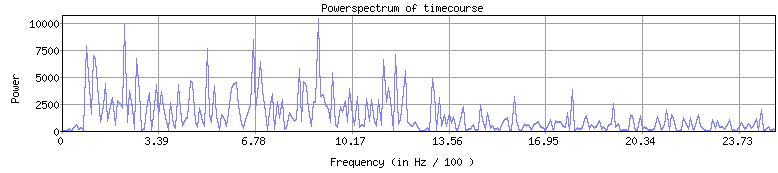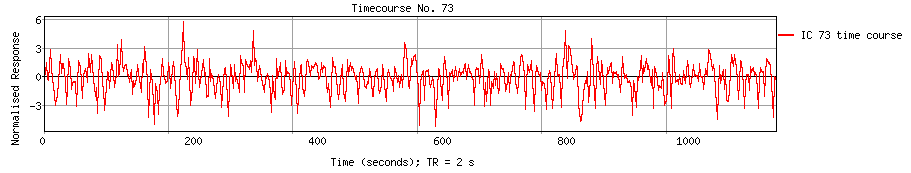 |
| 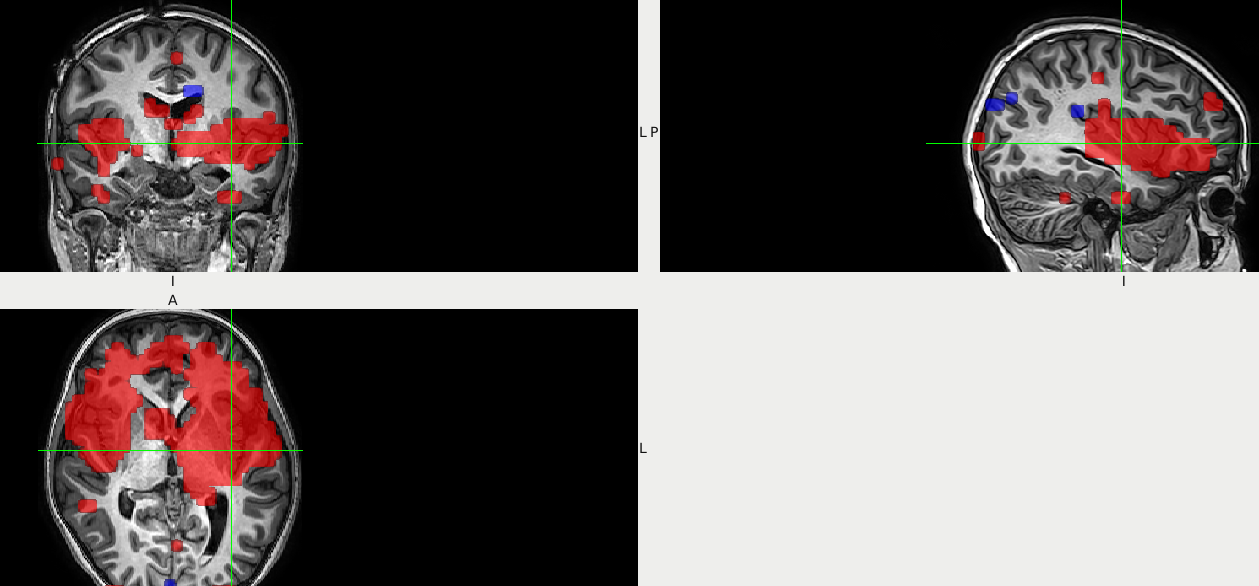  zstat 44 | 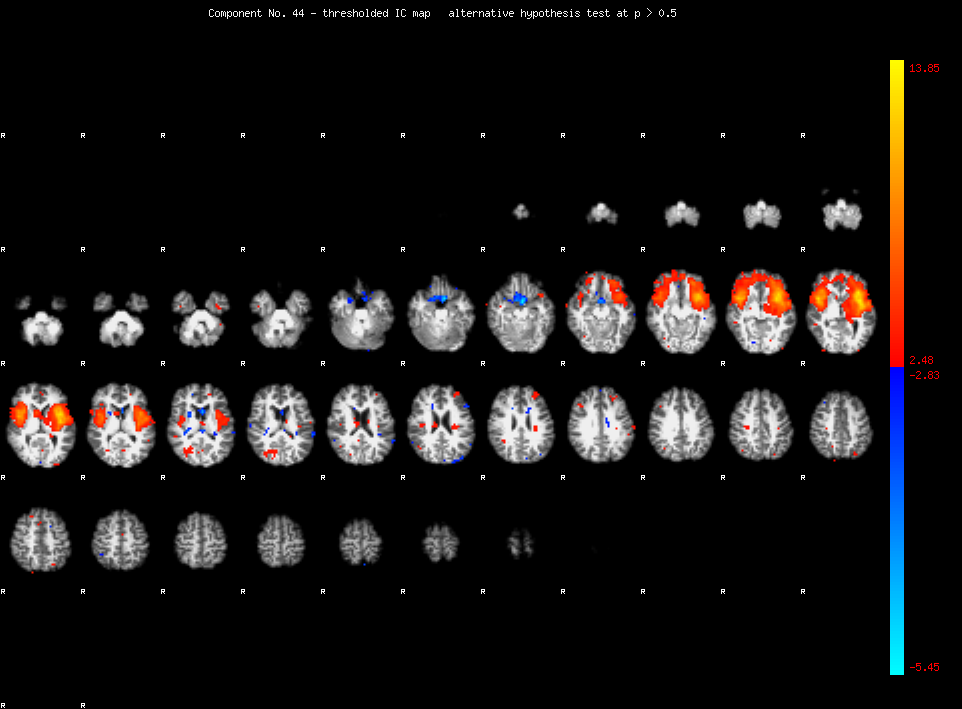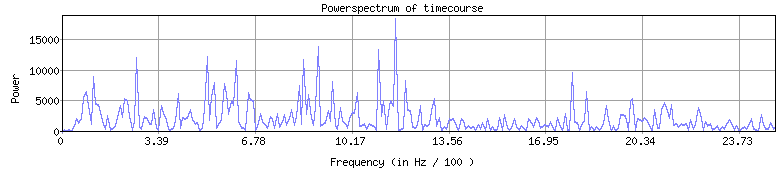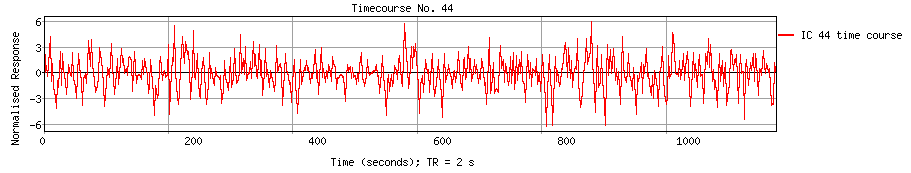 |
| 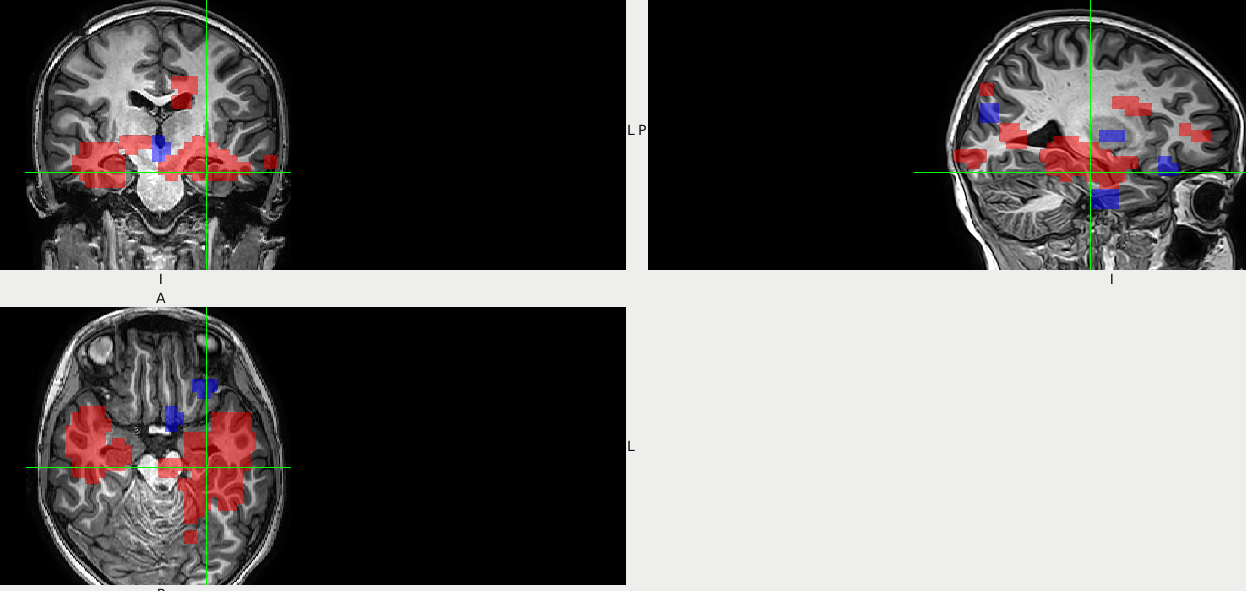  zstat 74 | 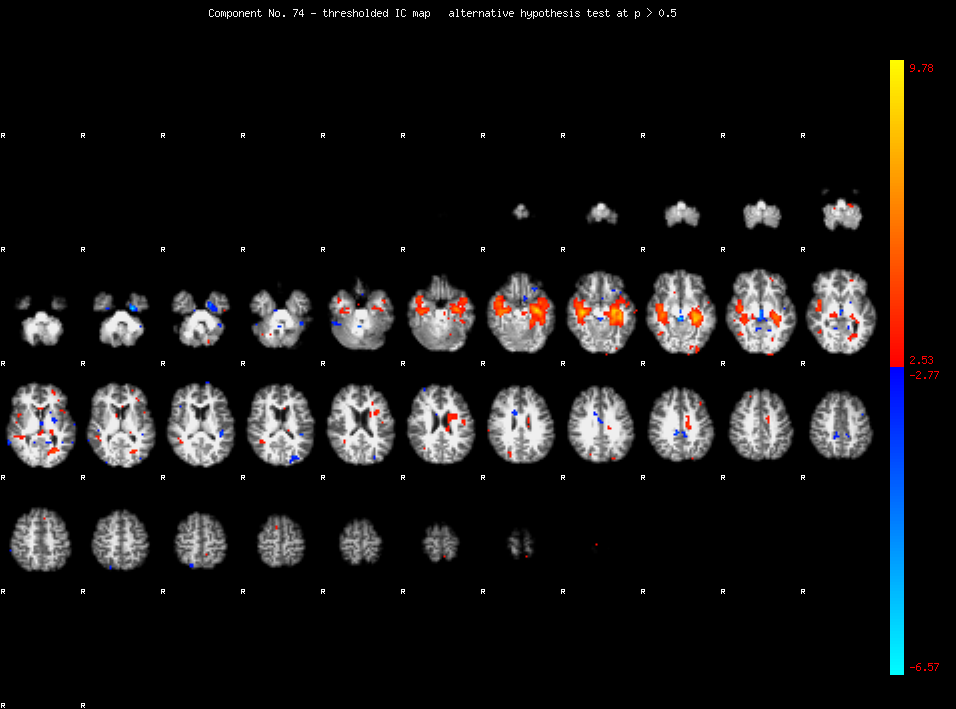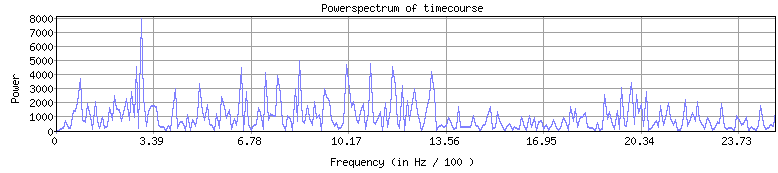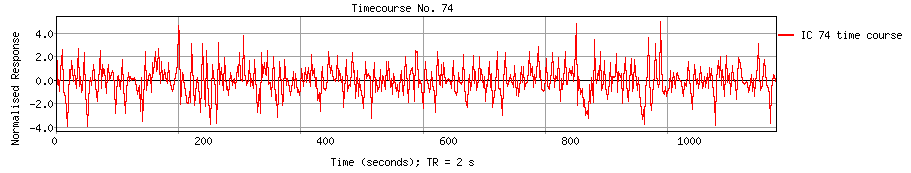 |
| 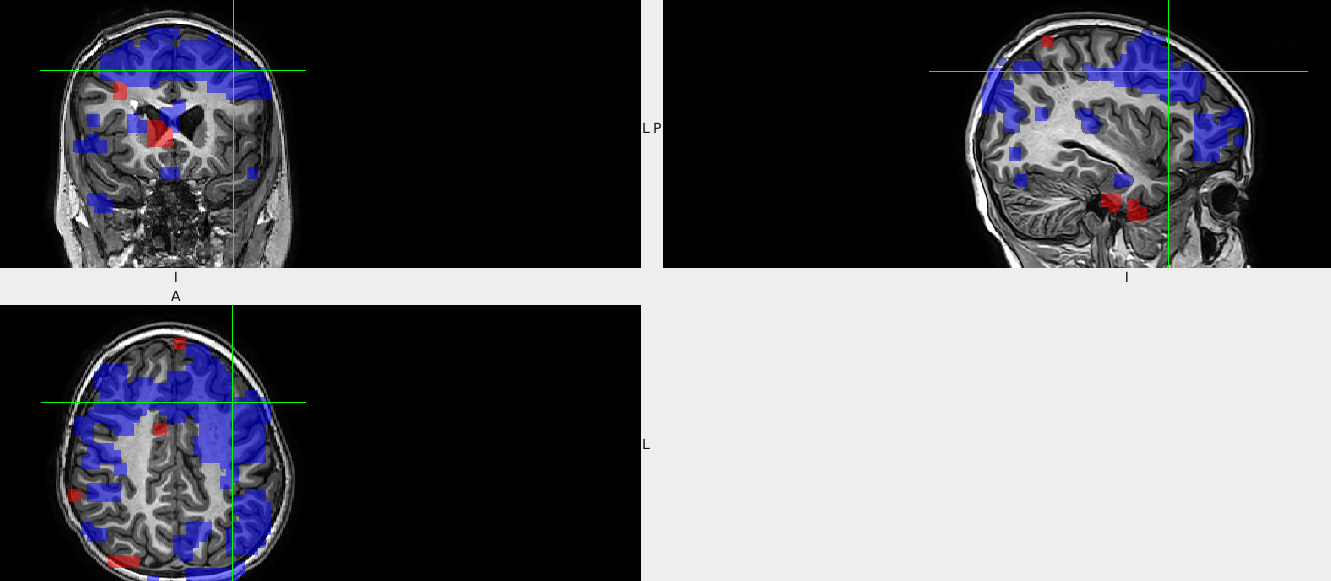  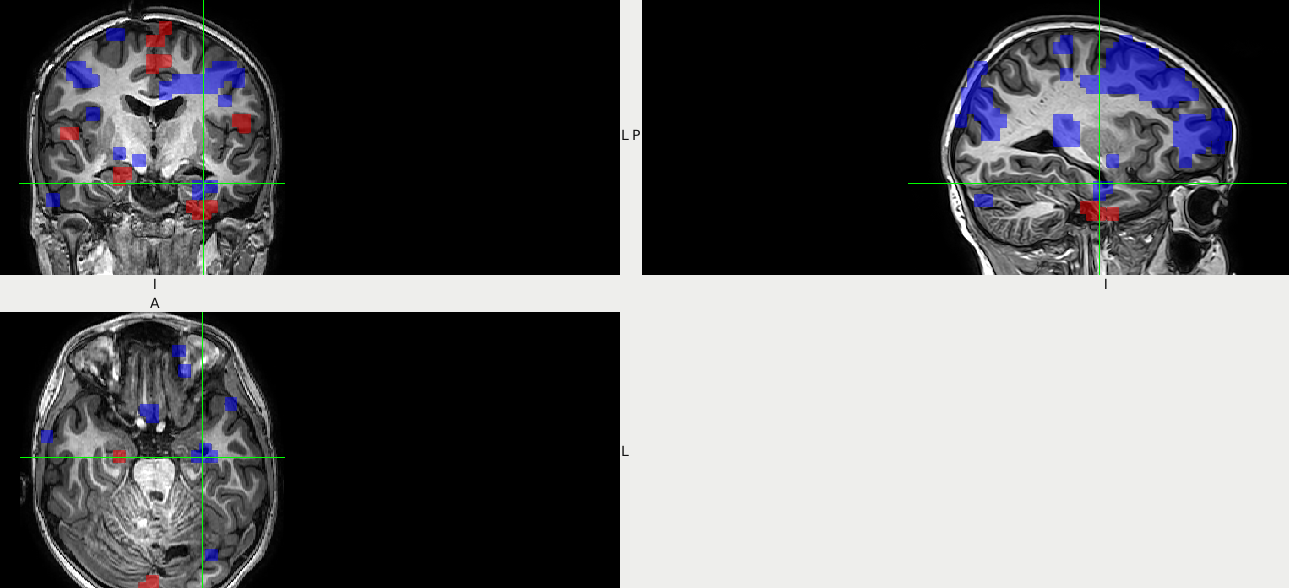  zstat 86 | 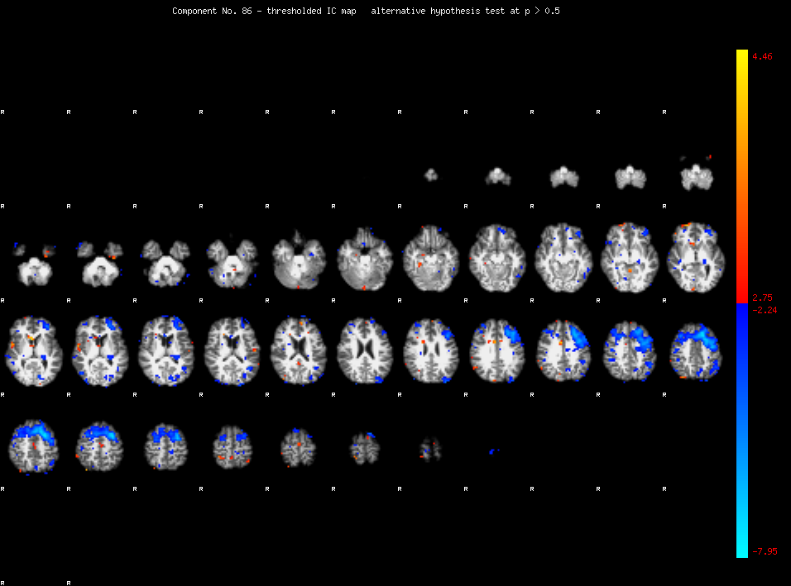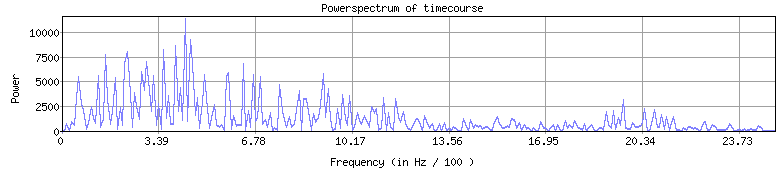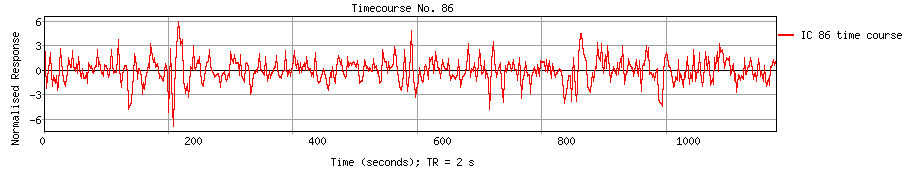 |
| 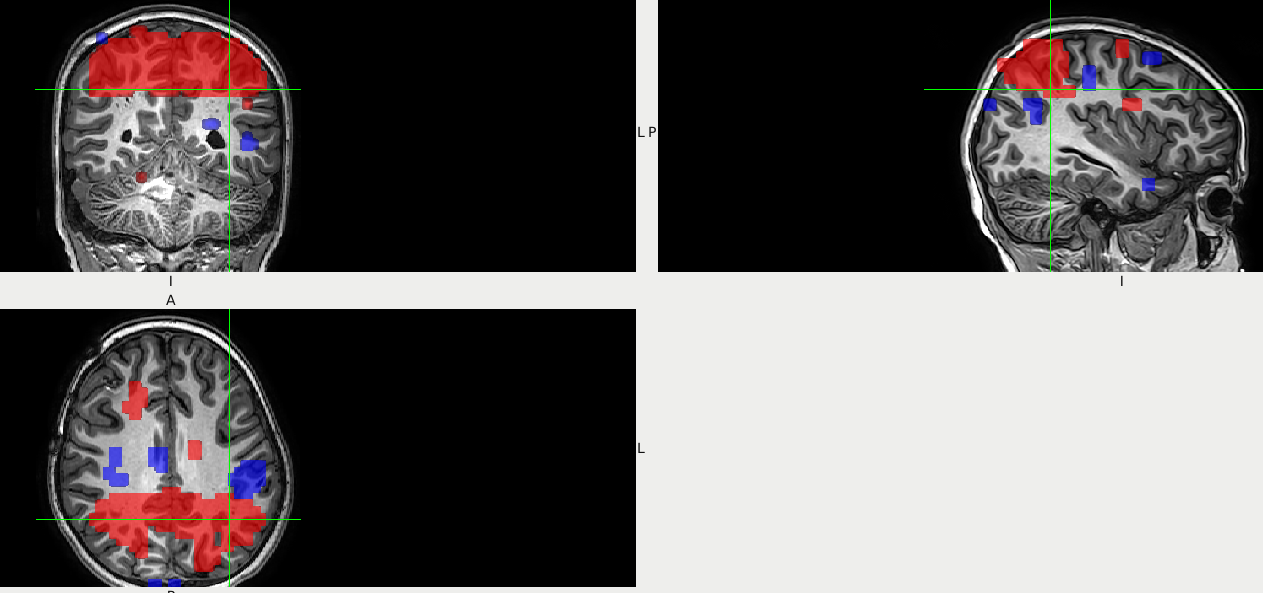  zstat 41 | 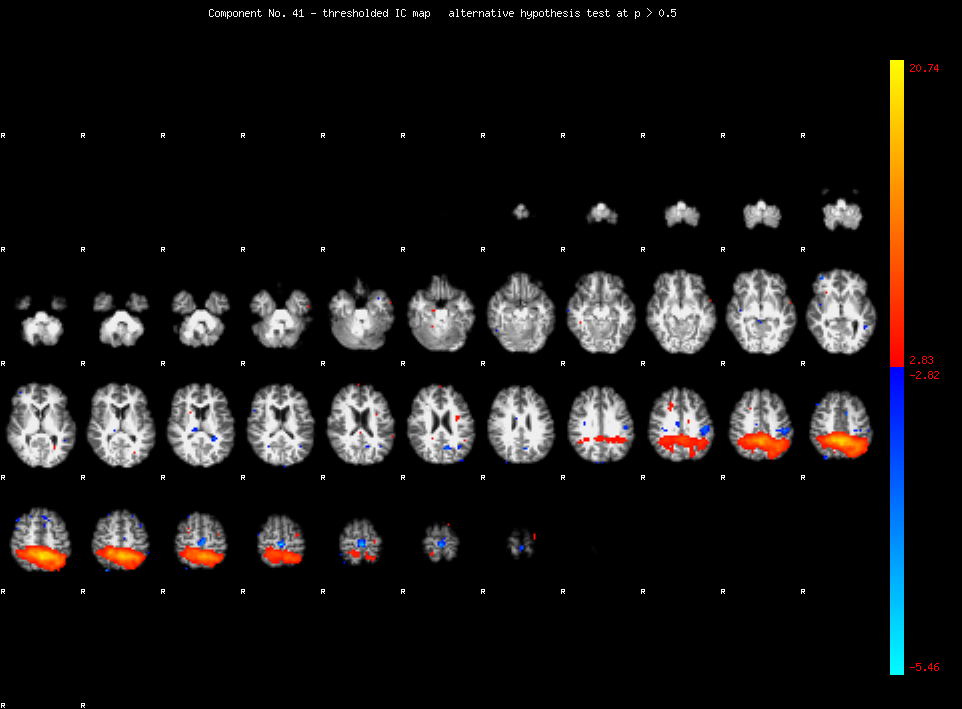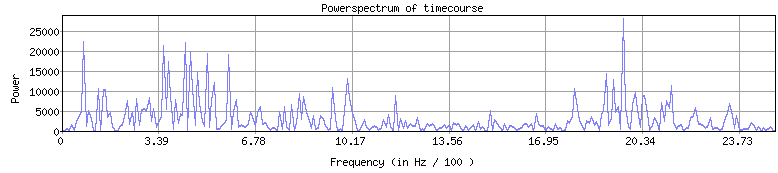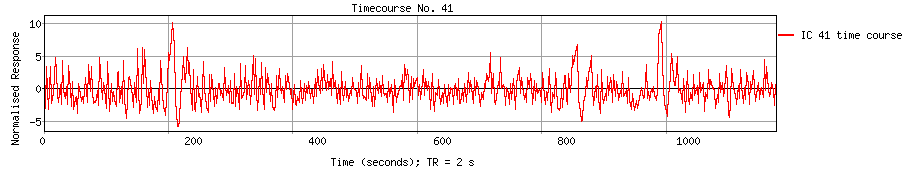 |
| 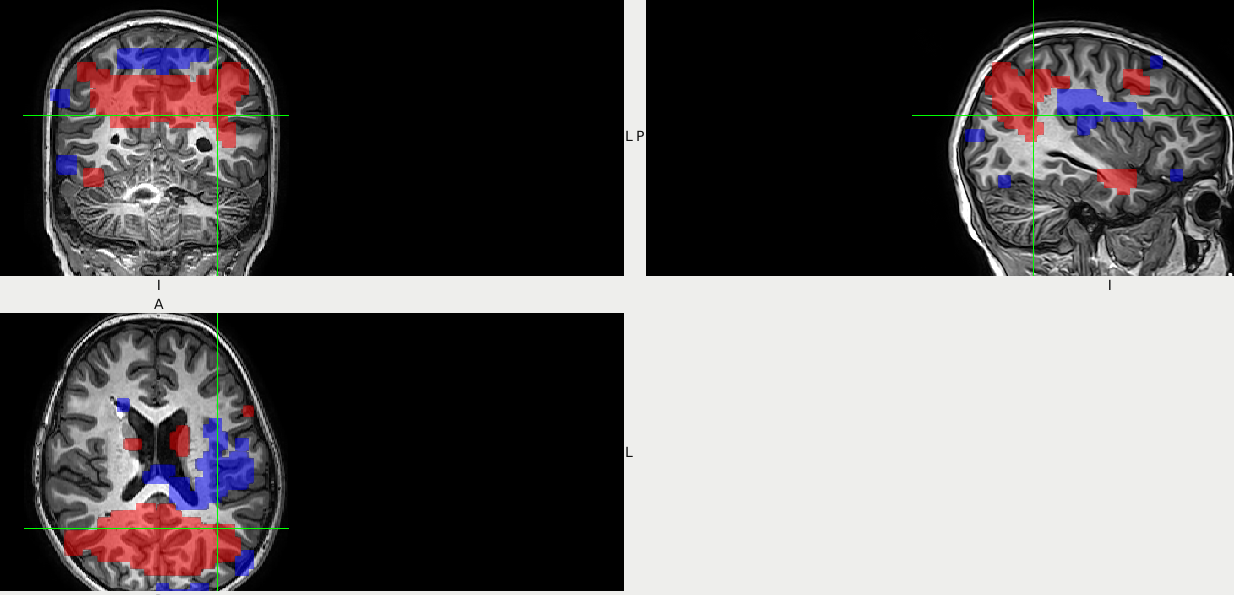  zstat 37 | 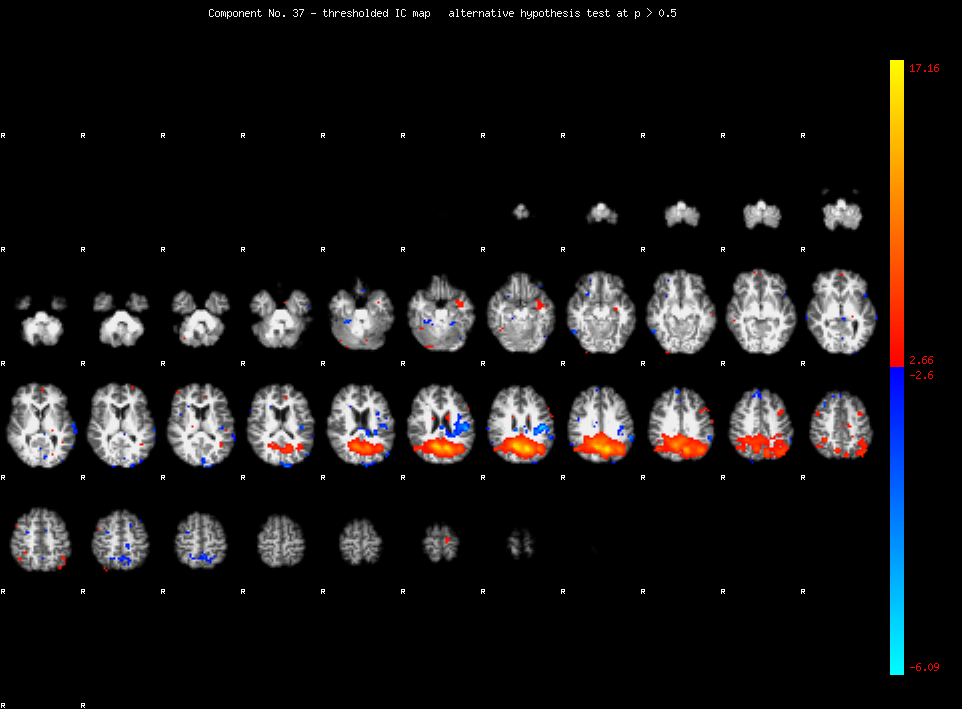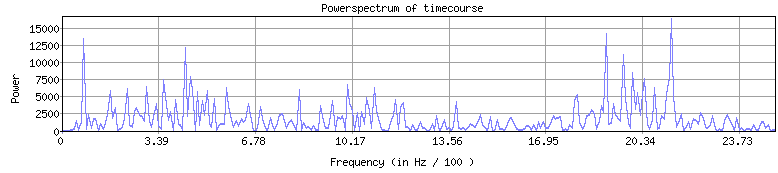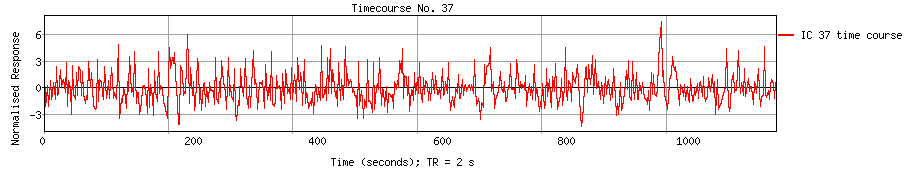 |

| **Atypical RSN** |
| --- |

|  |  |
| --- | --- |
|  |  |

Motor

| 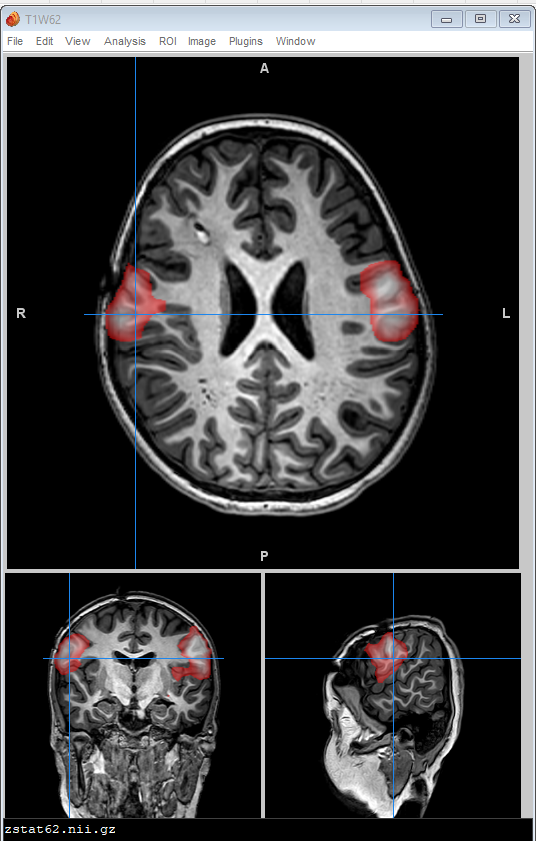  zstat 62 | 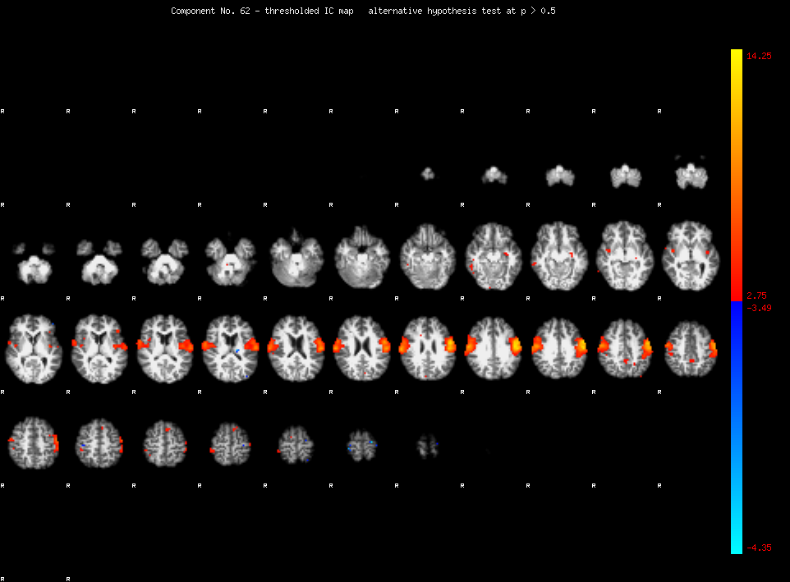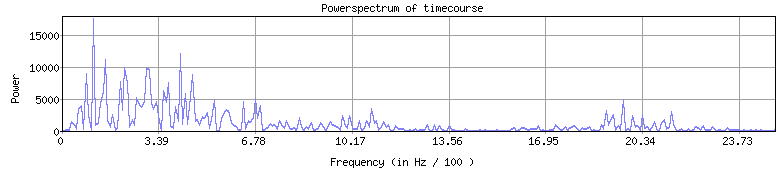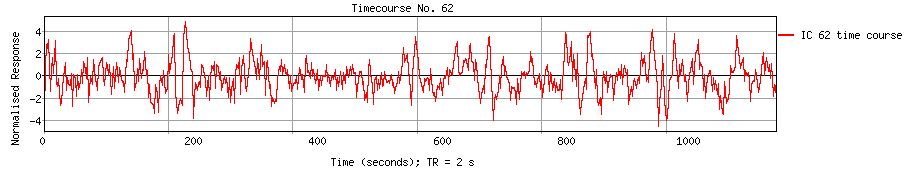 |
| --- | --- |
| 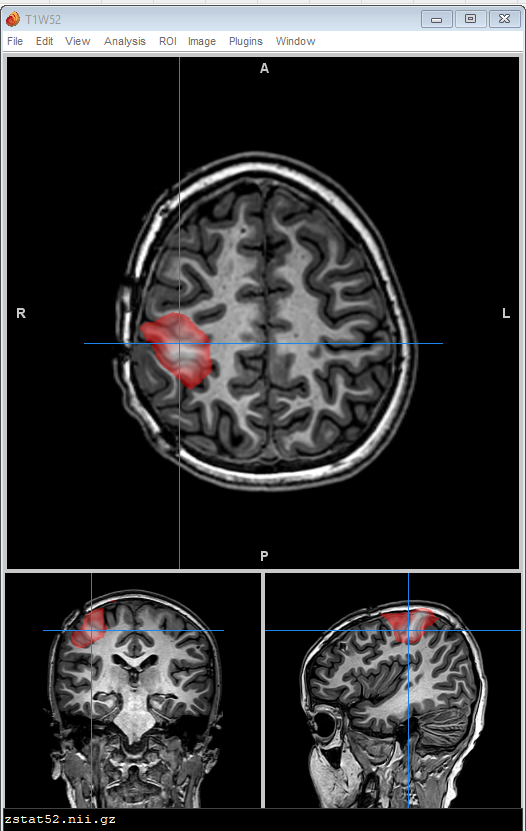  zstat 52 | 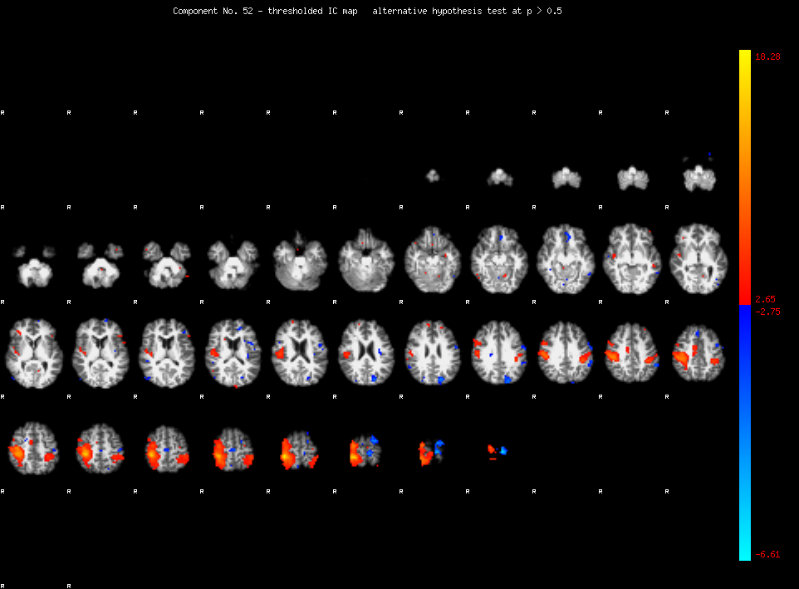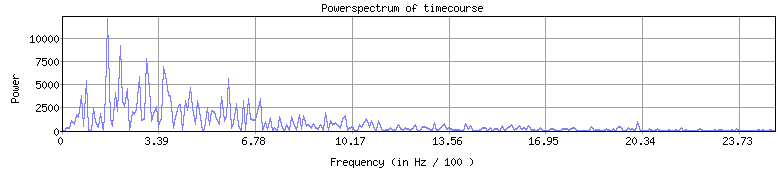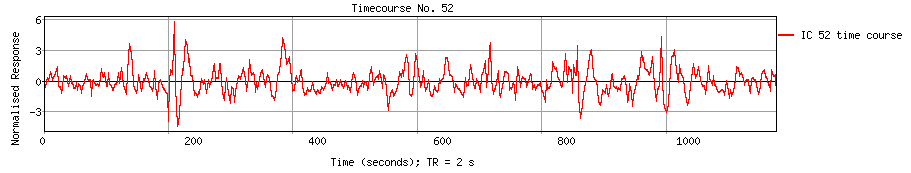 |
| 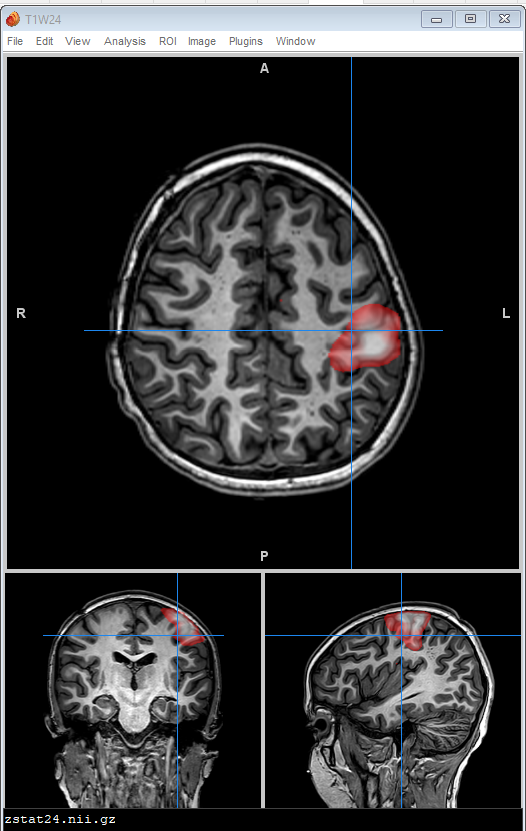  zstat 24 | 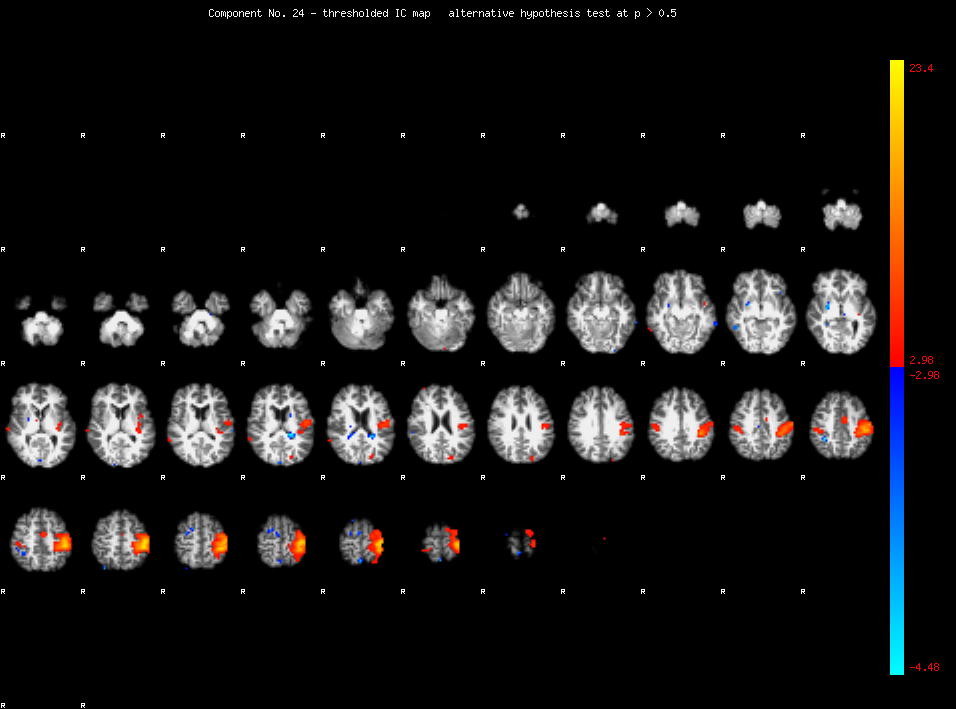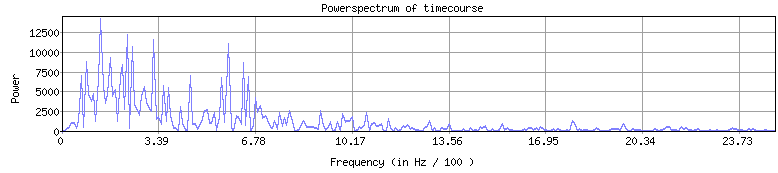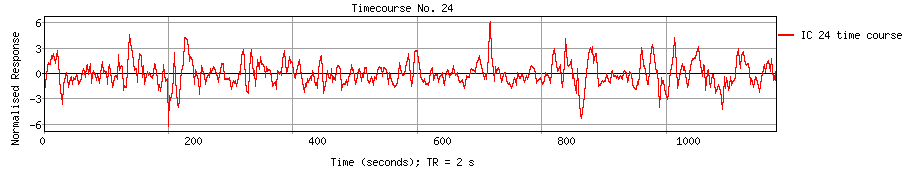 |
|  |  |
| 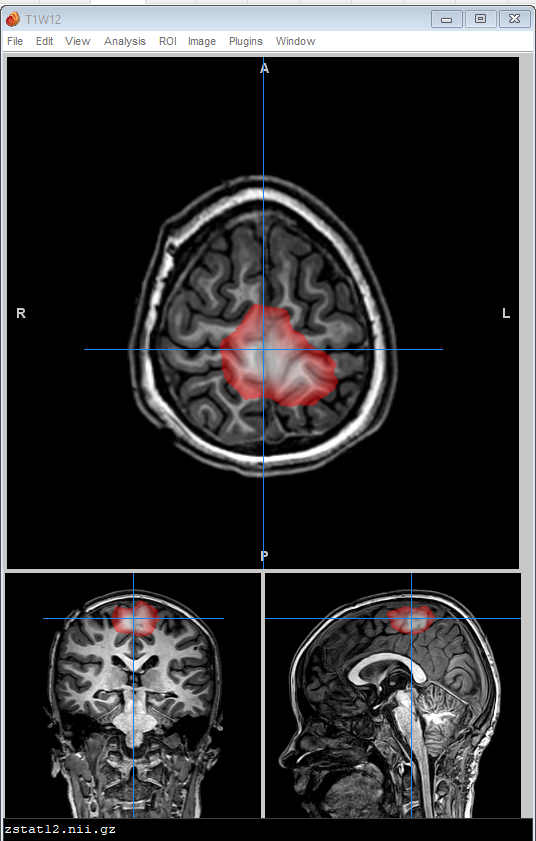  zstat 12 | 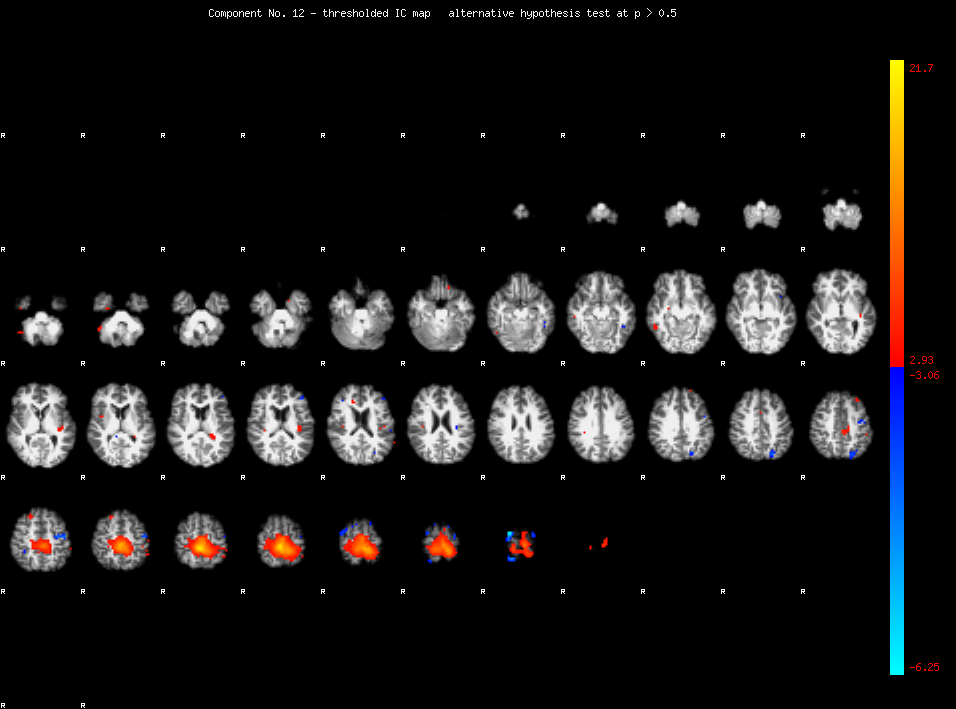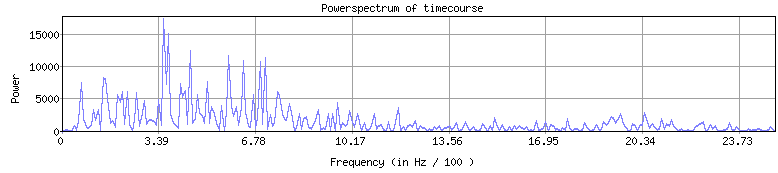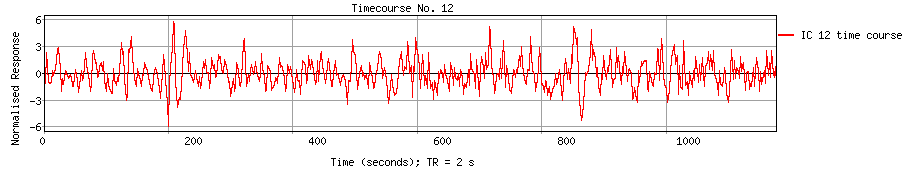 |
|  |  |

Language

| 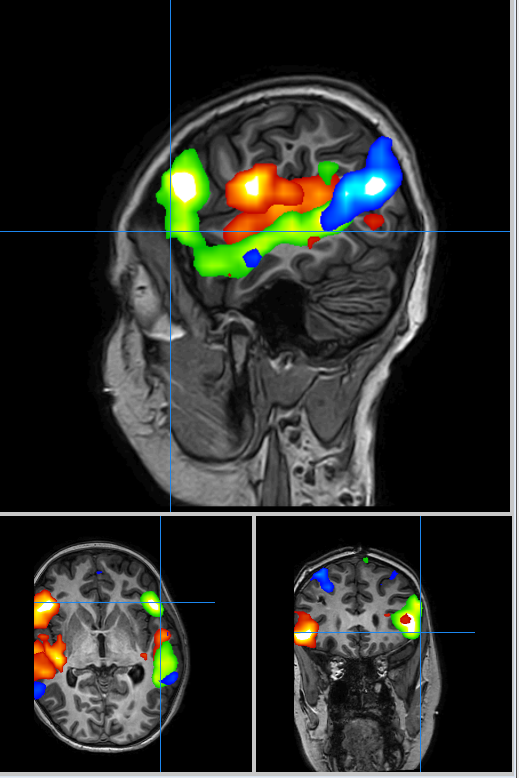 | 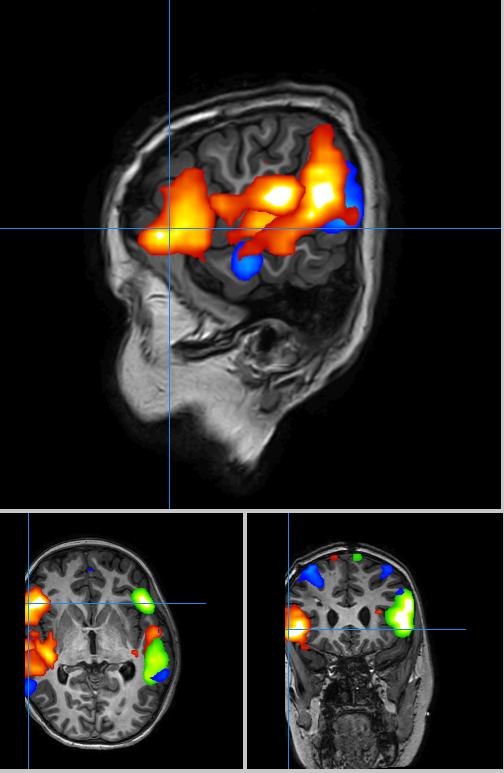 |
| --- | --- |
| Left language summary image | Right language summary image |
| 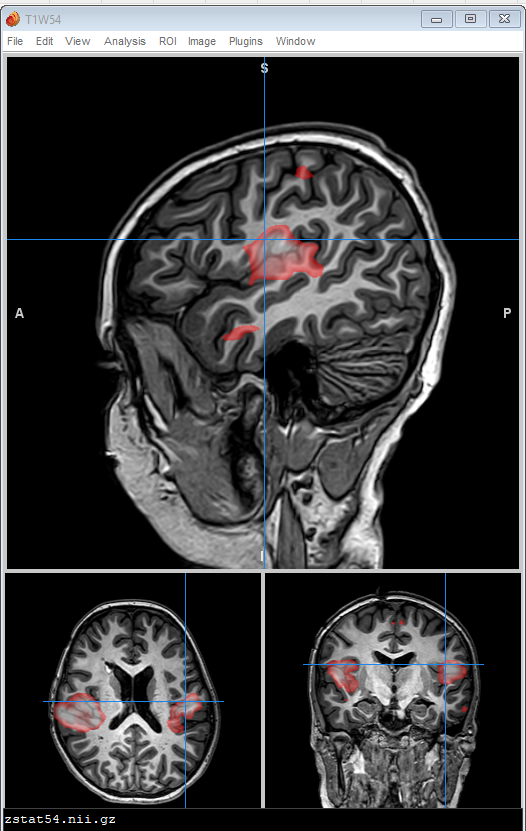  zstat 54 | 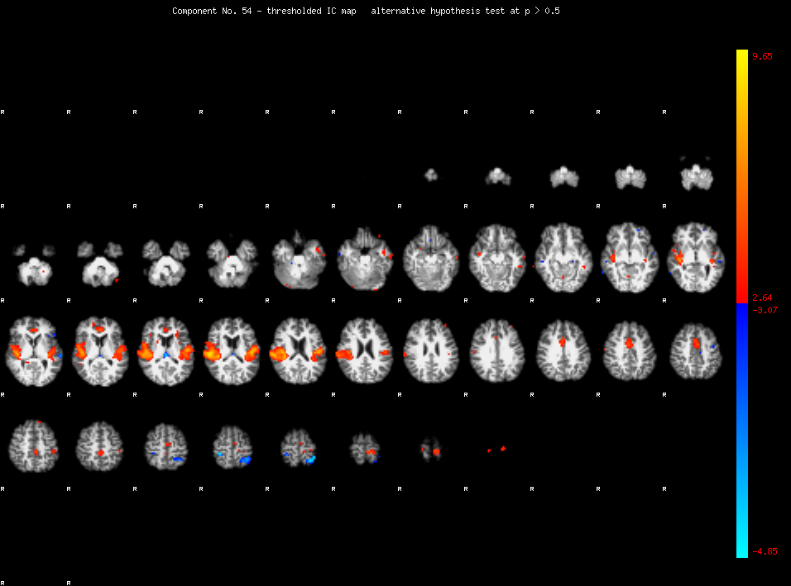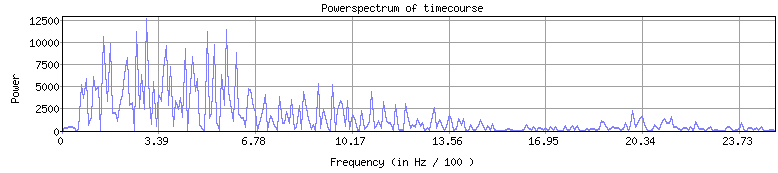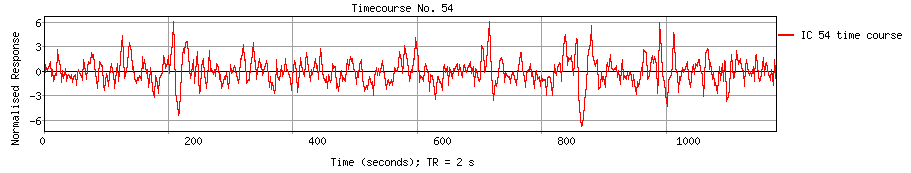 |
| 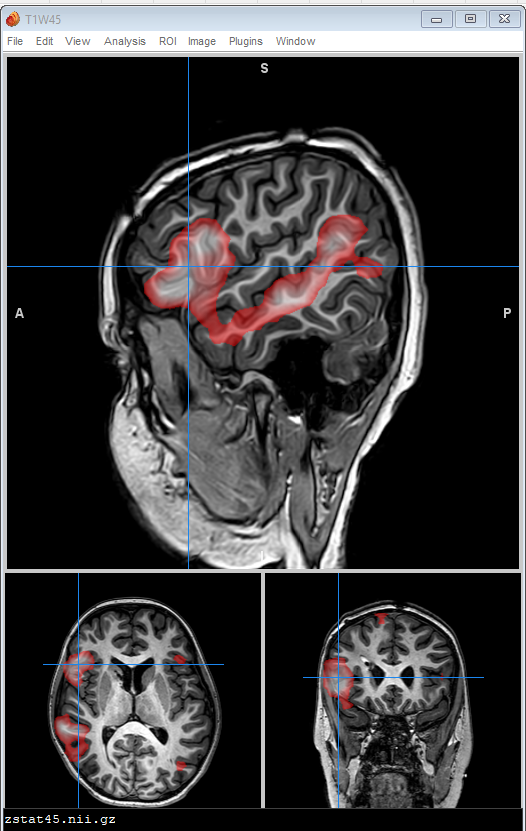  zstat 45 | 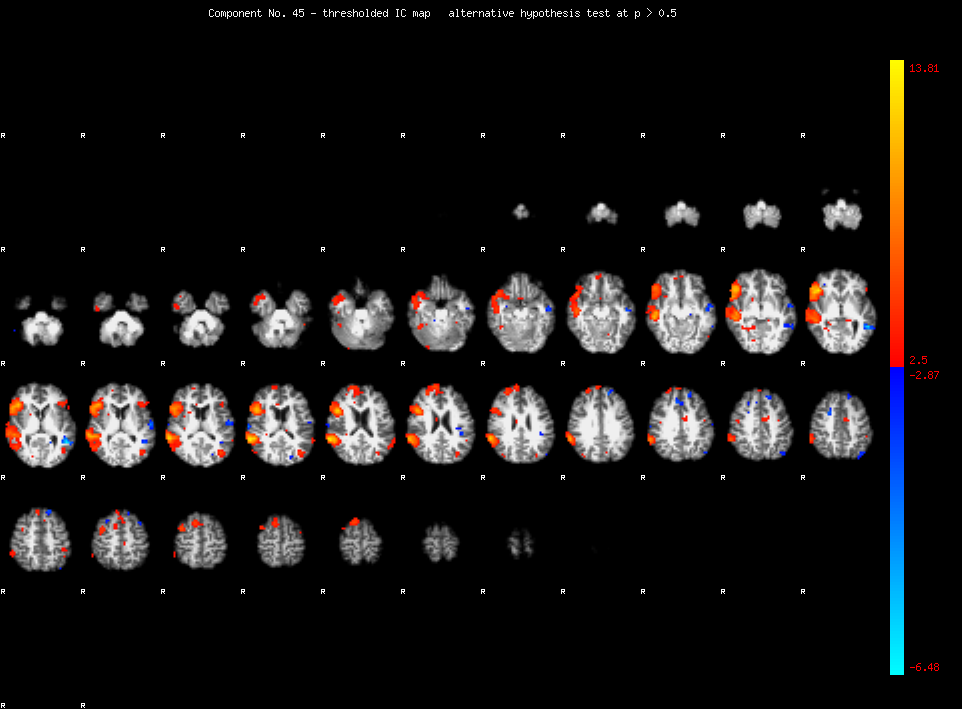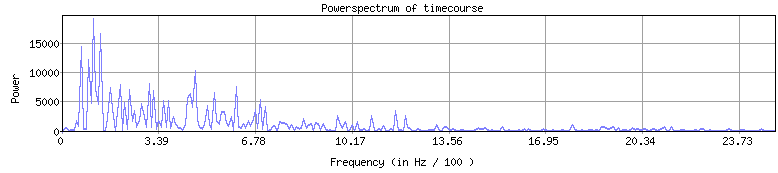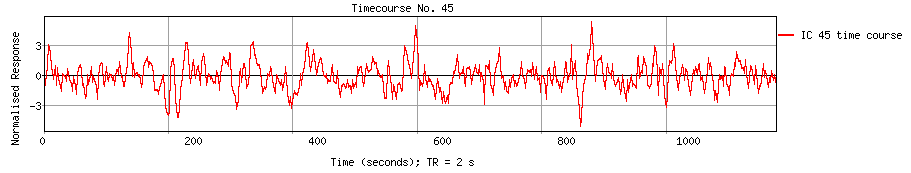 |
| 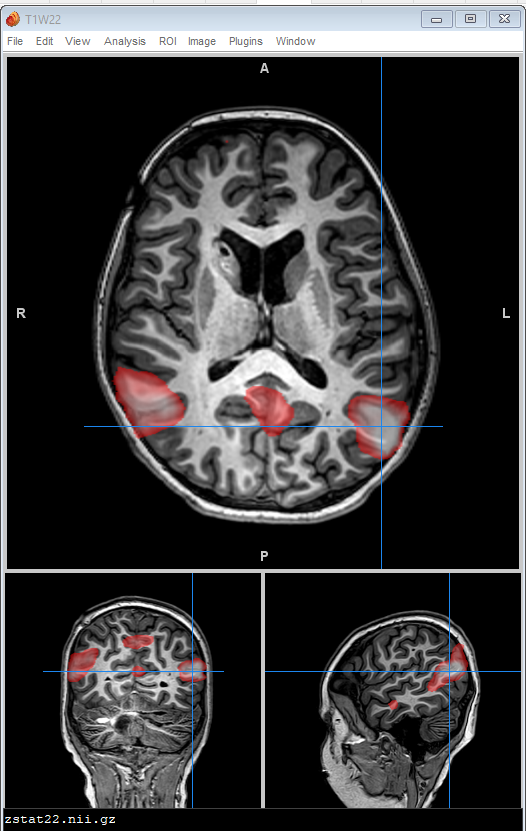  zstat 22 | 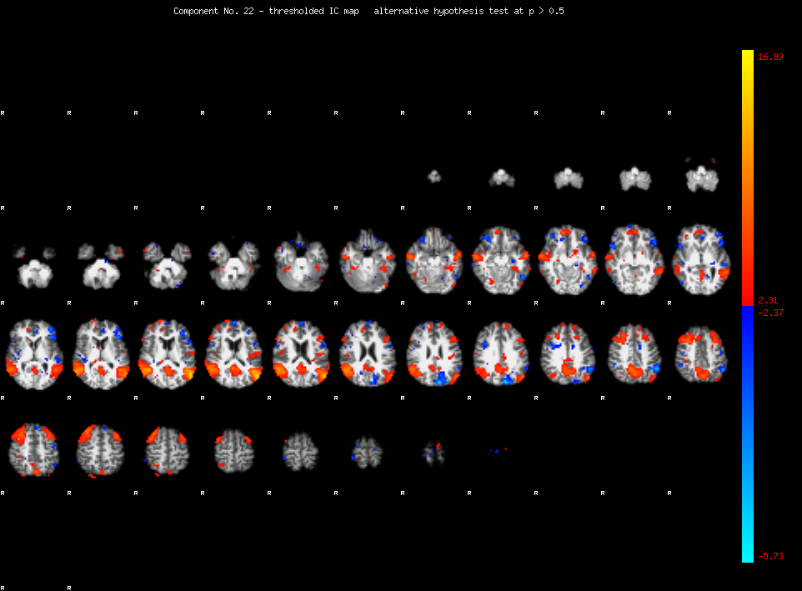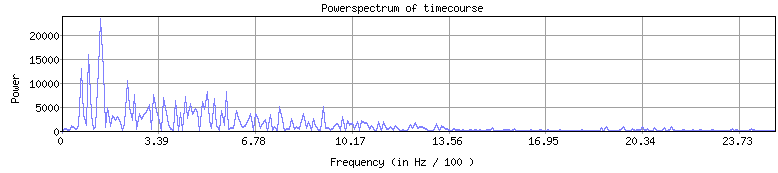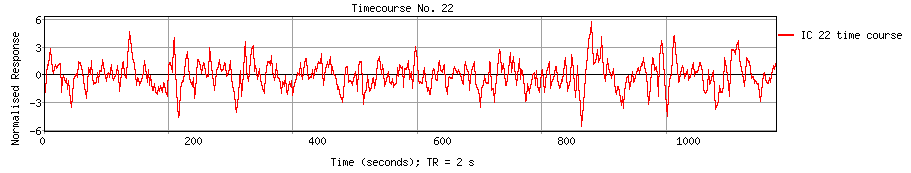 |
| 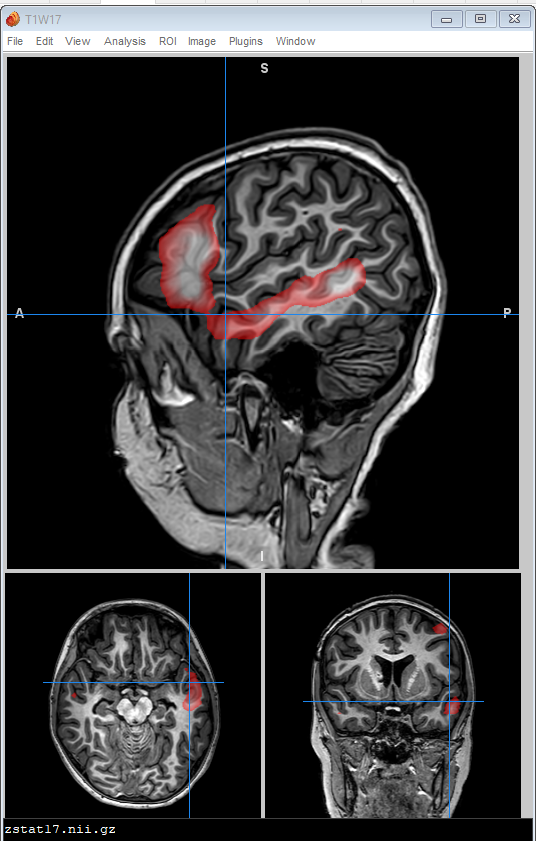  zstat 17 | 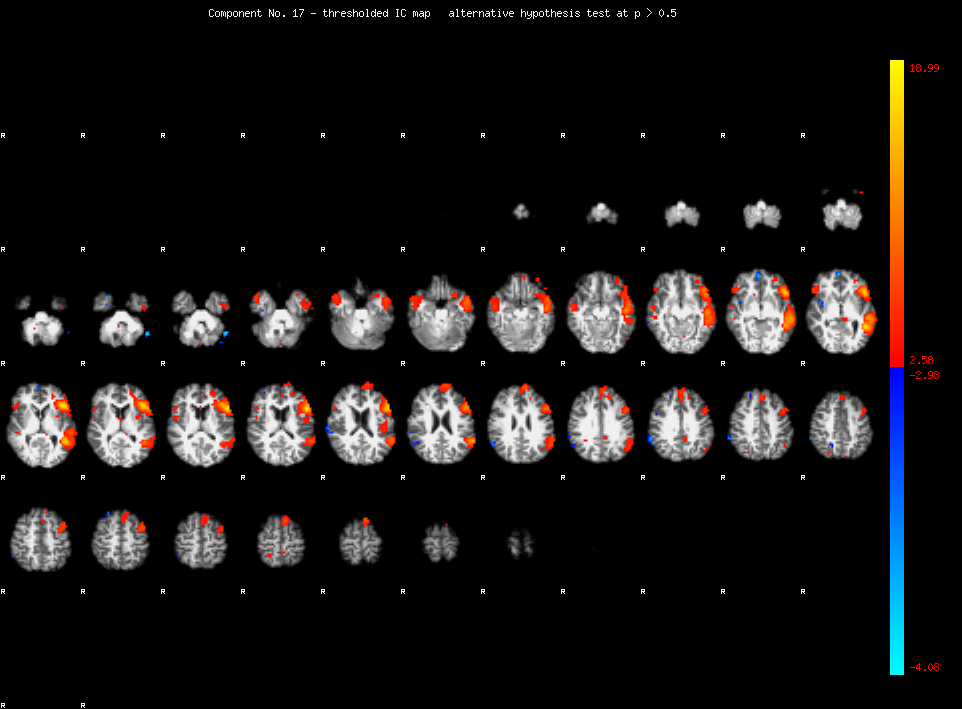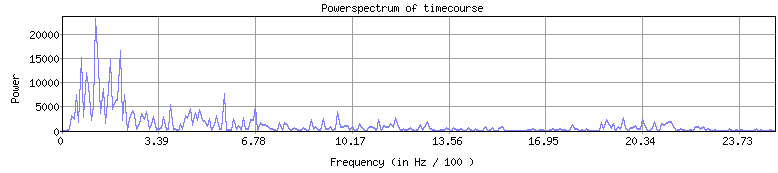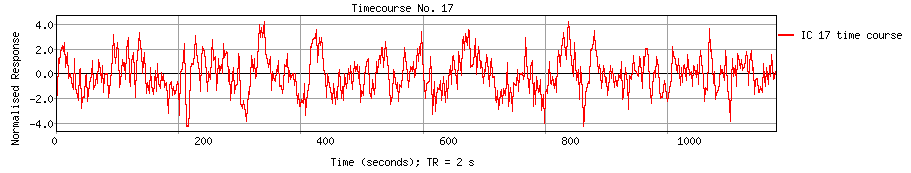 |
| 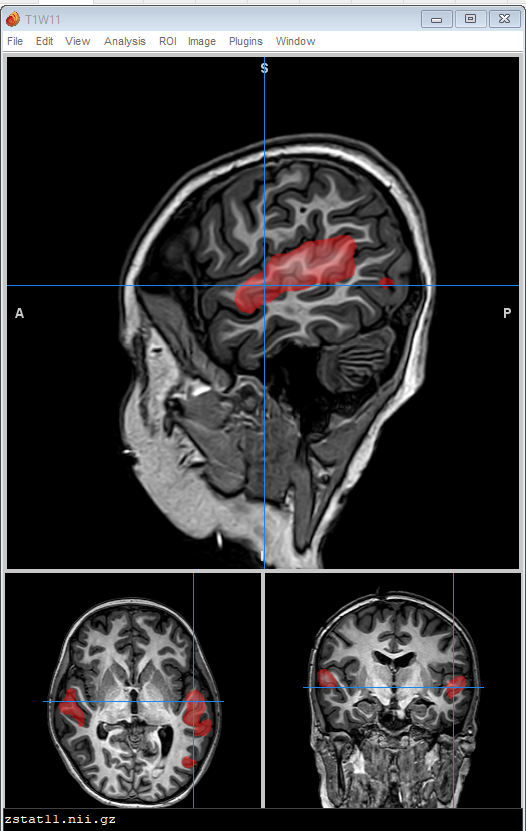  zstat 11 | 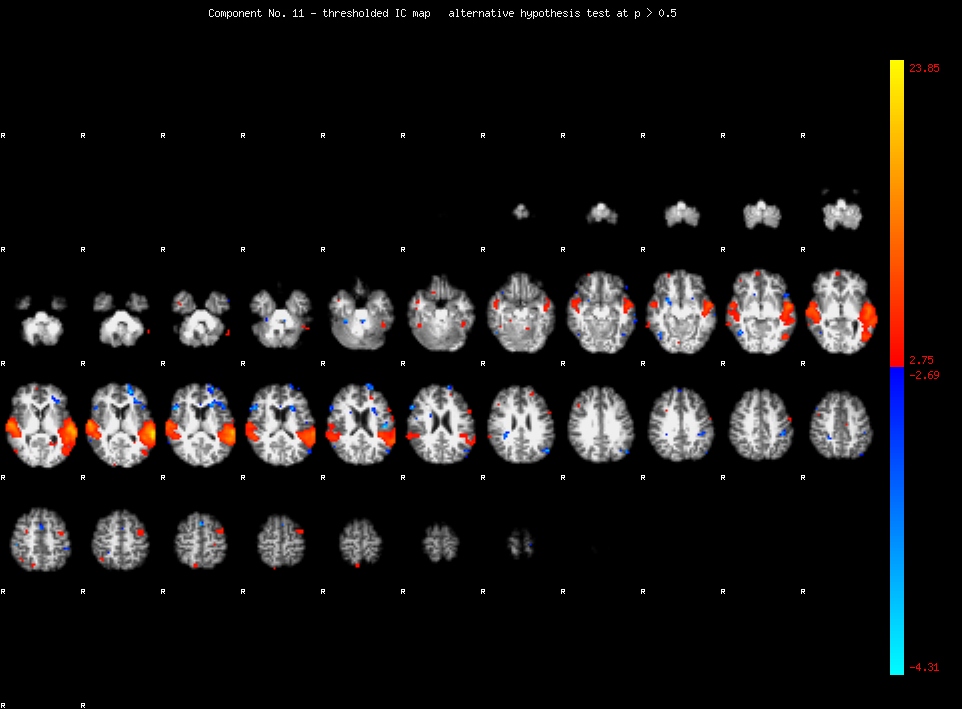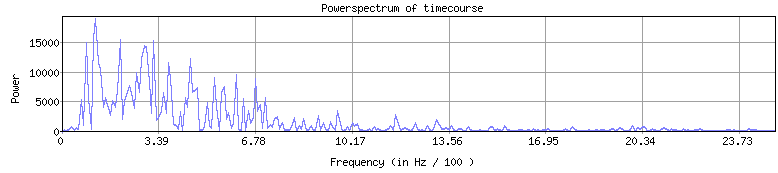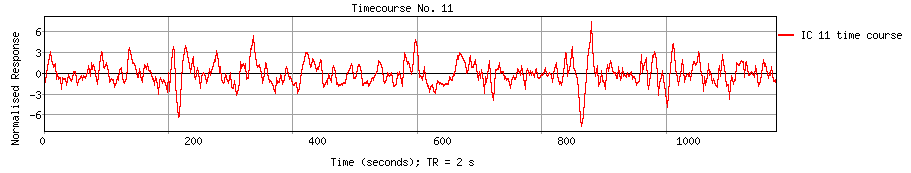 |
|  |  |

Parietal

| 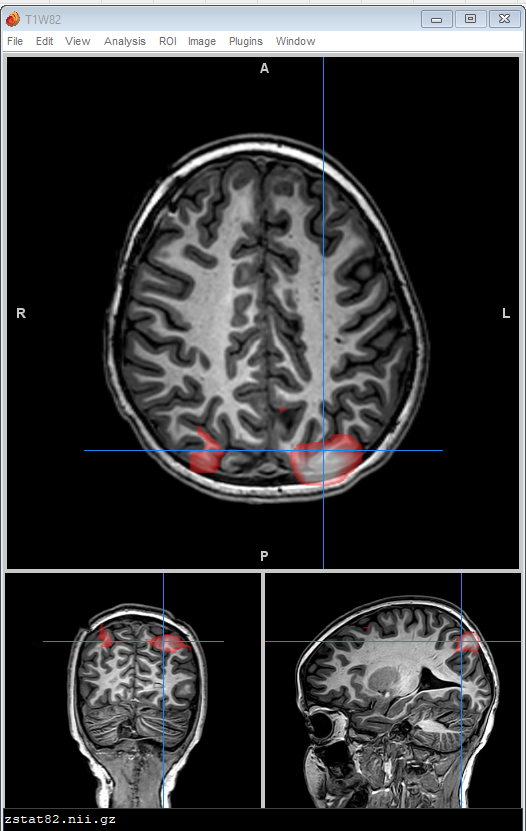  zstat 82 | 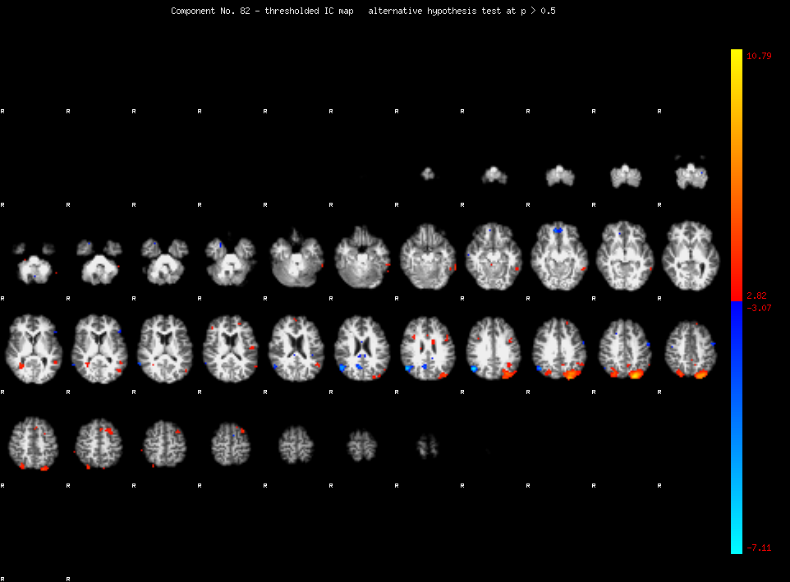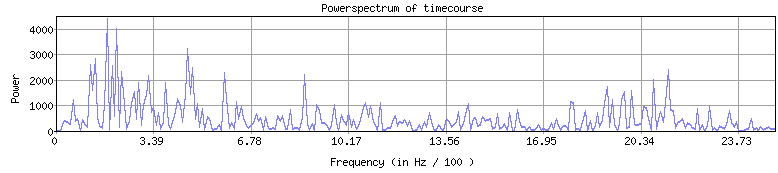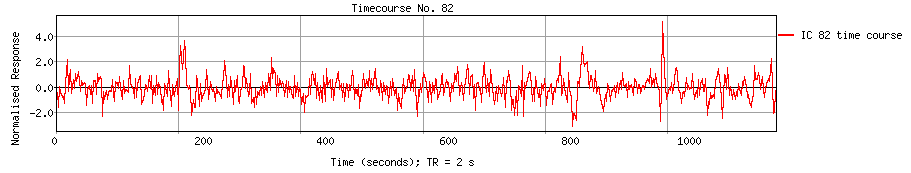 |
| --- | --- |
| 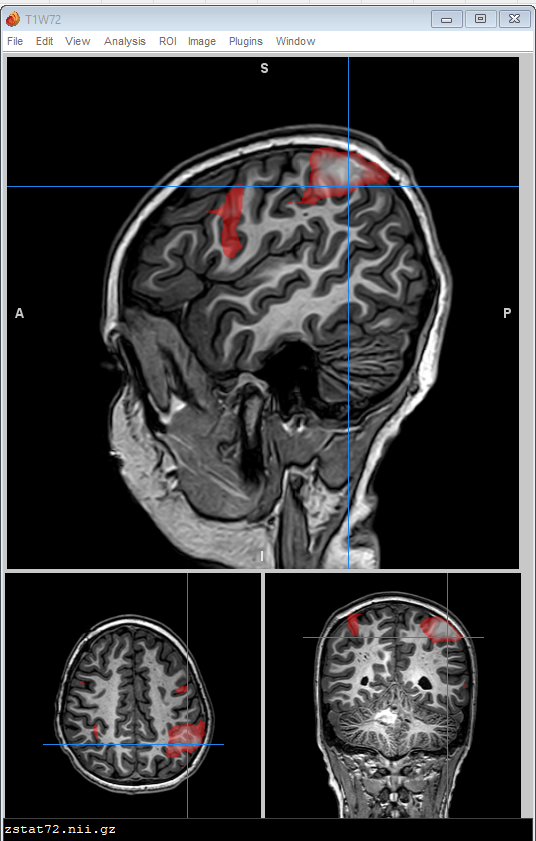  zstat 72 | 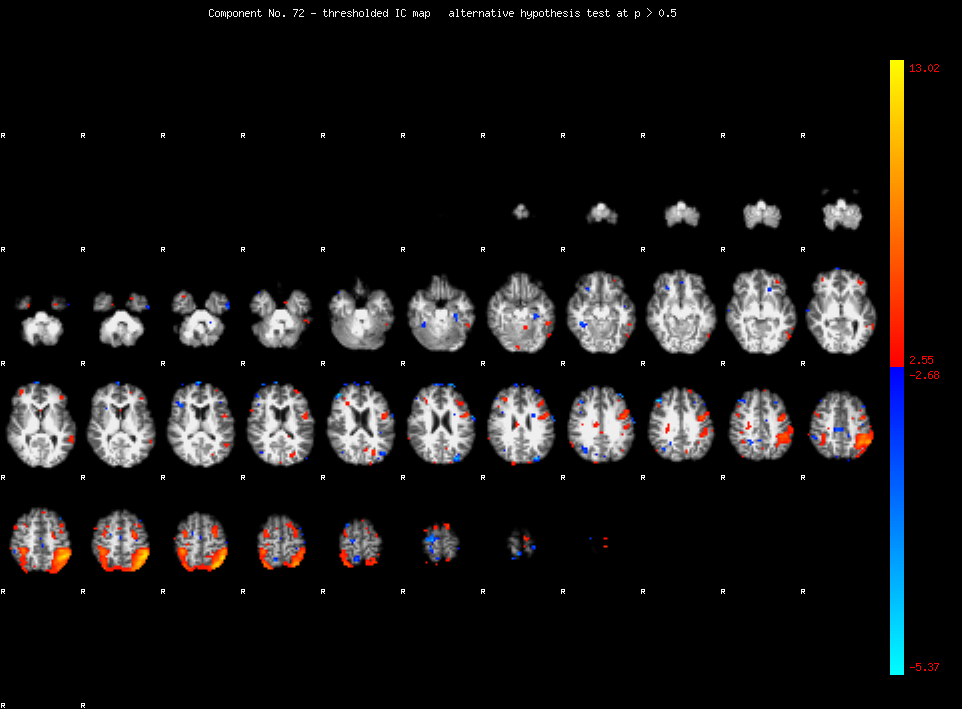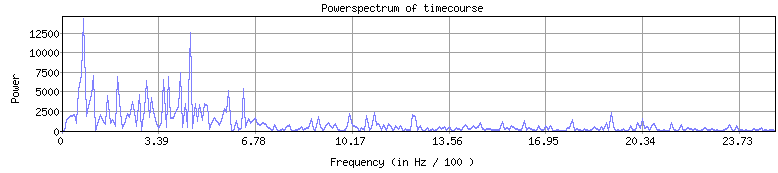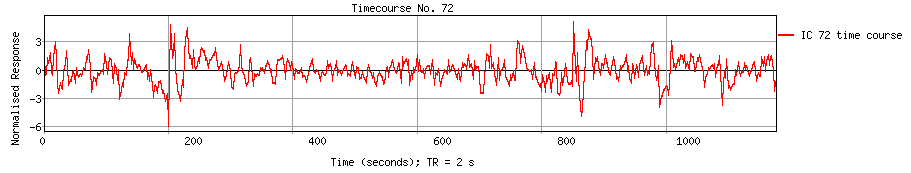 |
|  |  |
| 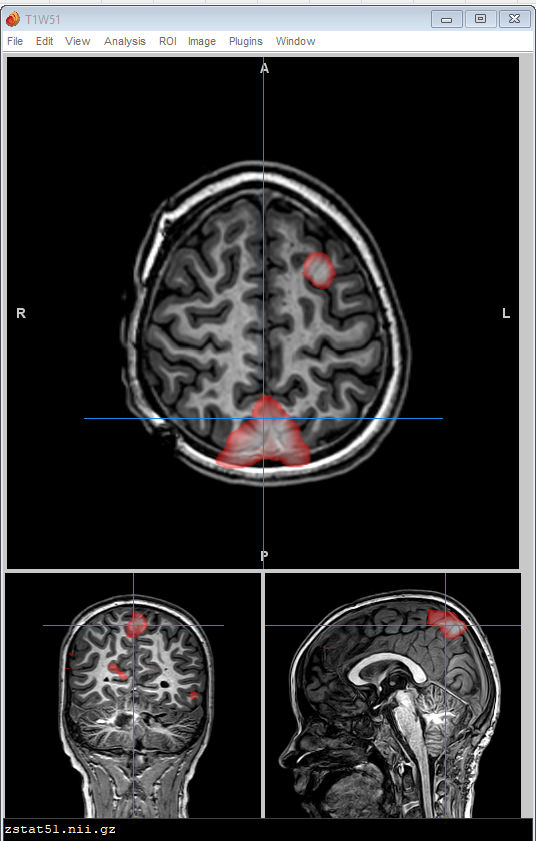  zstat 51 | 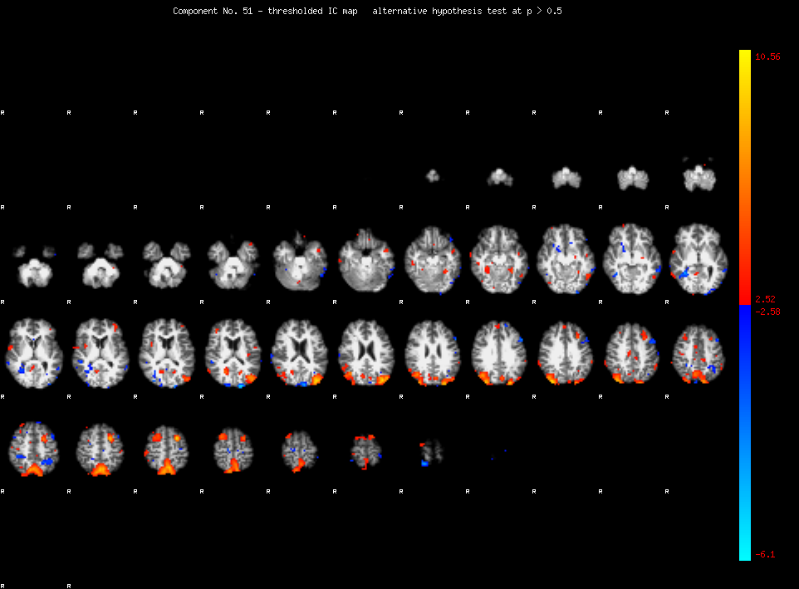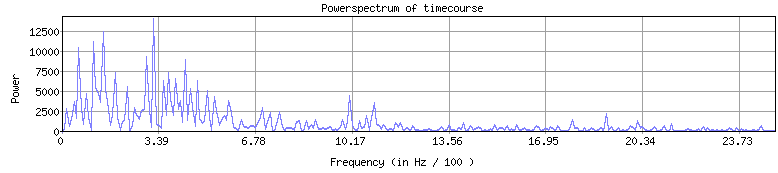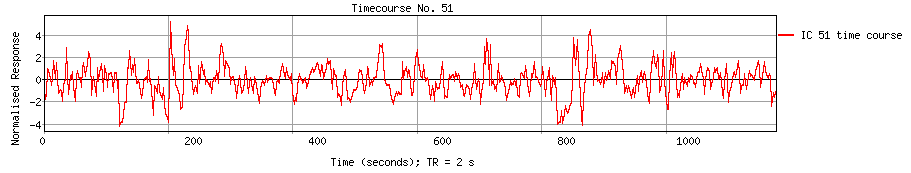 |
| 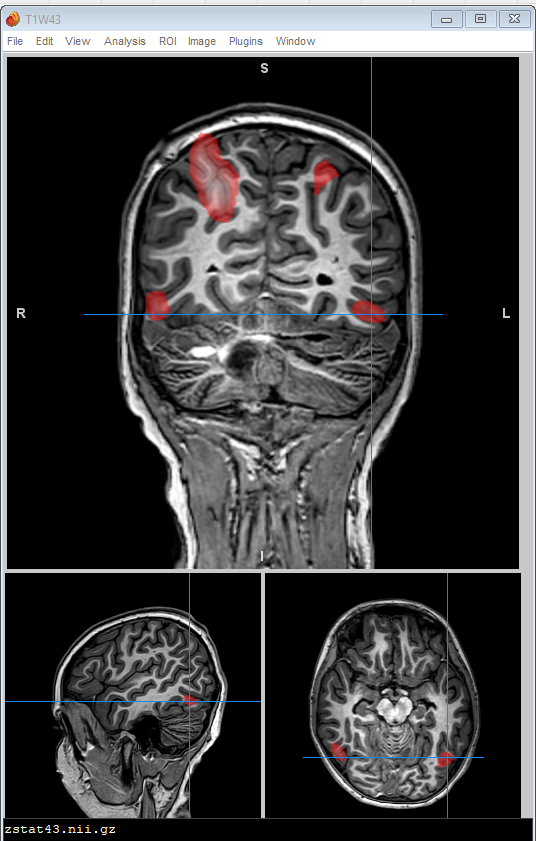  zstat 43 | 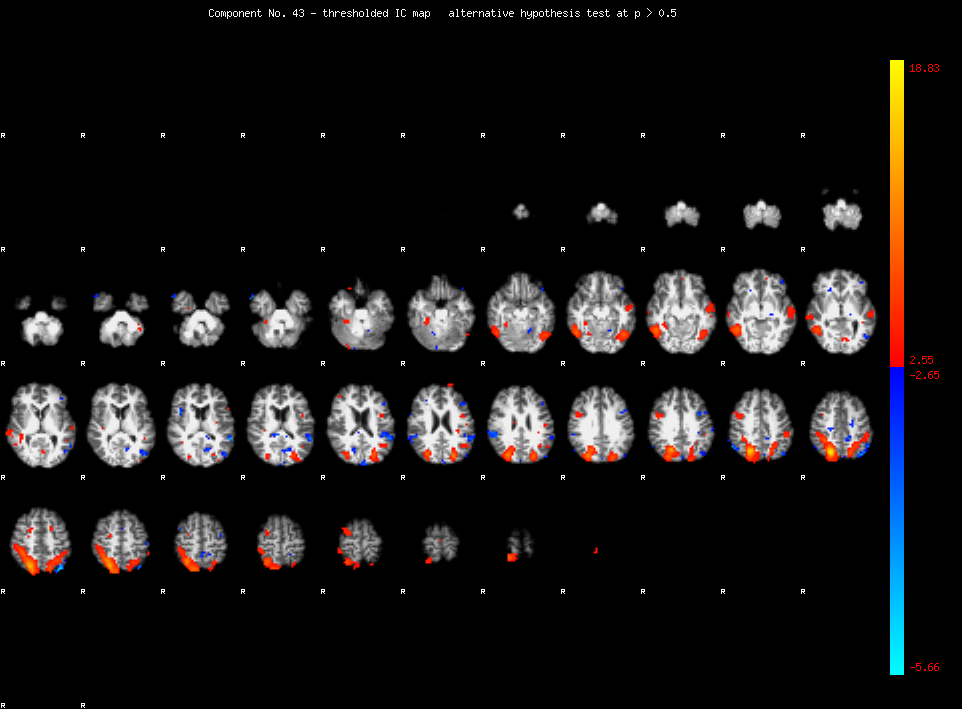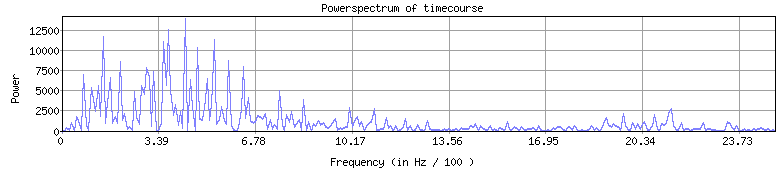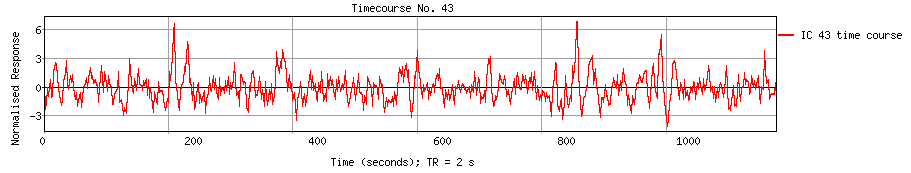 |
|  |  |
|  |  |
| 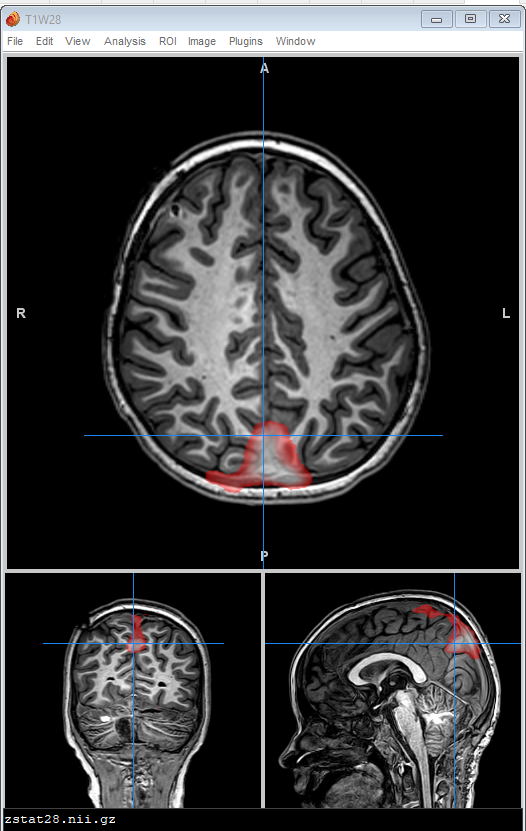  zstat 28 | 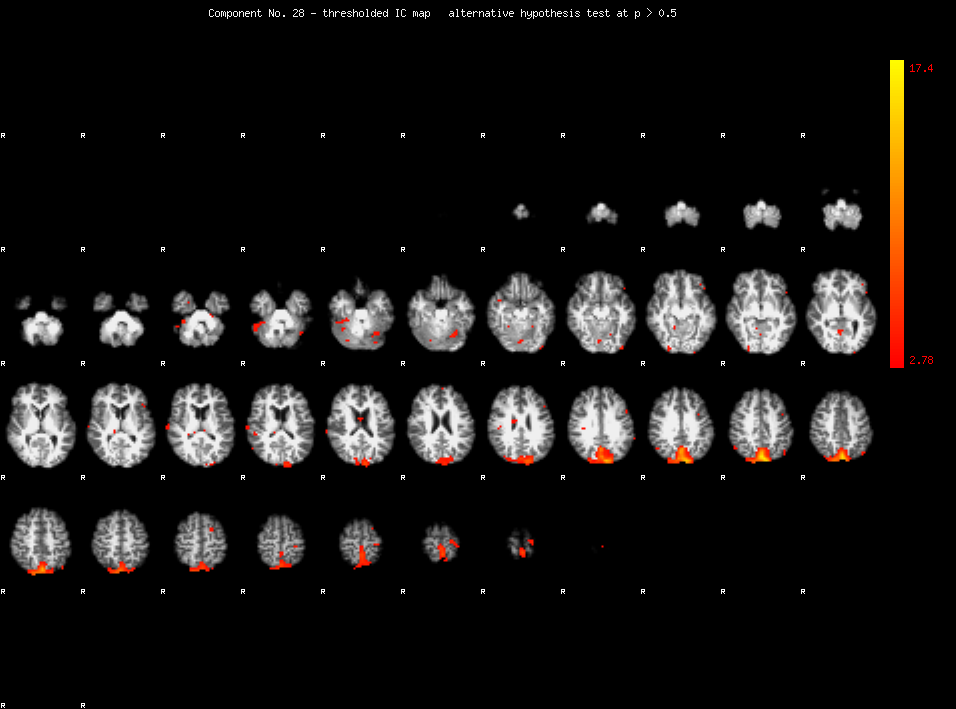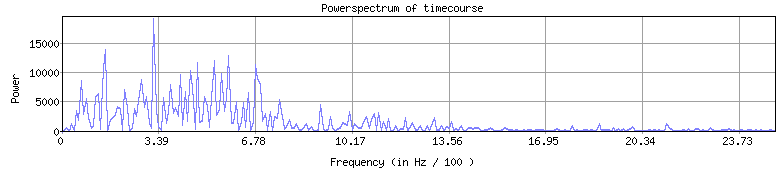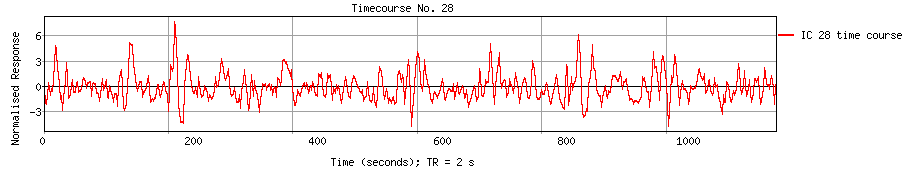 |
|  |  |

Frontal

| 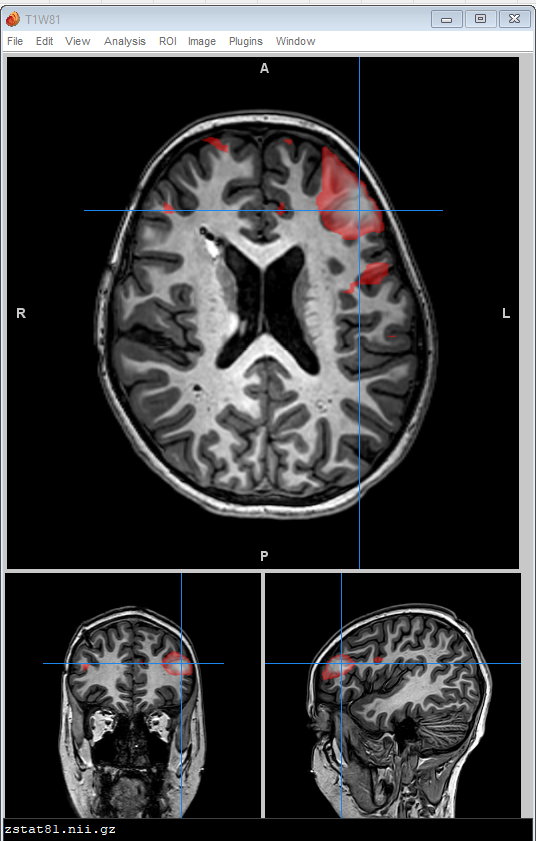  zstat 81 | 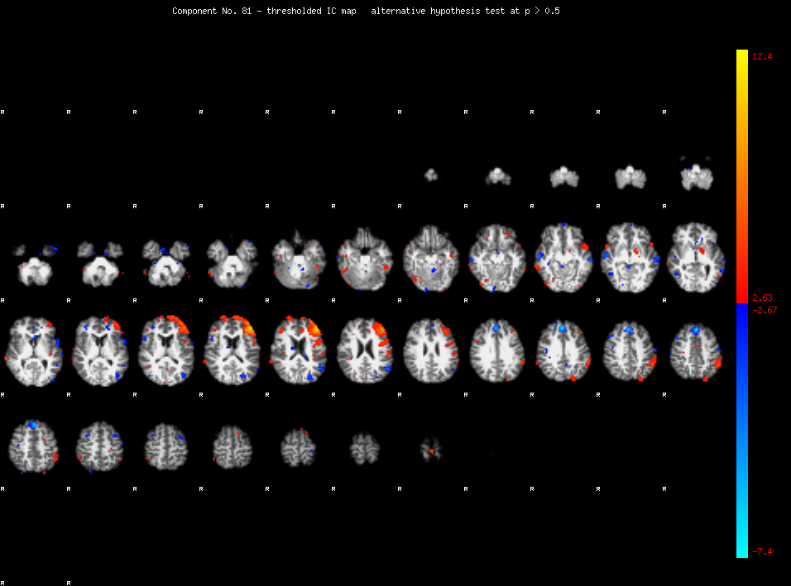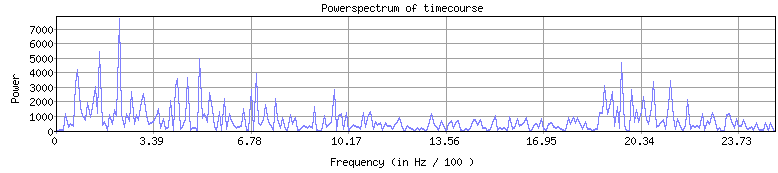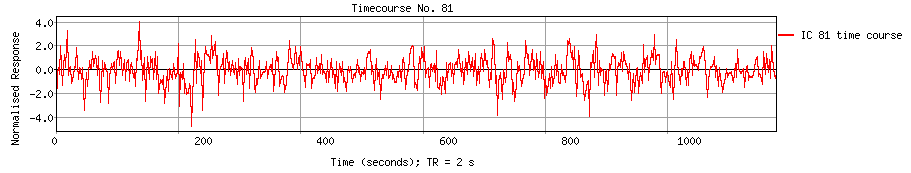 |
| --- | --- |
| 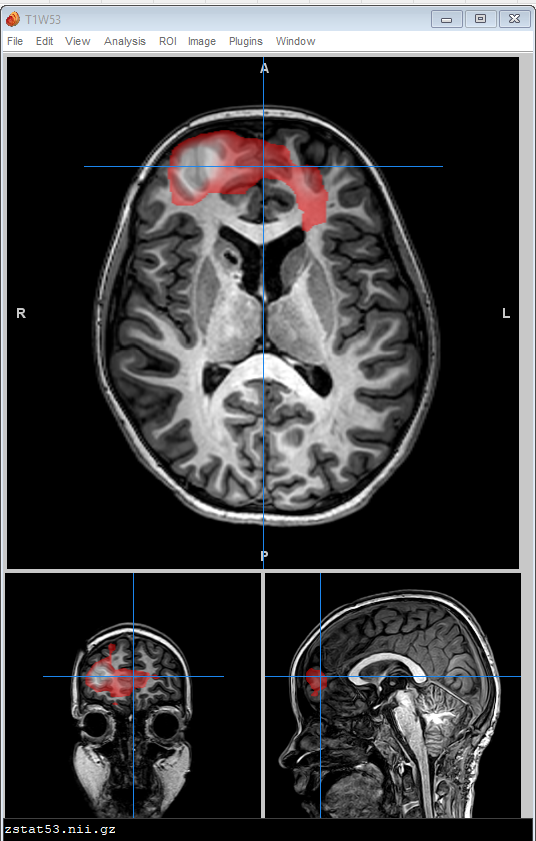  zstat 53 | 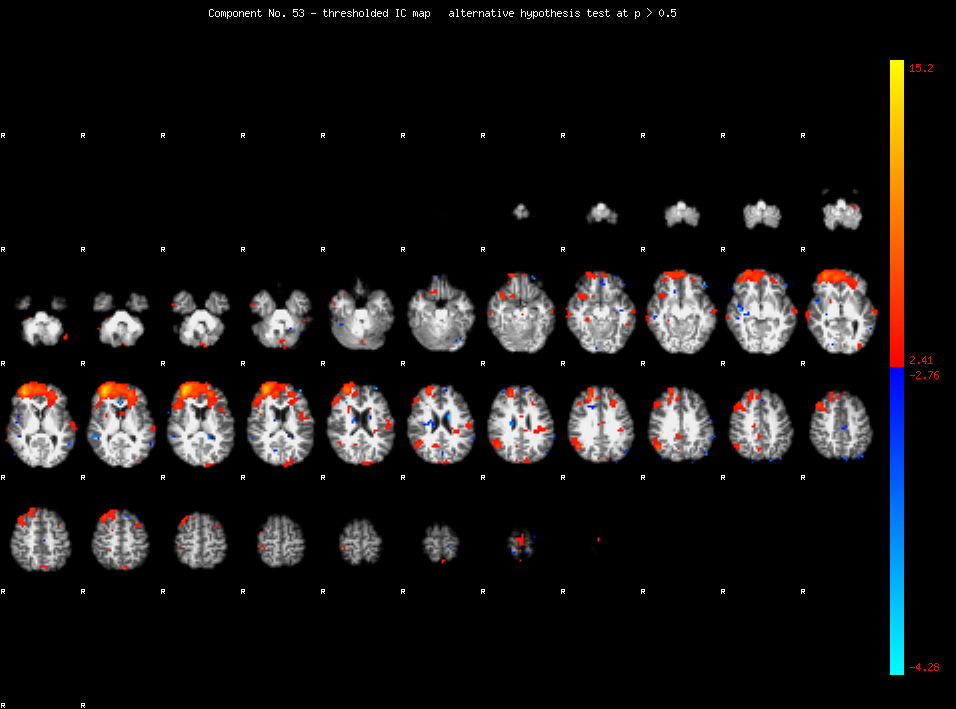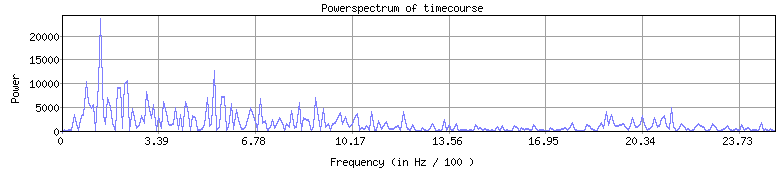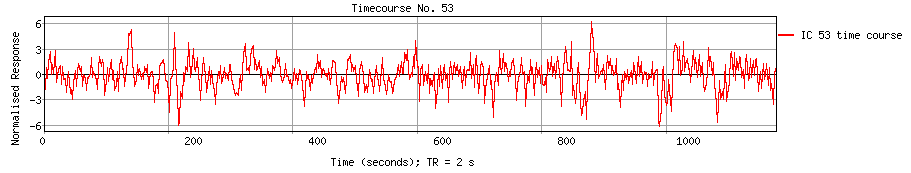 |
| zstat 50 |  |
| zstat 39 |  |
| zstat 20 |  |
| zstat 16 |  |
|  |  |

Temporal

| zstat 69 |  |
| --- | --- |
| zstat 68 |  |
| zstat 32 |  |
|  |  |

Vision

| zstat 85 |  |
| --- | --- |
| zstat 65 |  |
| zstat 26 |  |
| zstat 14 |  |
|  |  |

Deep Grey

|  |  |
| --- | --- |
|  |  |

Modulating

| zstat 70 |  |
| --- | --- |
|  |  |

Association

| zstat 63 |  |
| --- | --- |
| zstat 27 |  |
|  |  |

Other

|  |  |
| --- | --- |
|  |  |

**Abbreviation Guide**

ACG Anterior Cingulate Gyrus

APF Anterior PreFrontal

aT anterior Temporal

B Bilateral

BG Basal Ganglia

DMN Default Mode Network

IFG Inferior Frontal Gyrus

IFS Inferior Frontal Sulcus

IPL Inferior Parietal Lobule

ITG Inferior Temporal Gyrus

L Left

LR-FTP Long Range Fronto-to-Parietal association network, anterior prefrontal IFG, MFG, posterior lateral parietal S2

LS2 – lateral Secondary Sensory area

MFG Middle Frontal Gyrus

mT mesial Temporal

MTG Middle Temporal Gyrus

mS2 – medial Secondary Sensory area

Opr- Operculum

OTG Occipito-temporal gyrus

PCG Posterior Cingulate Gyrus

PFC Prefrontal Cortex

PMC Premotor Cortex

PHG Parahippocampal Gyrus

POS Parietal Occipital Sulcus

R Right

S1 Primary somatosensory cortex

S2 Secondary somatosensory cortex or secondary sensory association area

SFG Superior Frontal Gyrus

SFS Superior Frontal Sulcus

SMA Supplementary Motor Association area

SMG Supramarginal Gyrus

SPL Superior Parietal Lobule

STG Superior Temporal Gyrus

STS Superior Temporal Sulcus

TOJ – Temporal Occipital Junction network

V1 Primary visual cortex associated network

V2 Secondary visual cortex associated network

vmPFC

_<Slices>_
