# Supplementary material for: Pre- and post-therapy functional MRI connectivity in severe acute brain injury with suppression of consciousness: a comparative analysis to epilepsy features
Source: Front Neuroimaging. 2024 Oct 1;3:1445952. doi: 10.3389/fnimg.2024.1445952 (PMC11473429; doi:10.3389/fnimg.2024.1445952)
Supplement: Supplementary file 7 [file Table_7.docx]

==

Resting State functional MRI Whole Brain Map

| **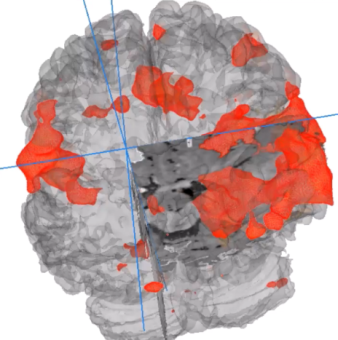** |  |  |
| --- | --- | --- |

PP5 MRI#1

Anatomical MRI - see separate radiological report,

Technique and analysis Methods 3T MRI, whole brain BOLD ICA

Data Quality Analysis 20 min; No interfering artifacts detected

Abbreviation index list at end of rs-report

Rest 1 mean displacement: absolute=0.06mm, relative=0.02mm

Rest 2 mean displacement: absolute=0.05mm, relative=0.02mm

Impression:

| 1. **Rs-fMRI seizure onset zone**: | Bilateral mesial temporal and mesial basal frontal regions |
| --- | --- |
| 1. **Language**: | detected |
| 1. **Whole brain networks:** | Well detected, possible covert consciousness, candidate for tb-fMRI |

| Rs-fMRI Informed Neuro-Prognostication | | |
| --- | --- | --- |
| Consciousness | Alertness and Awareness  Equivalent of 0=Coma, 1=Normal Consciousness, 2=MCS, 3=VS/UWS |  |
| Developmental Stream | 0=very poor, 1=Normal, 2=Atypical but not very poor, 3=Very poor, 4=Indeterminate | |
| Motor | Walking |  |
|  | Gross motor body movement |  |
|  | L - arm/hand fine motor | 1 |
|  | R- arm/hand fine motor |  |
|  | face/mouth motor coordination |  |
| Motor tone | R appendicular tone |  |
|  | L appendicular tone | 1 |
|  | Central tone |  |
| Language | Understanding words | 1 |
|  | Speaking in Words |  |
| Vision | R field primary visual reception |  |
|  | R field higher level visual stimuli interpretation | 1 |
|  | L field primary visual reception |  |
|  | L field higher level visual stimuli interpretation |  |
| Sensory | Touch/Sense of Spatial/complex relationship | 1 |
| Cognition/Learning/Memory | equivalent IQ >70 | 1 |
|  | equivalent IQ < 70 |  |
|  | Profound Intellectual Disability Equivalent IQ < 35 |  |

* Scoring unless otherwise specified is shown as the possible highest score expected or range

| Network Characterization | | | | |
| --- | --- | --- | --- | --- |
| 1=yes, 0=no | Detected | Normal | Atypical | Comment |
| Motor | 1 | 1 |  |  |
| Language | 1 | 1 |  | left |
| Parietal | 1 | 1 |  |  |
| Frontal | 1 | 1 |  |  |
| Temporal | 1 | 1 |  |  |
| Vision | 1 | 1 |  |  |
| Deep grey | 1 | 1 |  |  |
| Modulating | 1 | 1 |  |  |
| Association | 1 | 1 |  |  |
| Atypical/possible pathological | Summary SOZ: 6 temporal/basal frontal  Atypical RSN Summary:  (12) BMT and basal frontal  (17) Basal frontal an B MT  (24) B Temporal  (26) multifocal, right temporal and L OPC  (42) B temporal  (66) L temporal  Atypical RSN  (29) B mesial thalamus  (56) B BG + cortical motor regions  (72) R PfC | | | |

| **Atypical Networks** |
| --- |

| 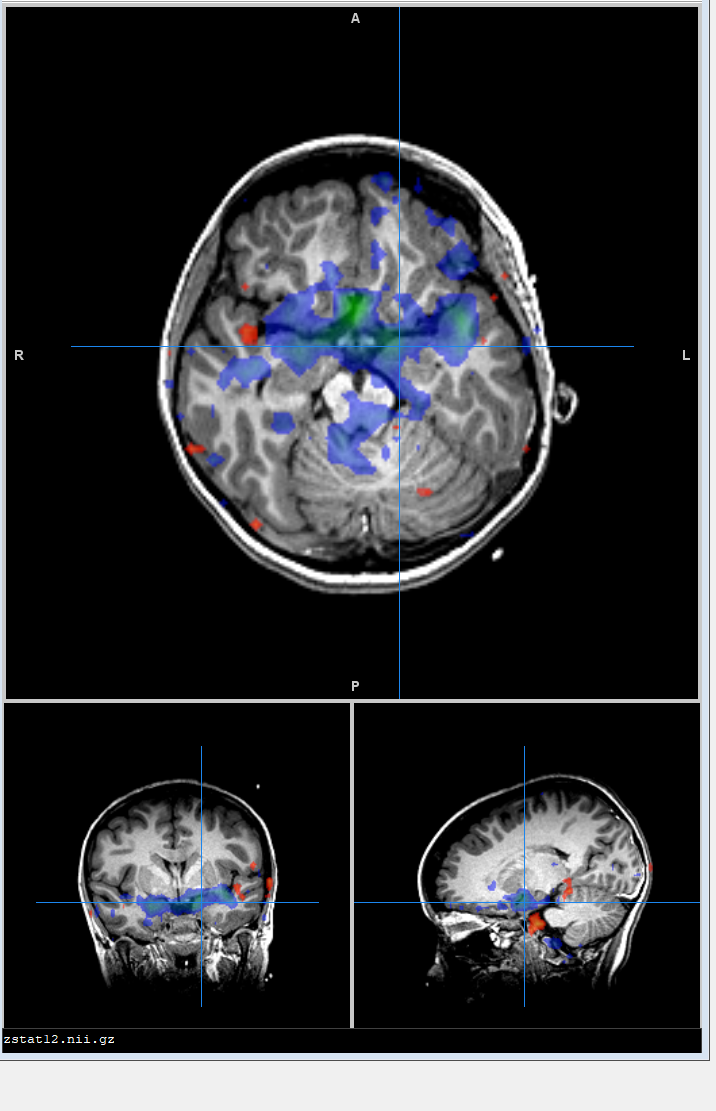 zstat12 | 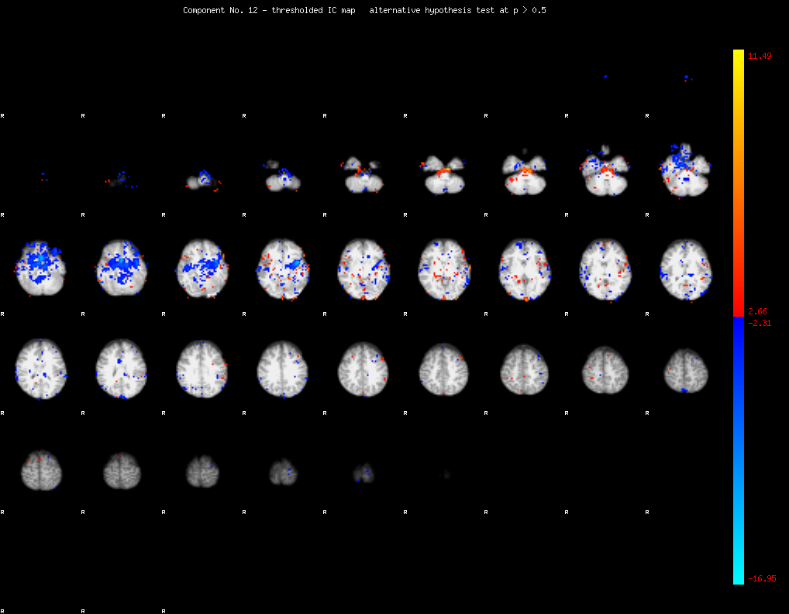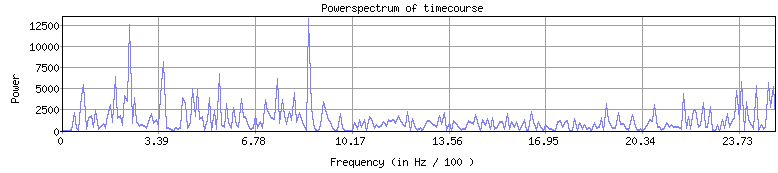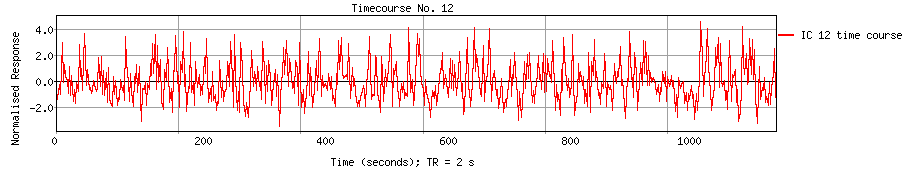 |
| --- | --- |
|  |  |
| 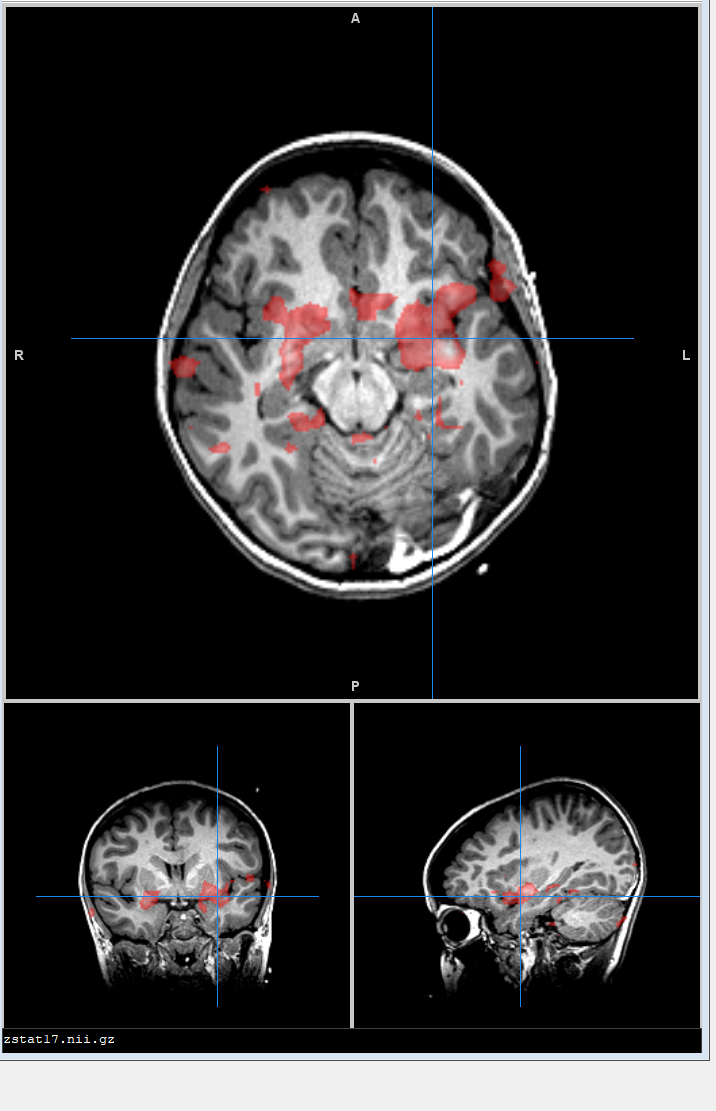 zstat17 | 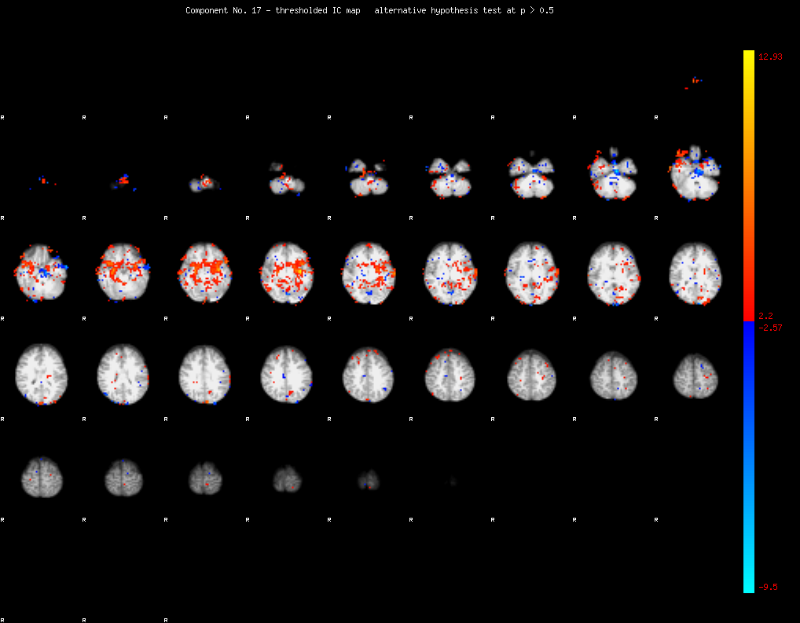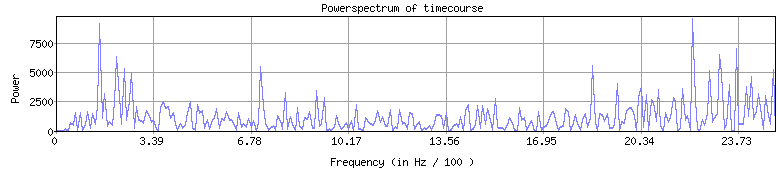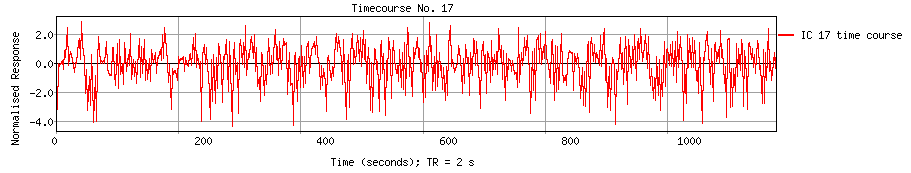 |
| 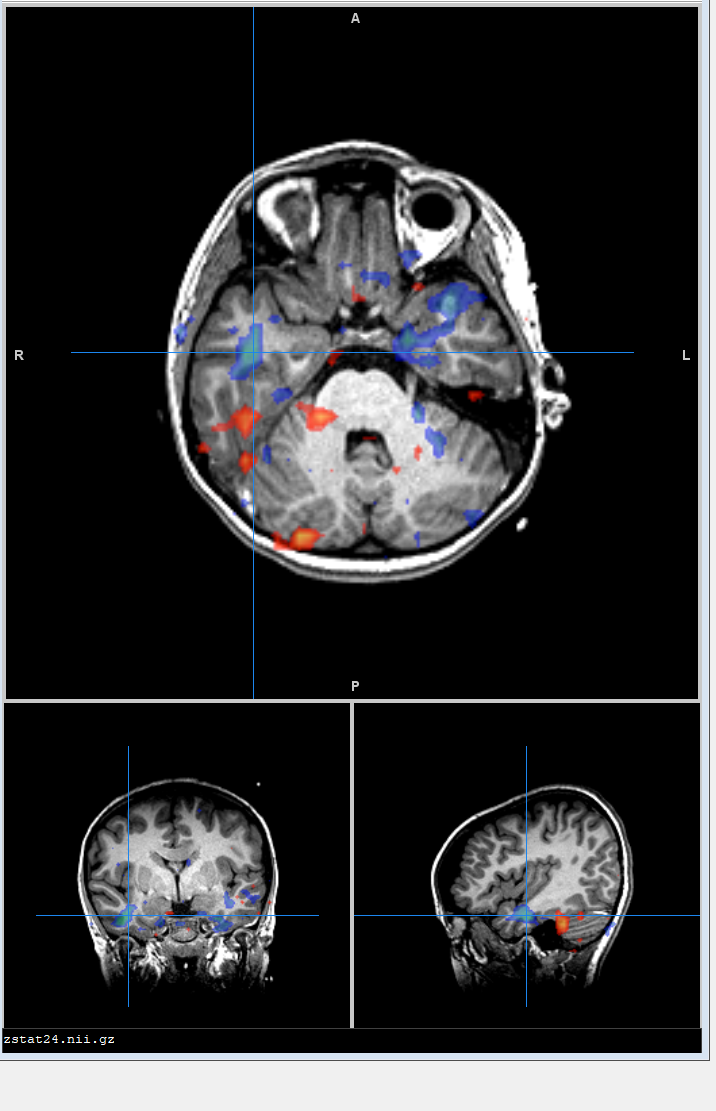 zstat24 | 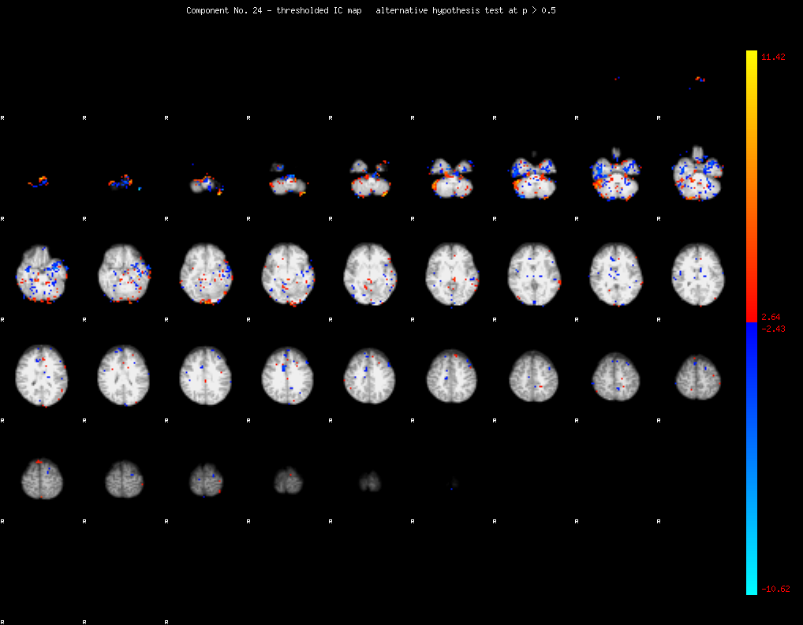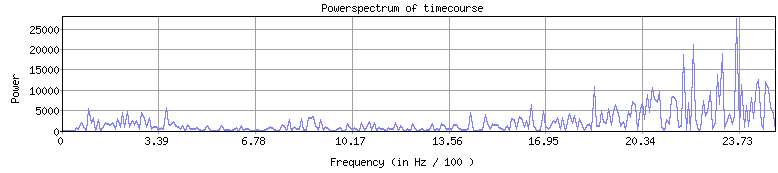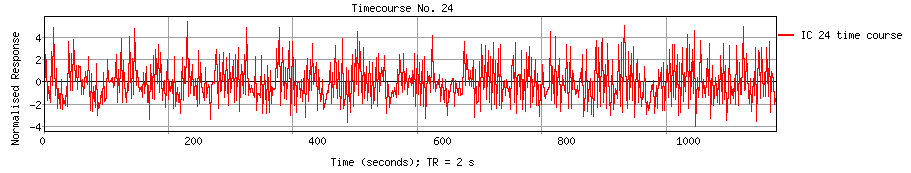 |
| 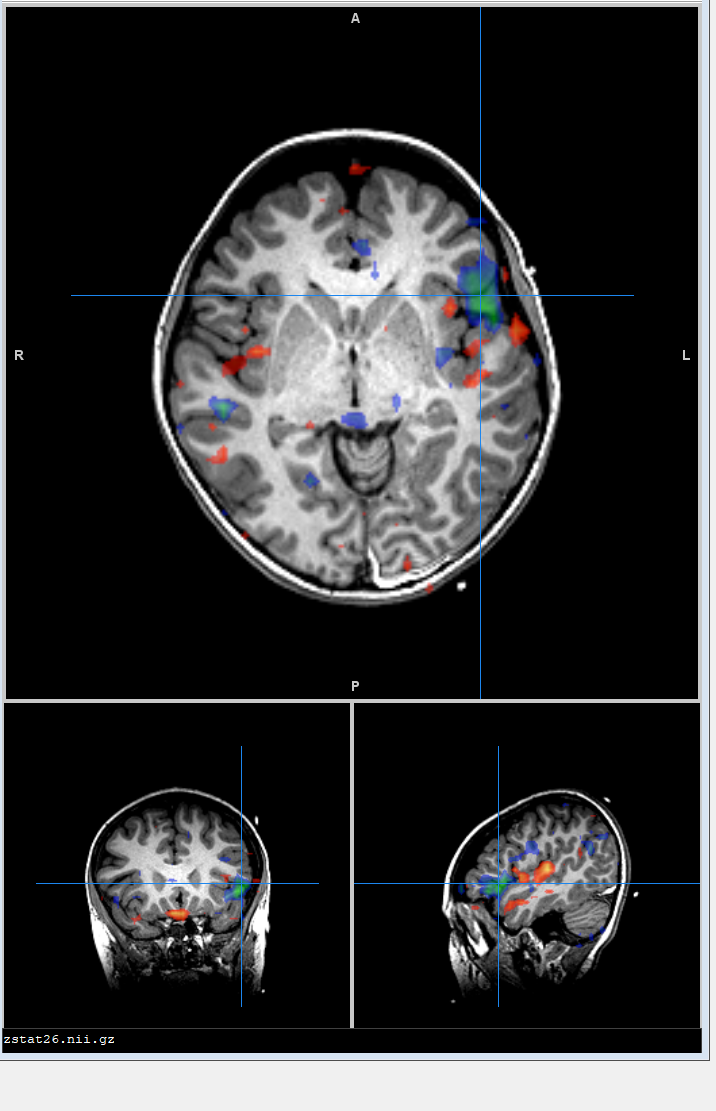 zstat26 | 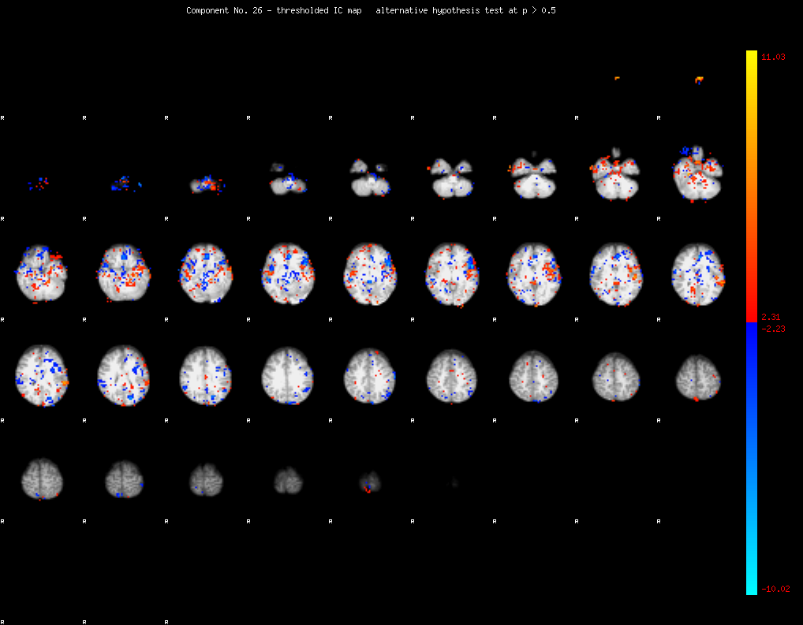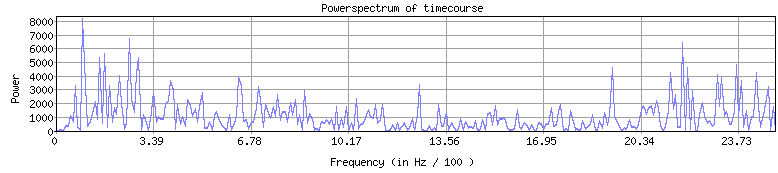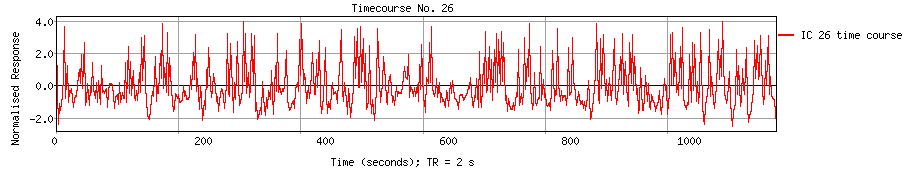 |
| 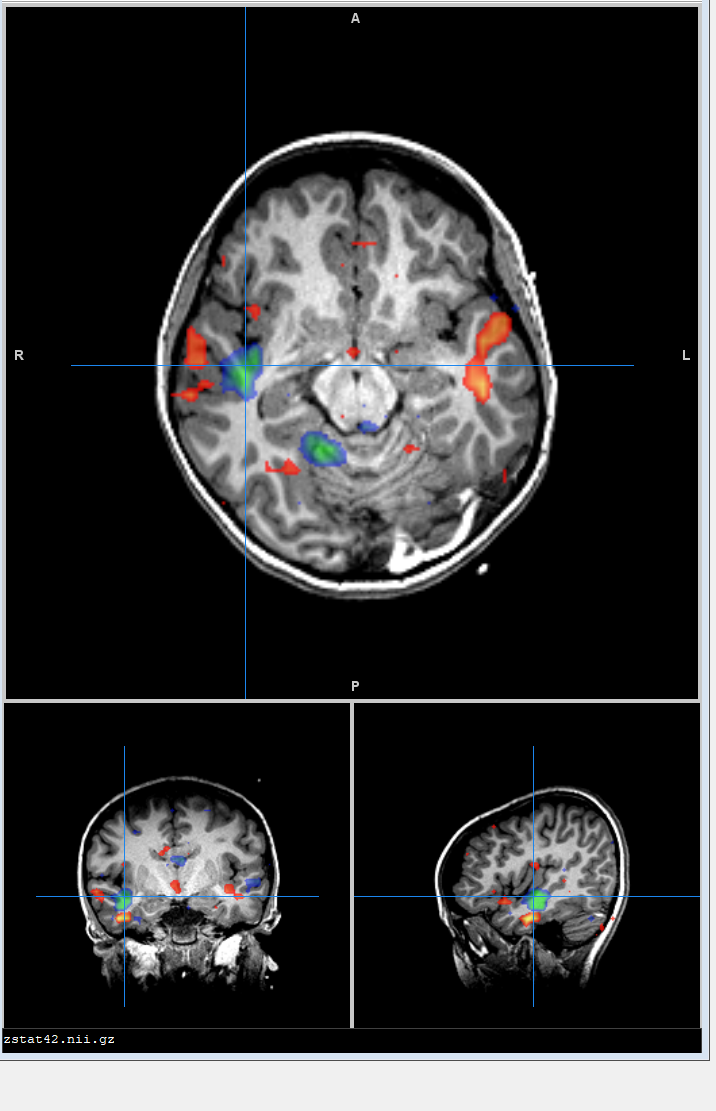 zstat42 | 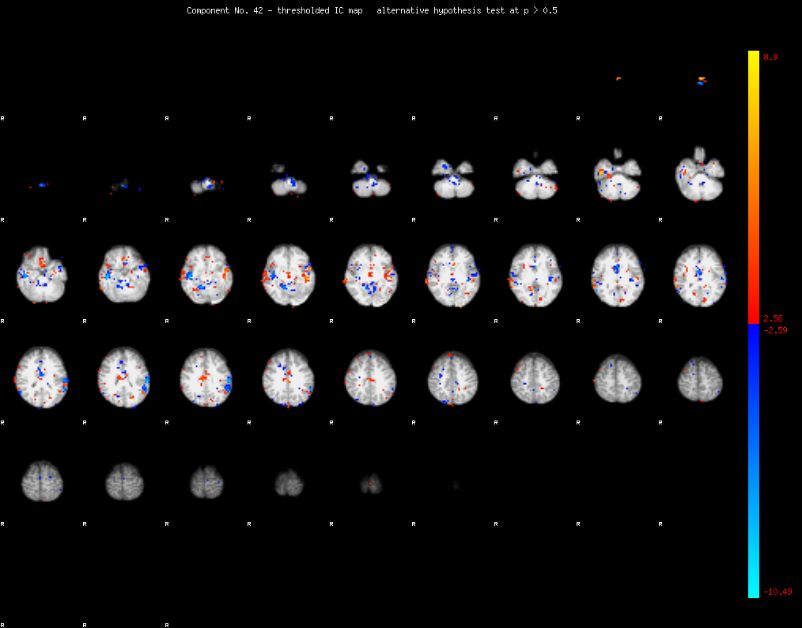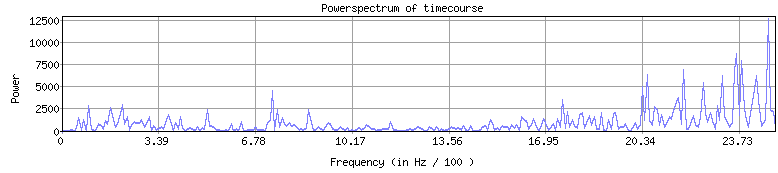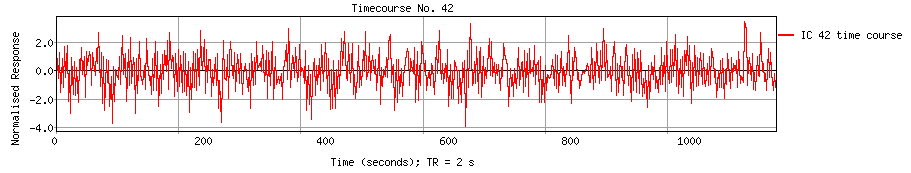 |
|  |  |
|  |  |
| 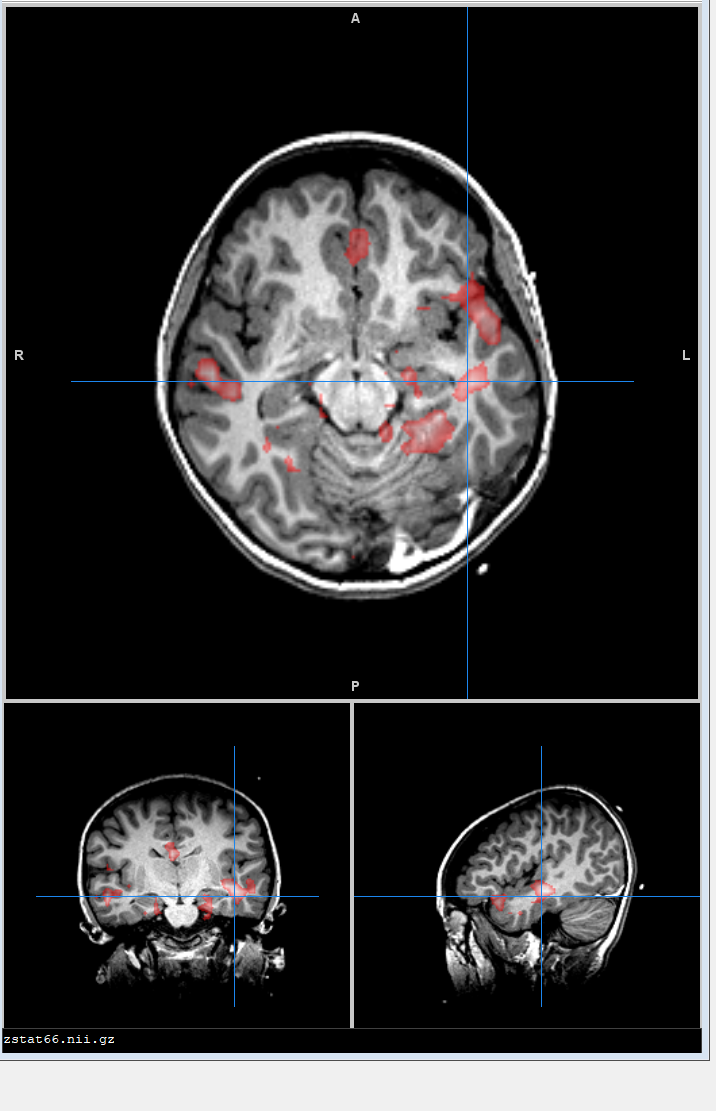 zstat66 | 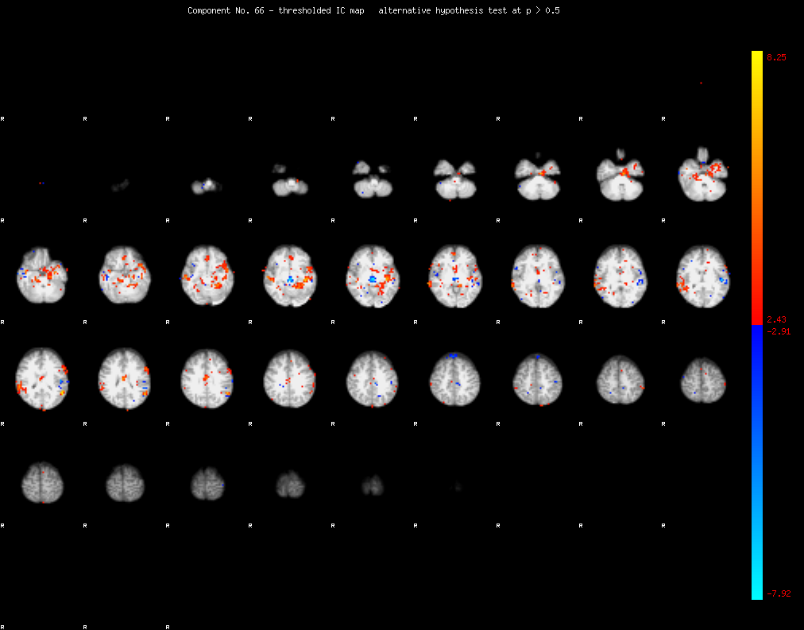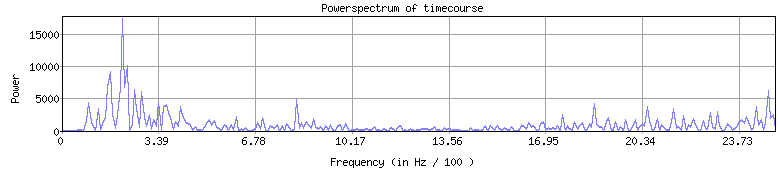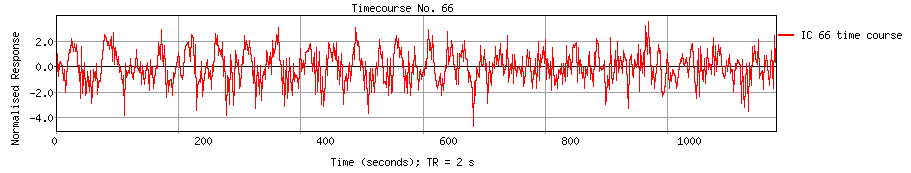 |
|  |  |

| **Atypical RSN** |
| --- |

| 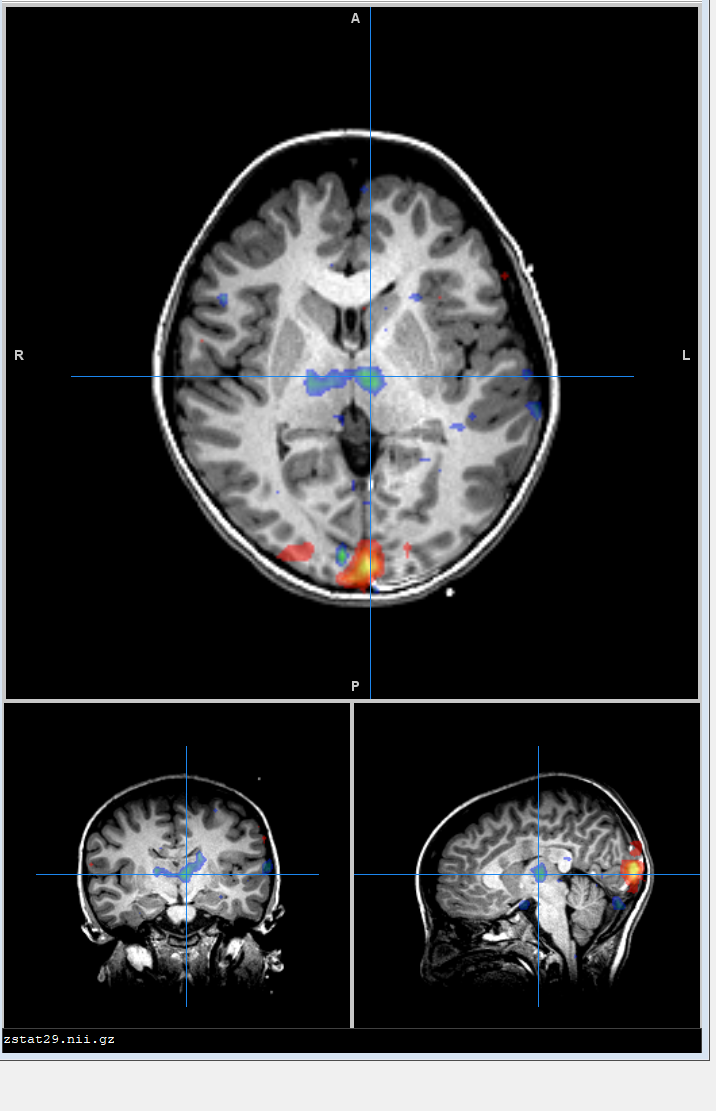 zstat29 | 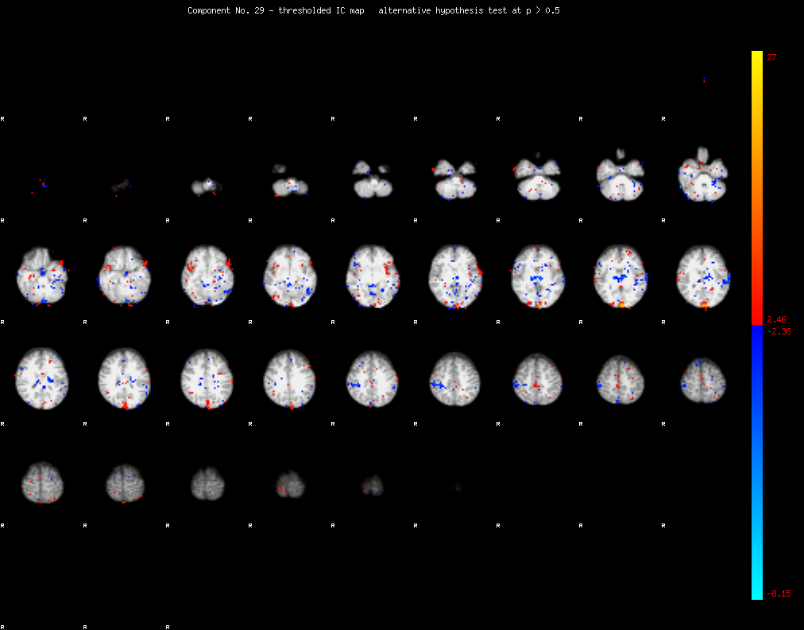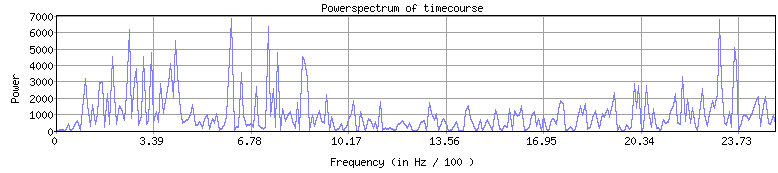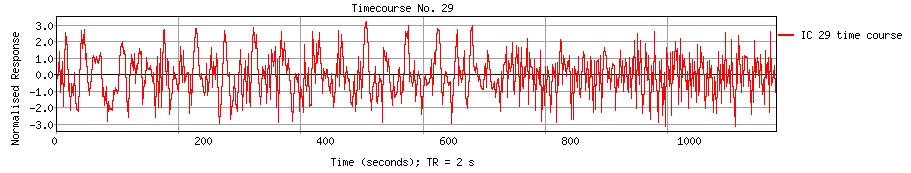 |
| --- | --- |
|  |  |
|  |  |

|  |  |
| --- | --- |
| 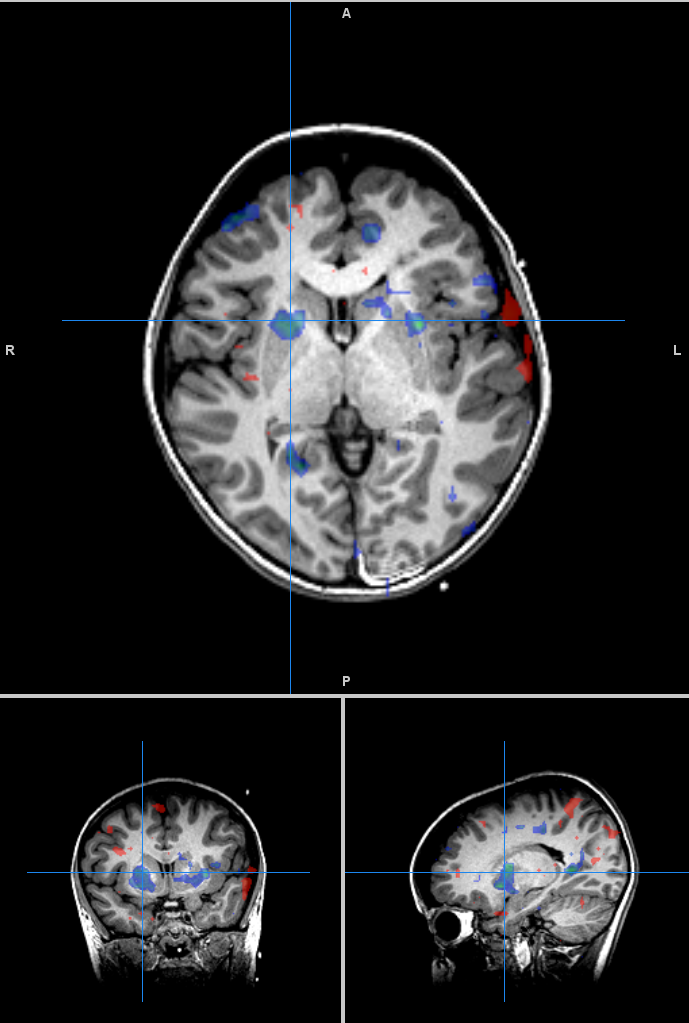 zstat56 | 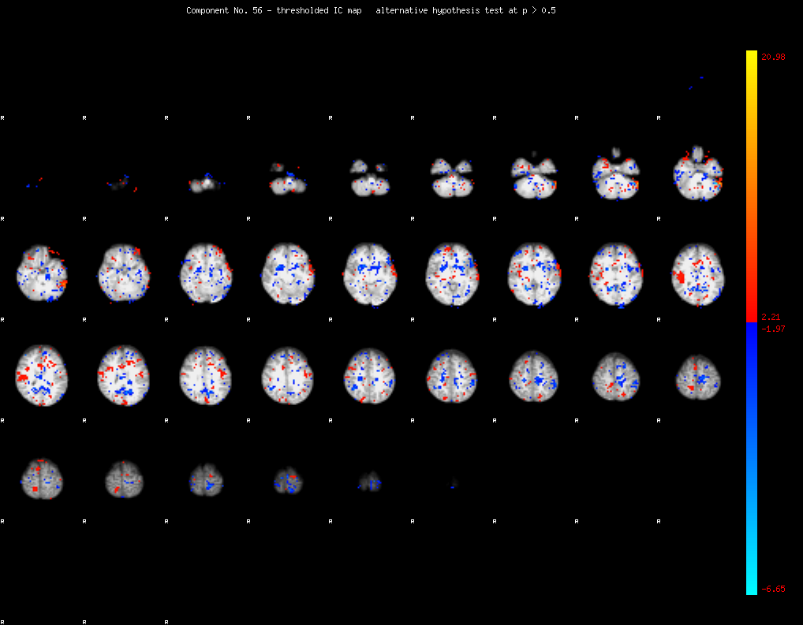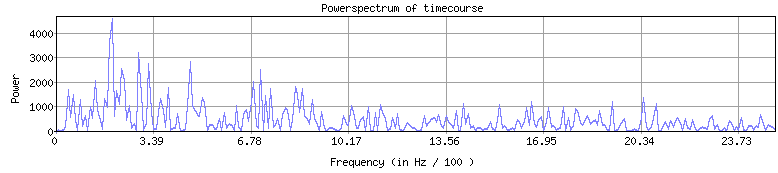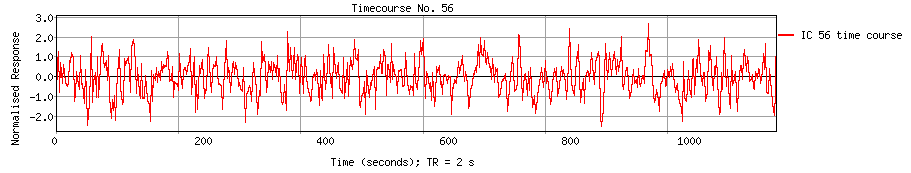 |
|  |  |
|  |  |
|  |  |
| 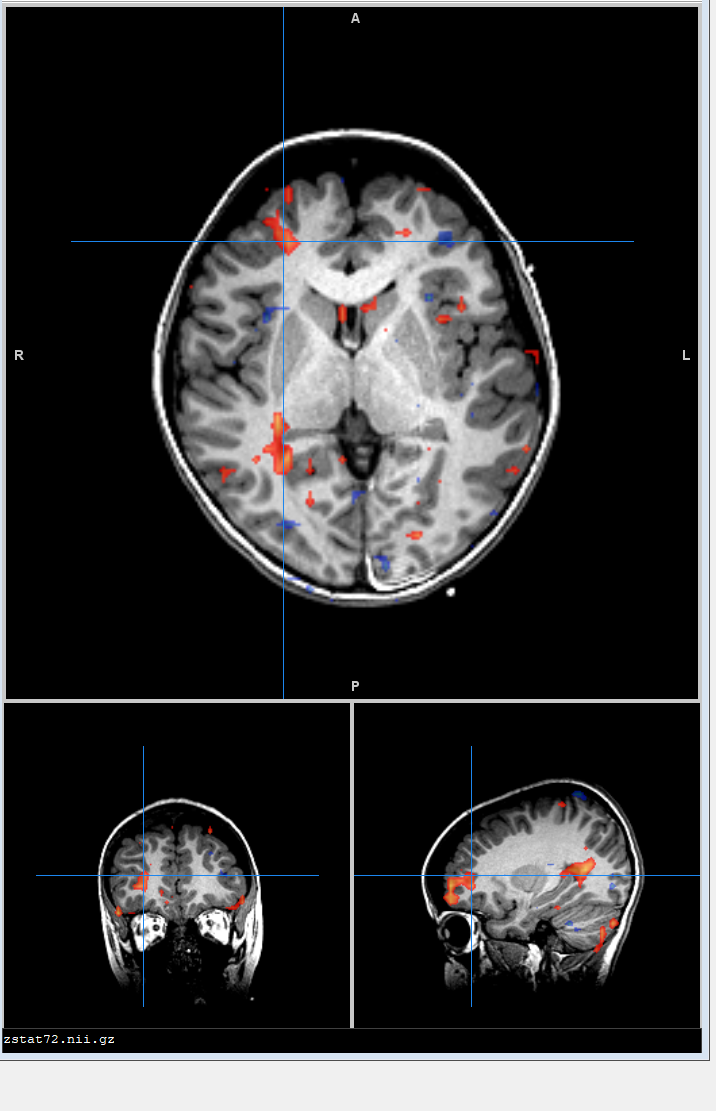 zstat72 | 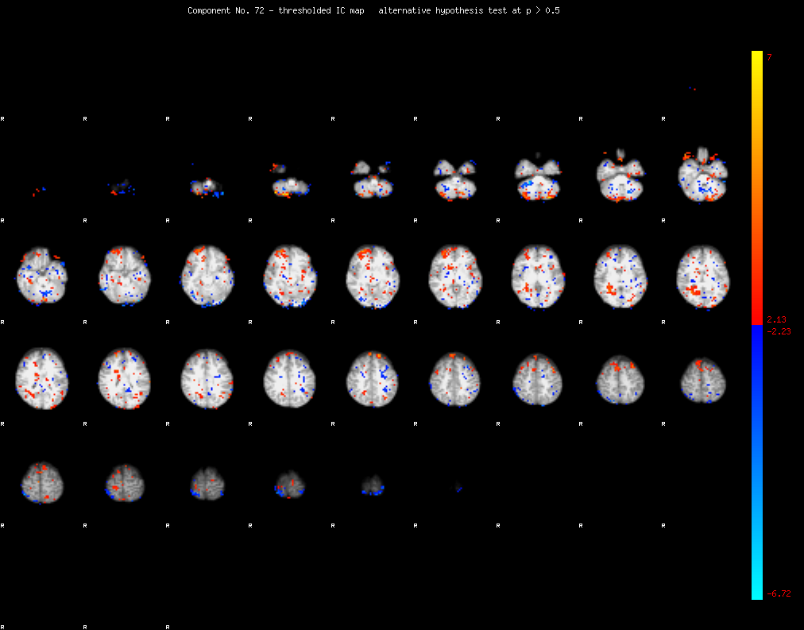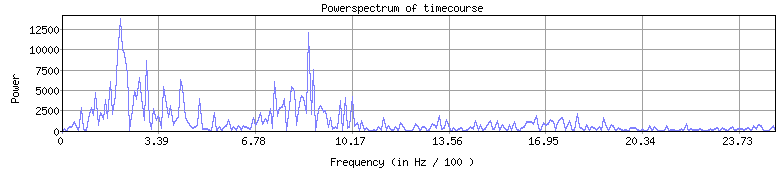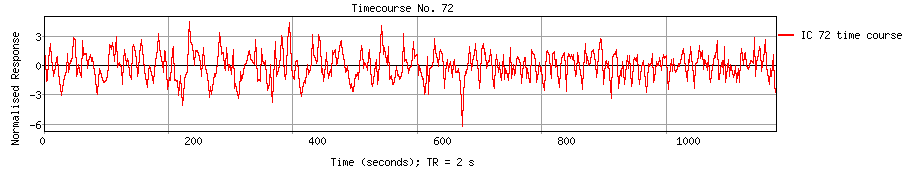 |
|  |  |
|  |  |
|  |  |
|  |  |
|  |  |

| **Motor** |
| --- |

| 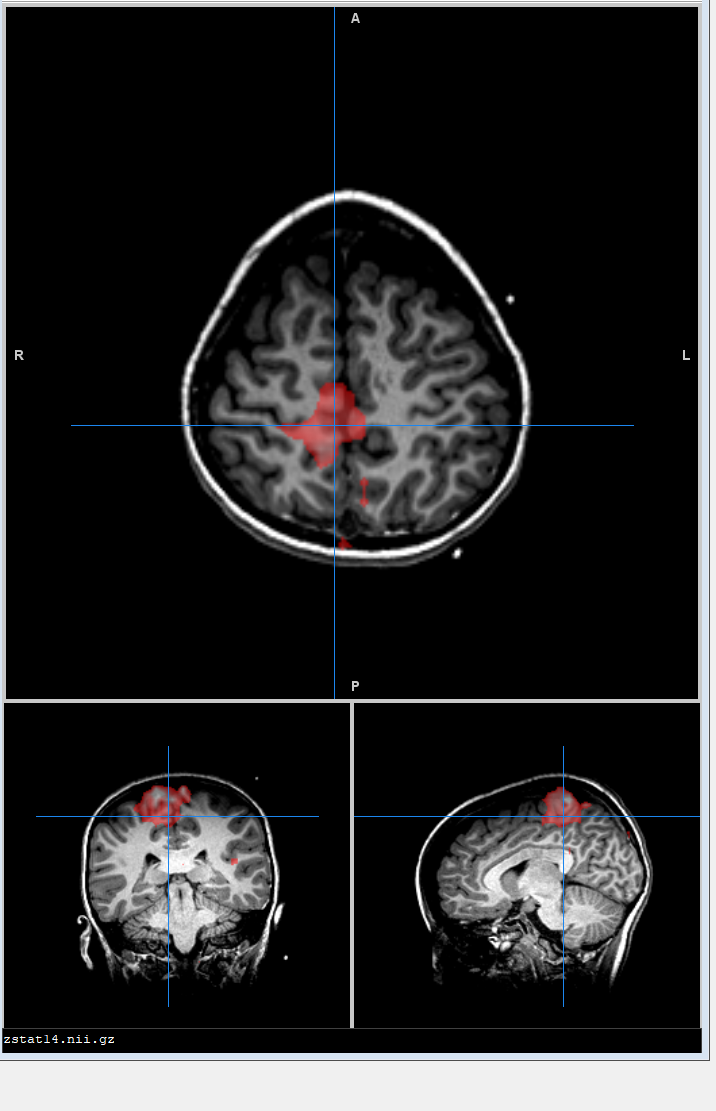 zstat14 | 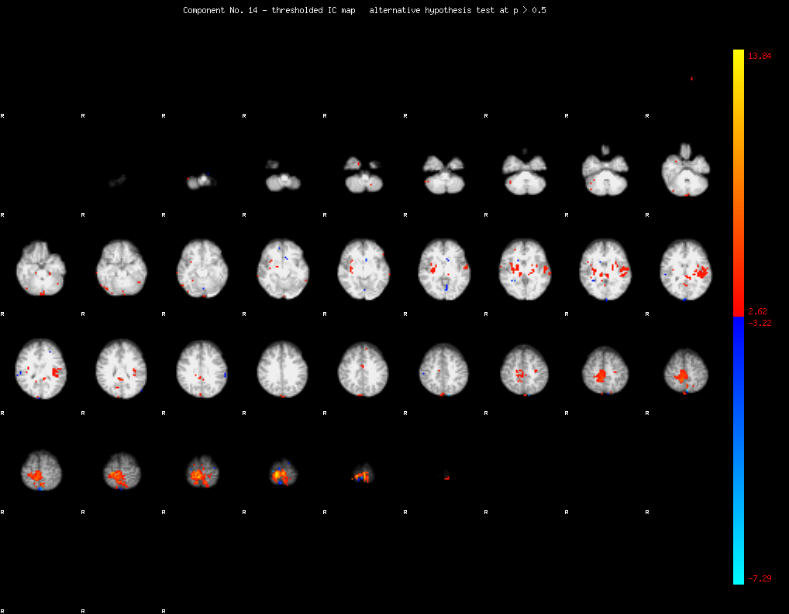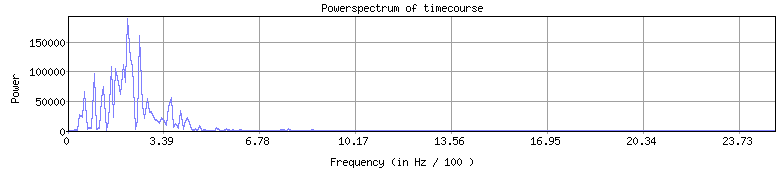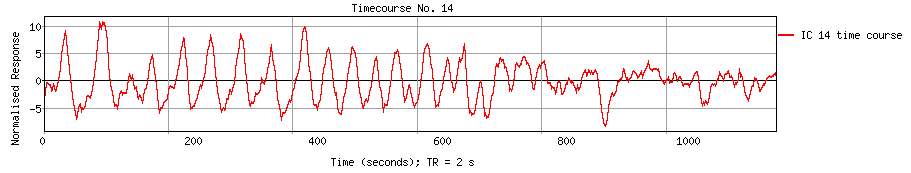 |
| --- | --- |
| 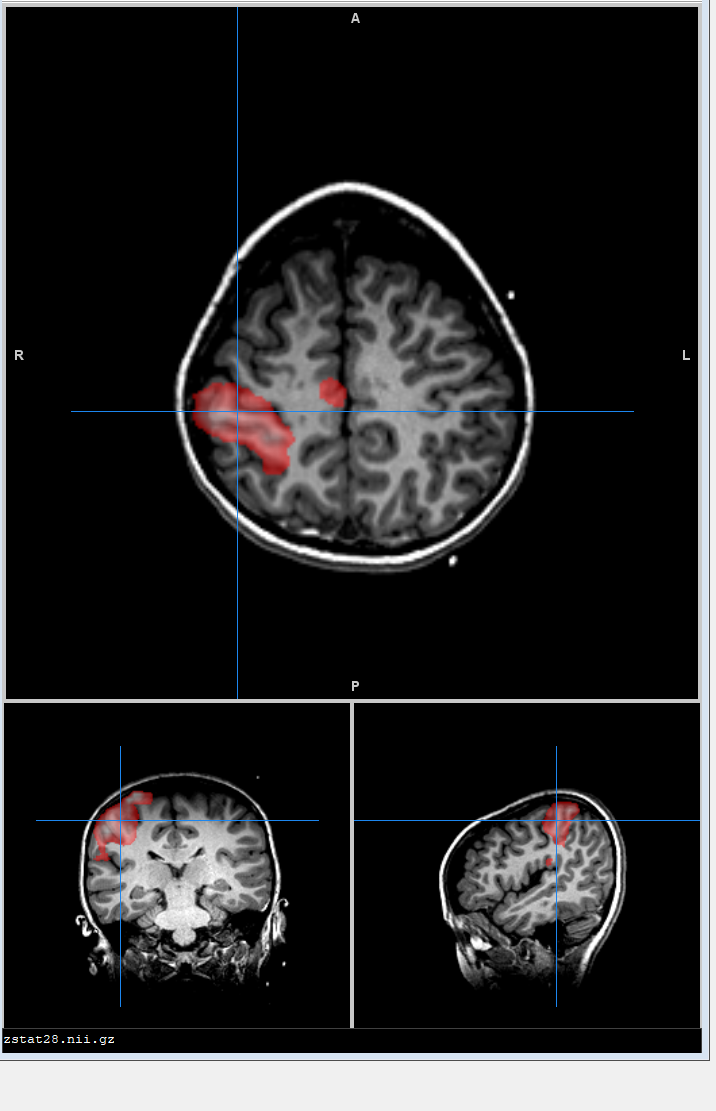 zstat28 | 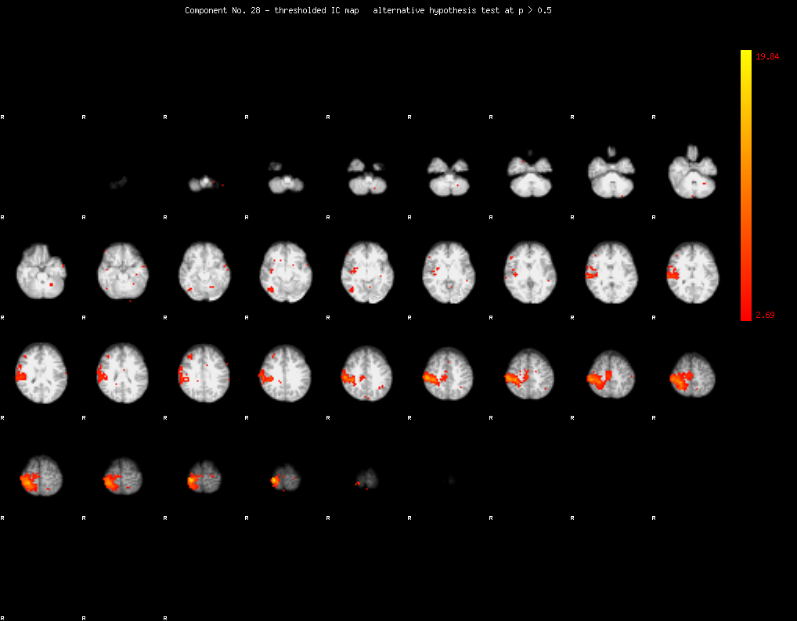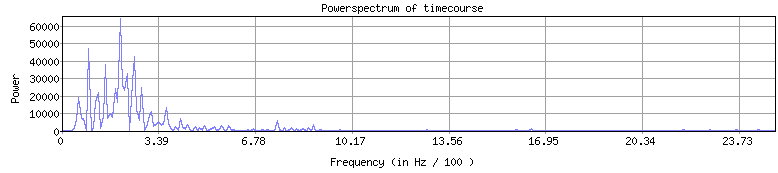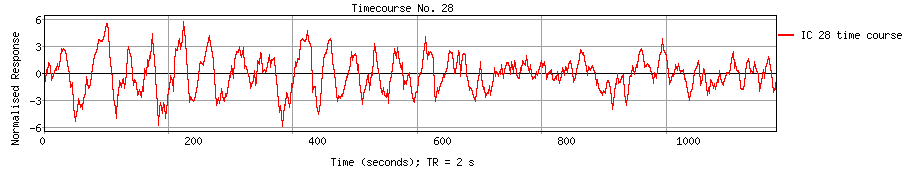 |
| 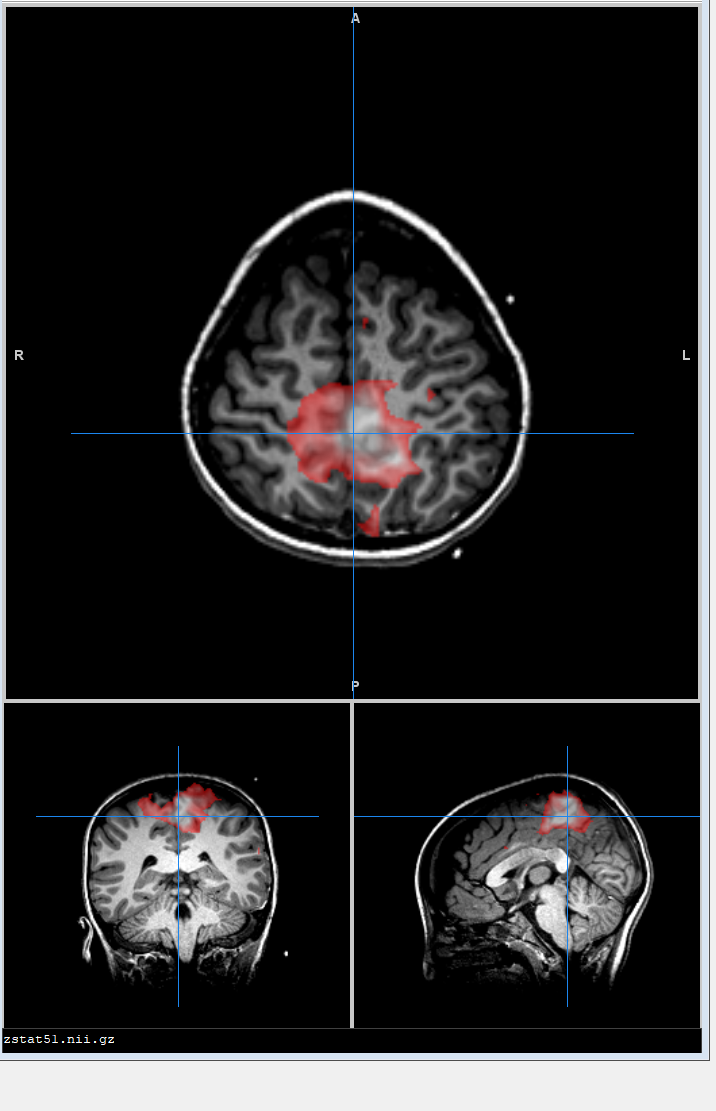 zstat51 | 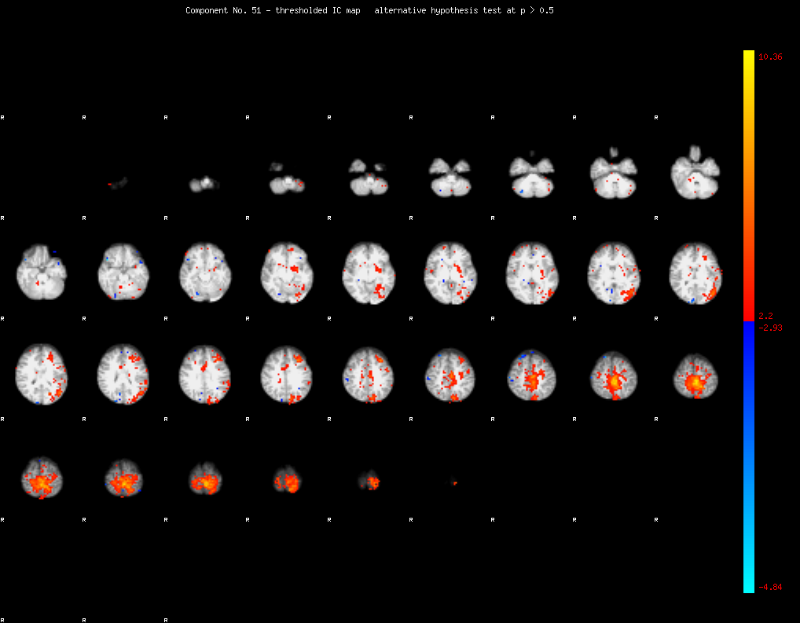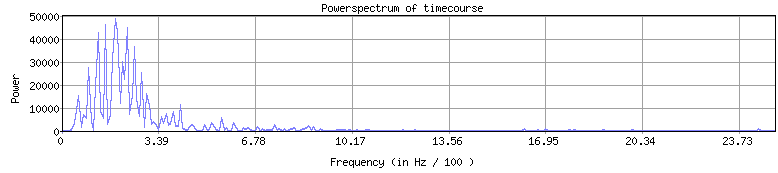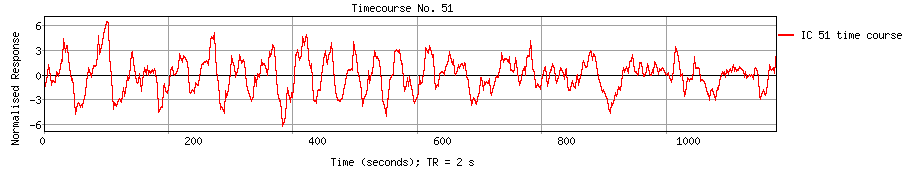 |
| 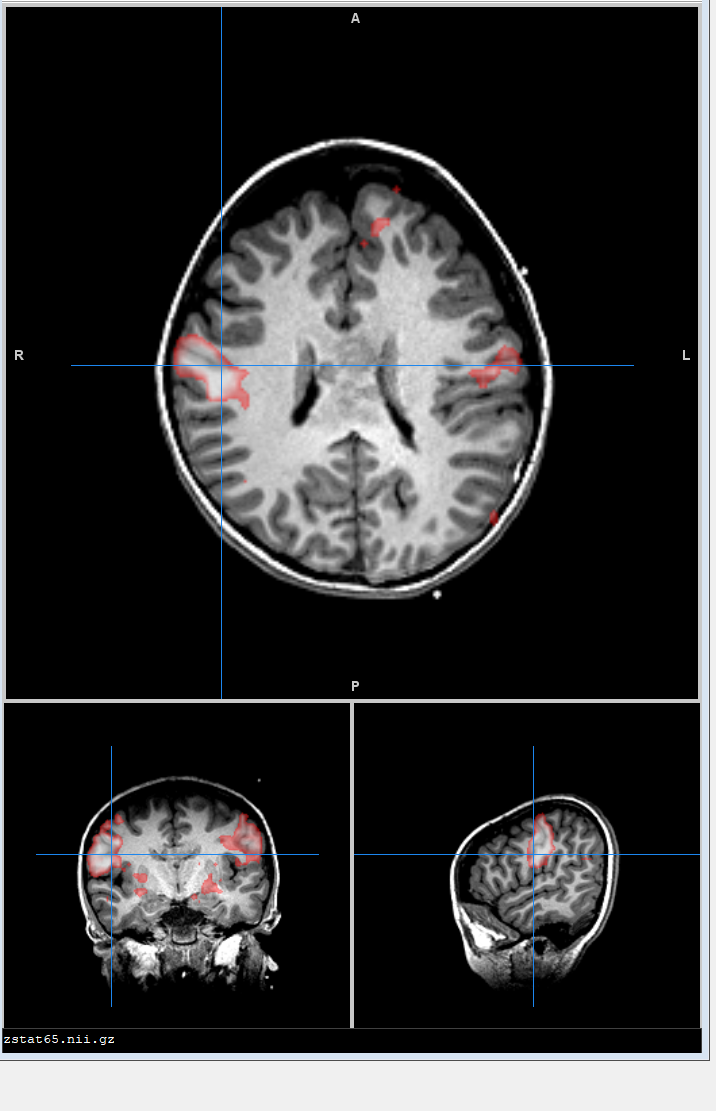 zstat65 | 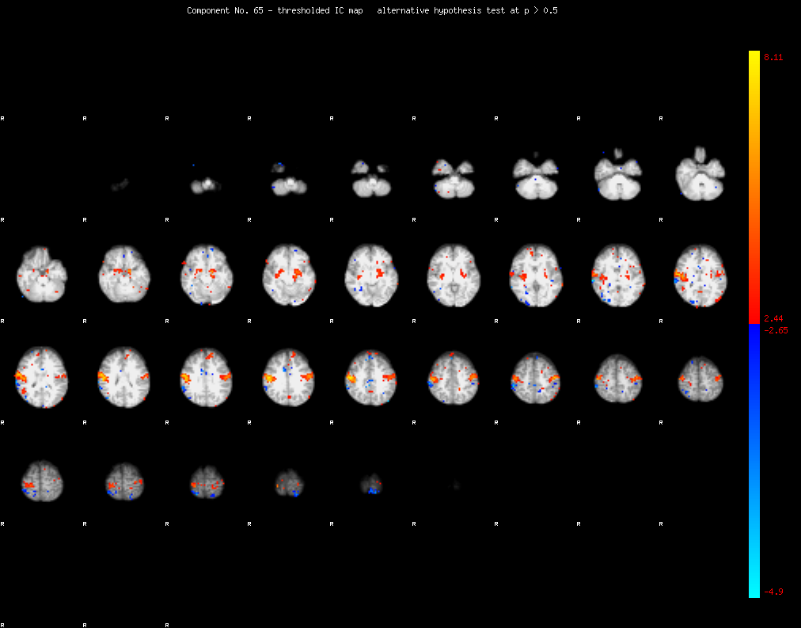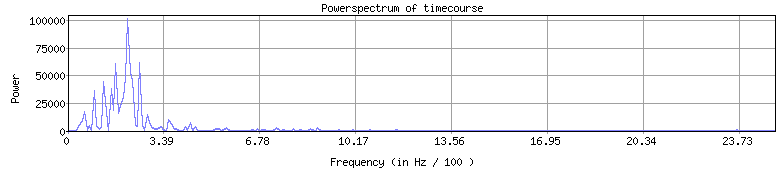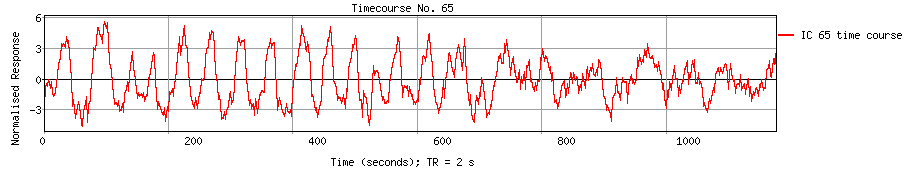 |
|  |  |

| **Language** |
| --- |

|  |  |
| --- | --- |
| Left language summary image | Right language summary image |
| 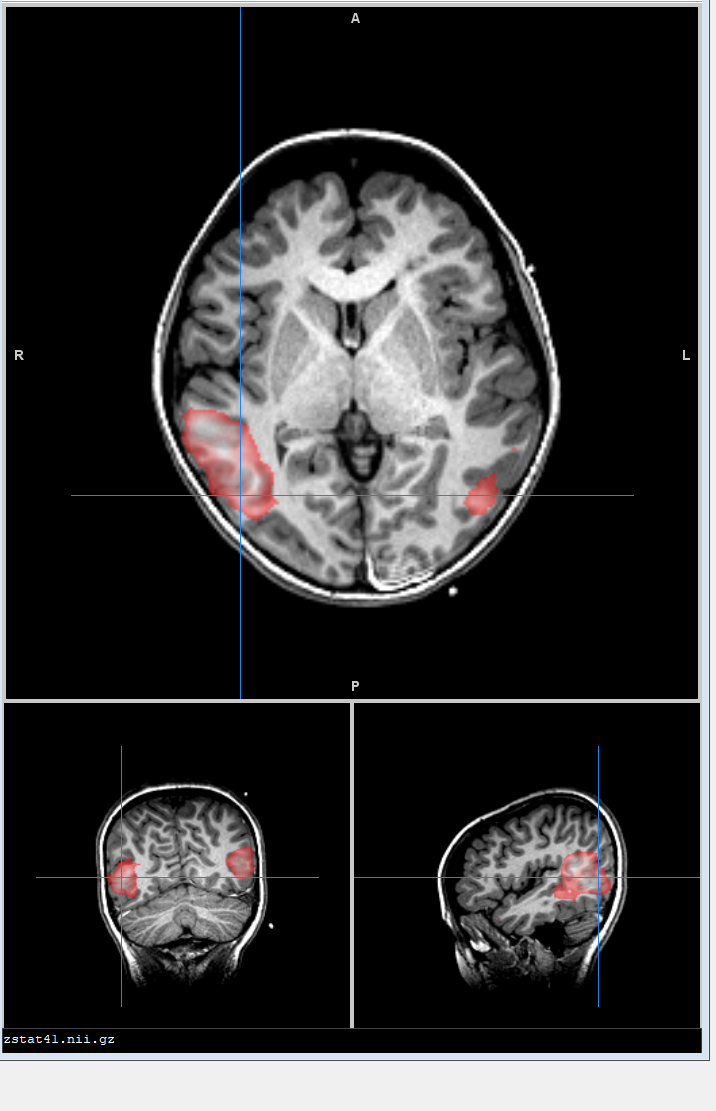 zstat41 | 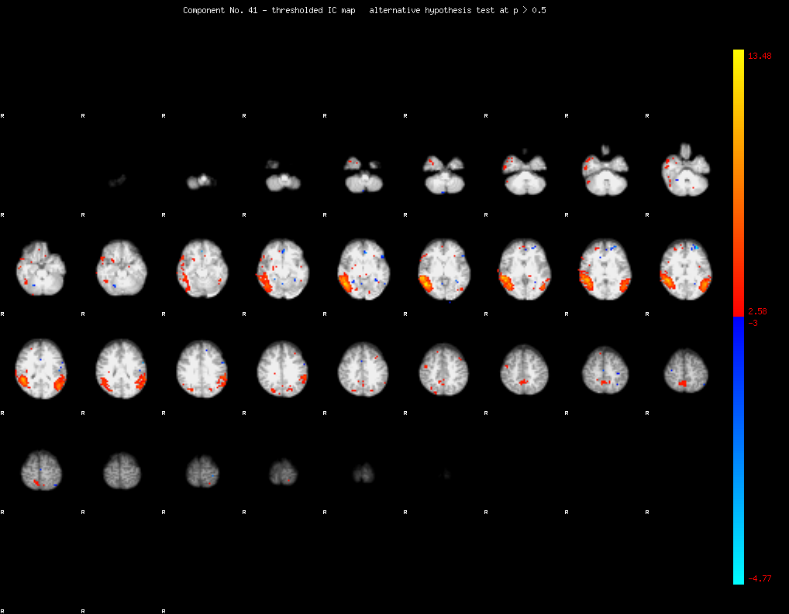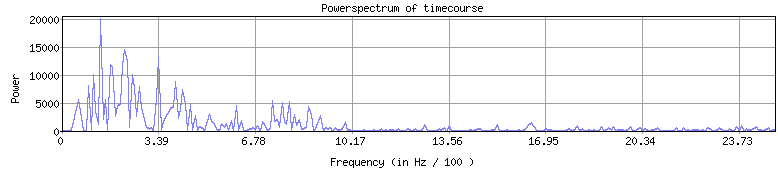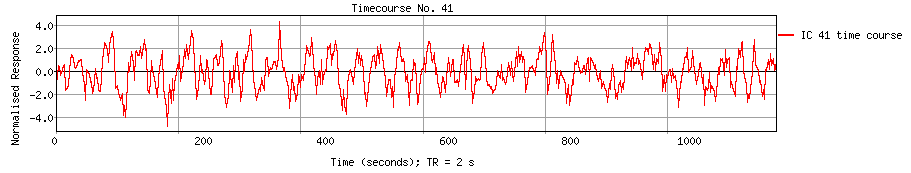 |
| 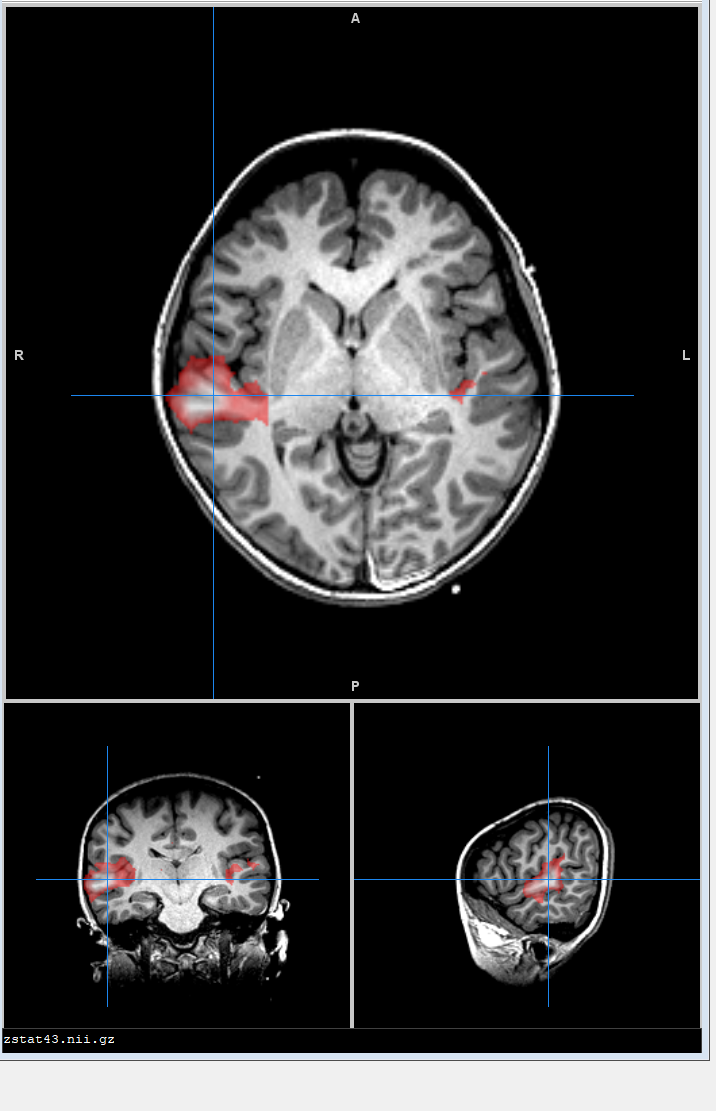 zstat43 | 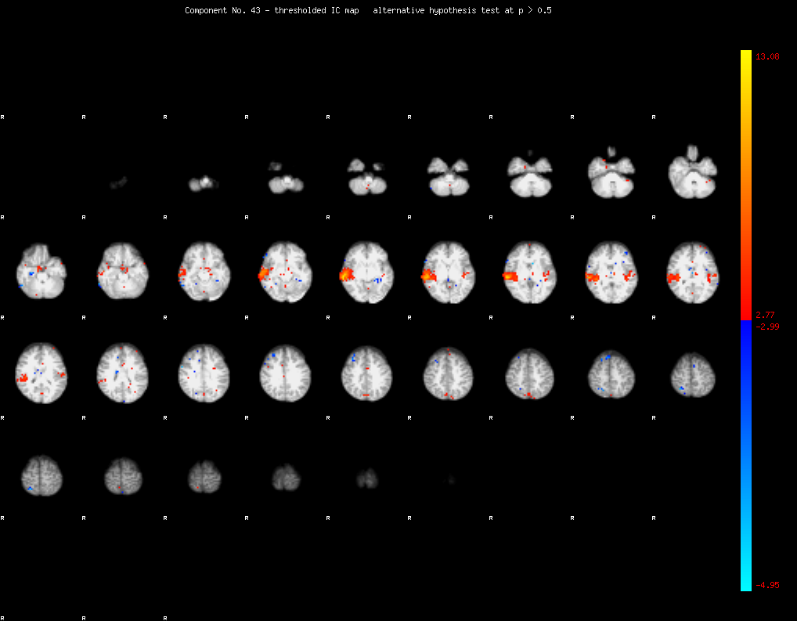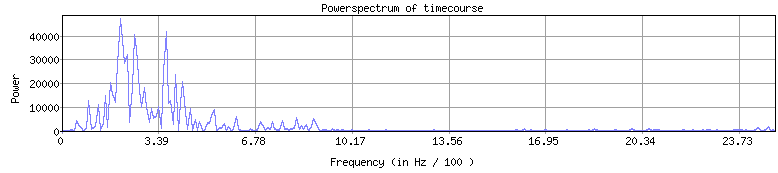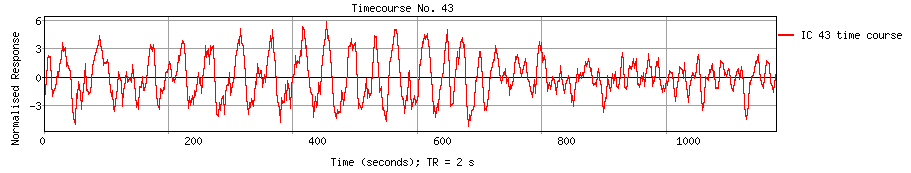 |
| 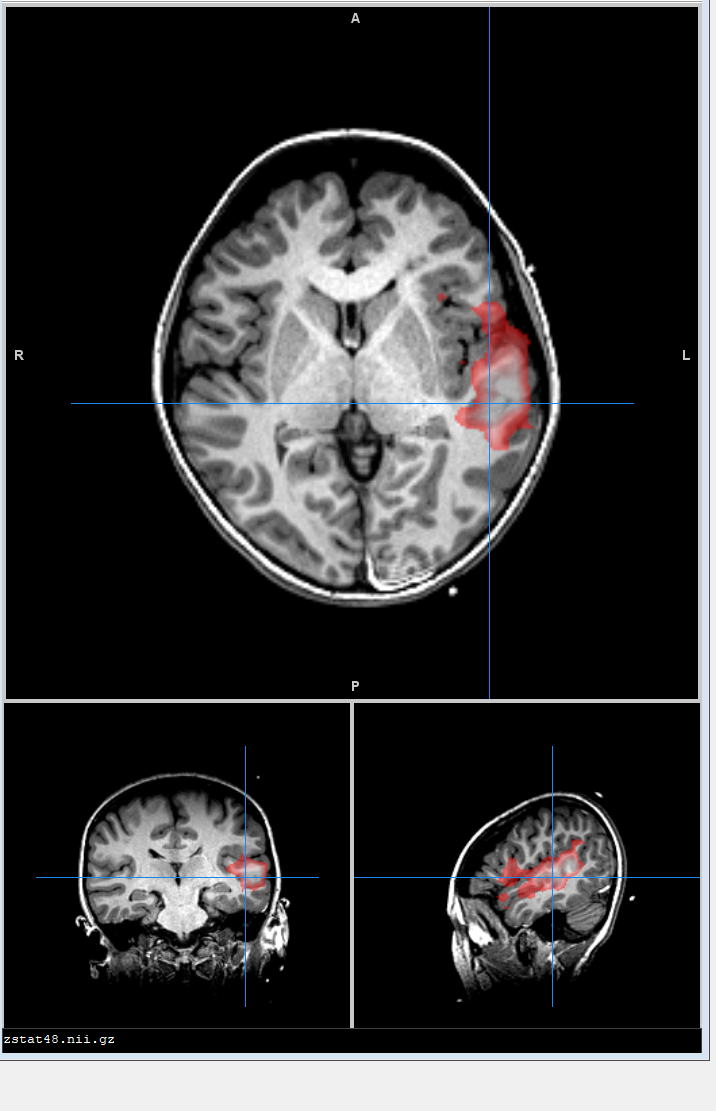 zstat48 | 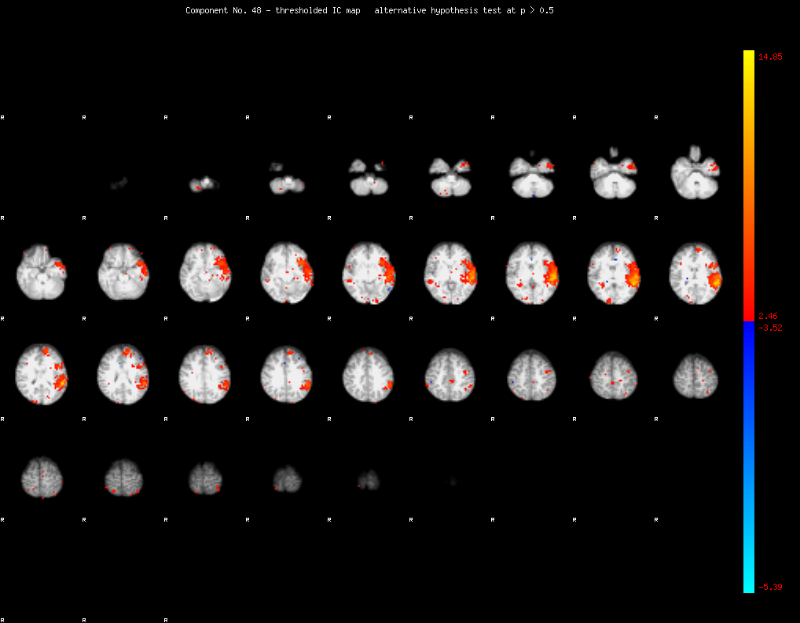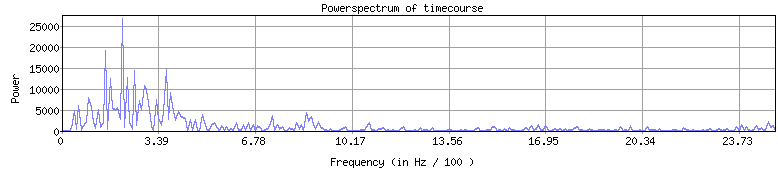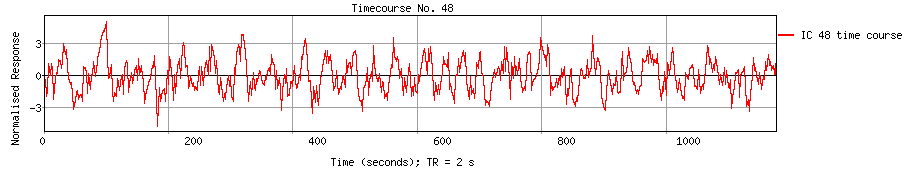 |
|  |  |
| 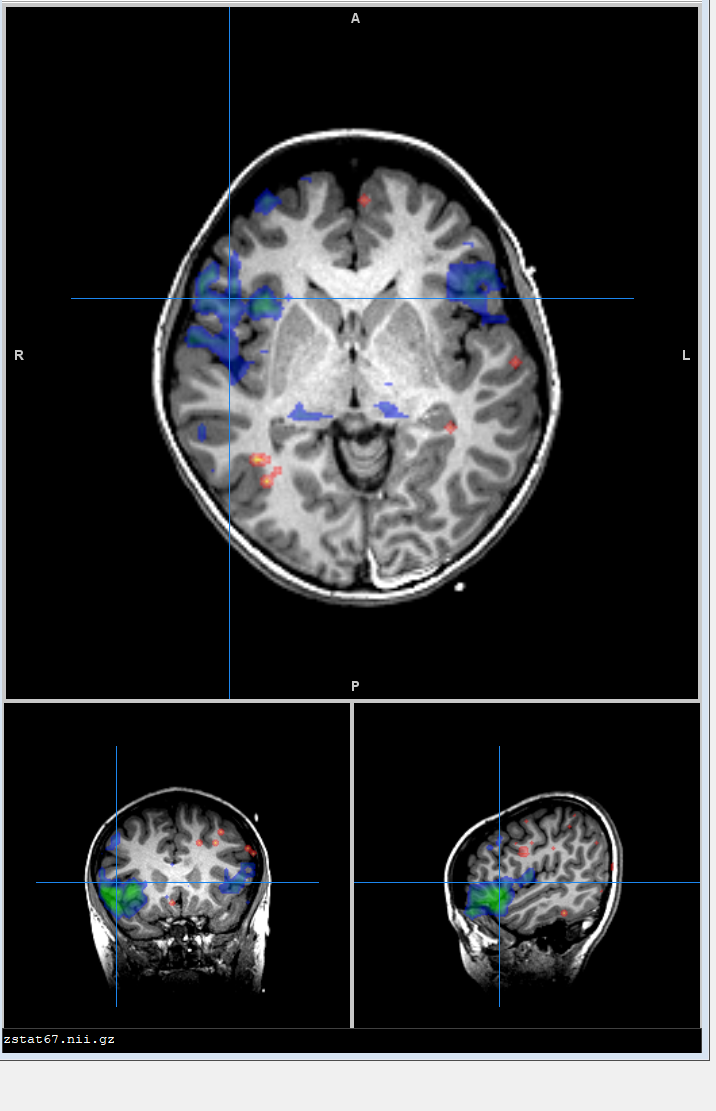 zstat67 | 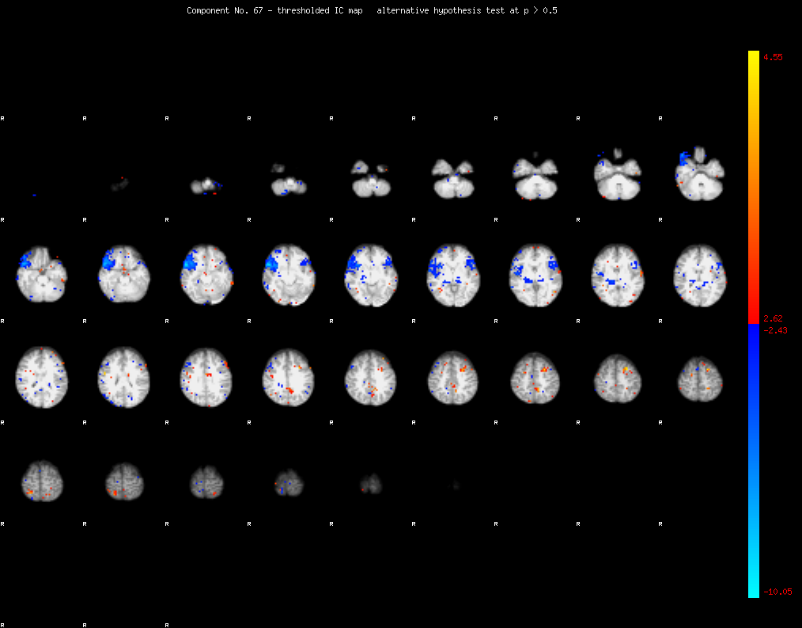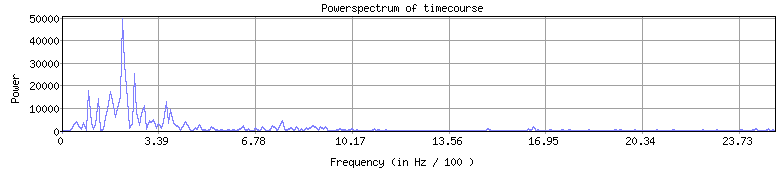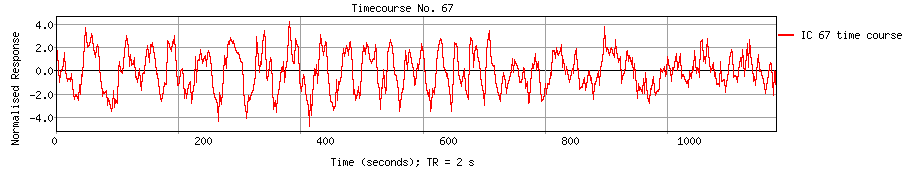 |
|  |  |

| **Parietal** |
| --- |

| 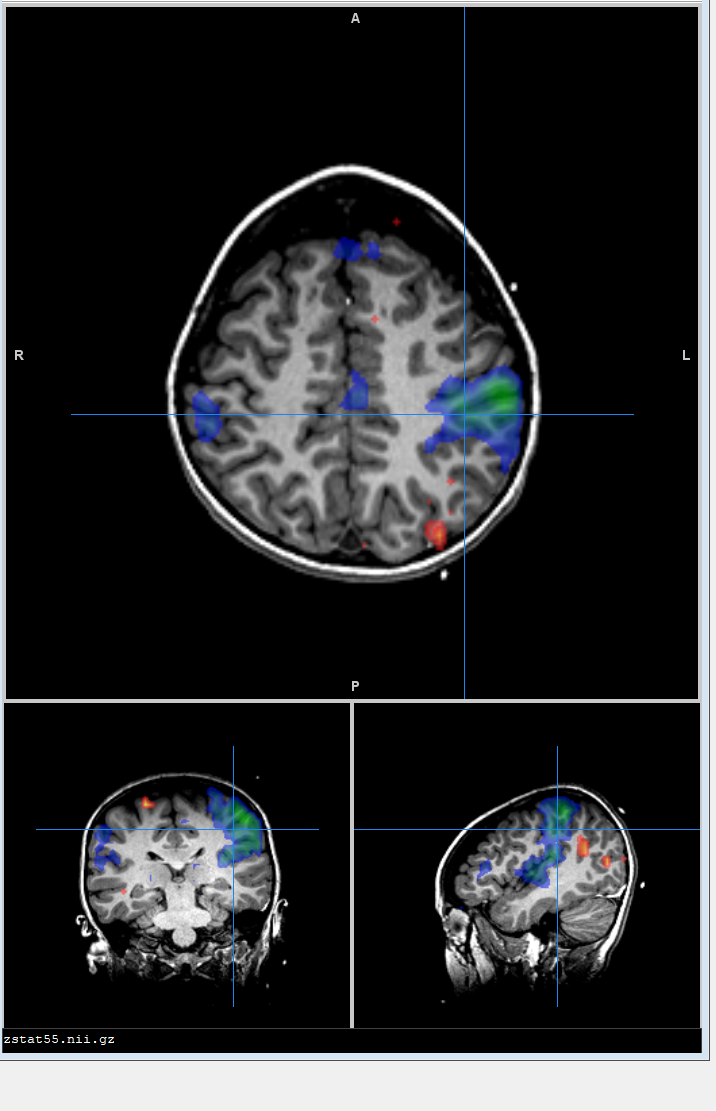 zstat55 | 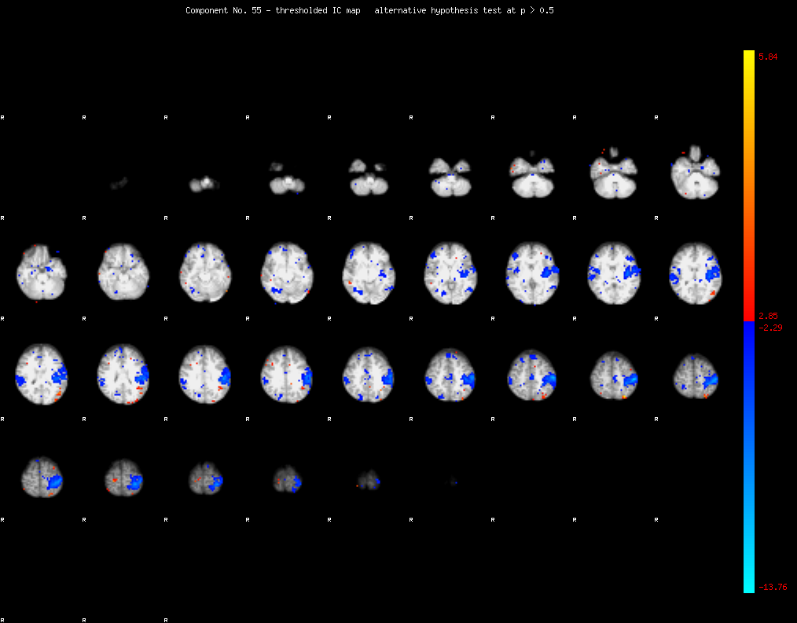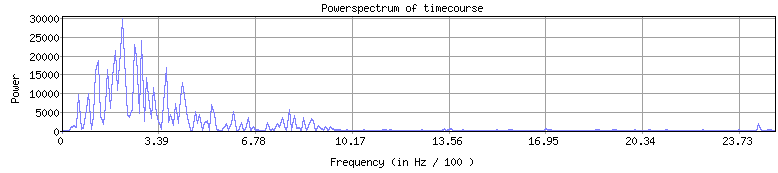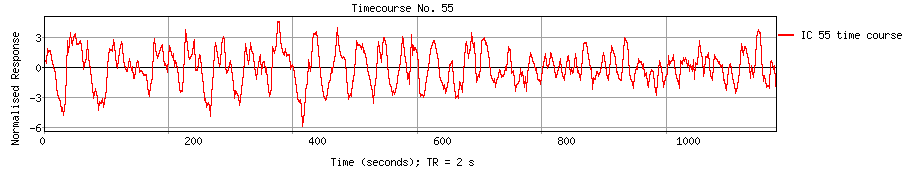 |
| --- | --- |
| 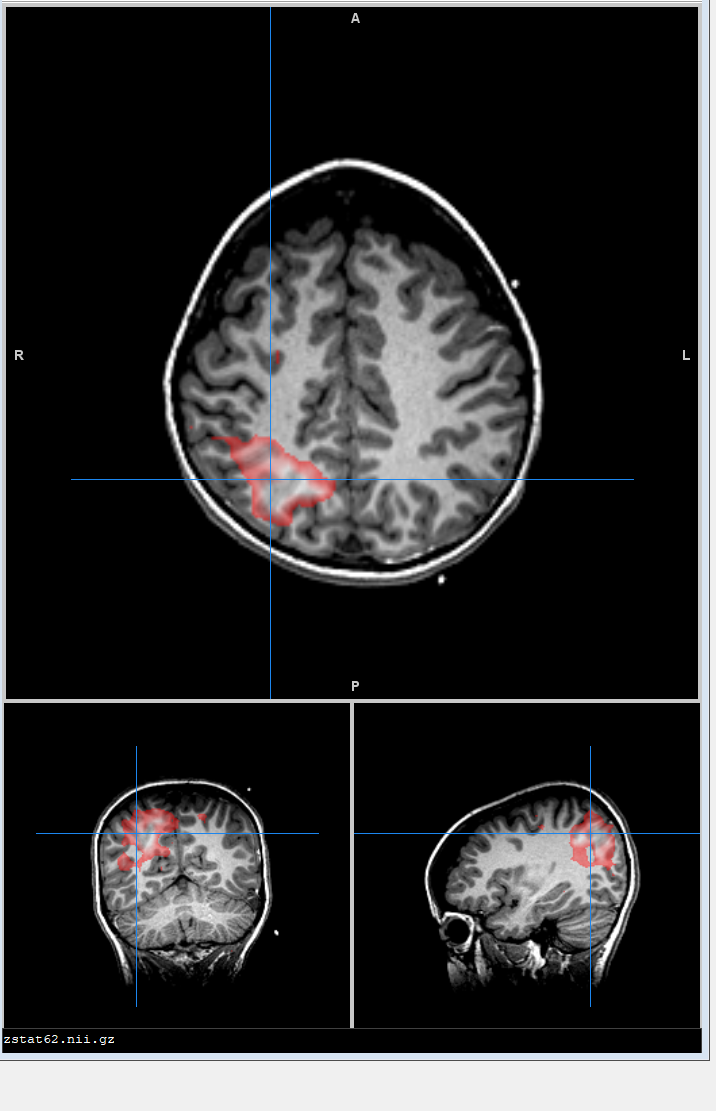 zstat62 | 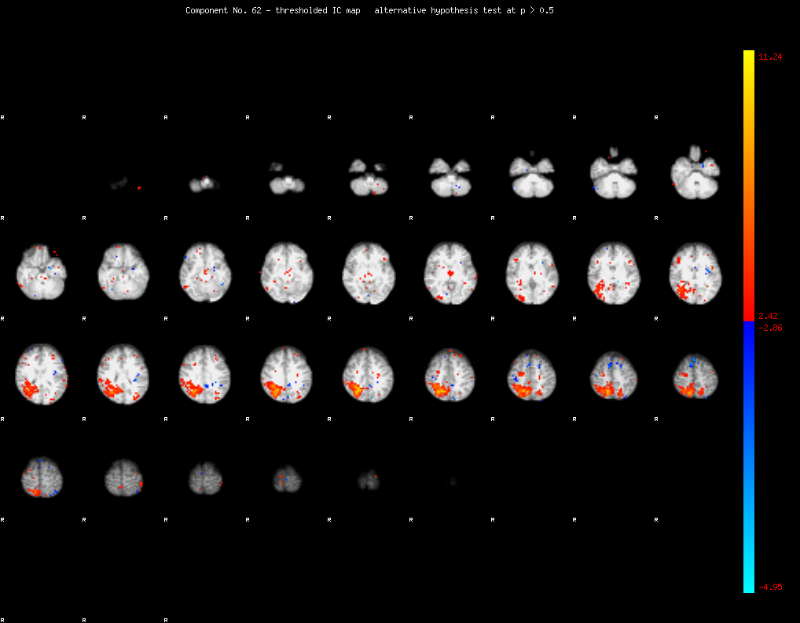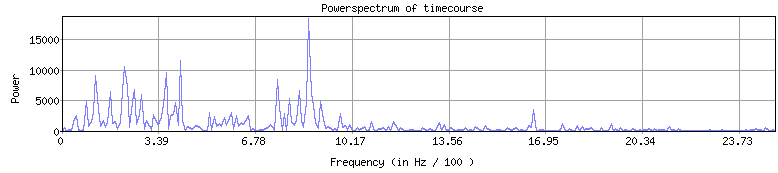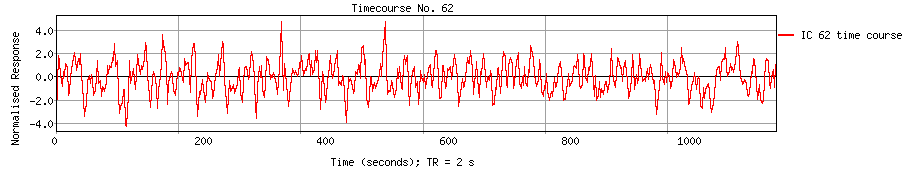 |
|  |  |

| **Frontal** |
| --- |

|  |  |
| --- | --- |
| 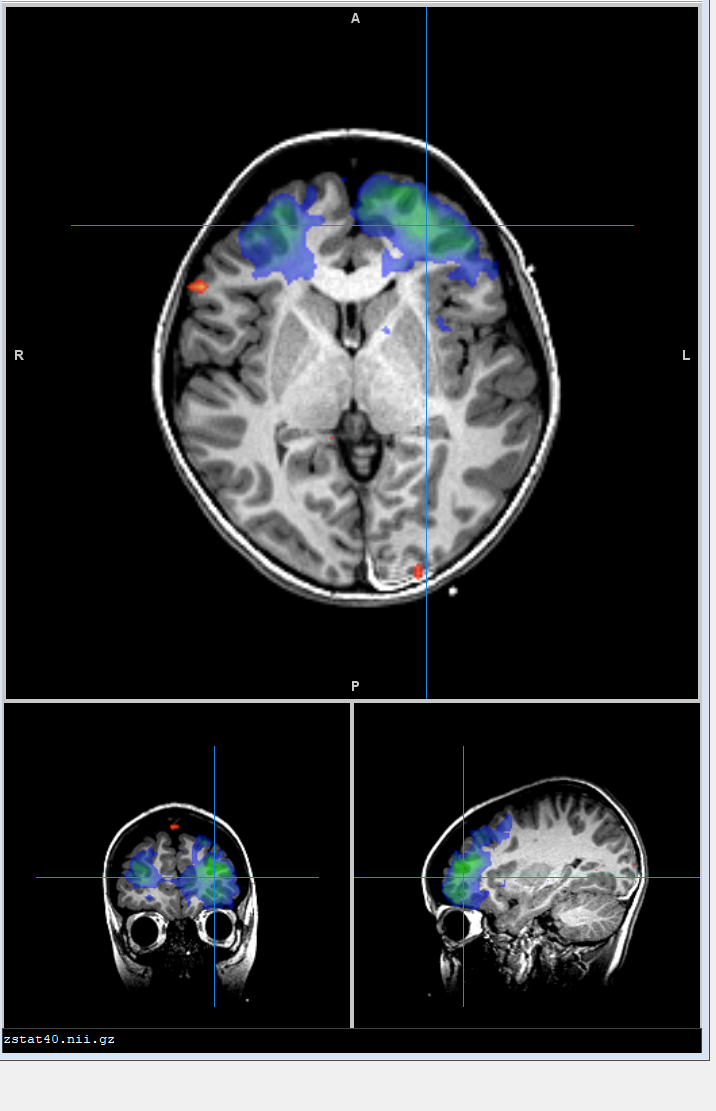 zstat40 | 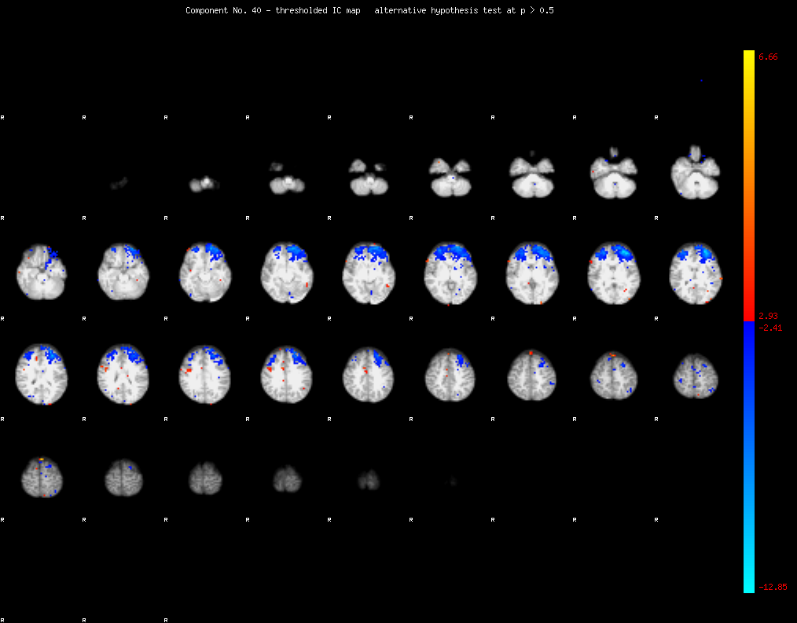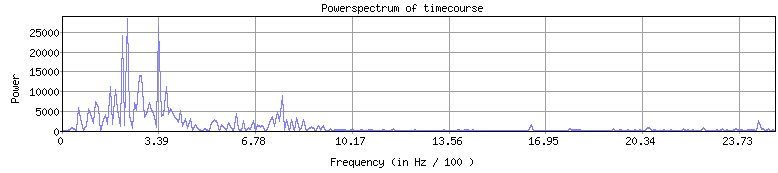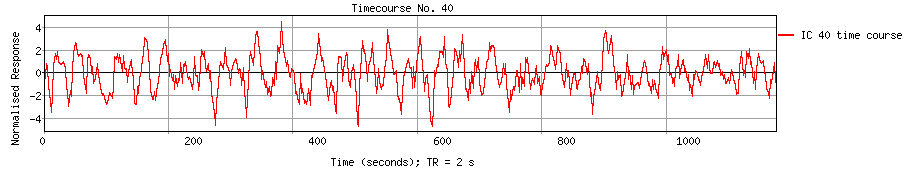 |
| 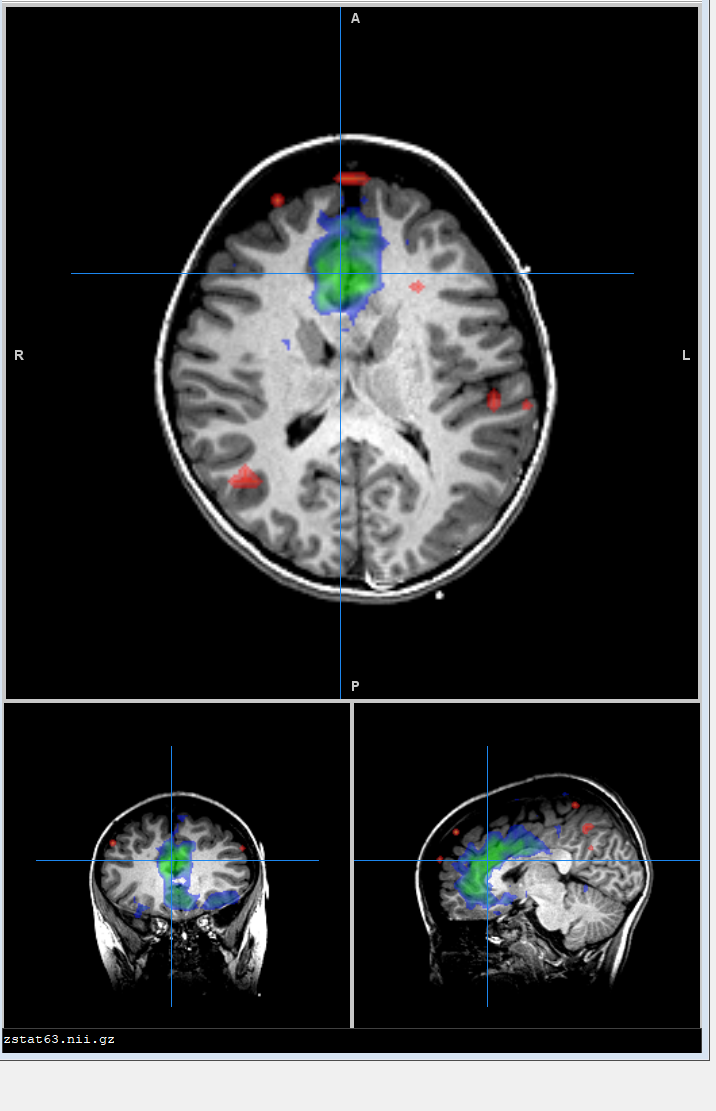 zstat63 | 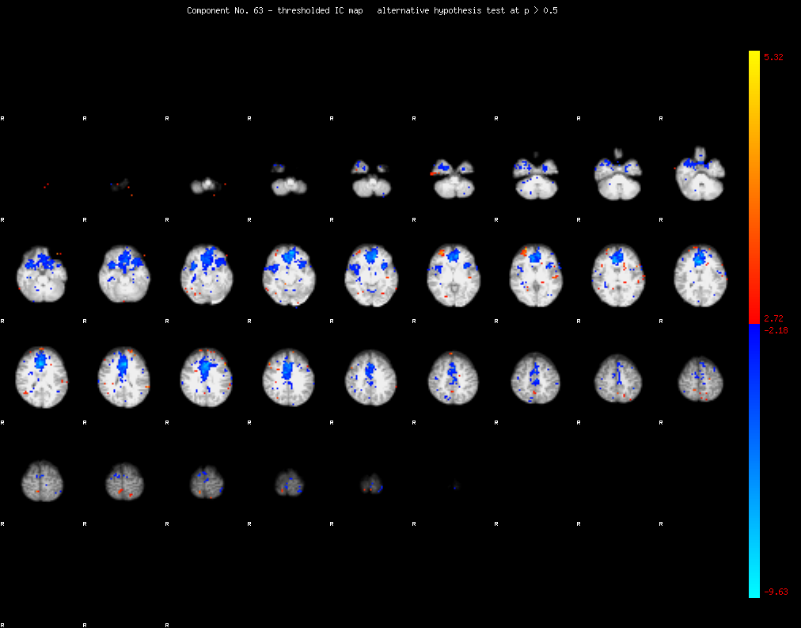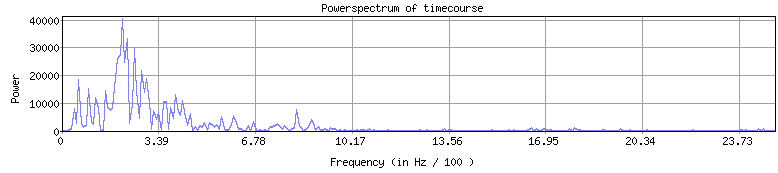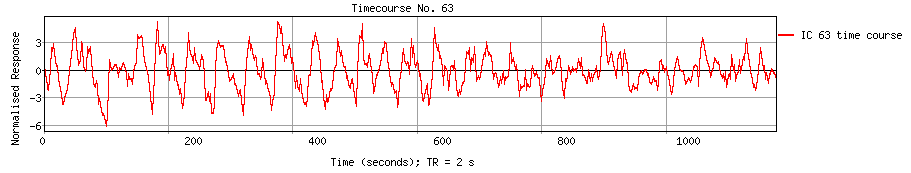 |
| 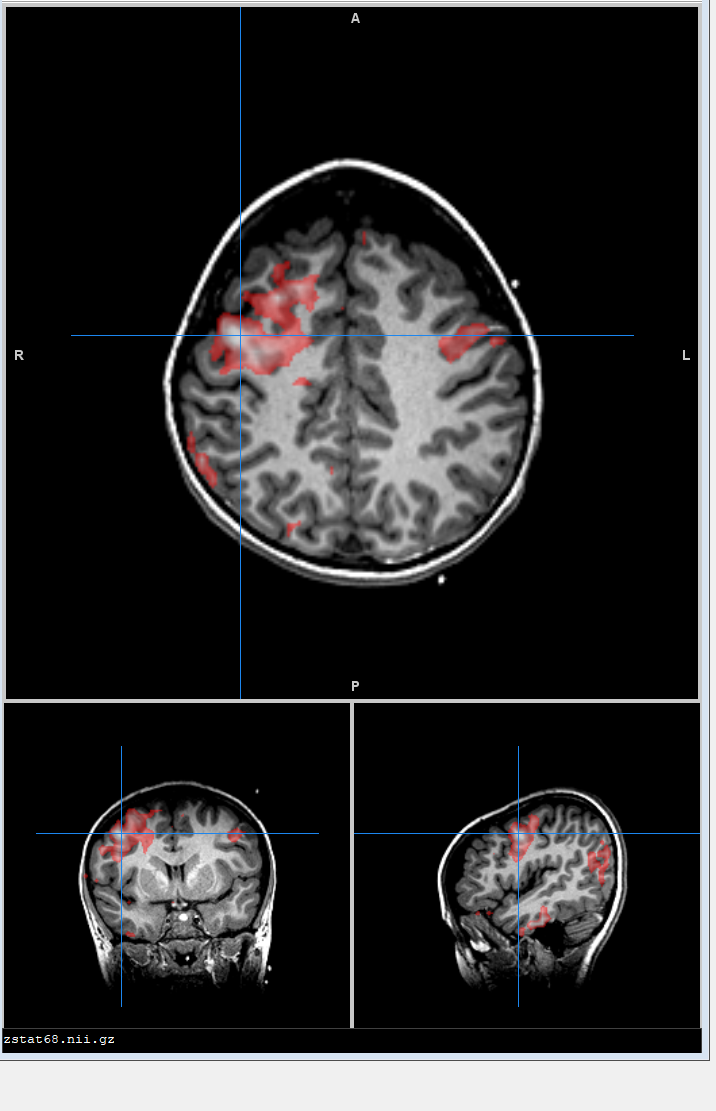 zstat68 | 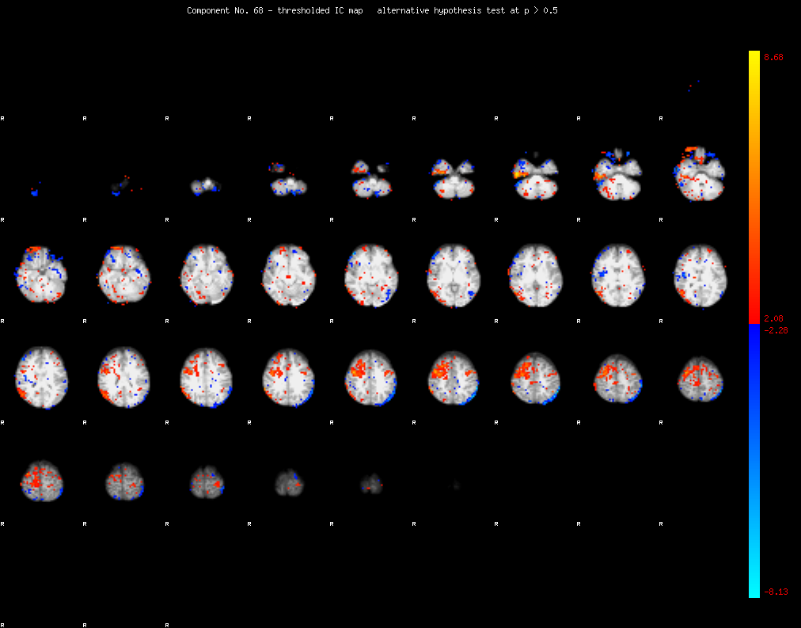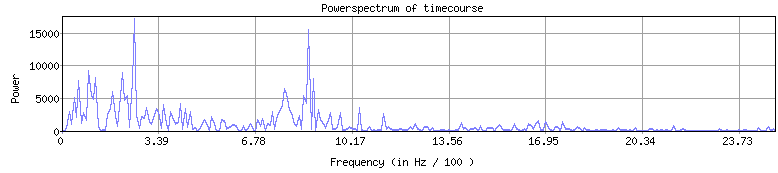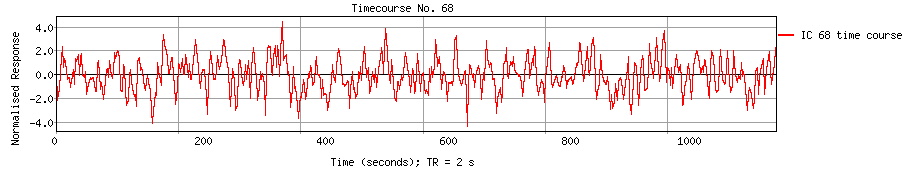 |
| 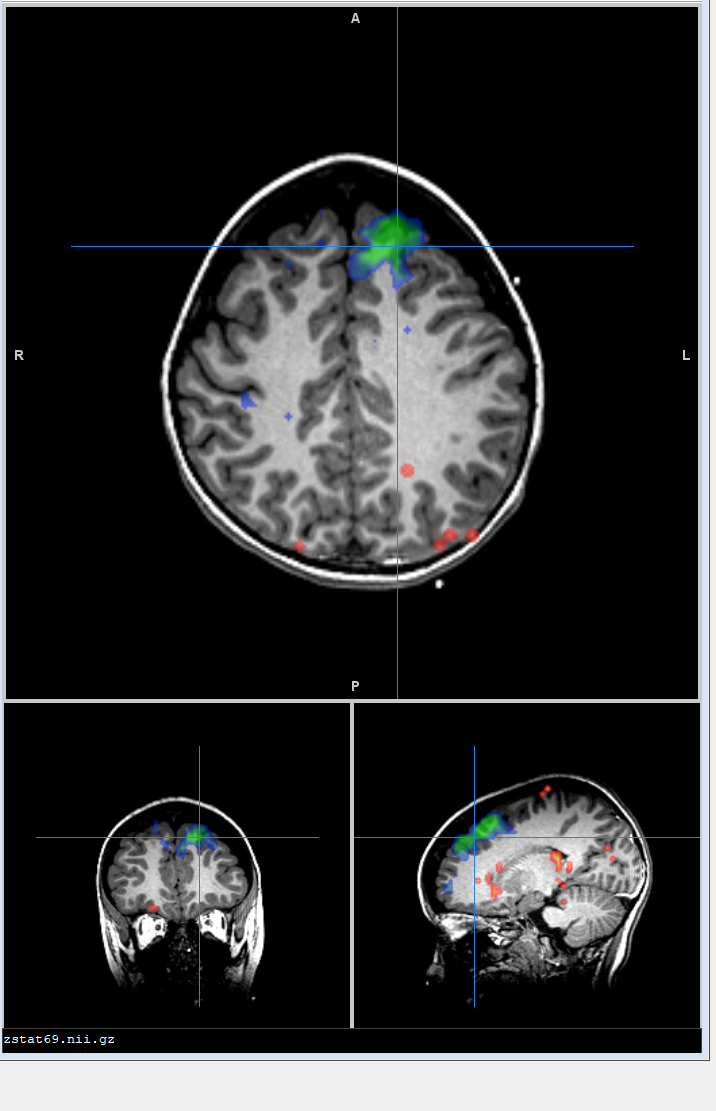 zstat69 | 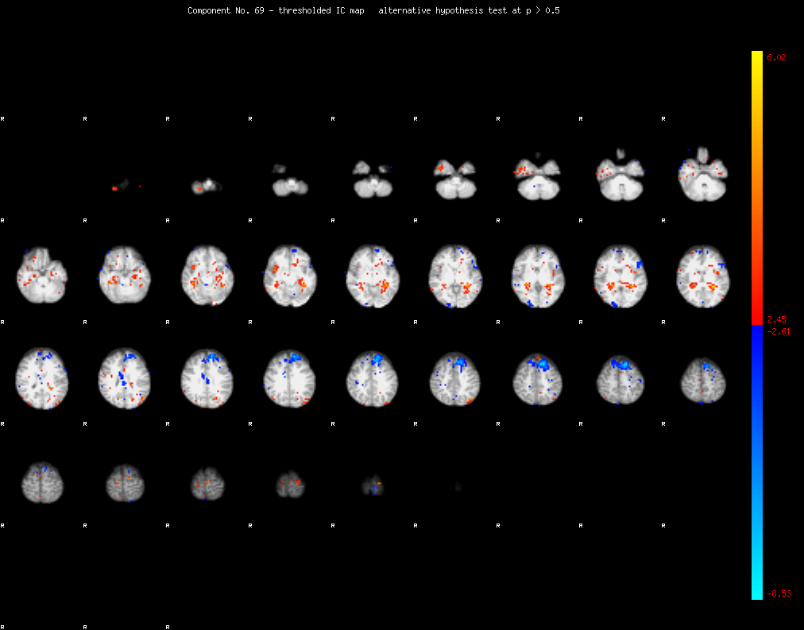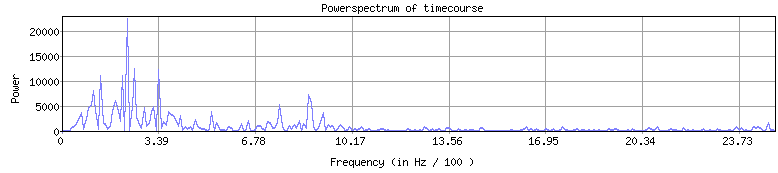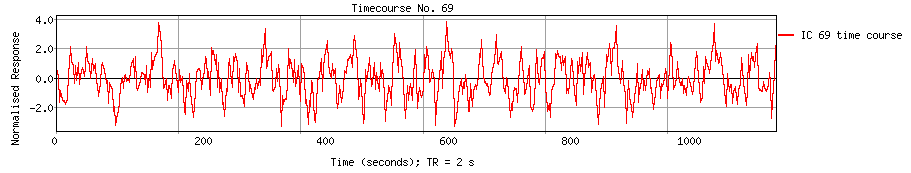 |
| 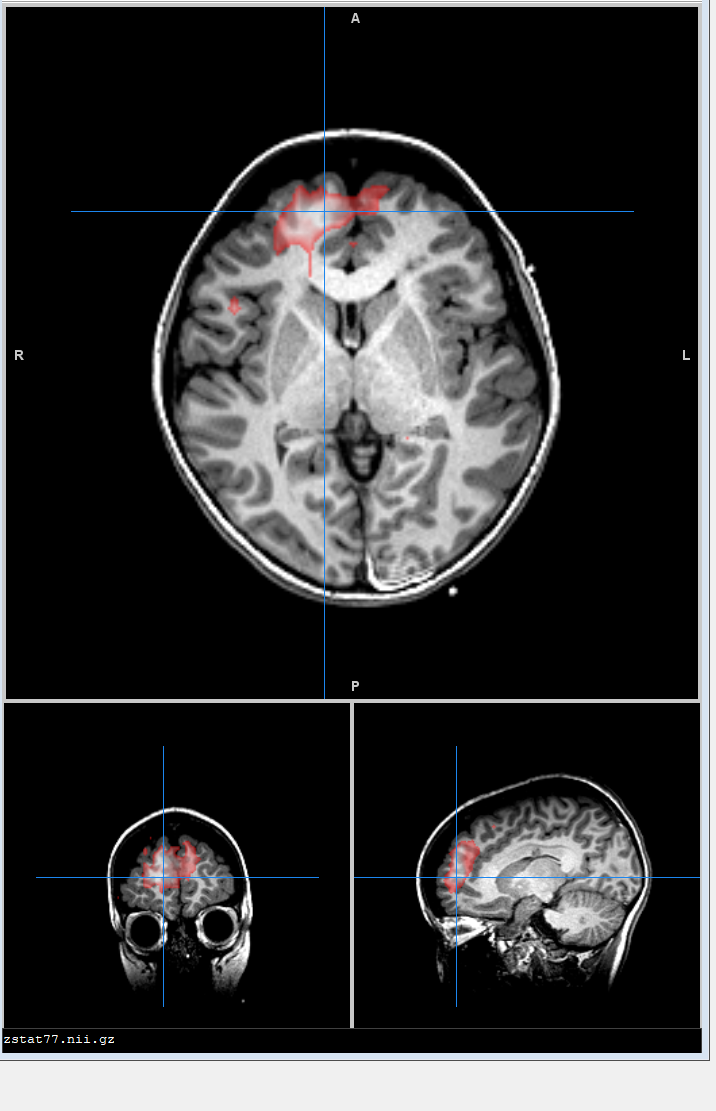 zstat77 | 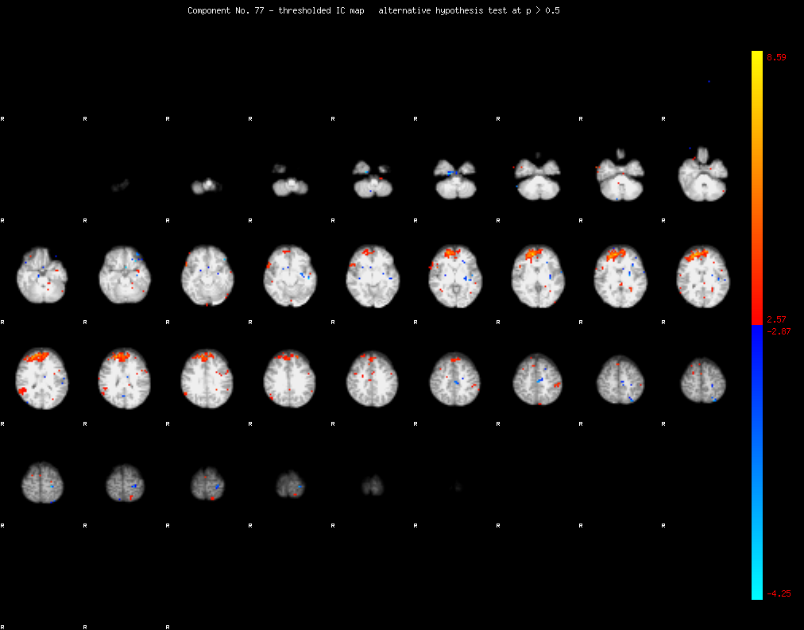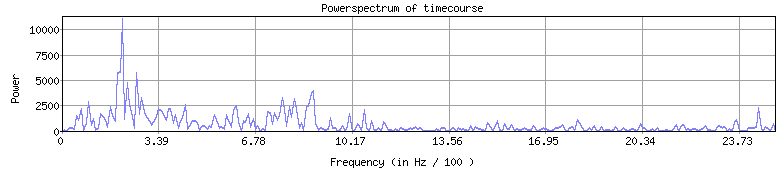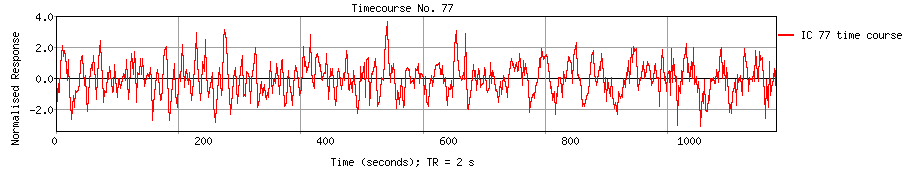 |
|  |  |

| **Temporal** |
| --- |

| 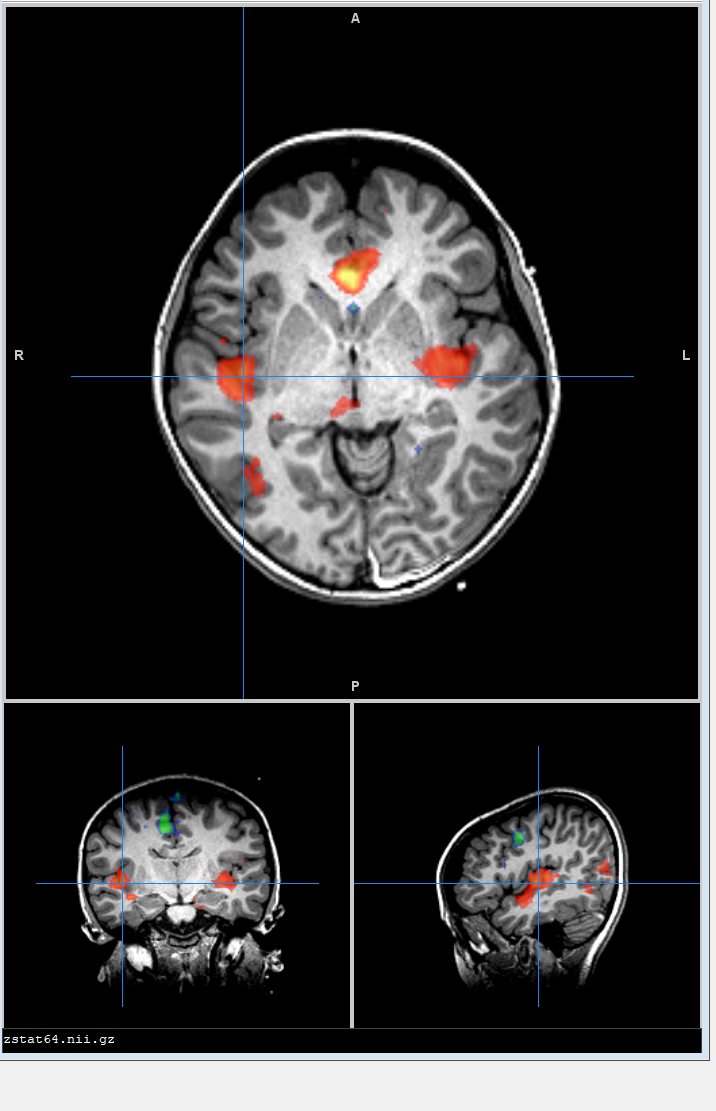 zstat64 | 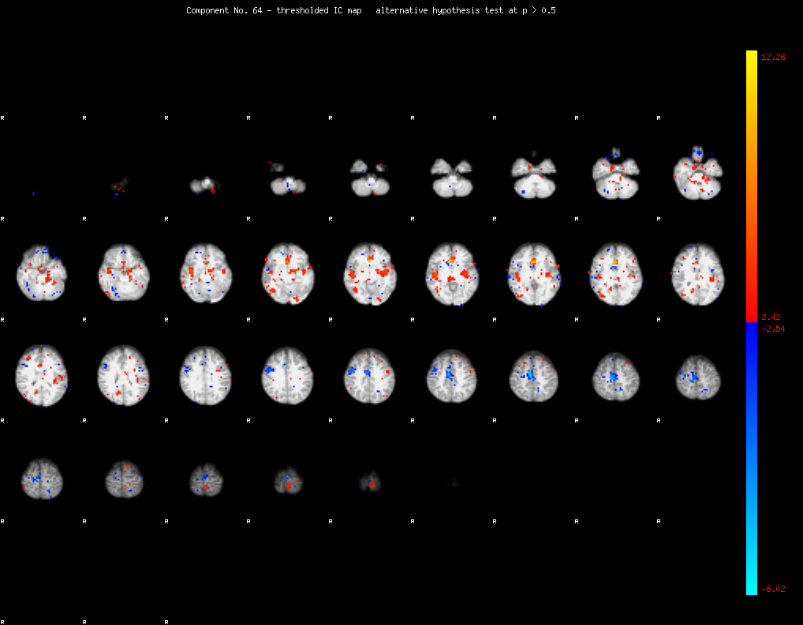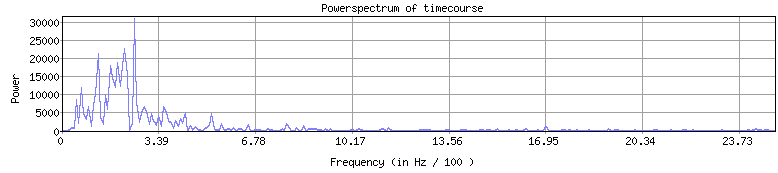 |
| --- | --- |

| **Vision** |
| --- |

| zstat8 |  |
| --- | --- |
| zstat11 |  |
| zstat52 |  |
|  |  |

| **Deep Grey** |
| --- |

| zstat25 |  |
| --- | --- |
| zstat47 |  |
| zstat71 |  |
|  |  |

| **Modulating** |
| --- |

| zstat36 |  |
| --- | --- |
| zstat37 |  |
|  |  |

| **Association: Long-range Fronto-Parietal** |
| --- |

| zstat39 |  |
| --- | --- |

| zstat34 |  |
| --- | --- |
| zstat50 |  |
|  |  |

| **Other** |
| --- |

| zstat45 |  |
| --- | --- |
|  |  |

| **Noise** |
| --- |

|  |  |
| --- | --- |
|  |  |
|  |  |
|  |  |
|  |  |
|  |  |
|  |  |
|  |  |
|  |  |
|  |  |
|  |  |
|  |  |
|  |  |
|  |  |
|  |  |
|  |  |
|  |  |
|  |  |
|  |  |
|  |  |
|  |  |
|  |  |
|  |  |
|  |  |
|  |  |
|  |  |
|  |  |
|  |  |
|  |  |
|  |  |
|  |  |
|  |  |
|  |  |
|  |  |
|  |  |
|  |  |
|  |  |
|  |  |

|  |  |
| --- | --- |

**Abbreviation Guide**

ACG Anterior Cingulate Gyrus

APF Anterior PreFrontal

AT anterior Temporal

B Bilateral

BG Basal Ganglia

DMN Default Mode Network

IFG Inferior Frontal Gyrus

IFS Inferior Frontal Sulcus

IPL Inferior Parietal Lobule

ITG Inferior Temporal Gyrus

L Left

LR-FTP Long Range Fronto-to-Parietal association network, anterior prefrontal IFG, MFG, posterior lateral parietal S2

LS2 – lateral Secondary Sensory area

MFG Middle Frontal Gyrus

MT mesial Temporal

MTG Middle Temporal Gyrus

mS2 – medial Secondary Sensory area

OPC- Operculum

OTG Occipito-temporal gyrus

PCG Posterior Cingulate Gyrus

PFC Prefrontal Cortex

PMC Premotor Cortex

PHG Parahippocampal Gyrus

POS Parietal Occipital Sulcus

R Right

Rs-fMRI Resting state functional MRI

RS Resting State

RSN Resting state network – an expected brain network

S1 Primary somatosensory cortex

S2 Secondary somatosensory cortex or secondary sensory association area

SFG Superior Frontal Gyrus

SFS Superior Frontal Sulcus

SMA Supplementary Motor Association area

SMG Supramarginal Gyrus

SOZ Seizure onset zone

SPL Superior Parietal Lobule

STG Superior Temporal Gyrus

STS Superior Temporal Sulcus

TOJ – Temporal Occipital Junction network

V1 Primary visual cortex associated network

V2 Secondary visual cortex associated network

vmPFC ventral medial prefrontal cortex

**Rest1 Motion:** absolute=0.06mm, relative=0.02mm

**Rest2 Motion:** absolute=0.05mm, relative=0.02mm
