# Supplementary material for: Pre- and post-therapy functional MRI connectivity in severe acute brain injury with suppression of consciousness: a comparative analysis to epilepsy features
Source: Front Neuroimaging. 2024 Oct 1;3:1445952. doi: 10.3389/fnimg.2024.1445952 (PMC11473429; doi:10.3389/fnimg.2024.1445952)
Supplement: Supplementary file 9 [file Table_9.DOCX]

Resting State functional MRI Whole Brain Map

| **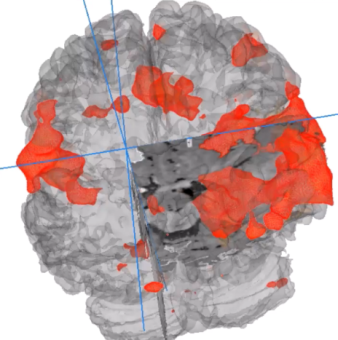** |  |  |
| --- | --- | --- |

PP1 MRI#1

Anatomical MRI - see separate radiological report,

Technique and analysis Methods 3T MRI, whole brain BOLD ICA

Data Quality Analysis 20 min; Head motion < 1 mm, no interfering artifacts detected

Abbreviation index list at end of rs-report

Impression:

1. **Rs-fMRI seizure onset zone**: 10 R MT/Opc with 5 being bilateral ***Possibly interfering with expression of consciousness
2. **Language**: well detected
3. **Whole brain networks**: Well detected overall (sparse in parietal, however Long-range fronto-parietal detected).

| Rs-fMRI Informed Neuro-Prognostication | | |
| --- | --- | --- |
| Consciousness | Alertness and Awareness  Equivalent of 0=Coma, 1=Normal Consciousness, 2=MCS, 3=VS/UWS | 1 |
| Developmental Stream | 0=very poor, 1=Normal, 2=Atypical but not very poor, 3=Very poor, 4=Indeterminate | |
| Motor | Walking | 1 |
|  | Gross motor body movement | 1 |
|  | L - arm/hand fine motor | 1 |
|  | R- arm/hand fine motor | 1 |
|  | face/mouth motor coordination | 1 |
| Motor tone | R appendicular tone | 1 |
|  | L appendicular tone | 1 |
|  | Central tone | 1 |
| Language | Understanding words | 1 |
|  | Speaking in Words | 1 |
| Vision | R field primary visual reception | 1 |
|  | R field higher level visual stimuli interpretation | 1 |
|  | L field primary visual reception | 1 |
|  | L field higher level visual stimuli interpretation | 1 |
| Sensory | Touch/Sense of Spatial/complex relationship | 1 |
| Cognition/Learning/Memory | equivalent IQ >70 | 1-2 |
|  | equivalent IQ < 70 |  |
|  | Profound Intellectual Disability Equivalent IQ < 35 |  |

* Scoring unless otherwise specified is shown as the possible highest score expected or range

| Network Characterization | | | | | |
| --- | --- | --- | --- | --- | --- |
| 1=yes, 0=no | Detected | Normal | Atypical | Comment | |
| Motor | 1 |  | 1 | Normal spatial, mild-mod freq incr regular | |
| Language | 1 |  | 1 | Deactivated largely, incr freq, regular, mildly sparse than typical number of networks | |
| Parietal | 1 |  | 1 | 1no bilateral connection, sparse | |
| Frontal | 1 |  | 1 | Mild to mod inc freq, regular | |
| Temporal | 1 |  | 1 | See language | |
| Vision | 1 |  | 1 | Normal spatial, regular inc freq | |
| Deep grey | 1 |  | 1 | Normal spatial, regular inc freq mild | |
| Modulating | 1 |  | 1 | Normal spatial, regular inc freq mild | |
| Association | 1 |  | 1 | Normal spatial, regular inc freq mild | |
| Atypical/possible pathological | Summary 10 R MT/Opc with 5 being bilateral  (18) R MT/R vmPFC  (1) R > L MT, T/O  (54) R T/Opc  (4) R >L T/Opc  (7) B T/Opc  (10) R > L T/Opc  (12) B mT/AT  (14) Opc  (15) B Opc  (2) B Opc RSN | | | | |
| **Atypical Networks** | | | | |  |

| zstat 18  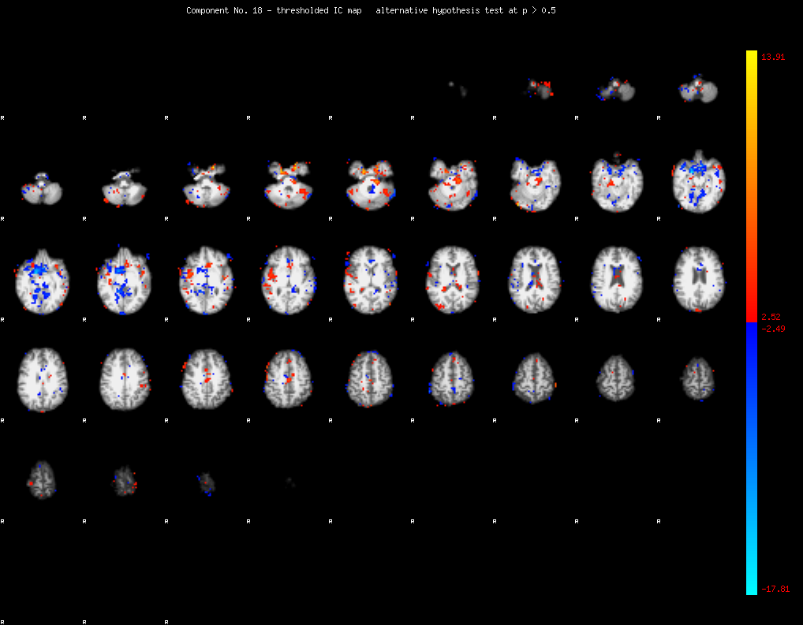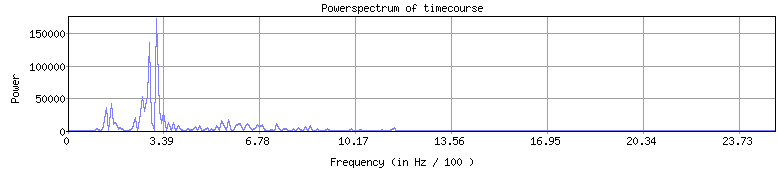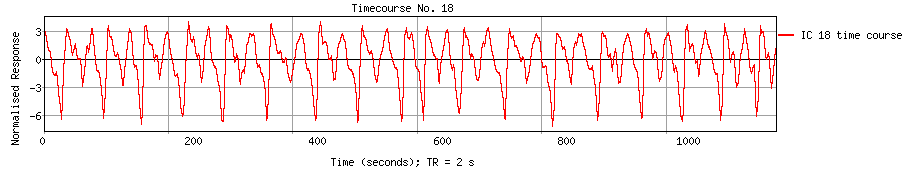 |
| --- |

| zstat 1  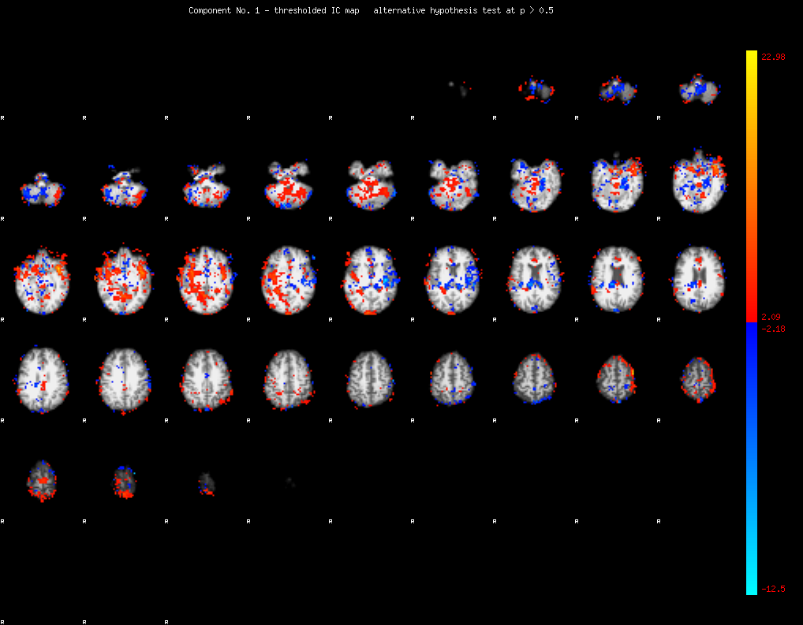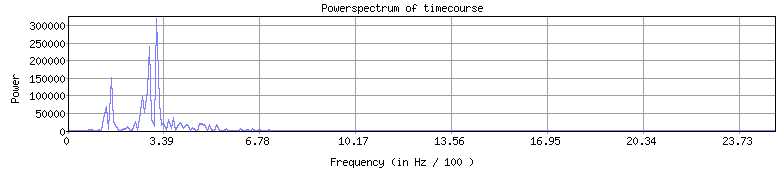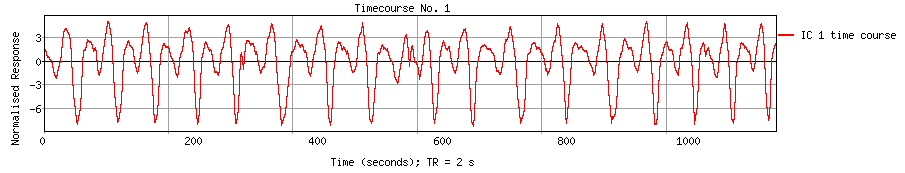 | |
| --- | --- |
|  |  |

zstat 54


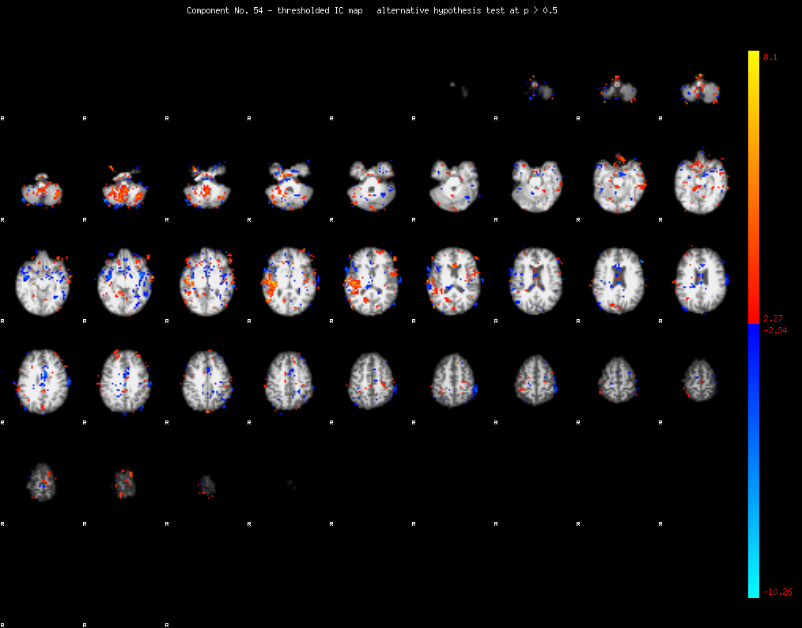

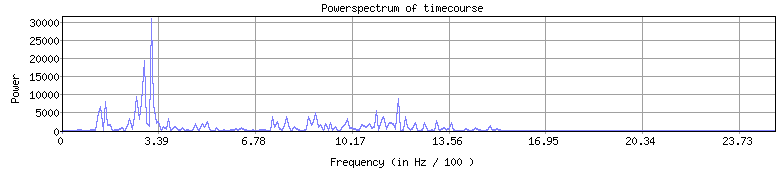

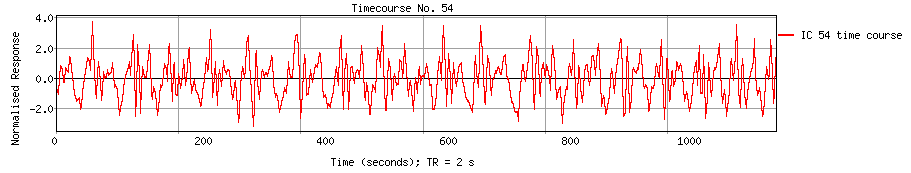


| zstat 4  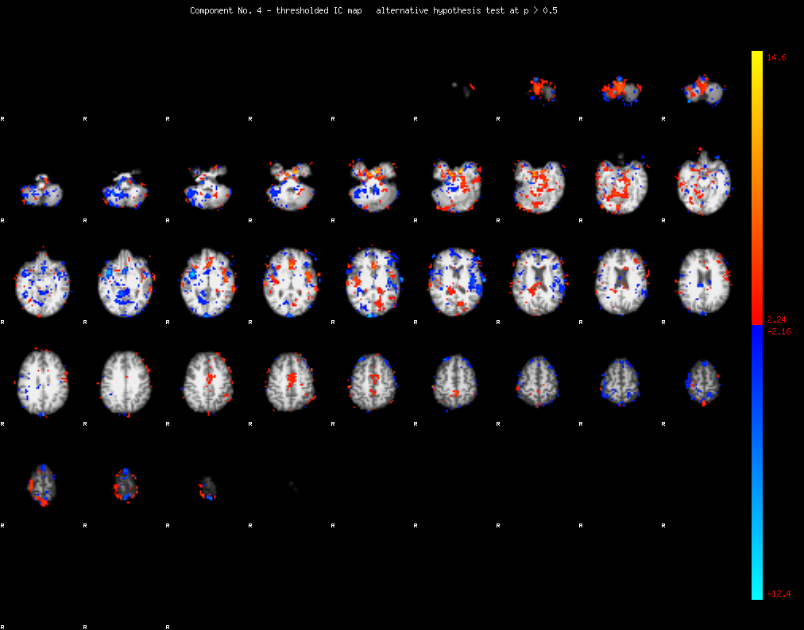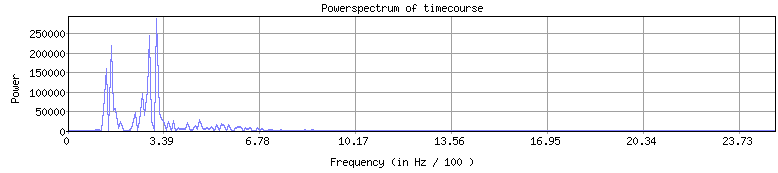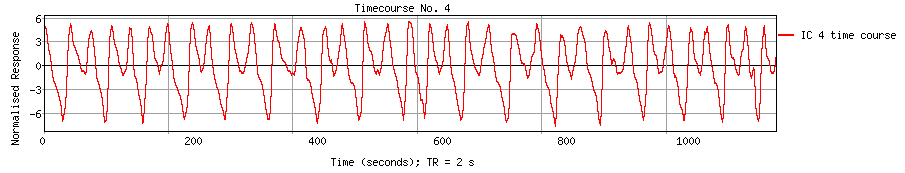 |
| --- |

| zstat 7  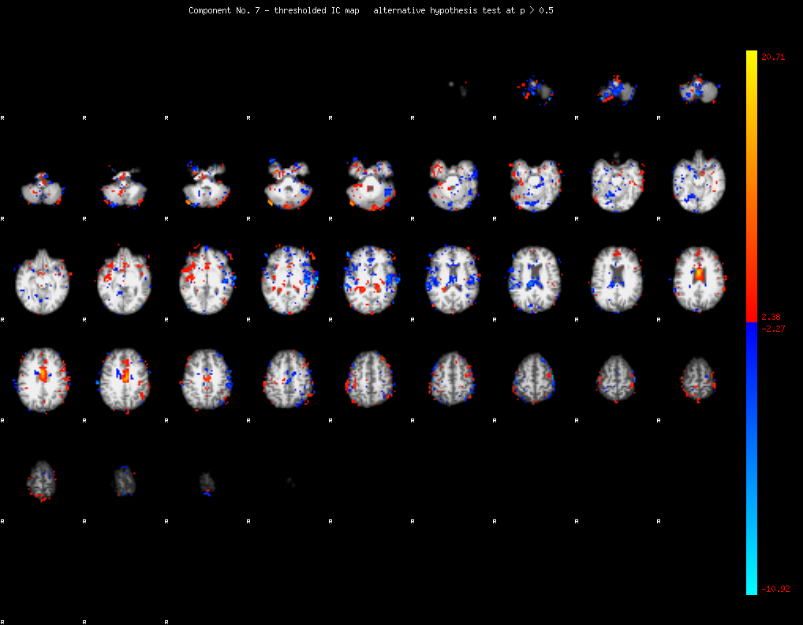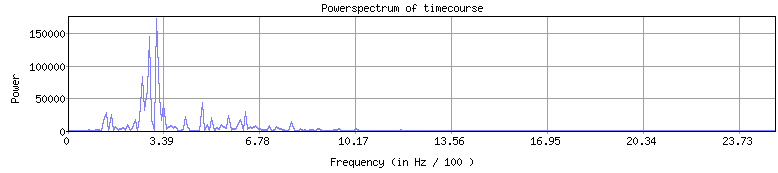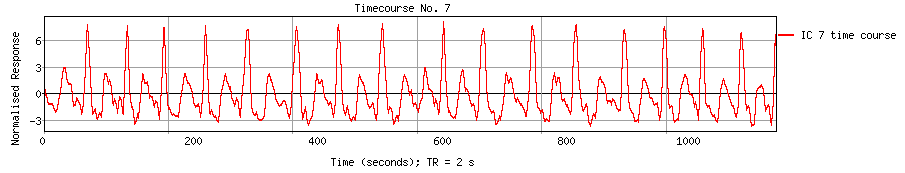 |
| --- |

| zstat 10  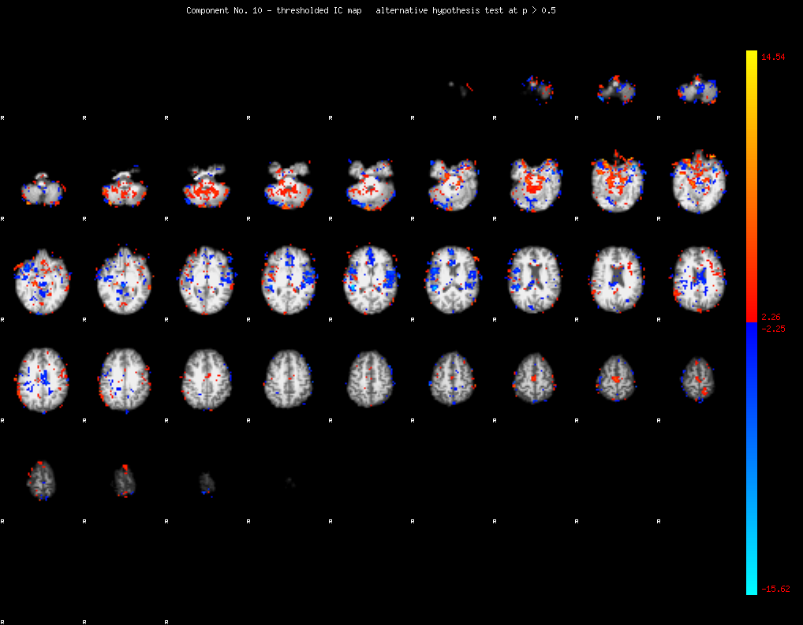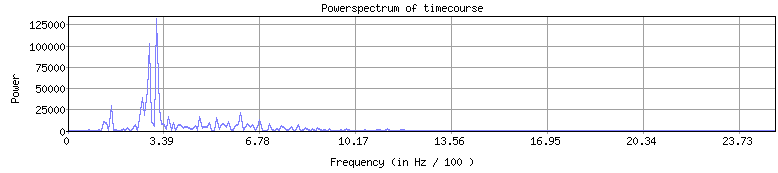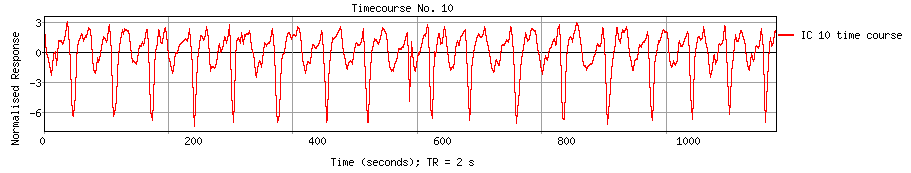 |
| --- |

| zstat 12  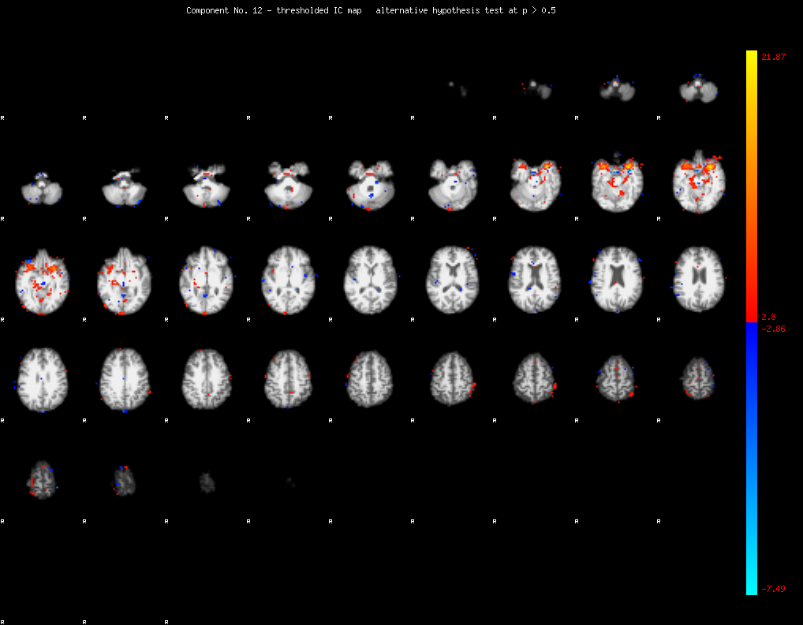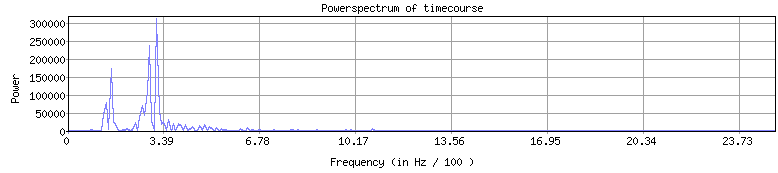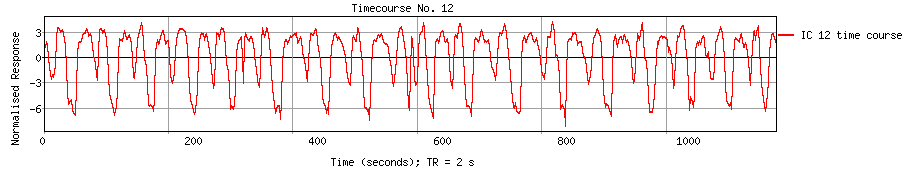 |
| --- |

| zstat 14  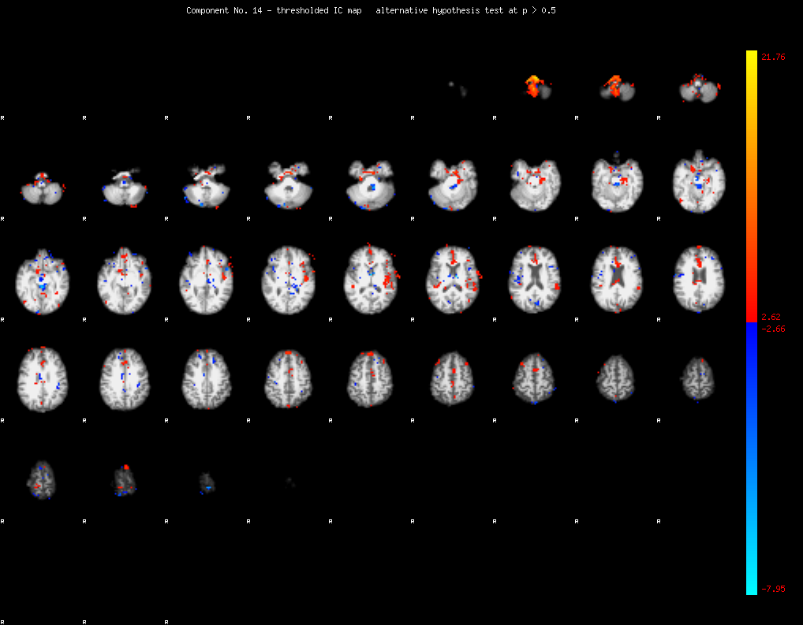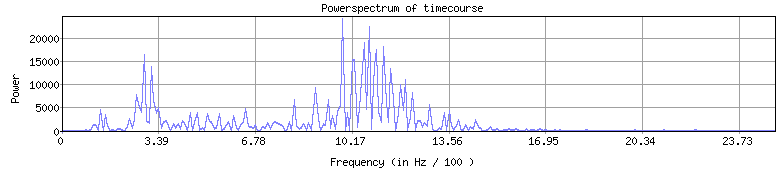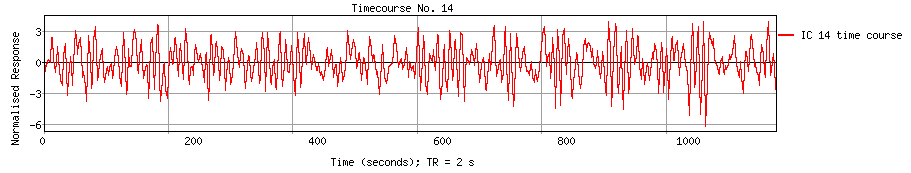 |
| --- |

| zstat 15  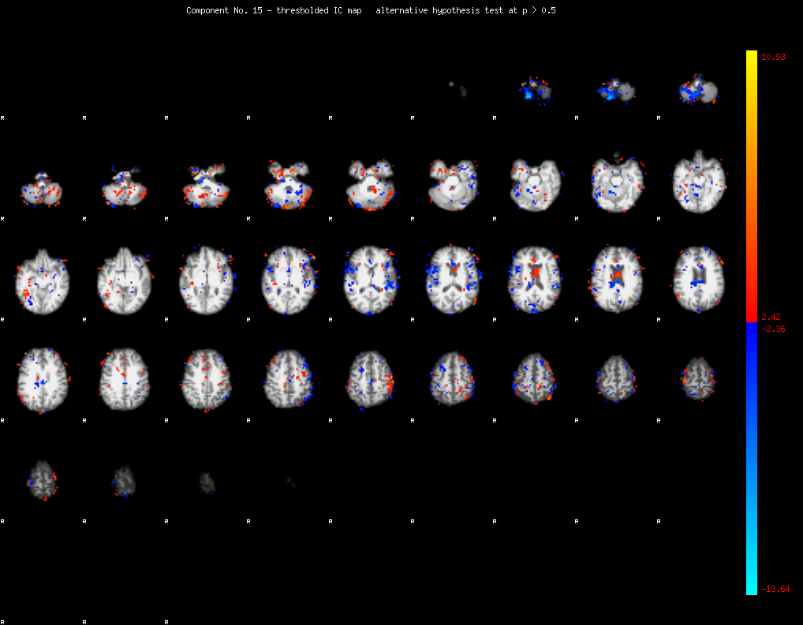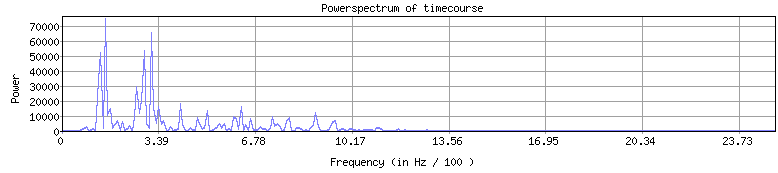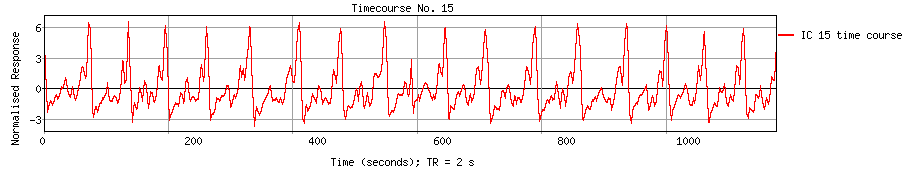 |
| --- |

**
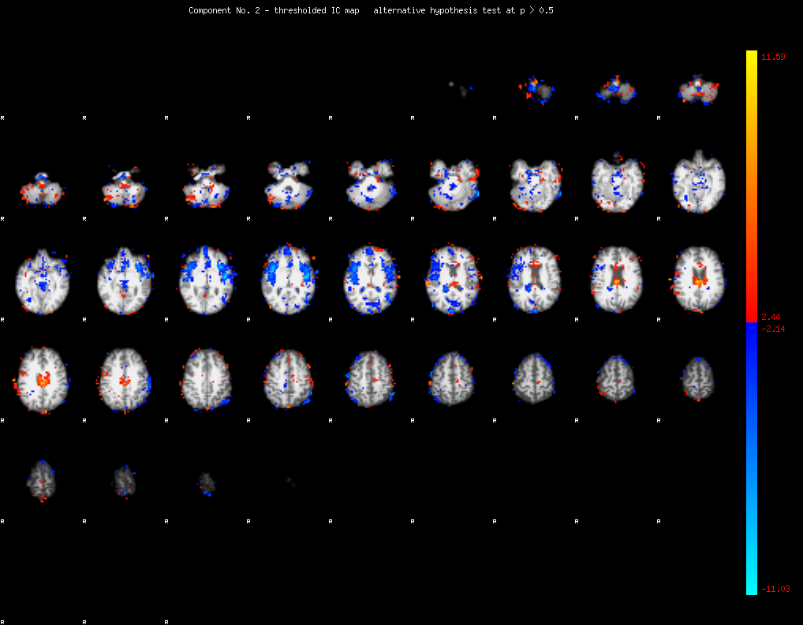

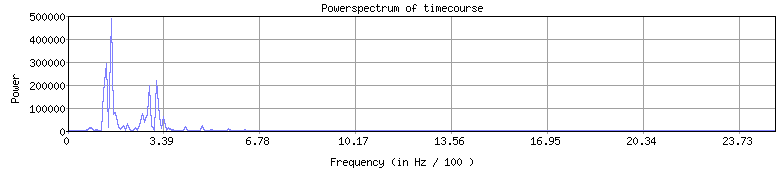

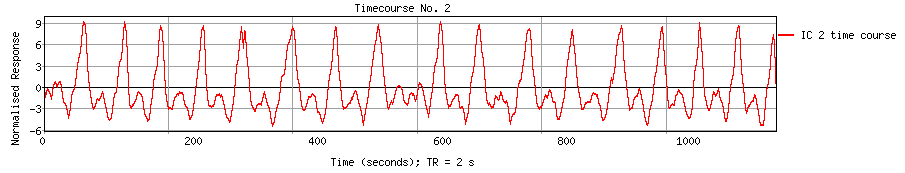
**

| **Atypical RSN** |
| --- |

|  |  |
| --- | --- |
|  |  |

| **Motor** |
| --- |

| 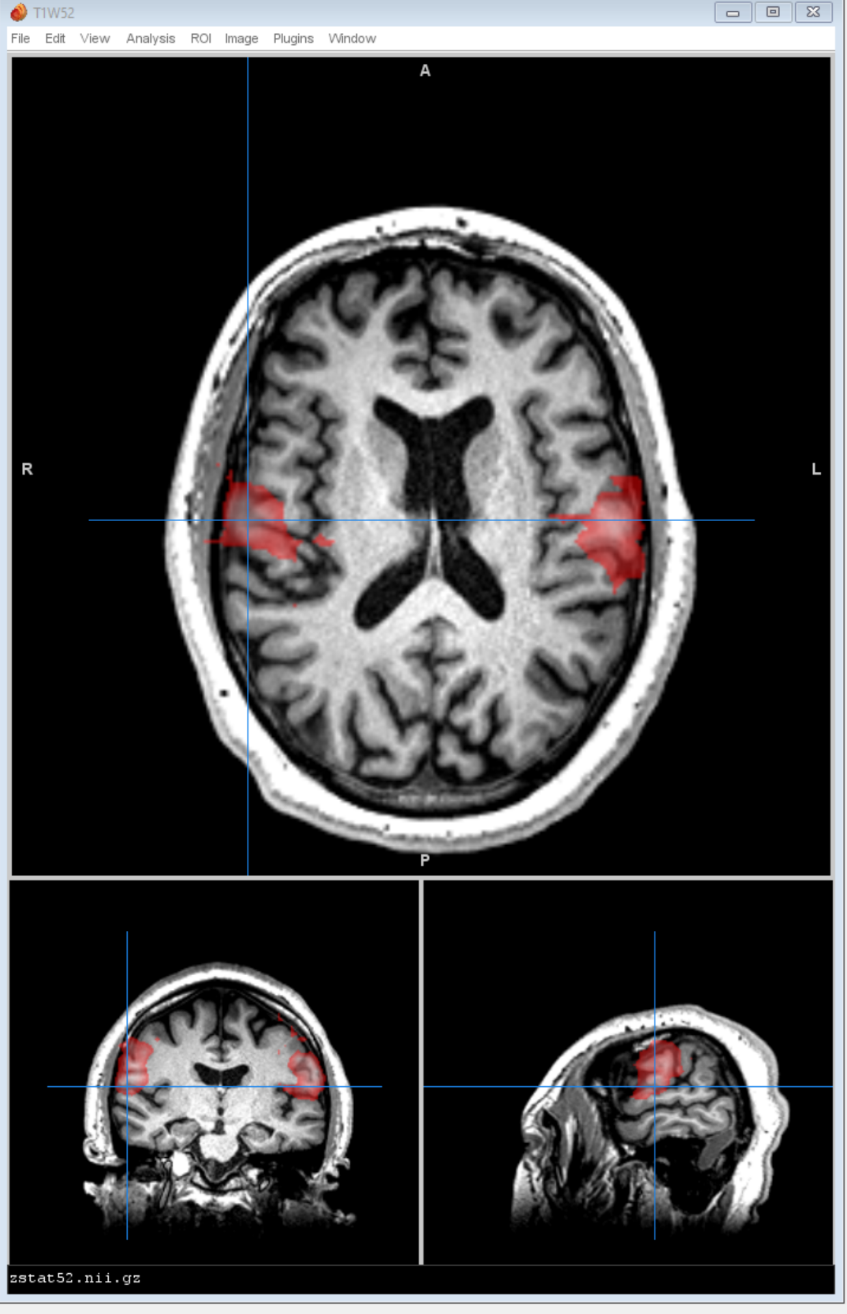 zstat 52 | 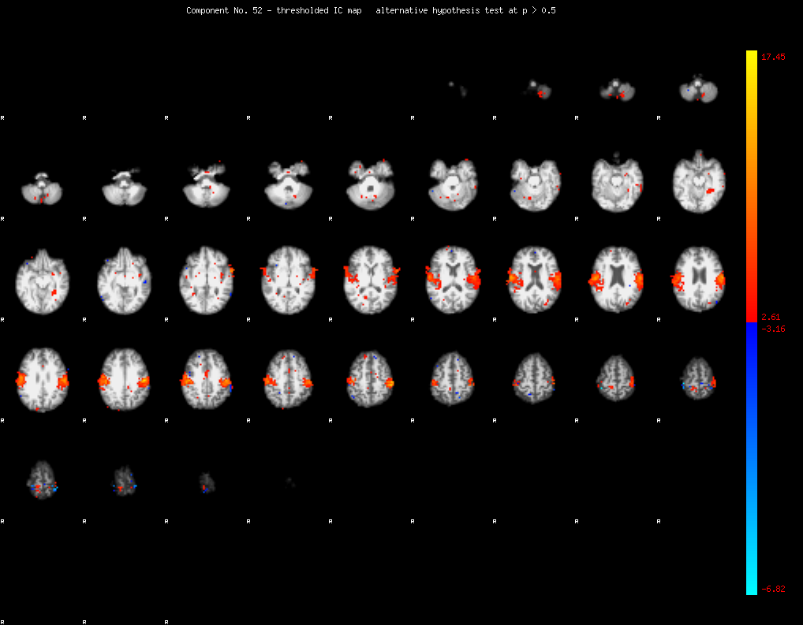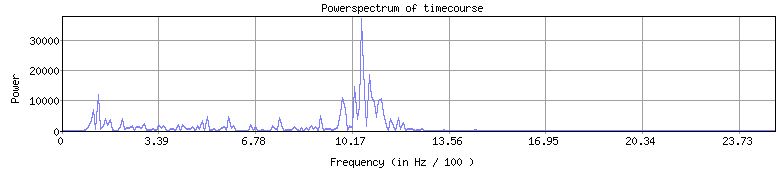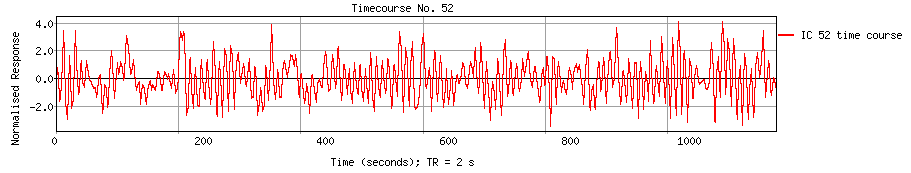 |
| --- | --- |
| 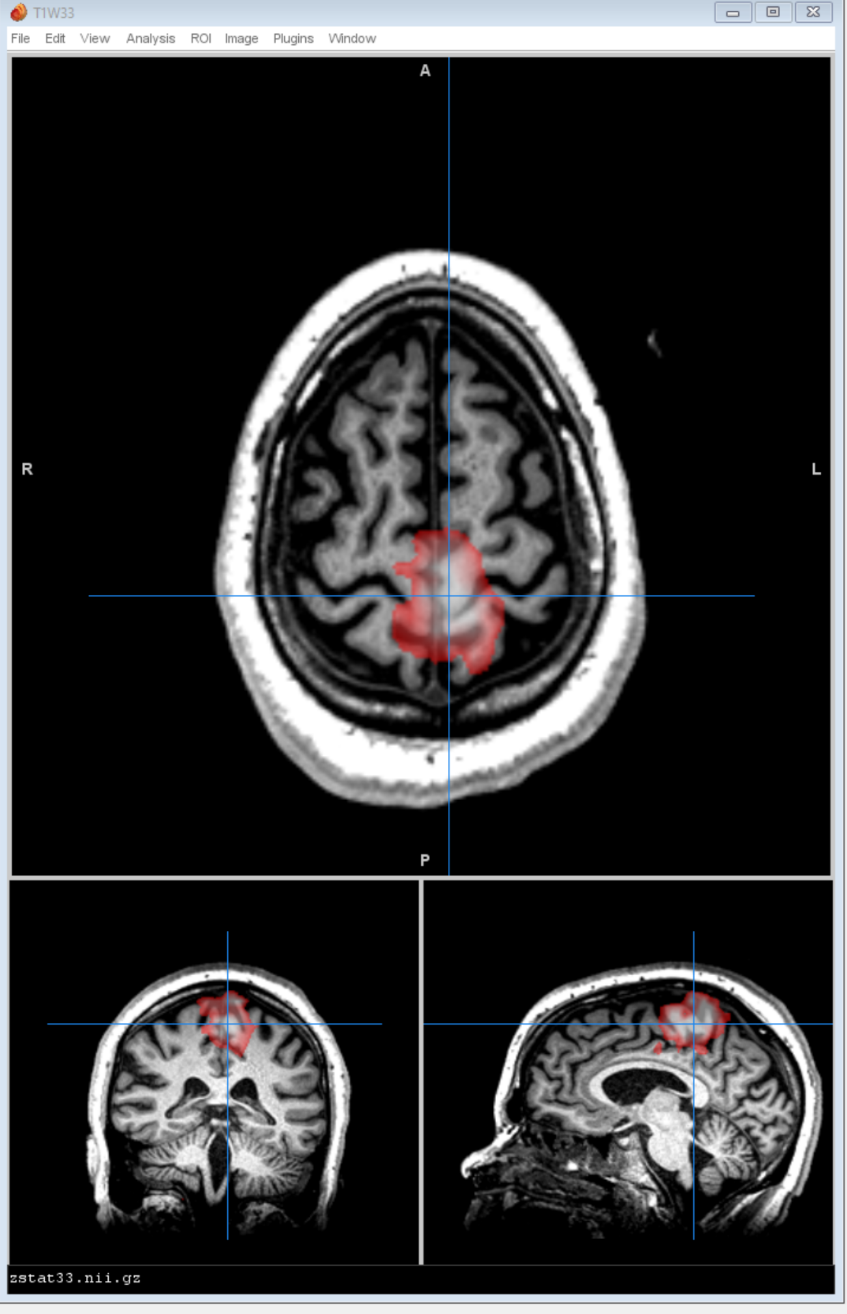 zstat 33 | 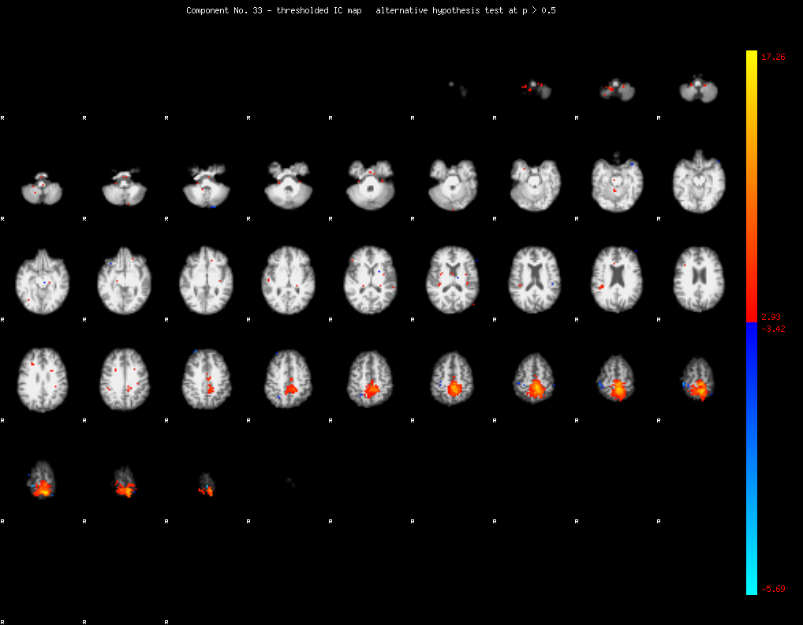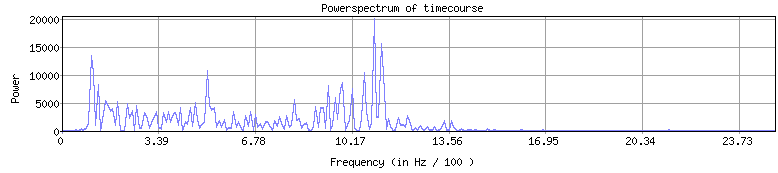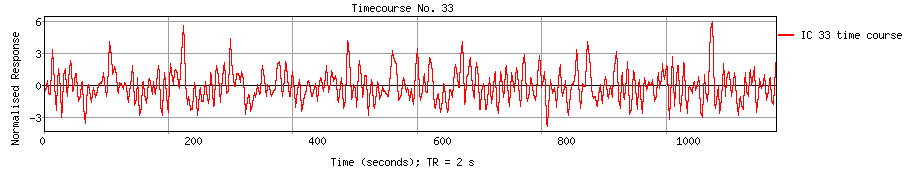 |

zstat 62

**
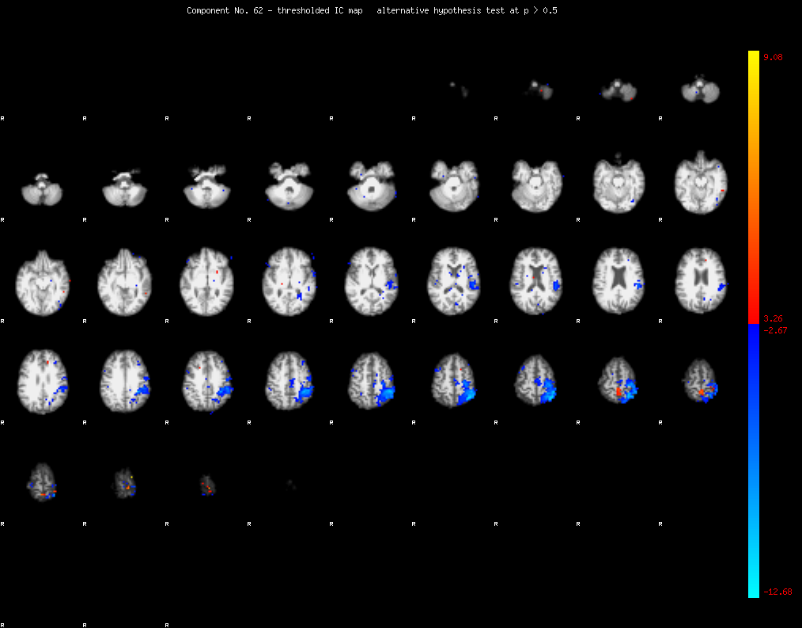

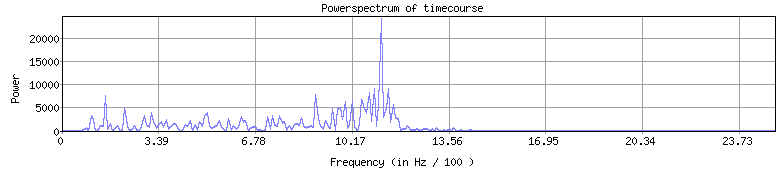

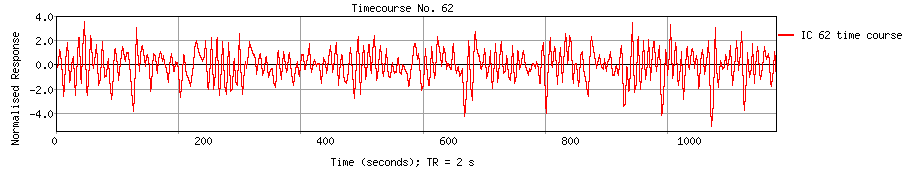
**

| zstat 11  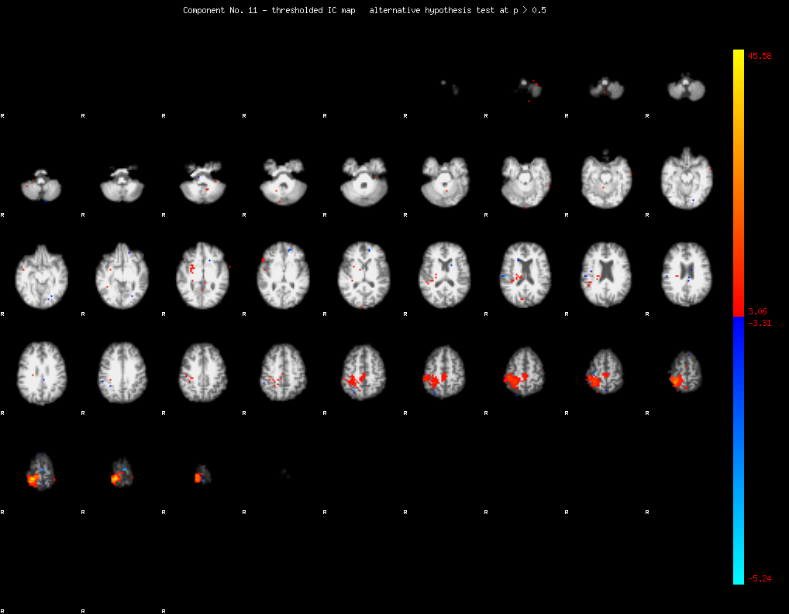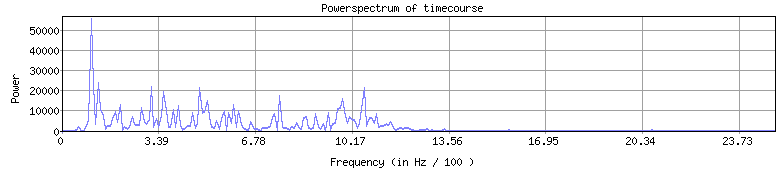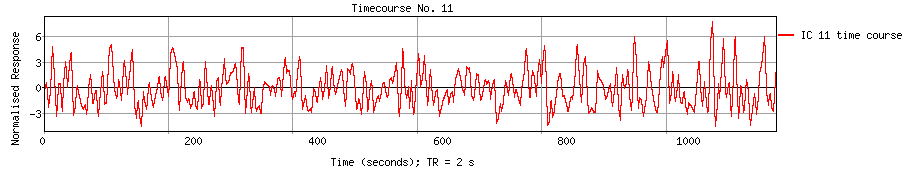 | |
| --- | --- |
|  |  |
|  |  |
| 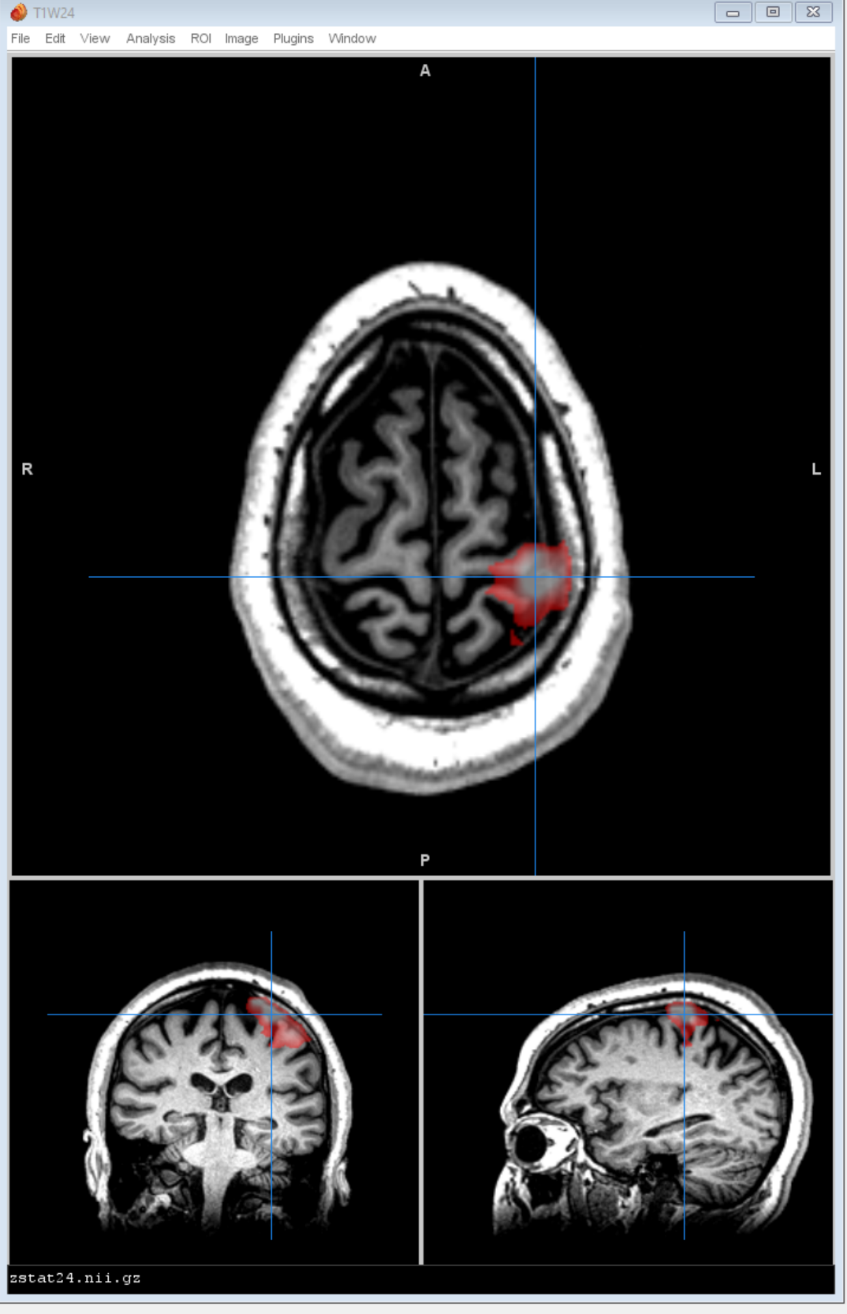 zstat 24 | 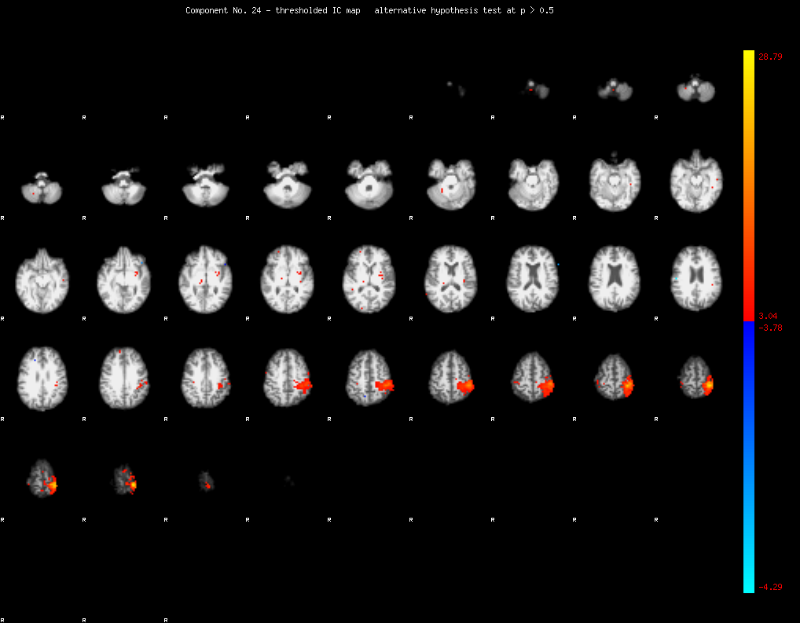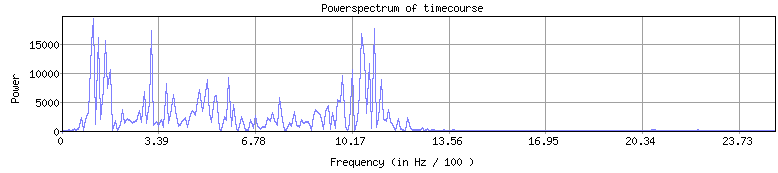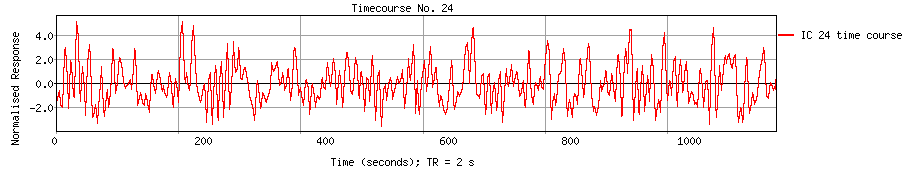 |

| **Language** |
| --- |

| zstat 72  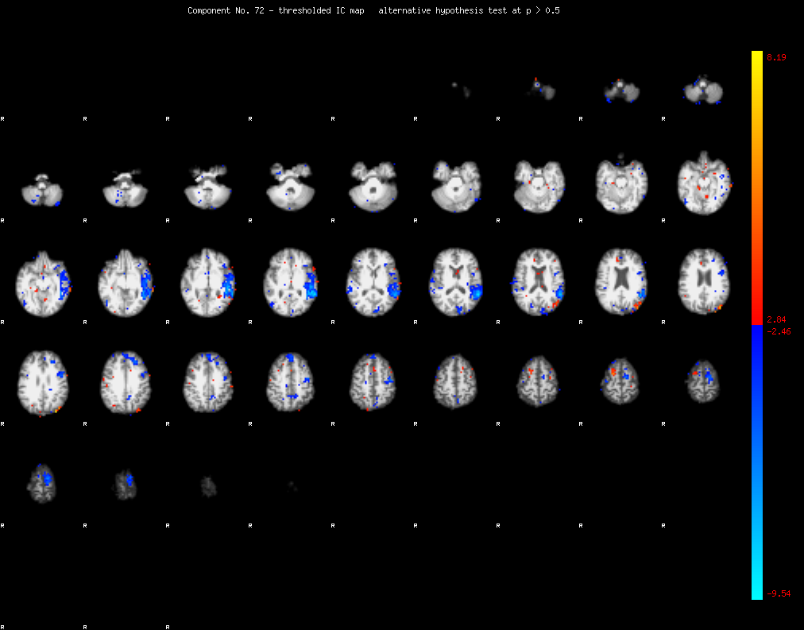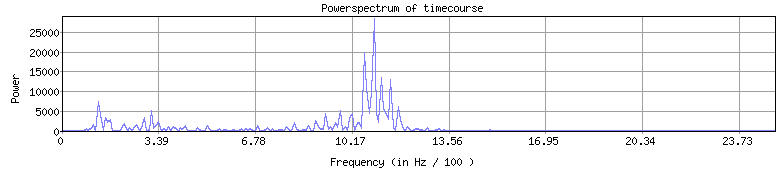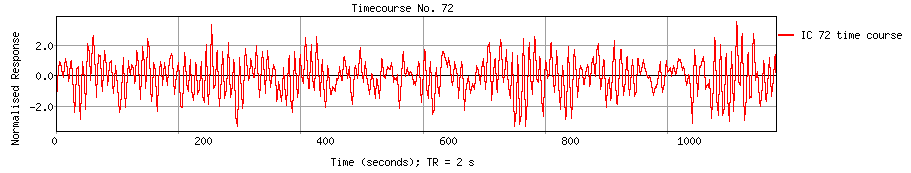 | |
| --- | --- |
|  |  |


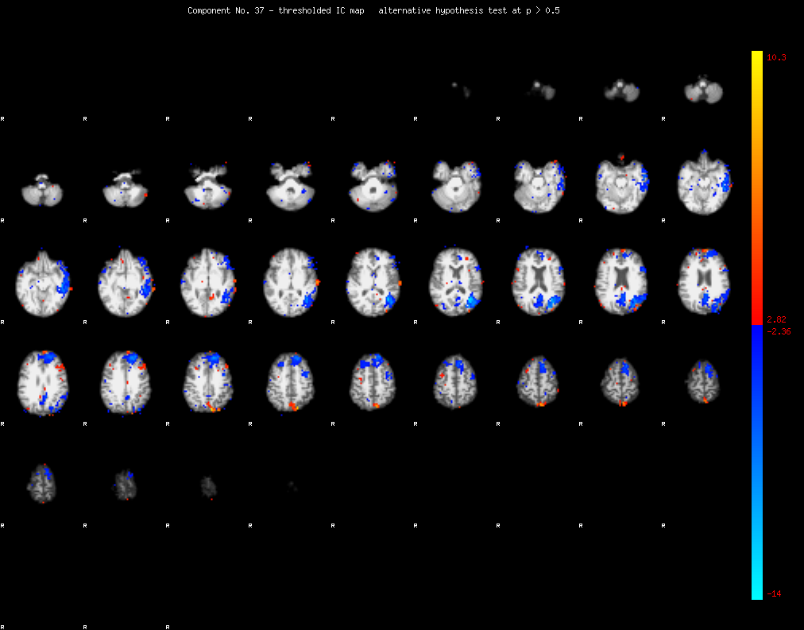

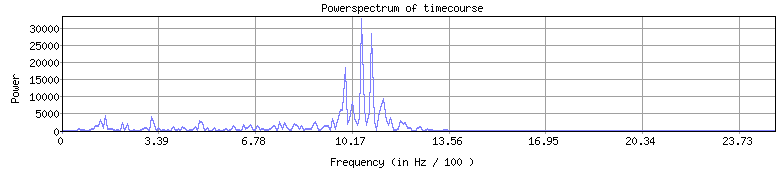

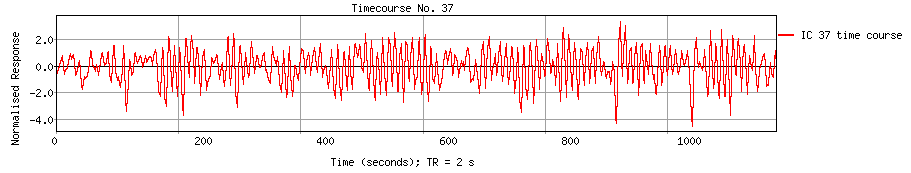


| 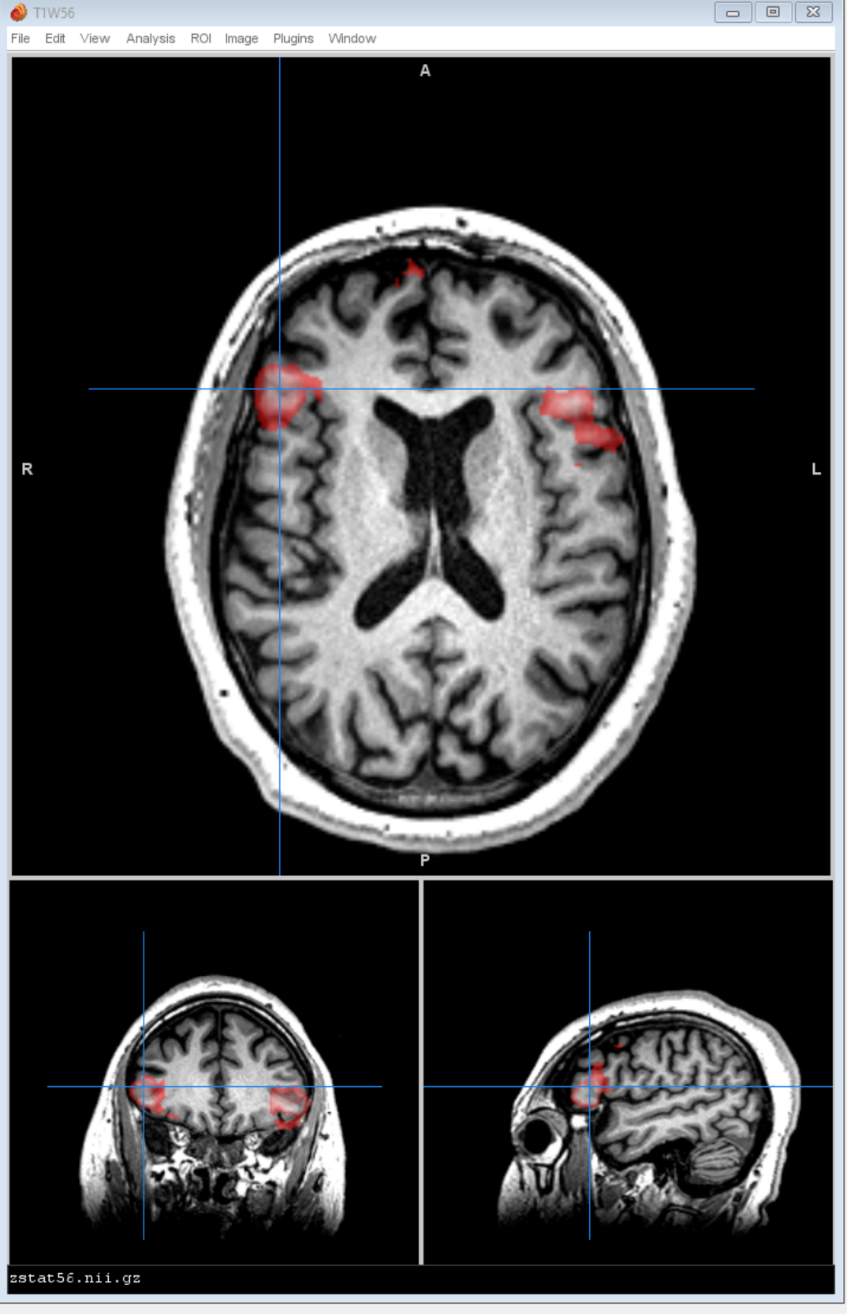 zstat 56 | 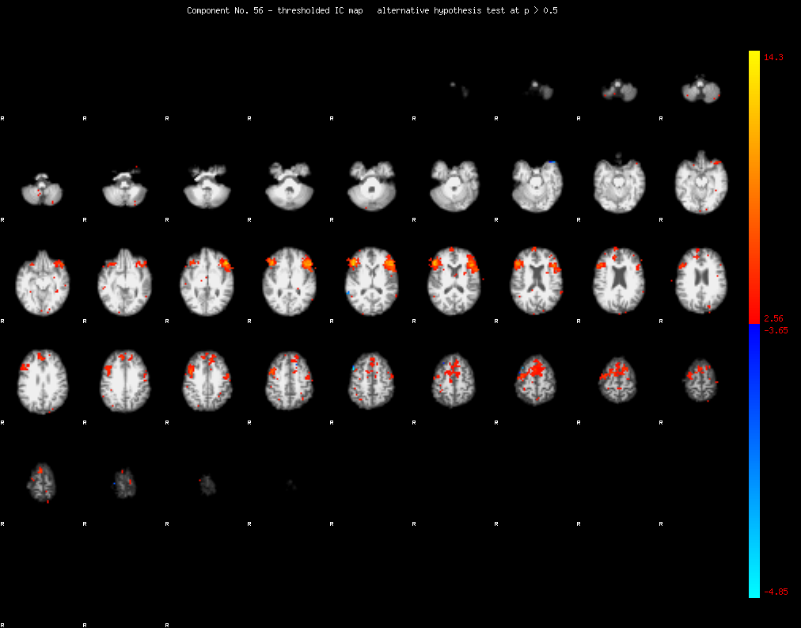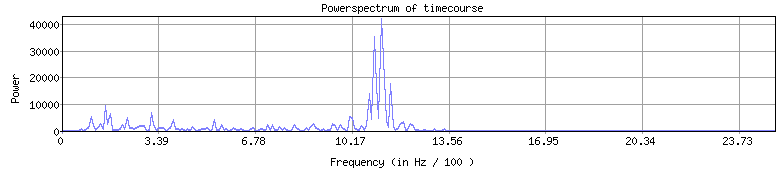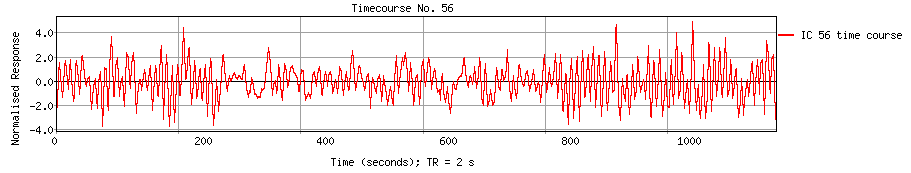 |
| --- | --- |

| 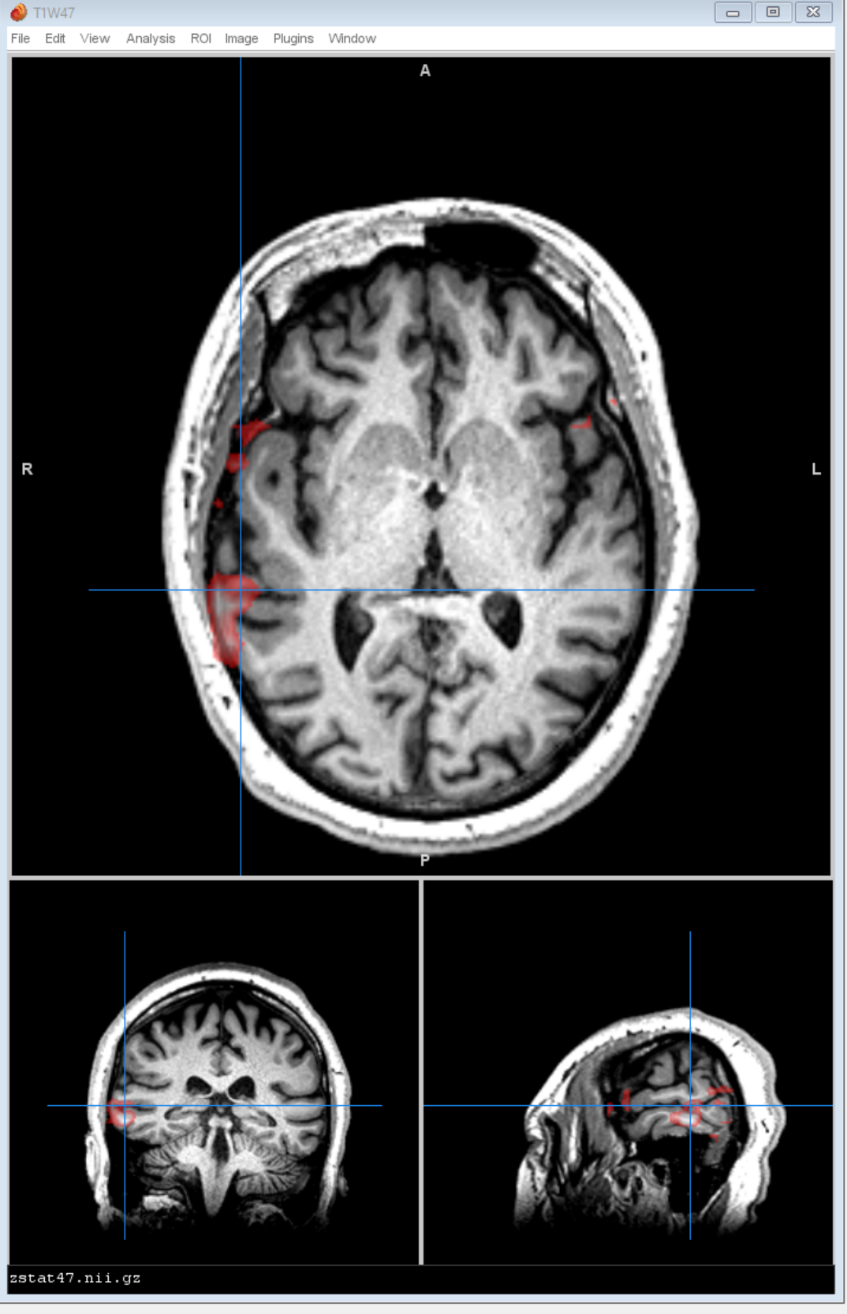 zstat 47 | 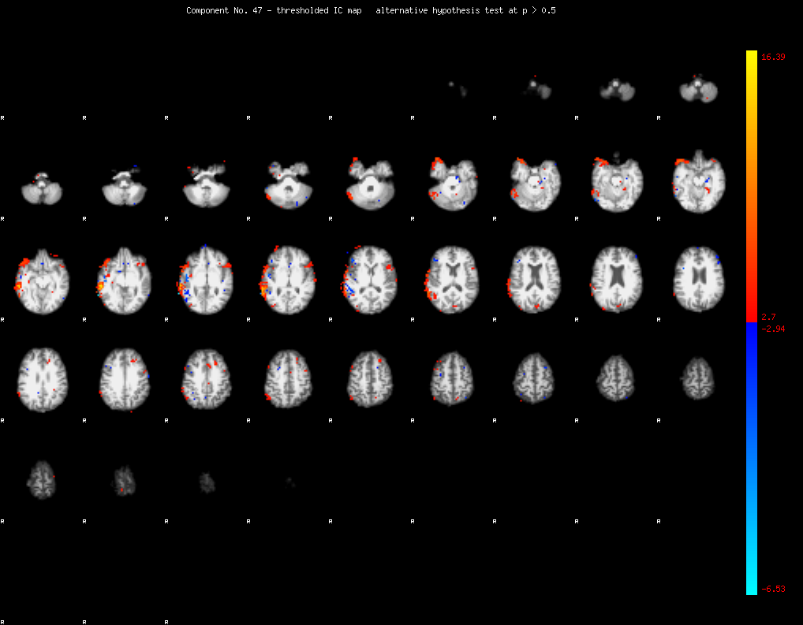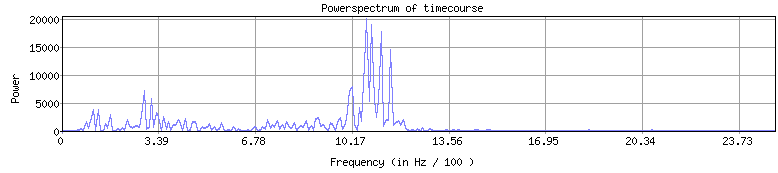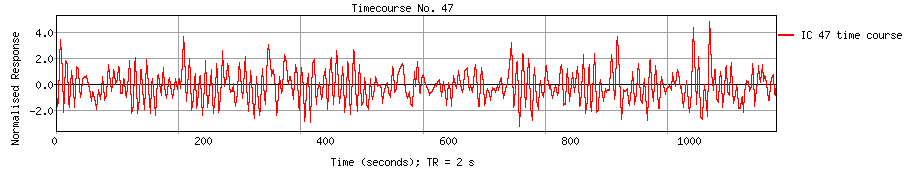 |
| --- | --- |

| 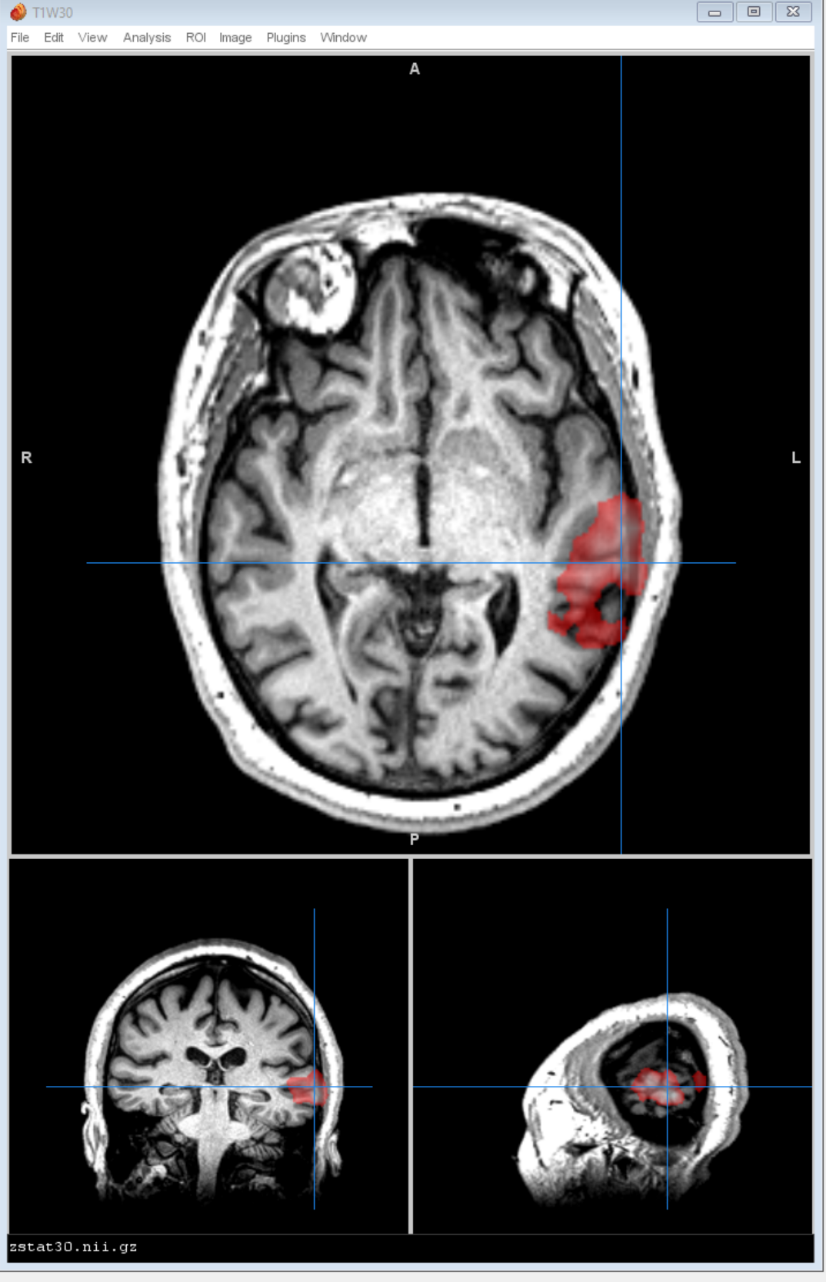 zstat 30 | 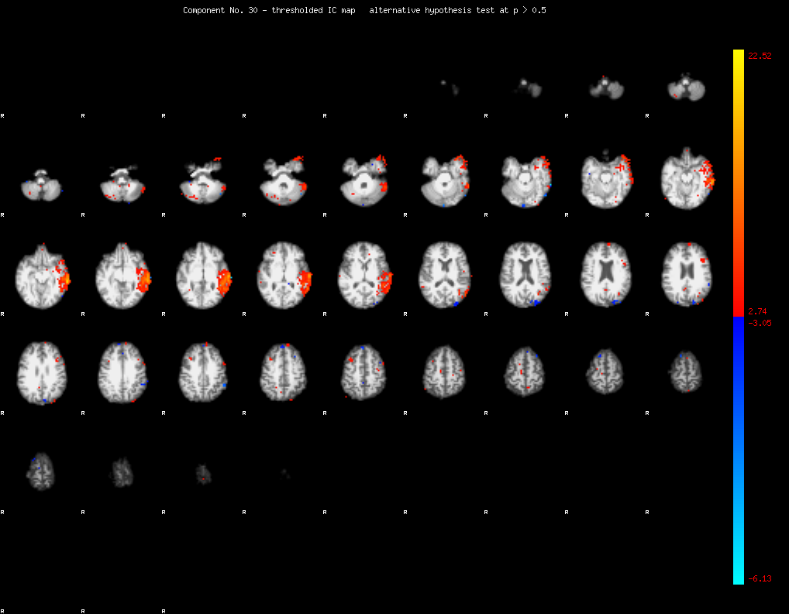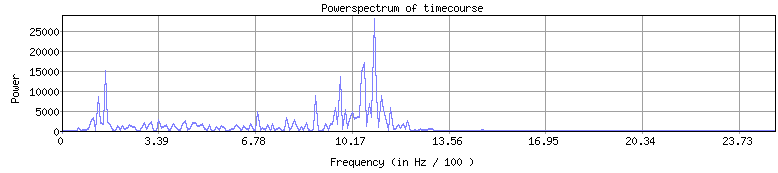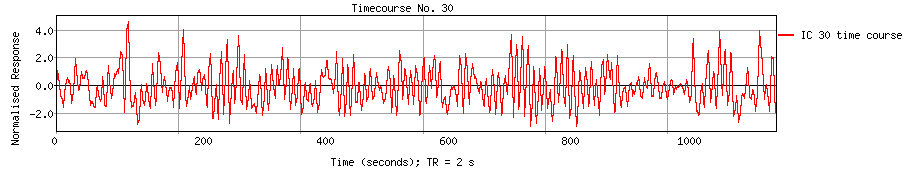 |
| --- | --- |

| 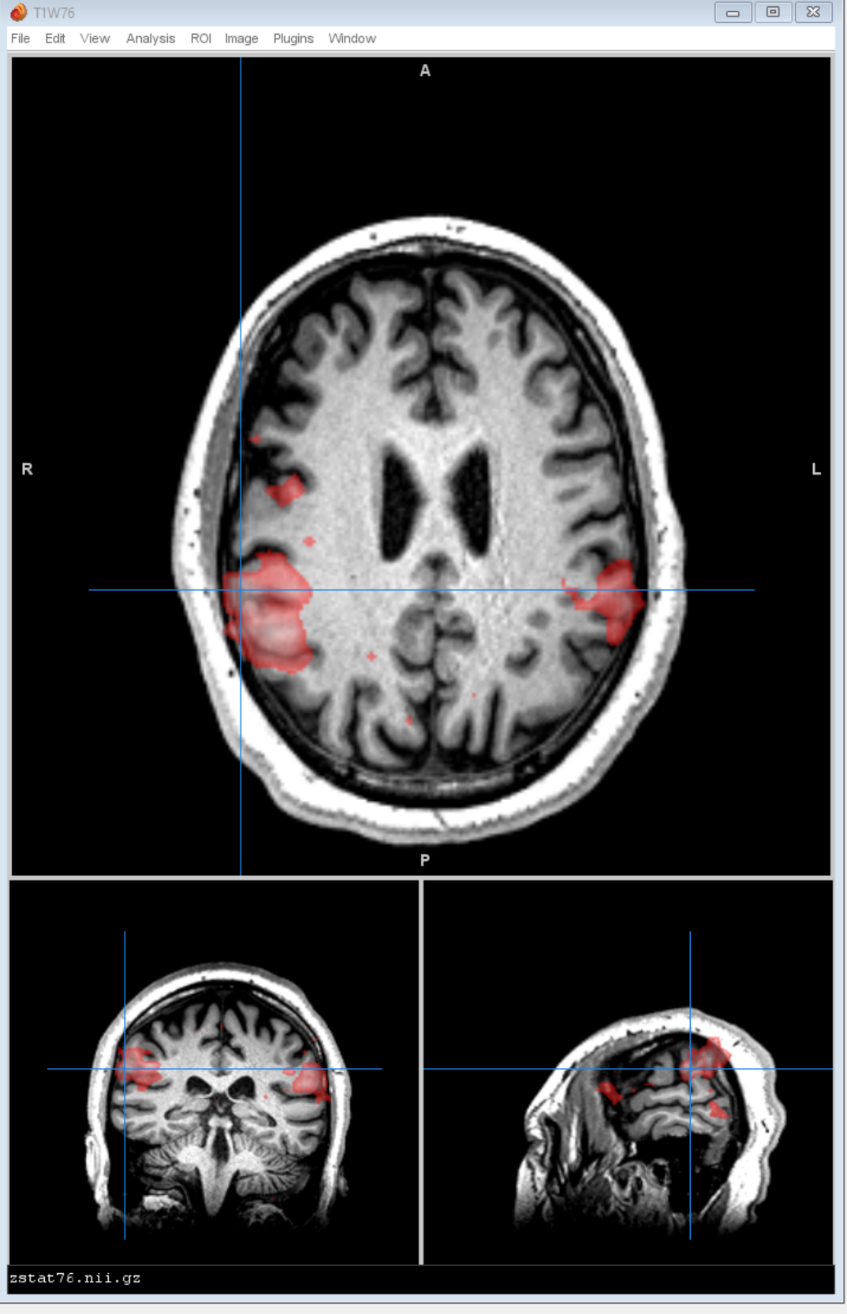 zstat 76 | 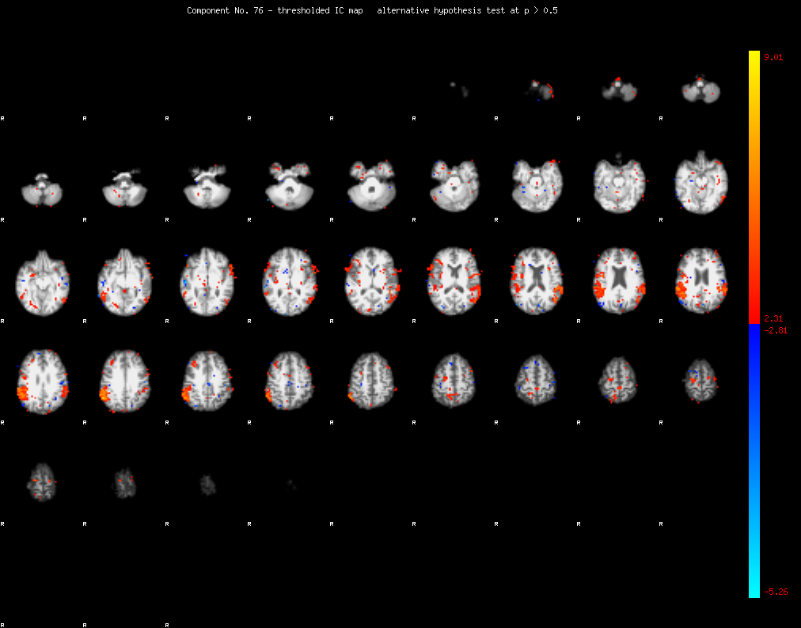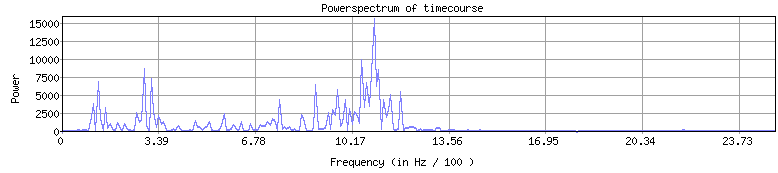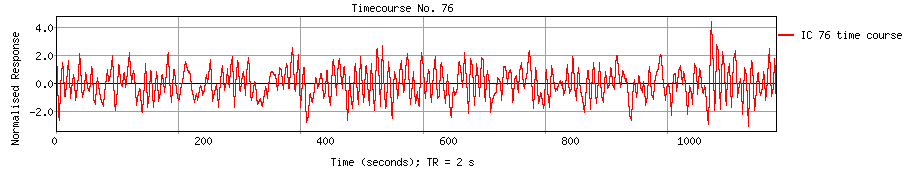 |
| --- | --- |

| **Parietal** | |
| --- | --- |
|  |  |
| 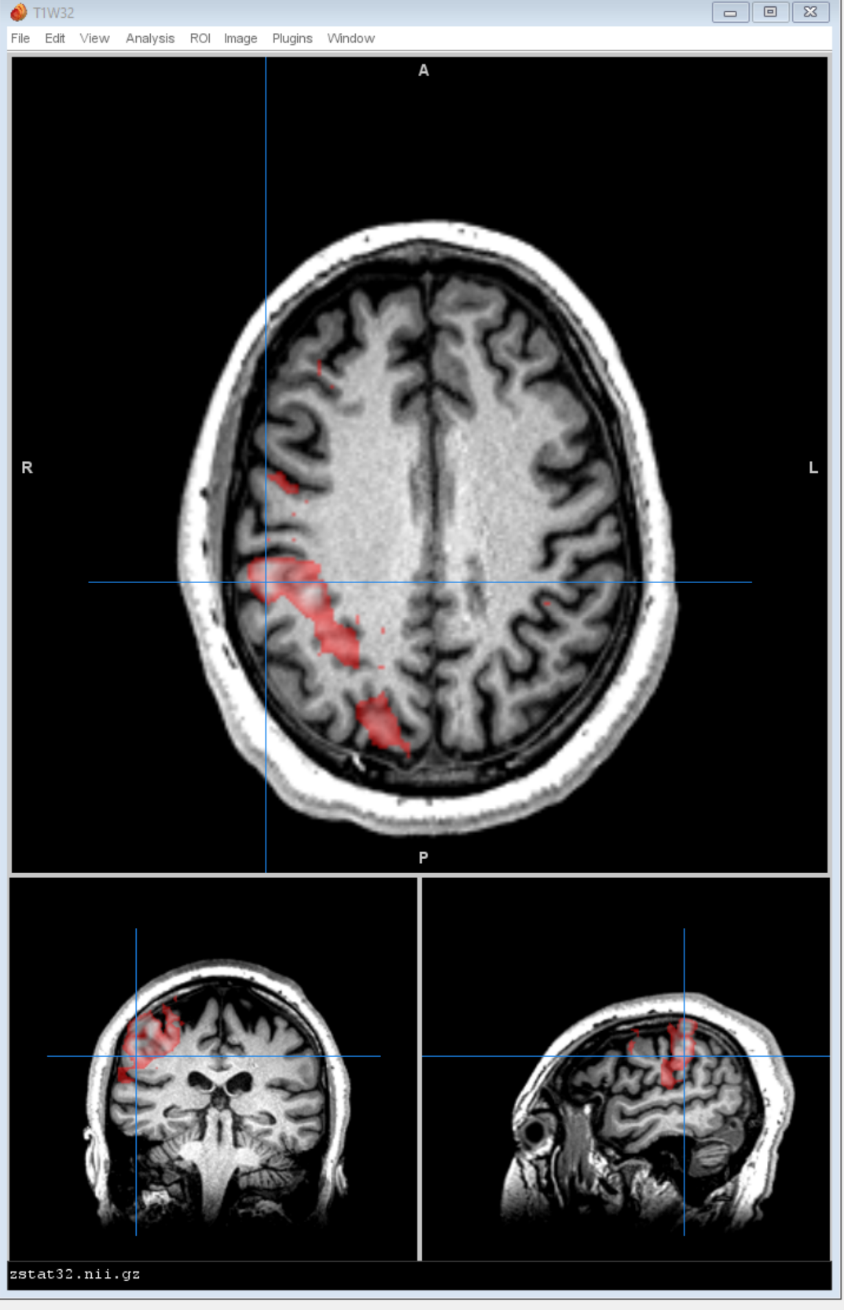 zstat 32 | 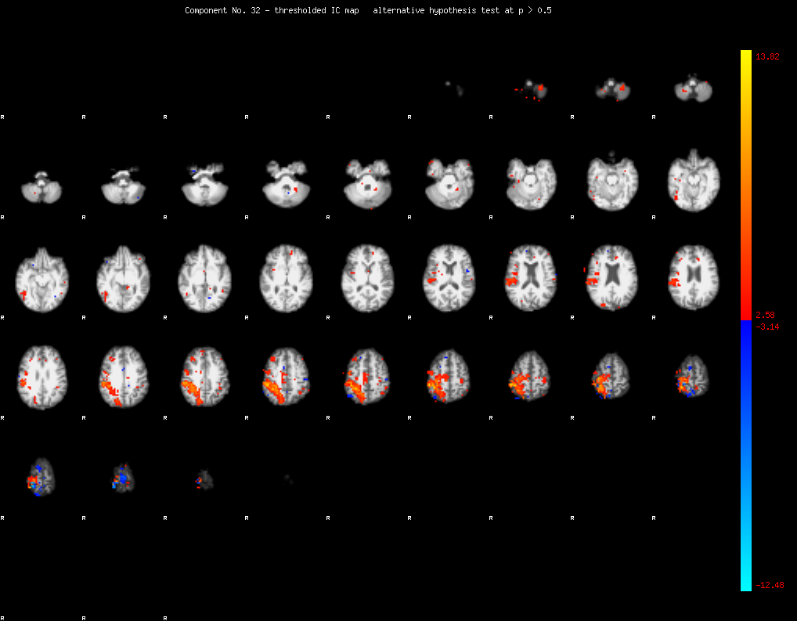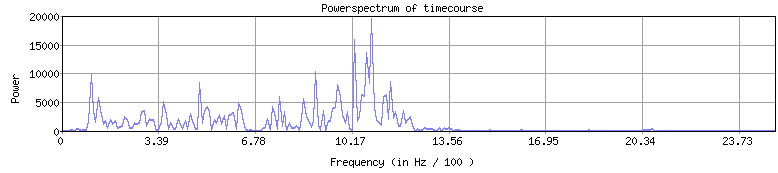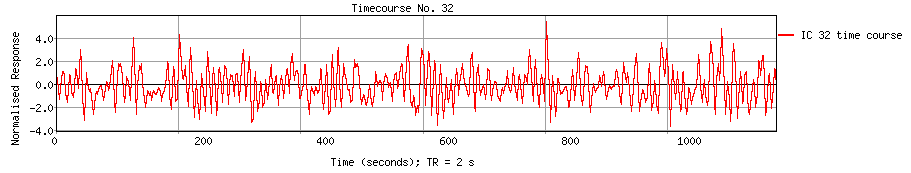 |
| 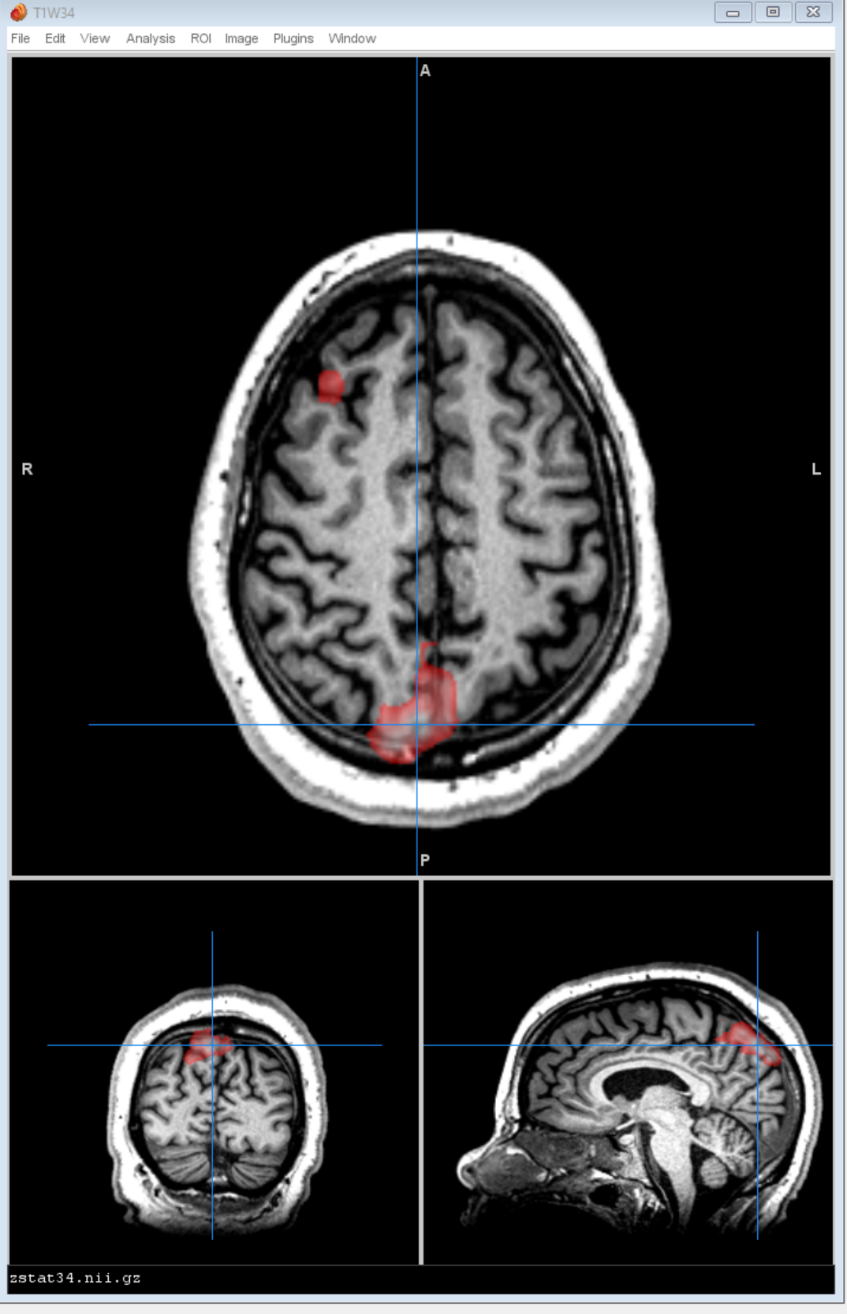 zstat 34 | 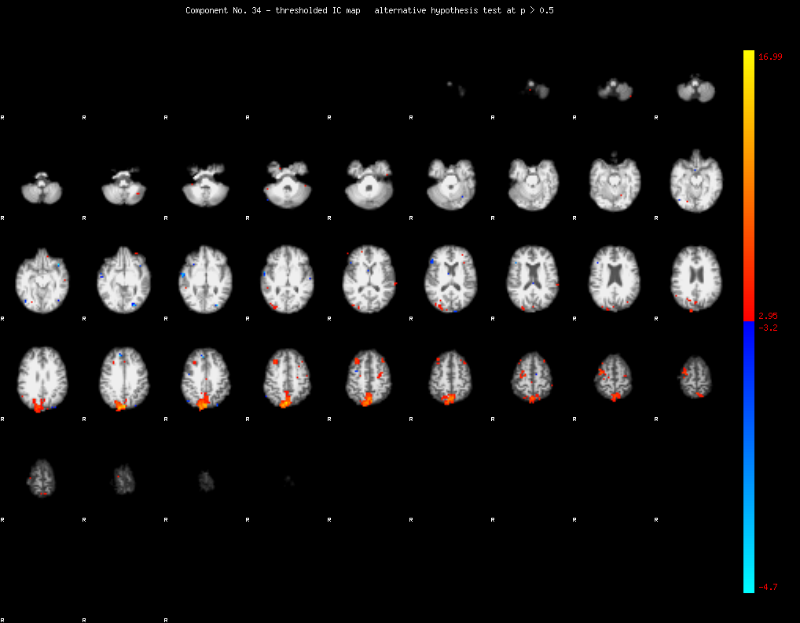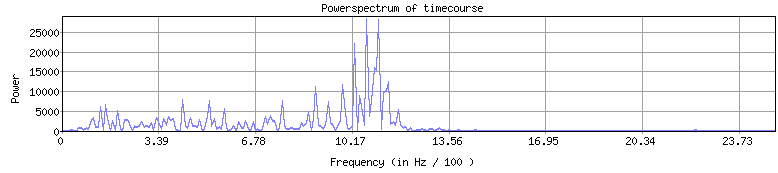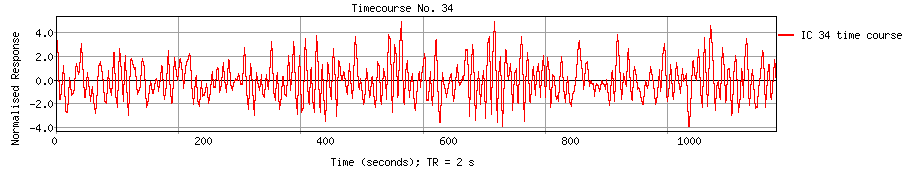 |
|  |  |

| zstat 55  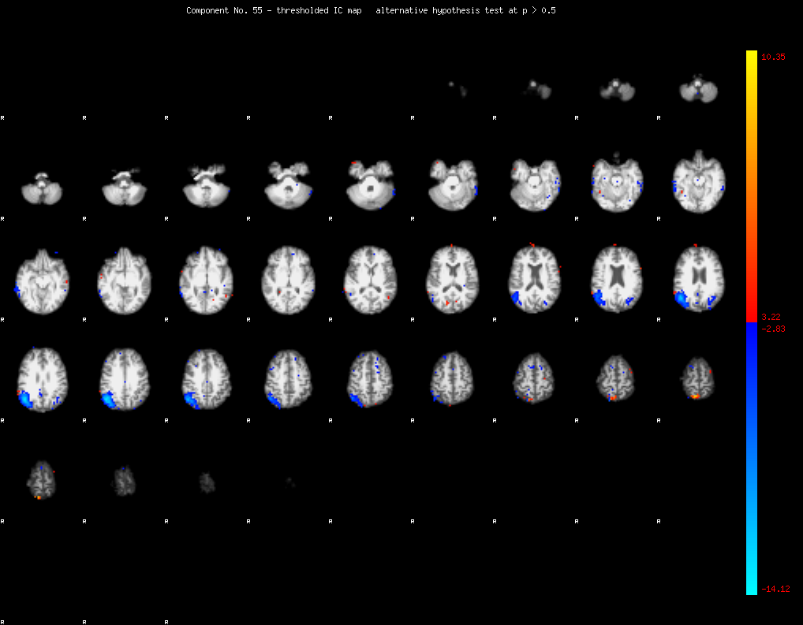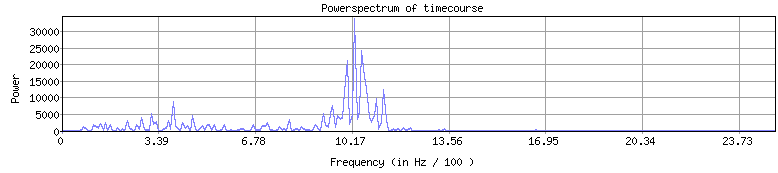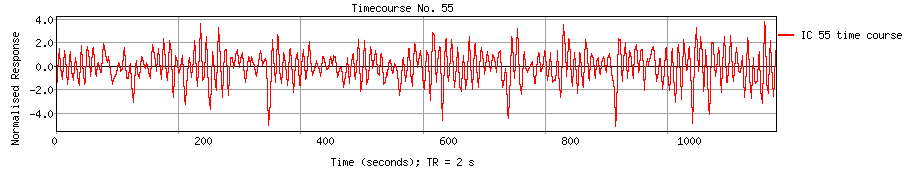 |
| --- |

| 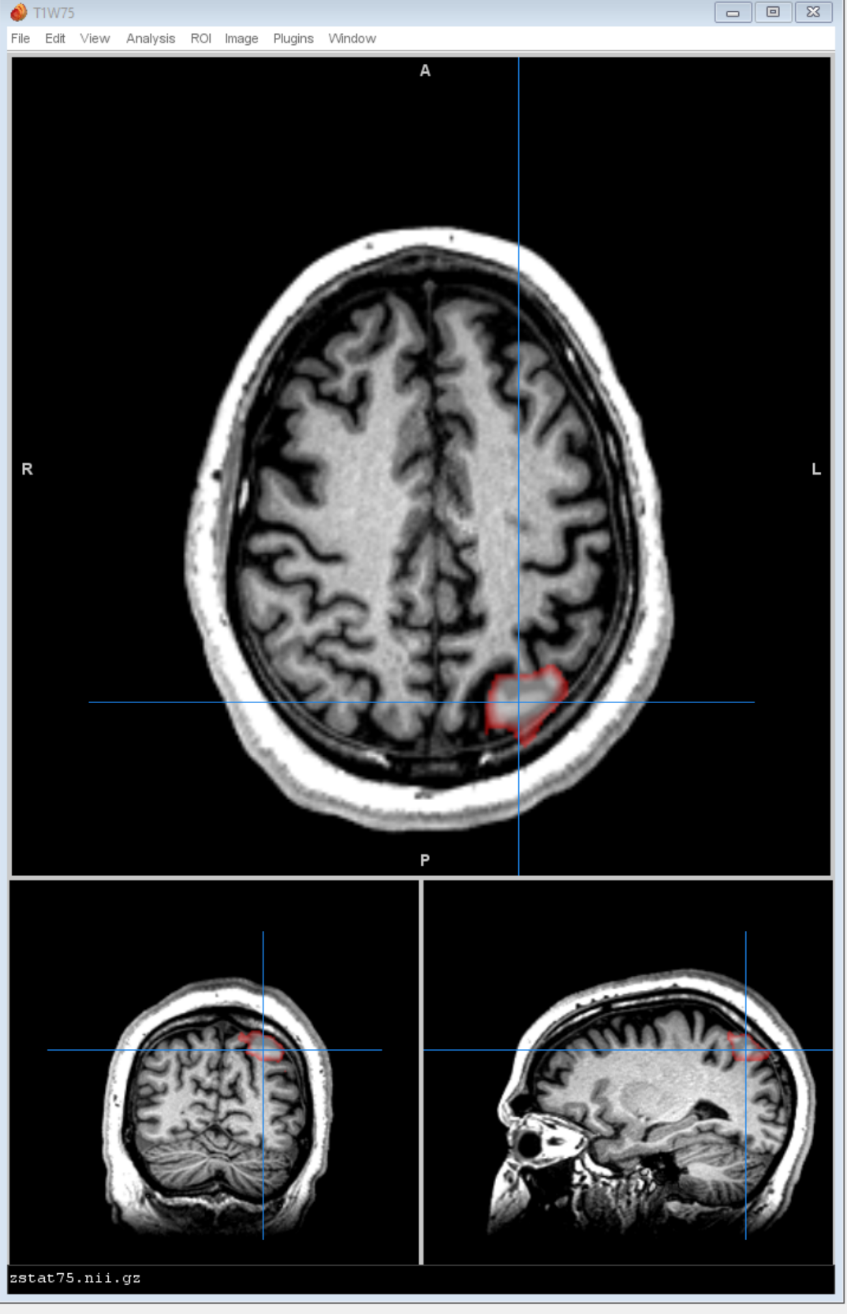 zstat 75 | 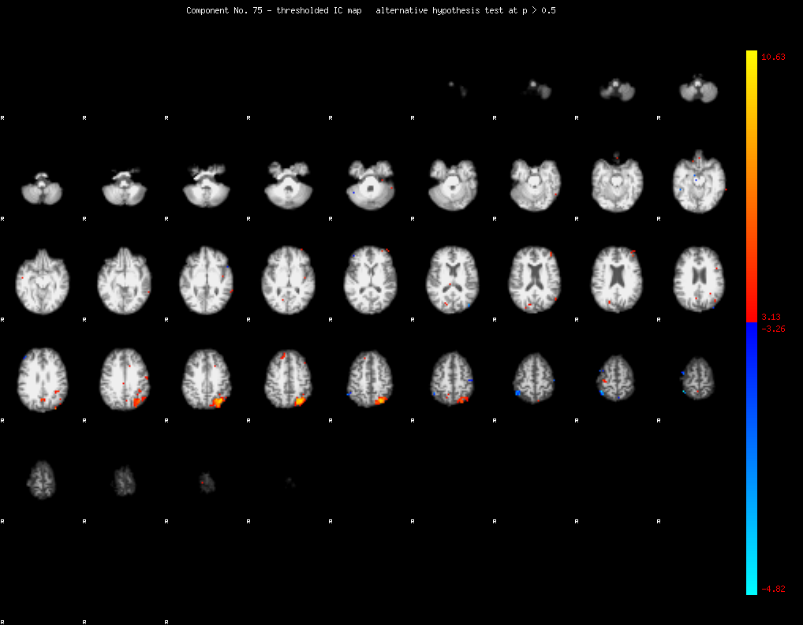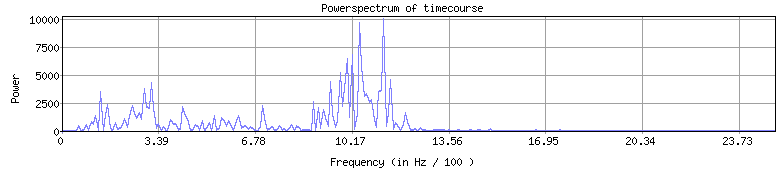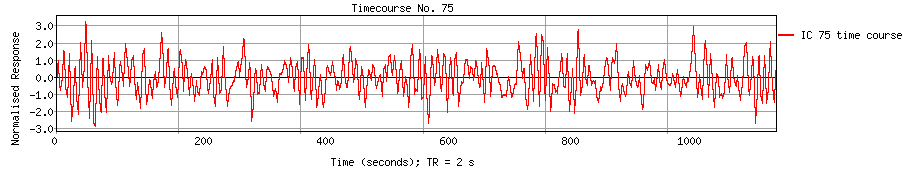 |
| --- | --- |

| **Frontal** | |
| --- | --- |
| 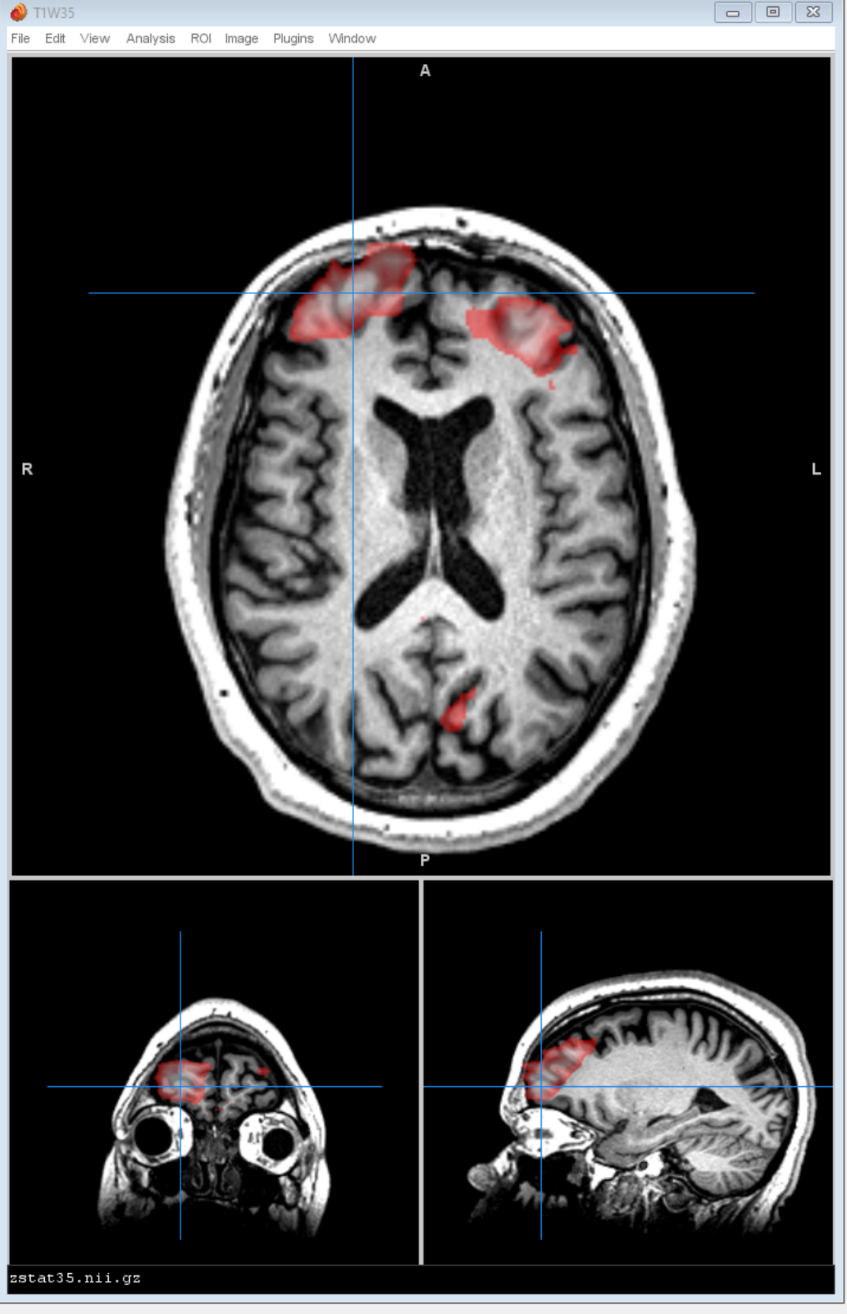 zstat 35 | 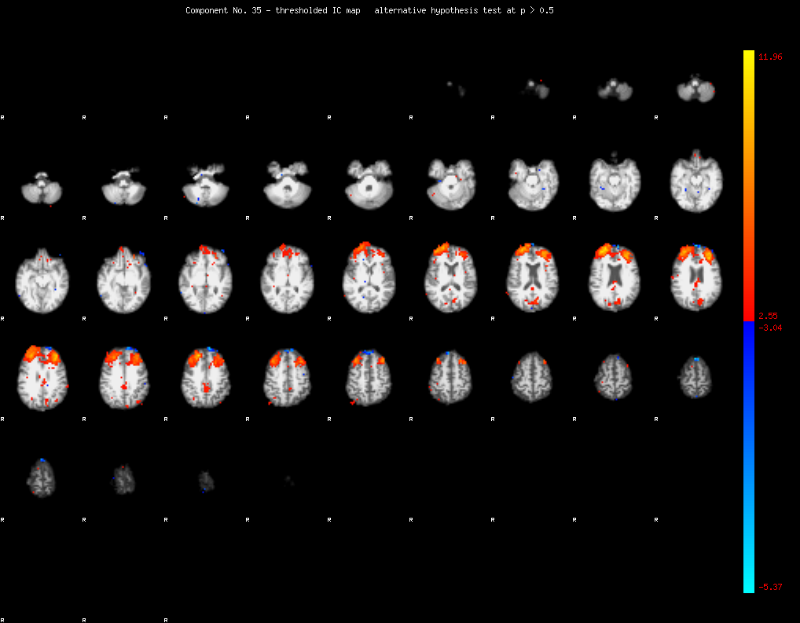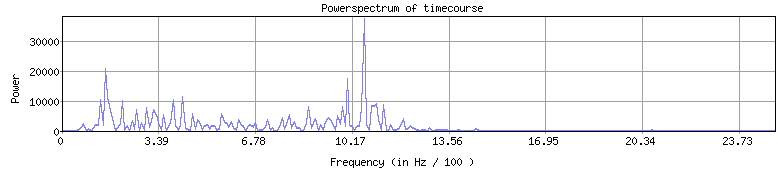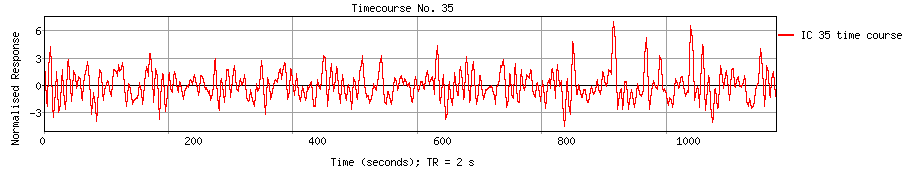 |
|  |  |

| 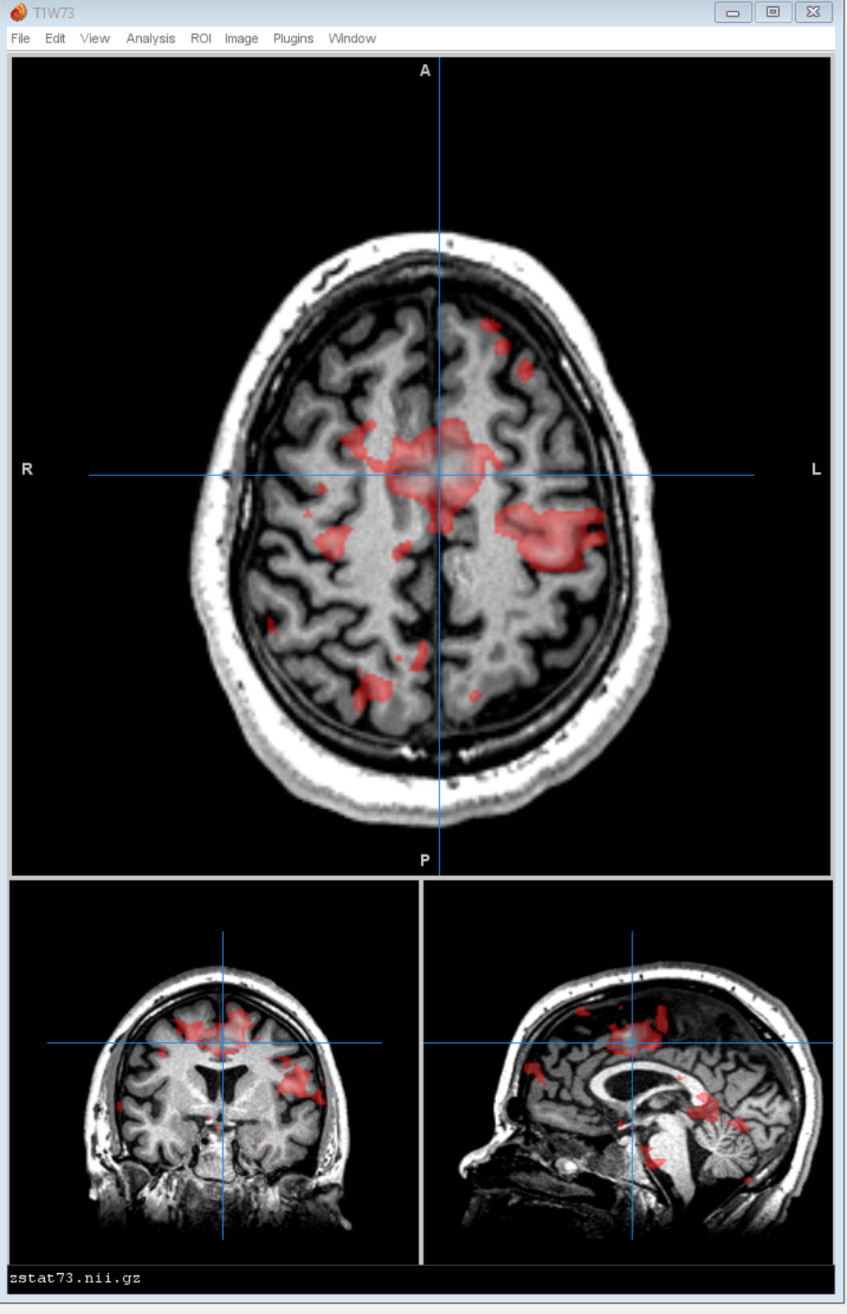 zstat 73 | 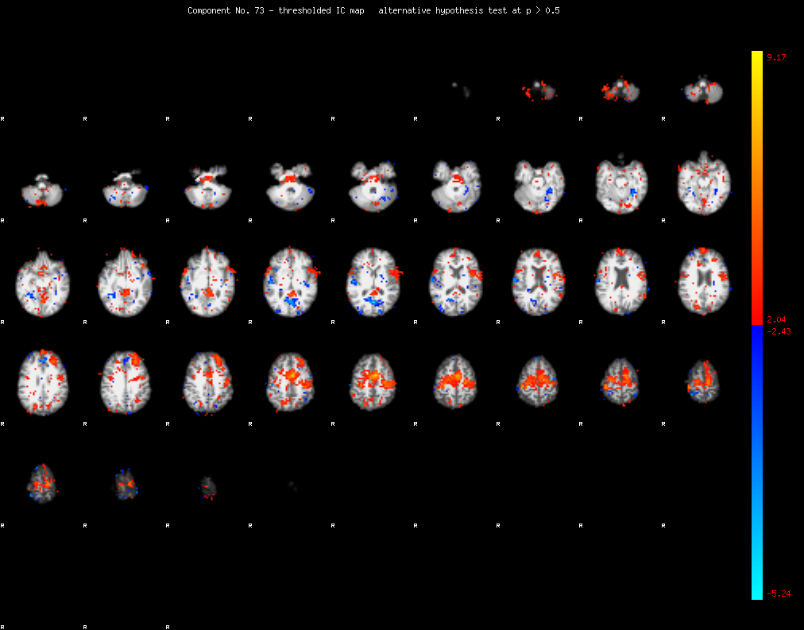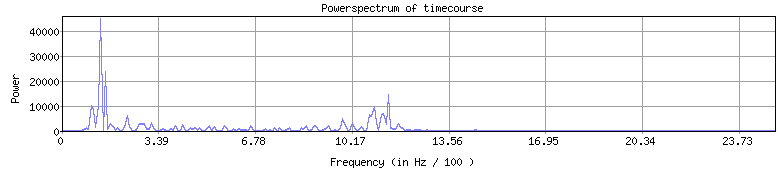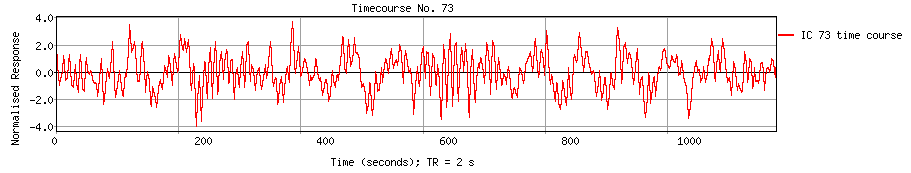 |
| --- | --- |

| zstat 28  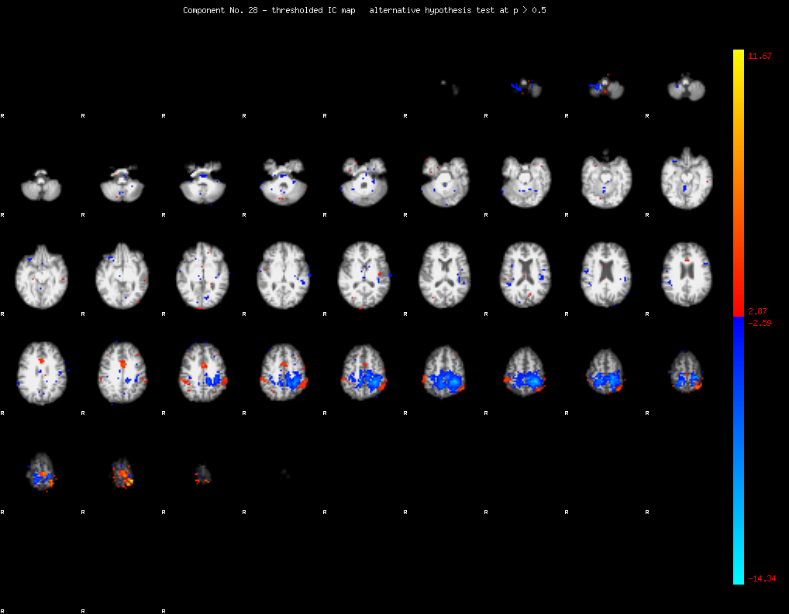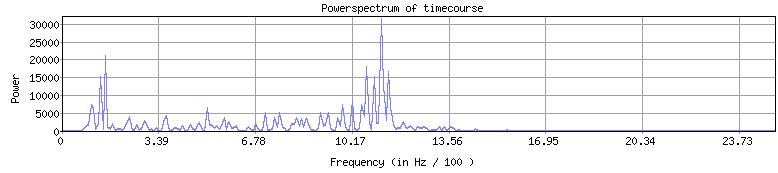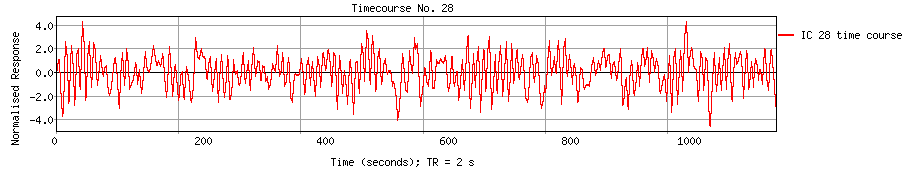 |
| --- |

| 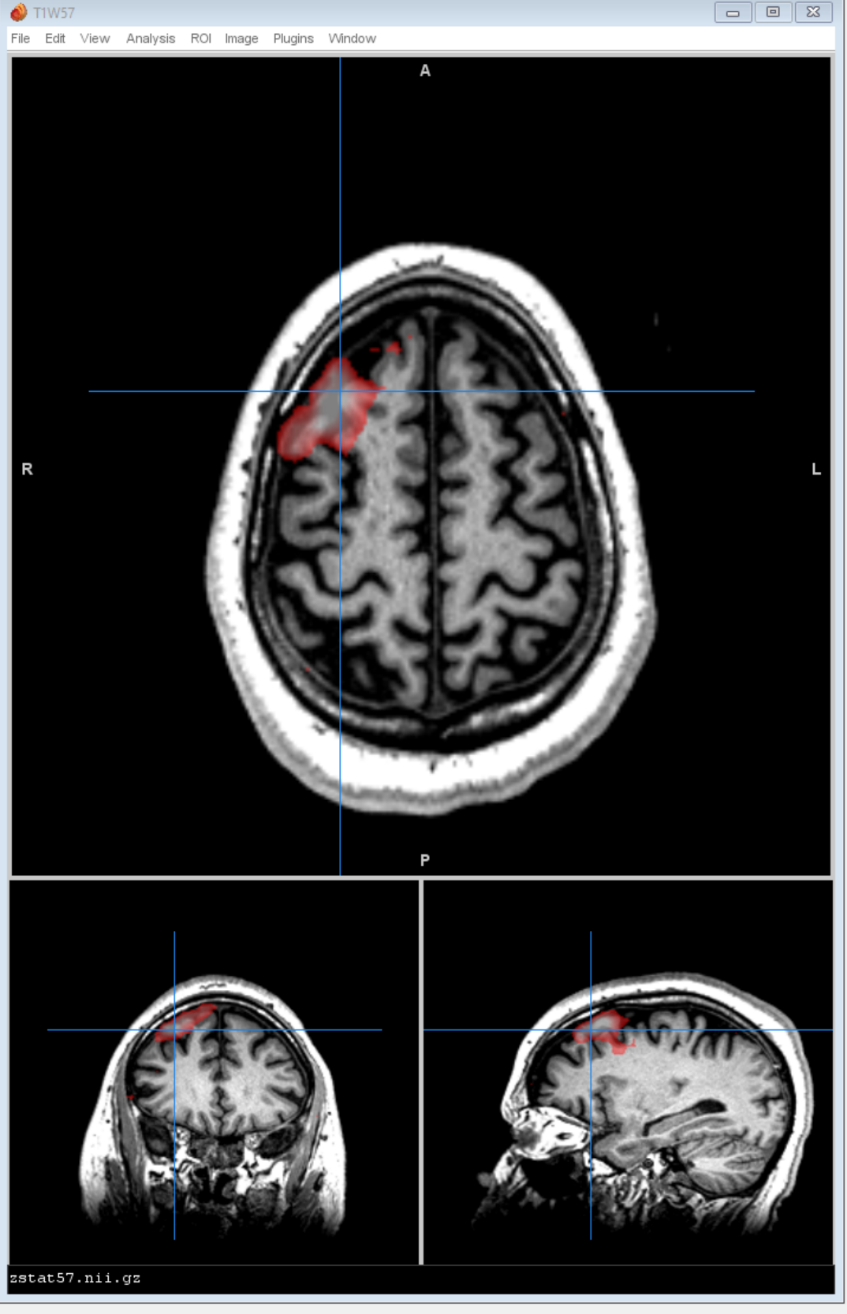 zstat 57 | 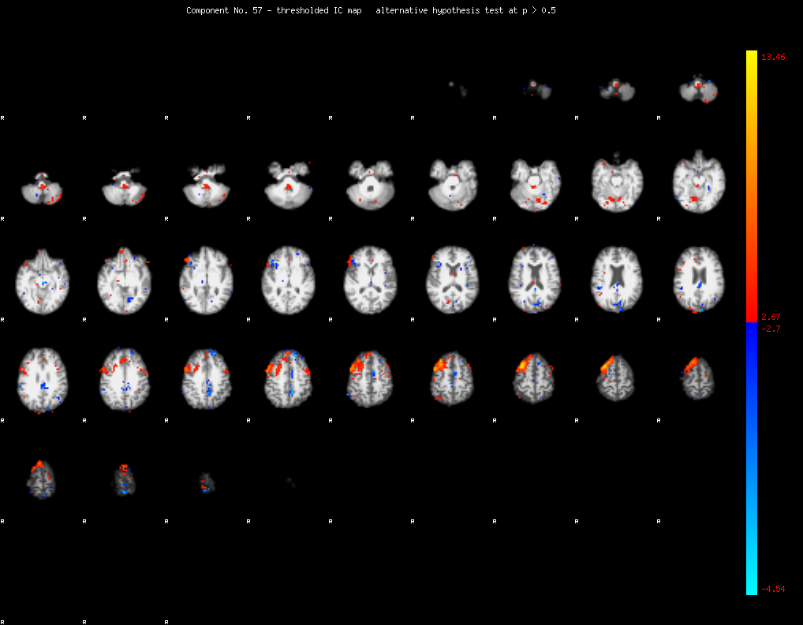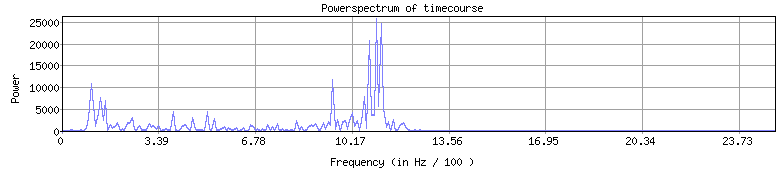 |
| --- | --- |

| **Temporal** | |
| --- | --- |
|  |  |
| zstat 68 |  |
|  |  |

| **Vision** | |
| --- | --- |
| zstat 50 | |
| zstat 25 |  |
|  |  |
| zstat 27 |  |
| zstat 39 |  |
| zstat 43 |  |

|  | |
| --- | --- |
| zstat 66 |  |
|  |  |
|  |  |
| zstat 80 |  |
|  |  |

| **Deep Grey** | |
| --- | --- |
| zstat 45 |  |

| zstat 58 |  |
| --- | --- |
|  |  |

| zstat 38 |  |
| --- | --- |

| **Modulating** | |
| --- | --- |
| zstat 59 |  |
|  |  |

| **Association: Long-range Fronto-Parietal** | |
| --- | --- |
| zstat 48 |  |

| zstat 50 |
| --- |

| zstat 74 | |
| --- | --- |
|  |  |

**Abbreviation Guide**

ACG Anterior Cingulate Gyrus

APF Anterior PreFrontal

AT anterior Temporal

B Bilateral

BG Basal Ganglia

DMN Default Mode Network

IFG Inferior Frontal Gyrus

IFS Inferior Frontal Sulcus

IPL Inferior Parietal Lobule

ITG Inferior Temporal Gyrus

L Left

LR-FTP Long Range Fronto-to-Parietal association network, anterior prefrontal IFG, MFG, posterior lateral parietal S2

LS2 – lateral Secondary Sensory area

MFG Middle Frontal Gyrus

MT mesial Temporal

MTG Middle Temporal Gyrus

mS2 – medial Secondary Sensory area

OPC- Operculum

OTG Occipito-temporal gyrus

PCG Posterior Cingulate Gyrus

PFC Prefrontal Cortex

PMC Premotor Cortex

PHG Parahippocampal Gyrus

POS Parietal Occipital Sulcus

R Right

Rs-fMRI Resting state functional MRI

RS Resting State

RSN Resting state network – an expected brain network

S1 Primary somatosensory cortex

S2 Secondary somatosensory cortex or secondary sensory association area

SFG Superior Frontal Gyrus

SFS Superior Frontal Sulcus

SMA Supplementary Motor Association area

SMG Supramarginal Gyrus

SOZ Seizure onset zone

SPL Superior Parietal Lobule

STG Superior Temporal Gyrus

STS Superior Temporal Sulcus

TOJ – Temporal Occipital Junction network

V1 Primary visual cortex associated network

V2 Secondary visual cortex associated network

vmPFC ventral medial prefrontal cortex
