# Supplementary material for: Pre- and post-therapy functional MRI connectivity in severe acute brain injury with suppression of consciousness: a comparative analysis to epilepsy features
Source: Front Neuroimaging. 2024 Oct 1;3:1445952. doi: 10.3389/fnimg.2024.1445952 (PMC11473429; doi:10.3389/fnimg.2024.1445952)
Supplement: Supplementary file 10 [file Table_10.DOCX]

Resting State functional MRI Whole Brain Map

| **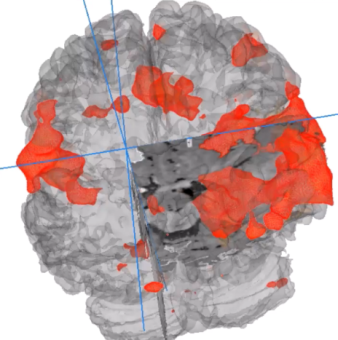** |  |  |
| --- | --- | --- |

PP1 MRI#2

Anatomical MRI - see separate radiological report,

Technique and analysis Methods 3T MRI, whole brain BOLD ICA

Data Quality Analysis 20 min; Head motion < 1 mm, no interfering artifacts detected

Abbreviation index list at end of rs-report

Impression:

1. Task-fMRI – no activation upon command to move arm or repeat words.
2. **Rs-fMRI seizure onset zone**: R > L Opc region
3. **Language**: well detected
4. **Whole brain networks**: Overall, improved from the most recent study about 1 month prior in that the degree and number of atypical networks in fewer AND the RSNs have reduced atypically increased frequency overall. Explaining continued reduced level of consciousness are: (1) The cognitive networks – which are the long-range fronto-parietal networks were detected in a R > L spatial distribution – HOWEVER the frequency was increased in these RSN and appear to be receiving downstream atypical signal from the R > L SOZ networks, thus reducing normally of the functional connectivity of these cognitive networks. (2) Further there remains atypical connectivity of the midbrain-diencephalon region. Combined, these can reduce consciousness. On the positive note the default mode network and the left lanague networks are stone cold normal, portending good potential for continued improvement in level of consciousness with some ability to communicate with language.

| Rs-fMRI Informed Neuro-Prognostication | | |
| --- | --- | --- |
| Consciousness | Alertness and Awareness  Equivalent of 0=Coma, 1=Normal Consciousness, 2=MCS, 3=VS/UWS | 1 |
| Developmental Stream | 0=very poor, 1=Normal, 2=Atypical but not very poor, 3=Very poor, 4=Indeterminate | |
| Motor | Walking | 1 |
|  | Gross motor body movement | 1 |
|  | L - arm/hand fine motor | 1 |
|  | R- arm/hand fine motor | 1 |
|  | face/mouth motor coordination | 1 |
| Motor tone | R appendicular tone | 1 |
|  | L appendicular tone | 1 |
|  | Central tone | 1 |
| Language | Understanding words | 1 |
|  | Speaking in Words | 1 |
| Vision | R field primary visual reception | 1 |
|  | R field higher level visual stimuli interpretation | 1 |
|  | L field primary visual reception | 1 |
|  | L field higher level visual stimuli interpretation | 1 |
| Sensory | Touch/Sense of Spatial/complex relationship | 1 |
| Cognition/Learning/Memory | equivalent IQ >70 | 2* see LR-FTP note |
|  | equivalent IQ < 70 |  |
|  | Profound Intellectual Disability Equivalent IQ < 35 |  |

| Network Characterization | | | | |
| --- | --- | --- | --- | --- |
| 1=yes, 0=no | Detected | Normal | Atypical | Comment |
| Motor | 1 | 1 |  | Normal homologous RSNs detected |
| Language | 1 | 1 |  | L STG and IFG in same network, normal |
| Parietal | 1 | 1 |  | Normal homologous RSNs detected |
| Frontal | 1 | 1 |  | Normal homologous RSNs detected |
| Temporal | 1 | 1 |  | Normal homologous RSNs detected |
| Vision | 1 | 1 |  | Normal homologous RSNs detected |
| Deep grey | 1 | 1* |  | Normal homologous RSNs detected**see below |
| Modulating | 1 | 1 |  | Normal homologous RSNs detected |
| Association, LR-FTP | 1 | 2 |  | R w deactivation and inc freq AND R > L long range association network spatial activity; these are the LR-FTP |
| Atypical/possible pathological | (23) R Opc, alternating activation-deactivation pattern, inc freq  (10) B Opc inc freq  (36) R > L Opc region, inc freq  (33) R > L Opc region, inc freq  (43) midbrain, inf midline diencephalon deactivation, inc freq  *(41) RSN Cerebellum inc freq | | | |

* Scoring unless otherwise specified is shown as the possible highest score expected or range

| **Atypical Networks** |
| --- |
| zstat 23  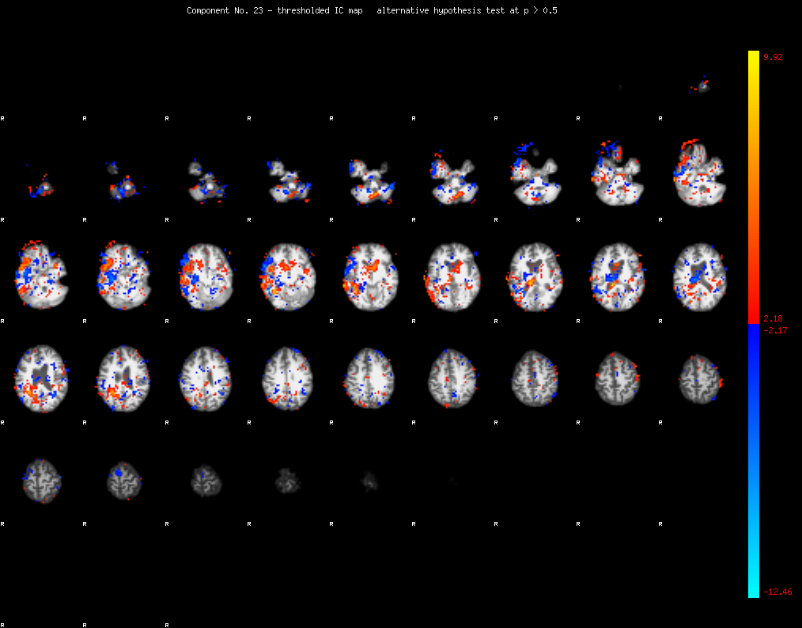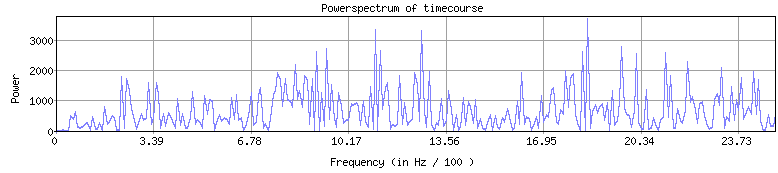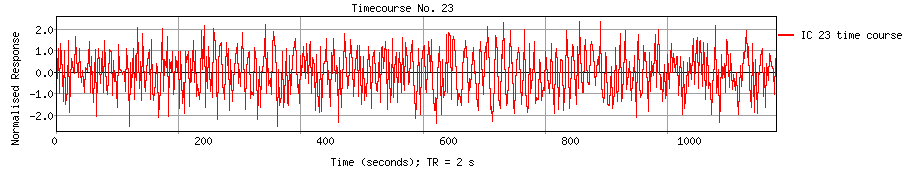 |

| zstat 10  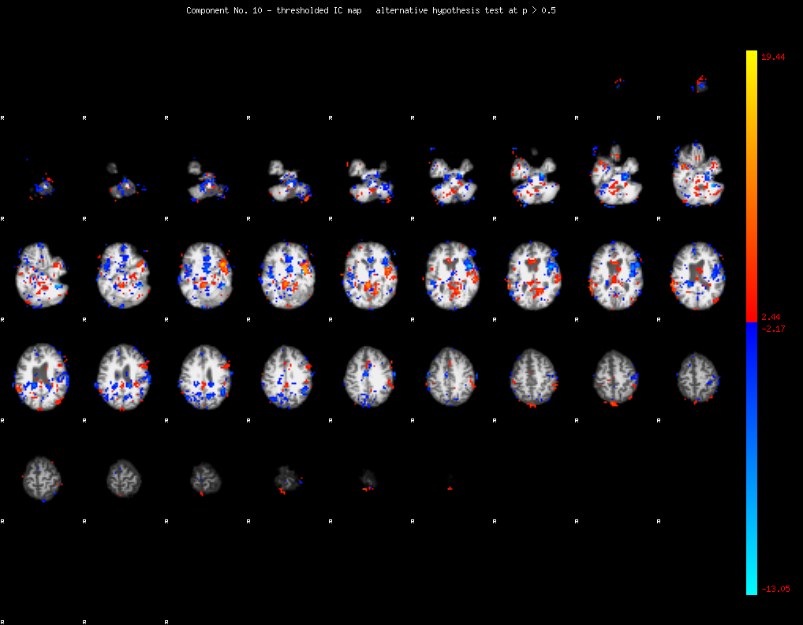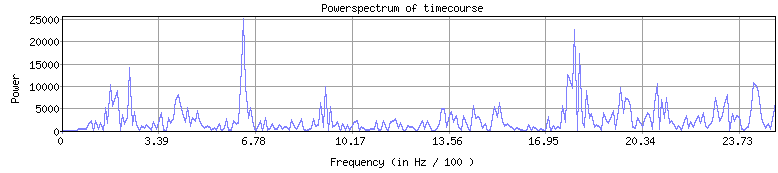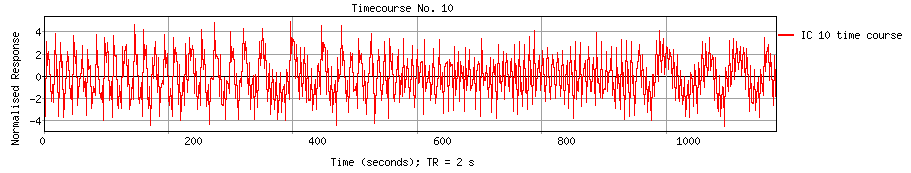 |
| --- |

| zstat 36  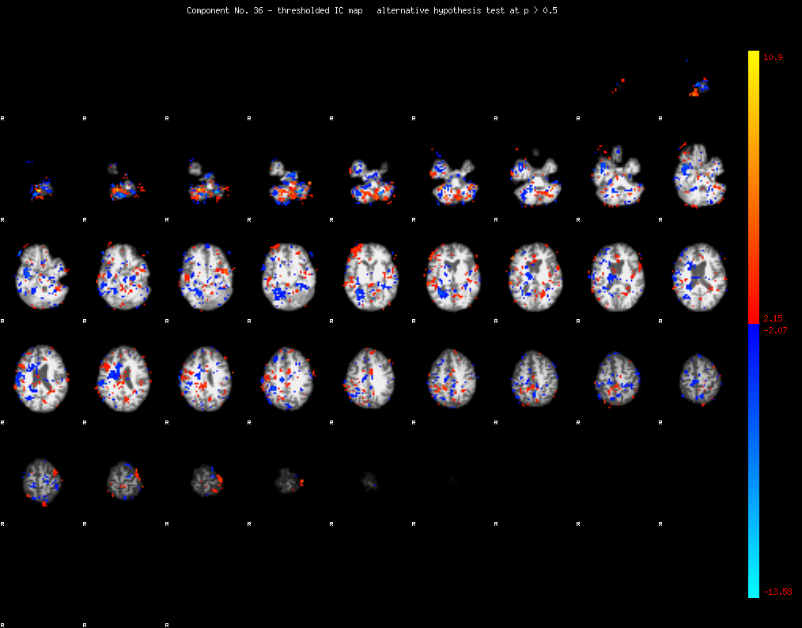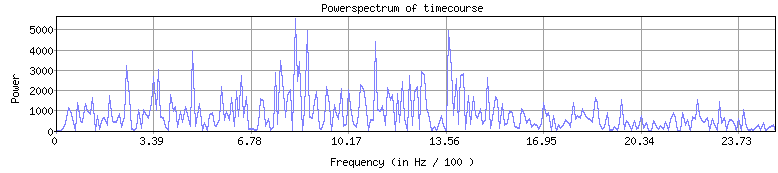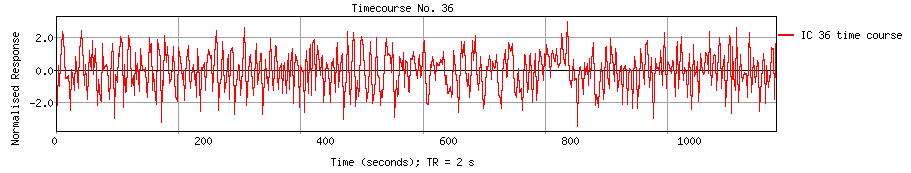 | |
| --- | --- |
|  |  |
|  |  |

| zstat 33  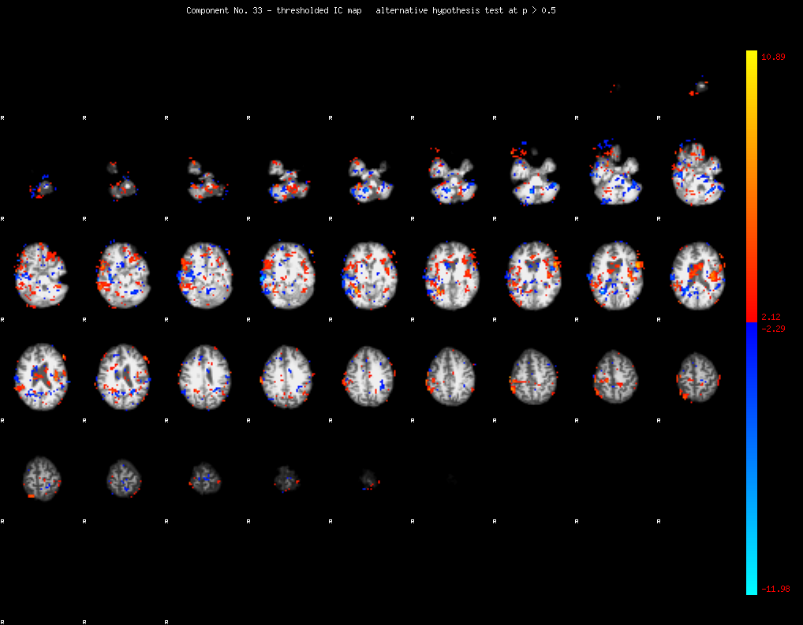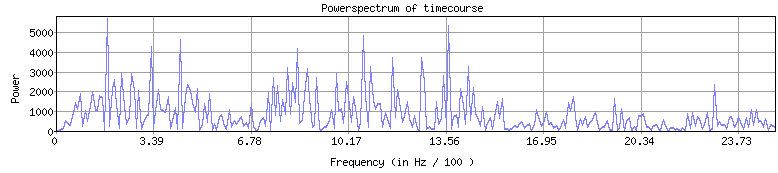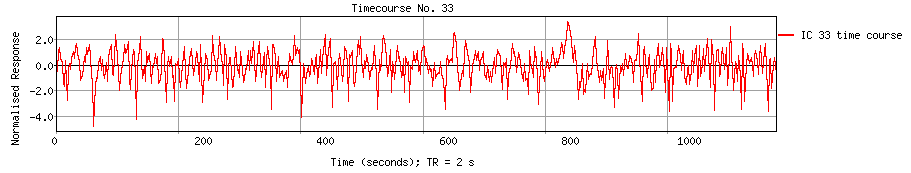 | |
| --- | --- |
|  |  |

| 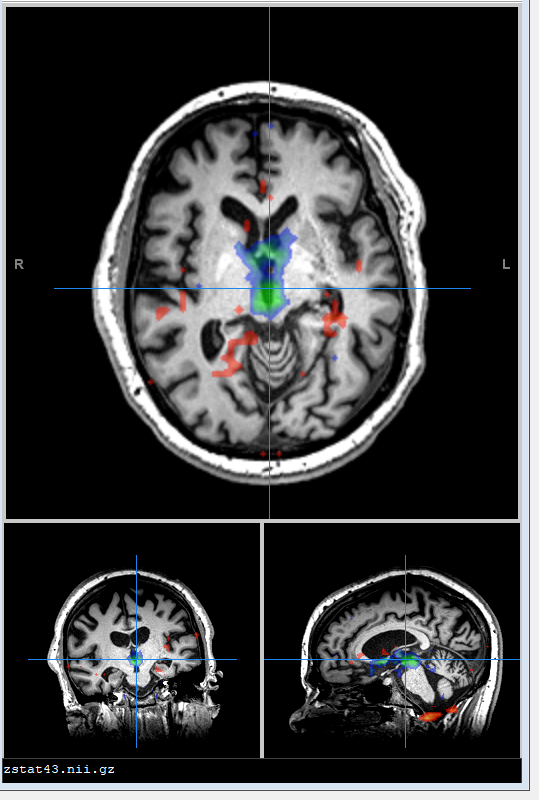  zstat 43 | 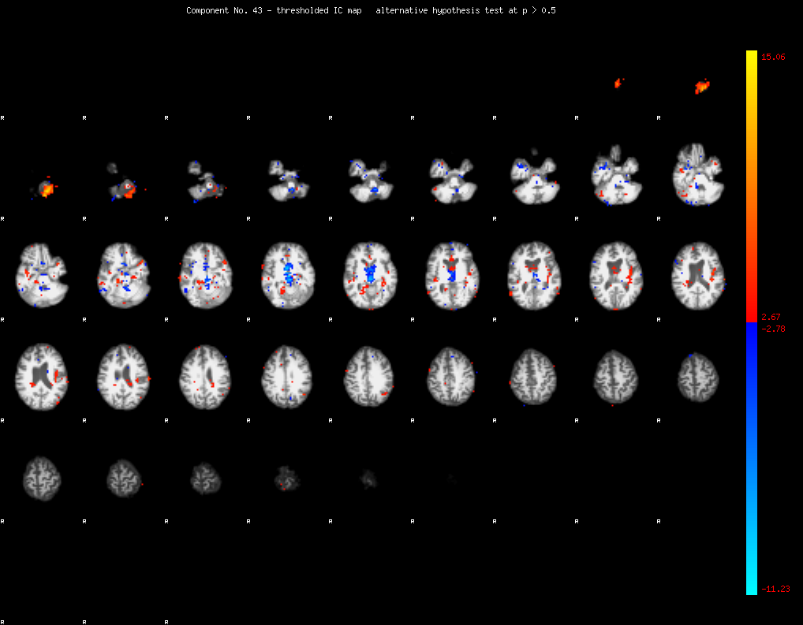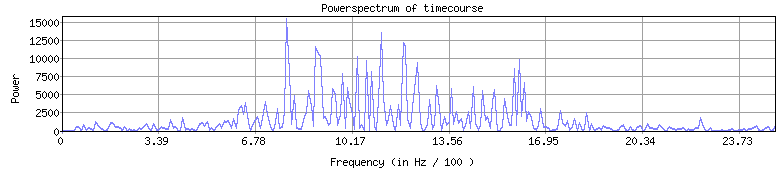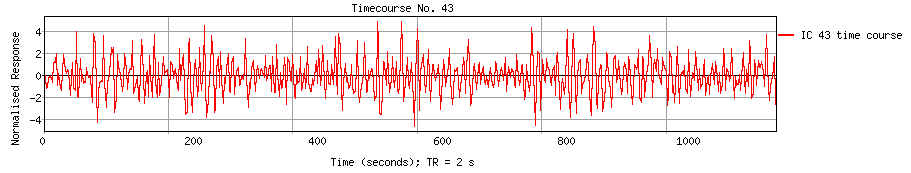 |
| --- | --- |

| 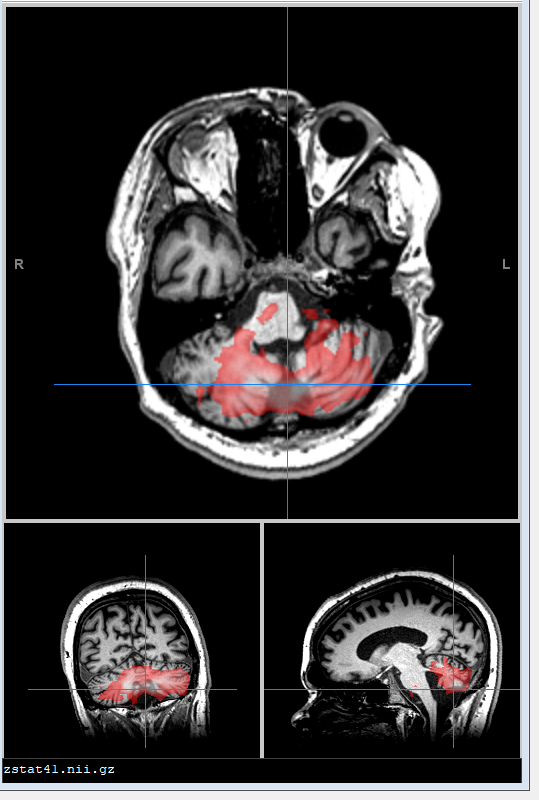  zstat 41 | 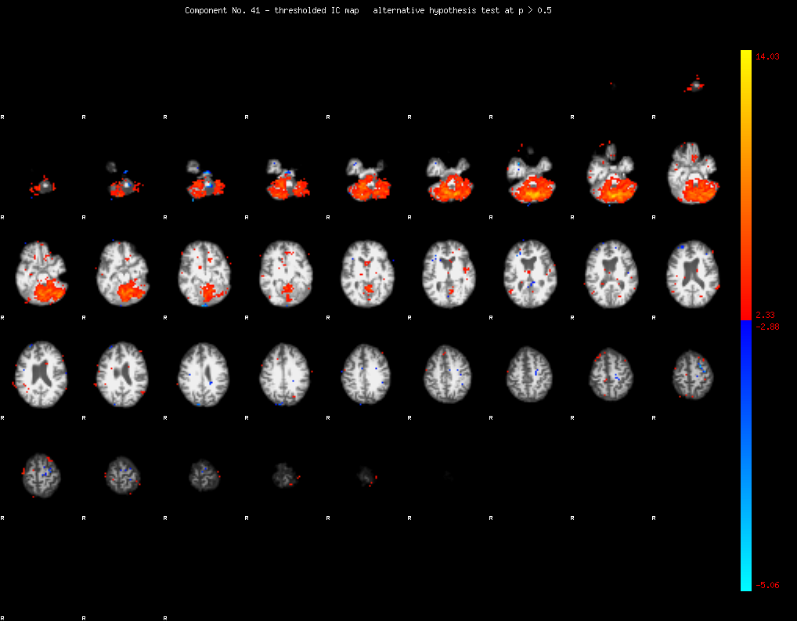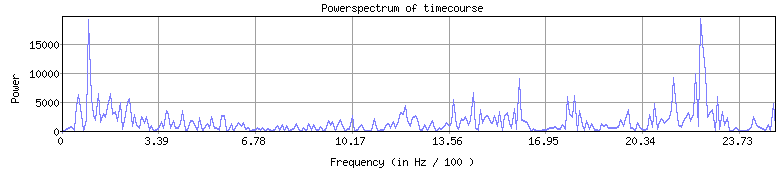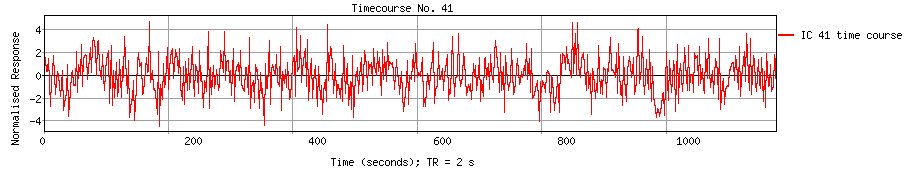 |
| --- | --- |

| **Atypical RSN** |
| --- |

| 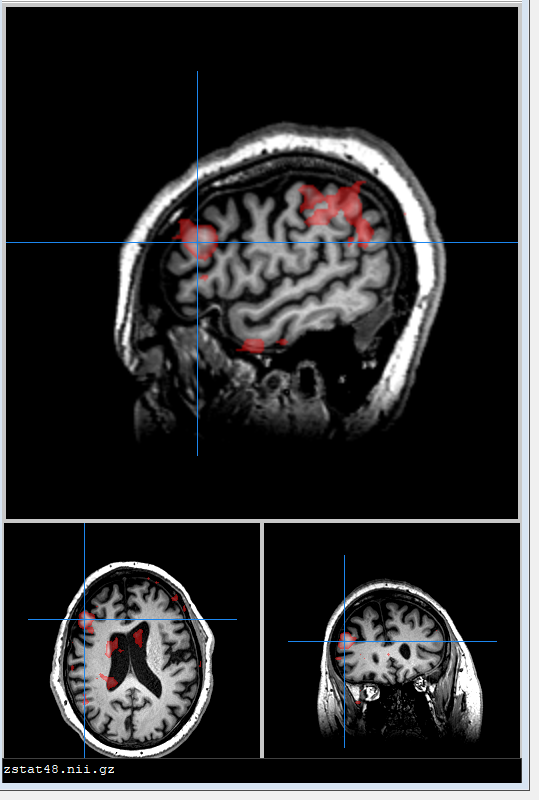  zstat 48 | | 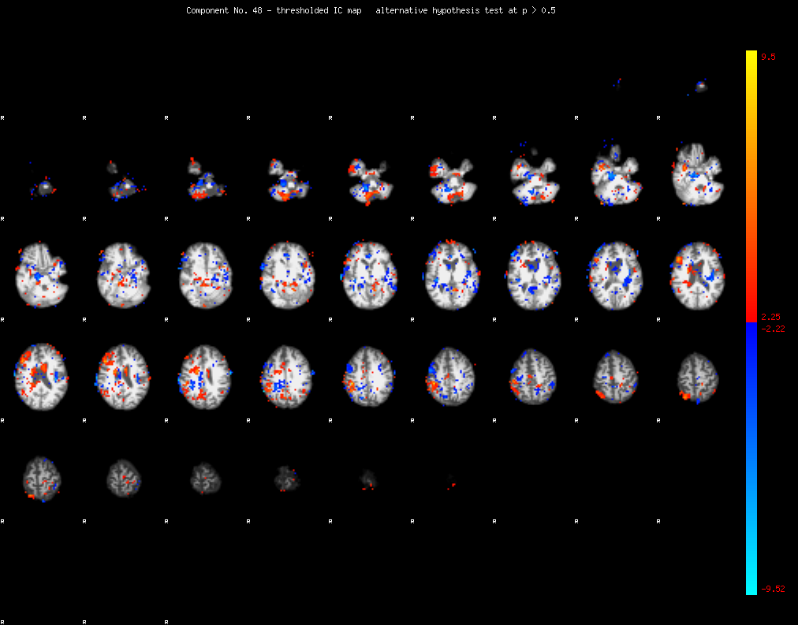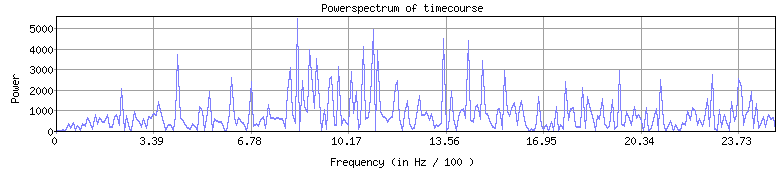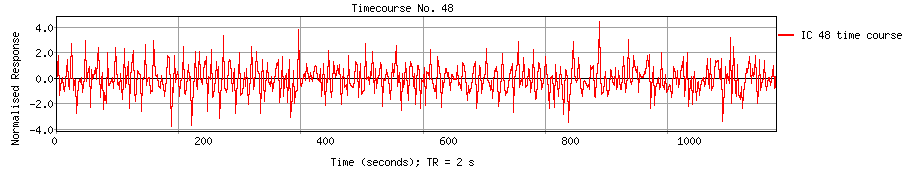 | |
| --- | --- | --- | --- |
|  |  | |  |
|  |  | |  |

| **Motor** | | | |
| --- | --- | --- | --- |
| 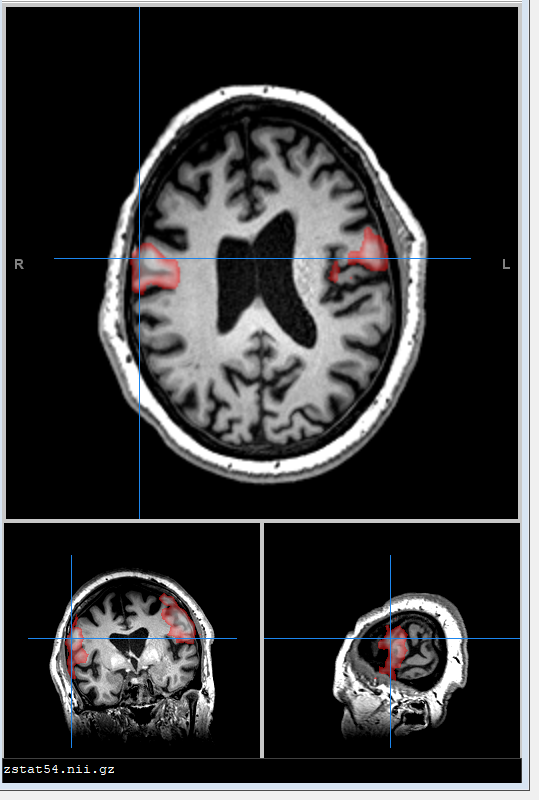  zstat 54 | | 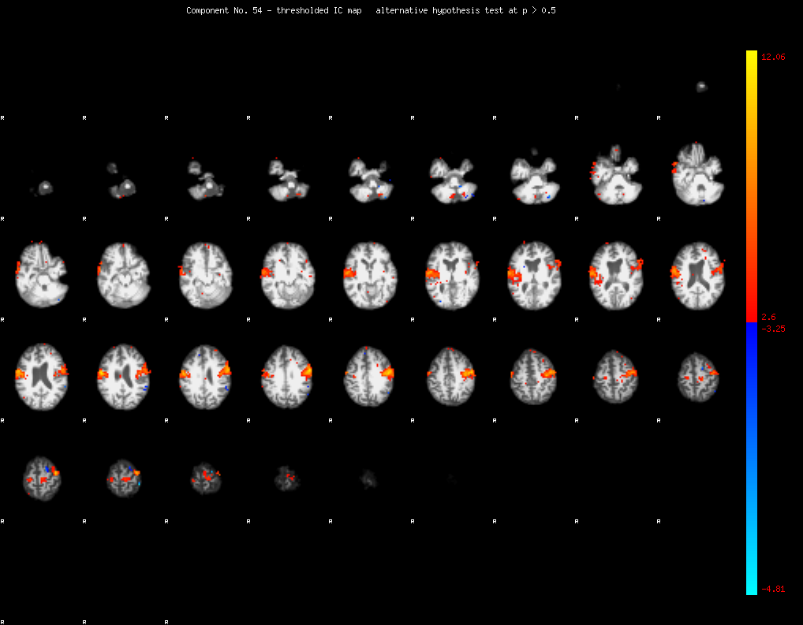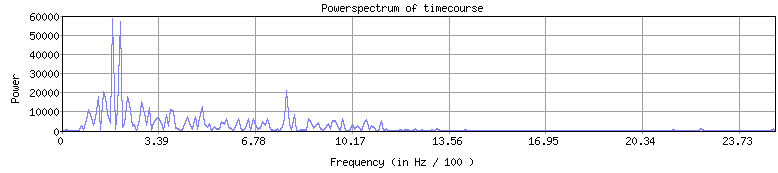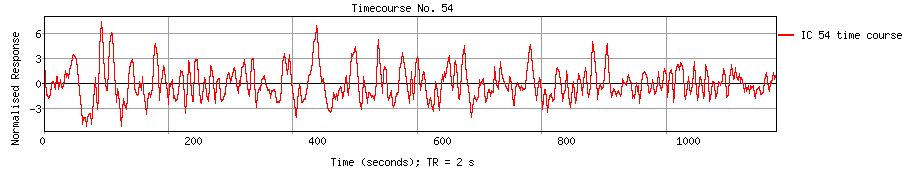 | |
| 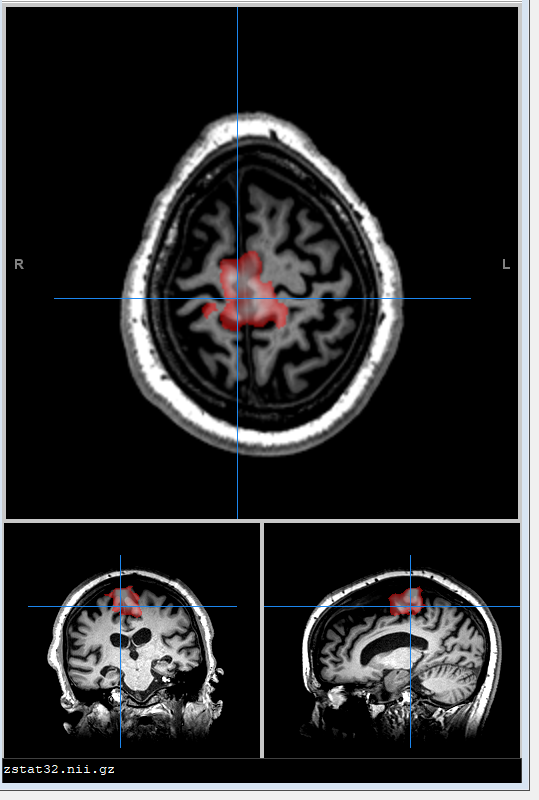  zstat 32 | | 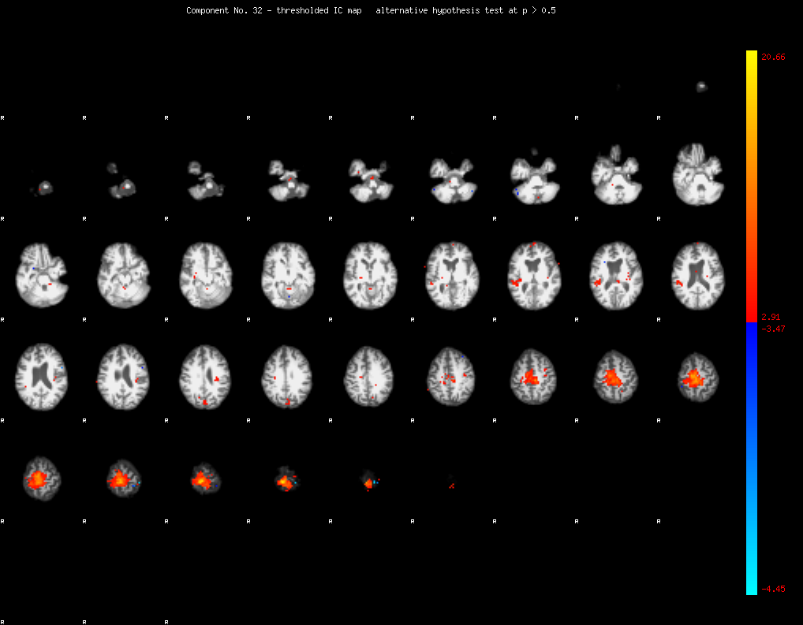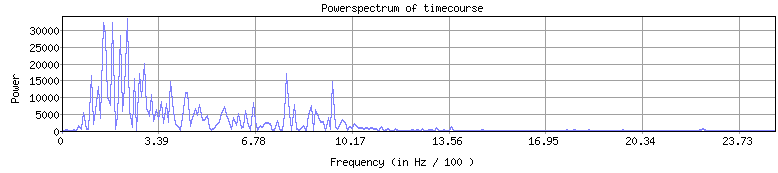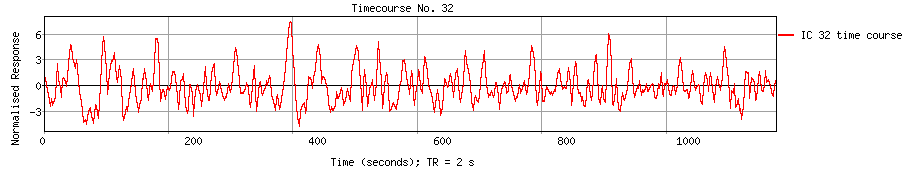 | |
| 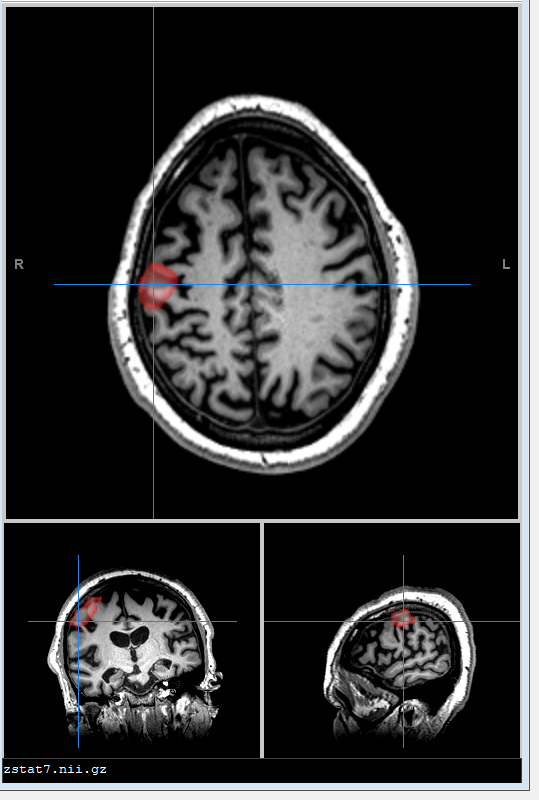  zstat 7 | 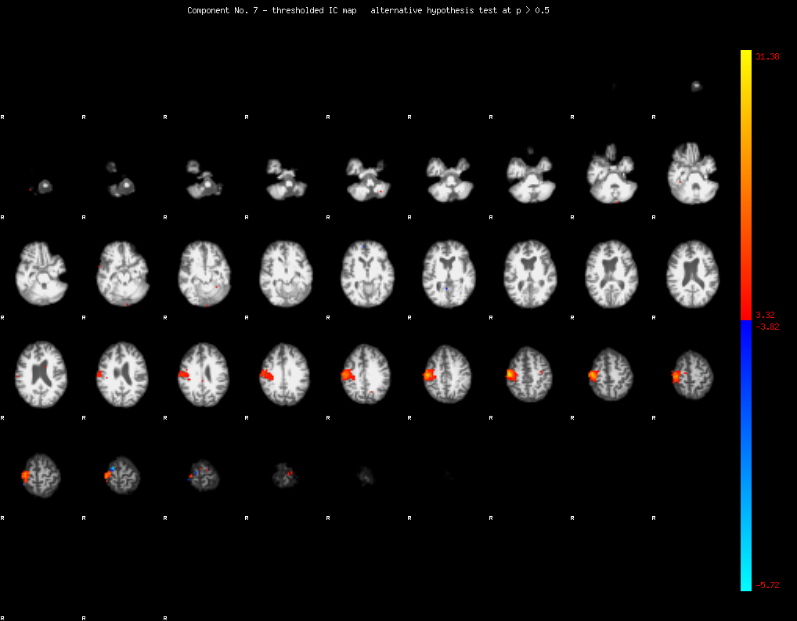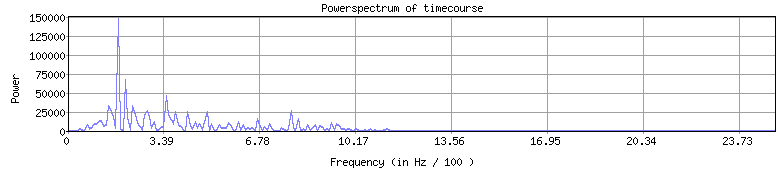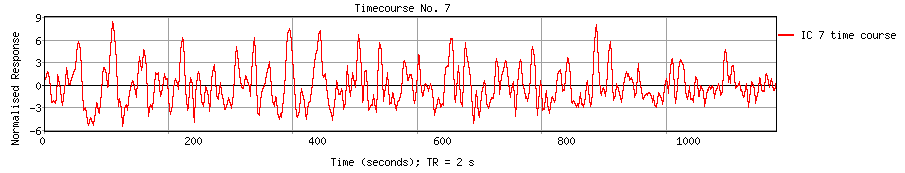 | |  |
| 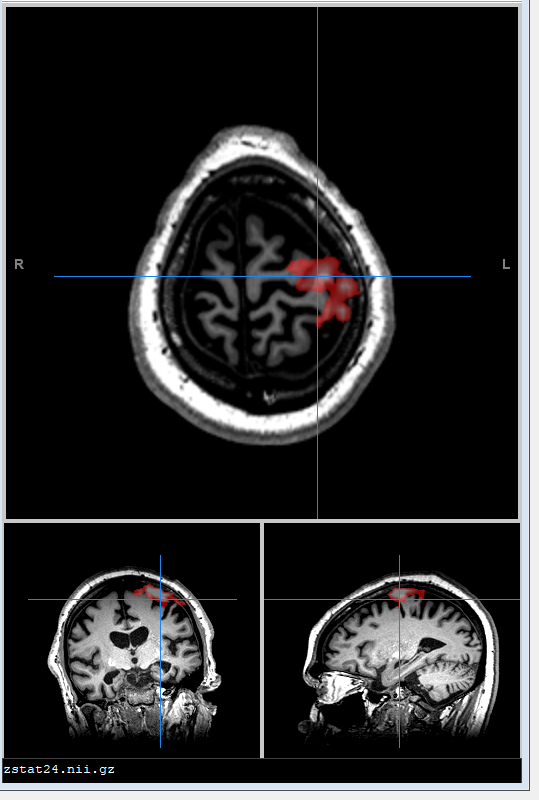  zstat 24 | 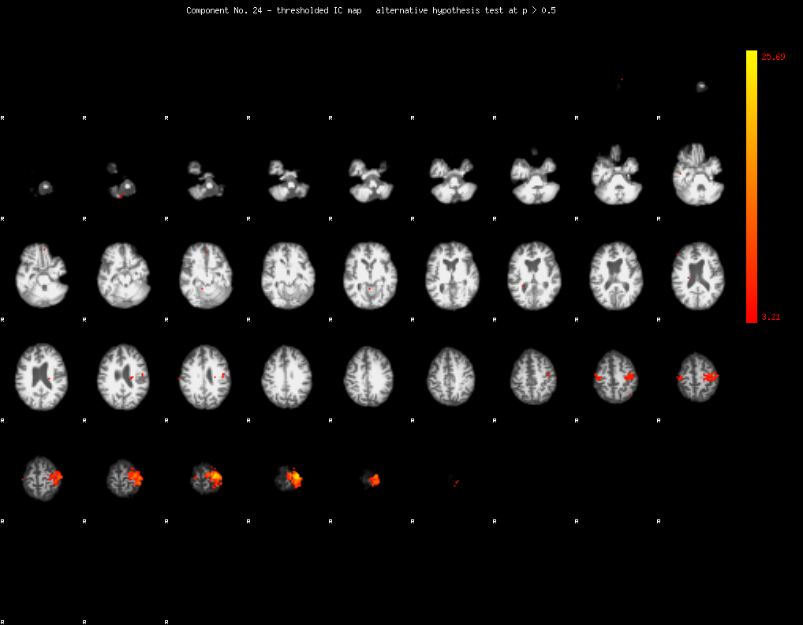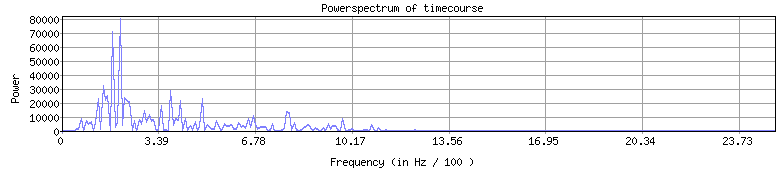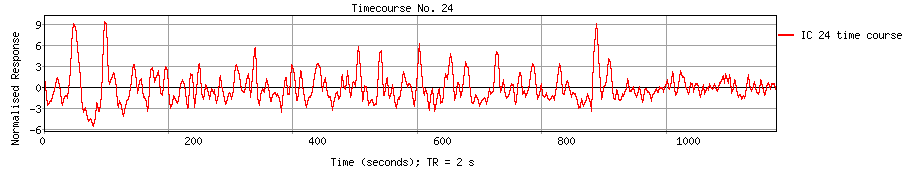 | |  |
|  |  | |  |
|  |  | |  |
| 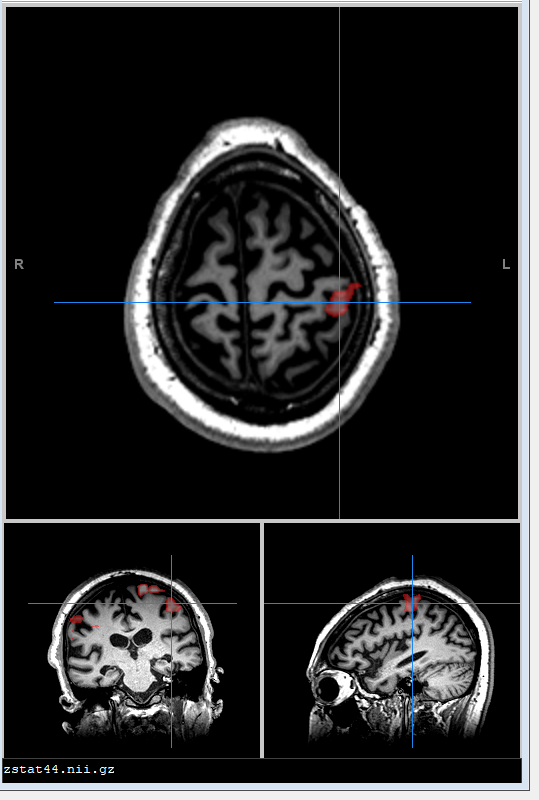  zstat 44 | 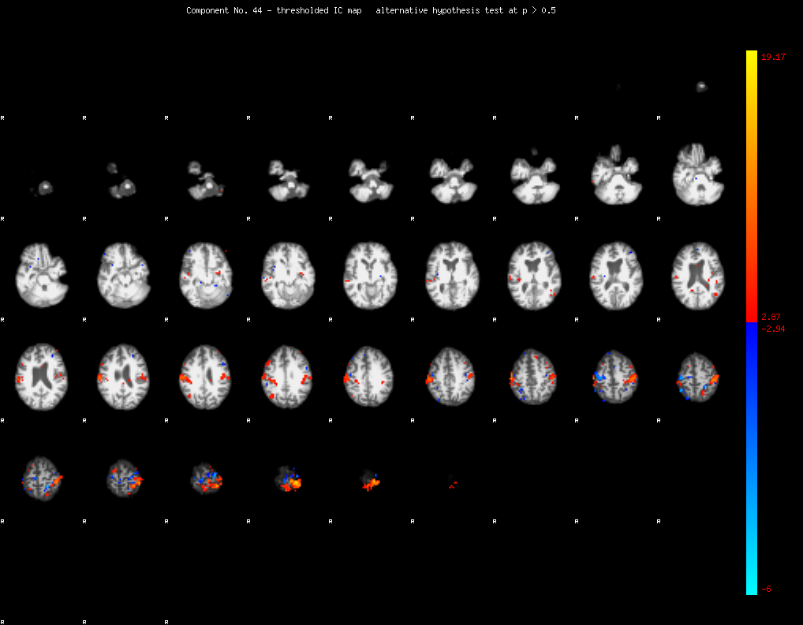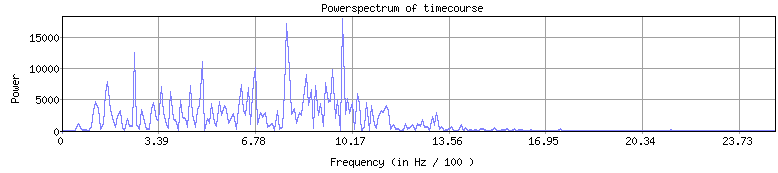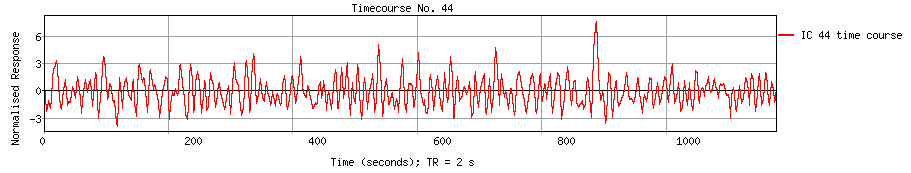 | |  |
| 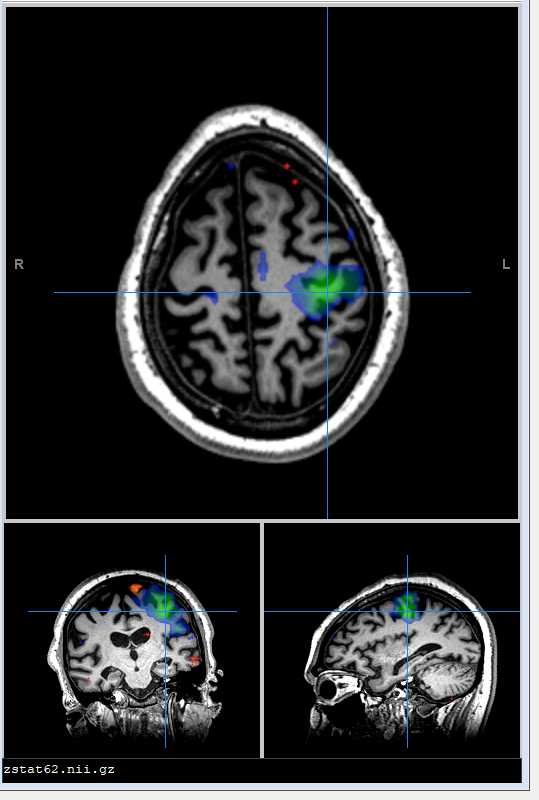  zstat 62 | 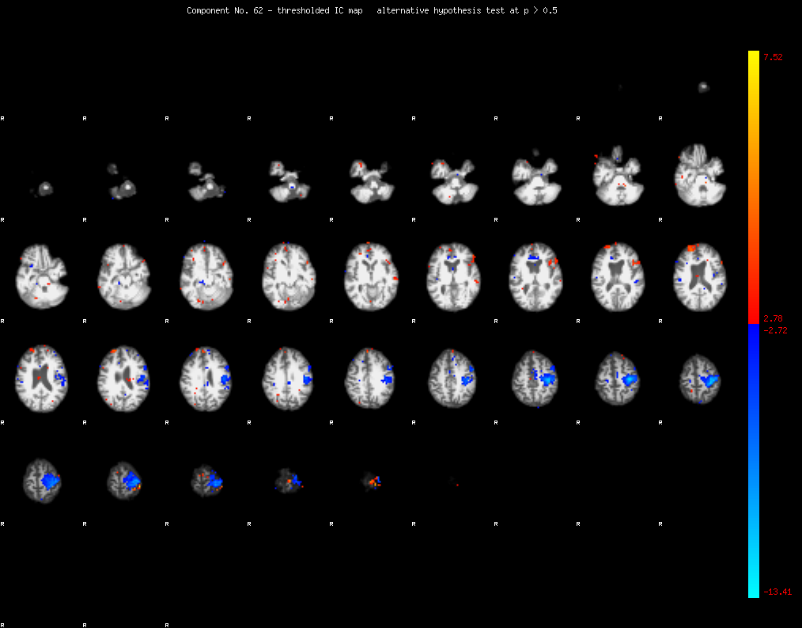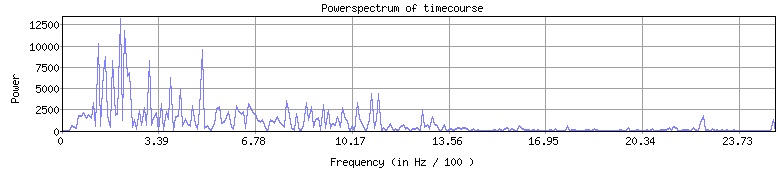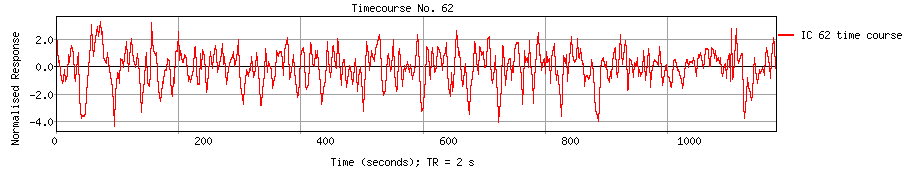 | |  |
|  |  | |  |
|  |  | |  |

| **Language** |
| --- |

| 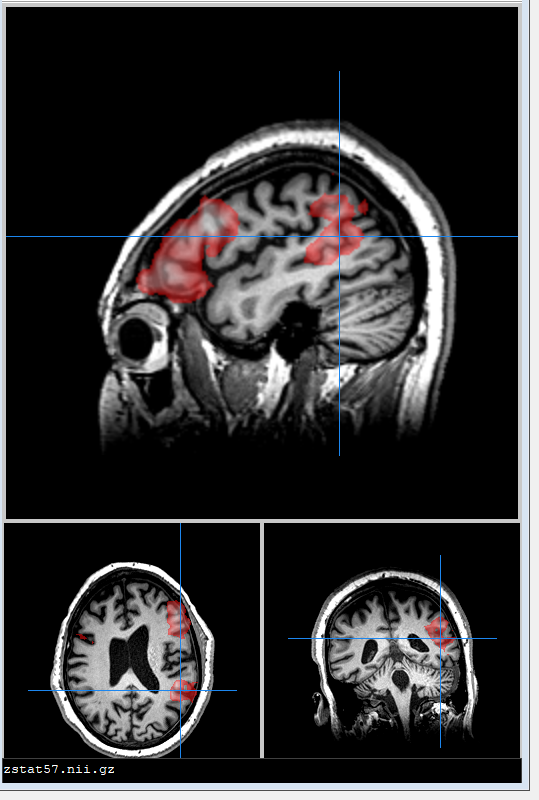  zstat 57 | 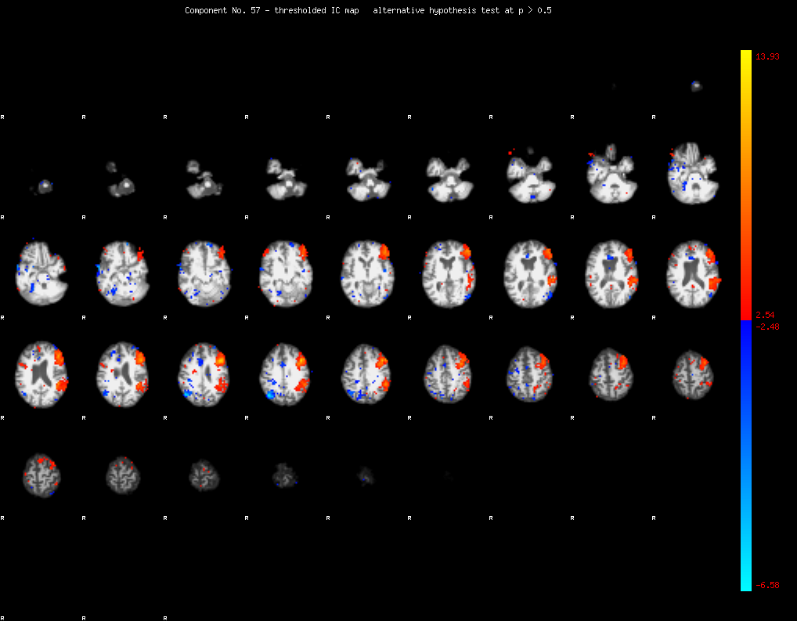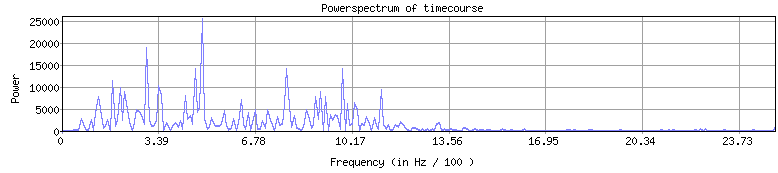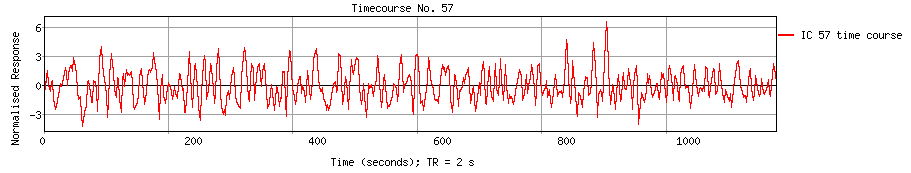 |
| --- | --- |
| 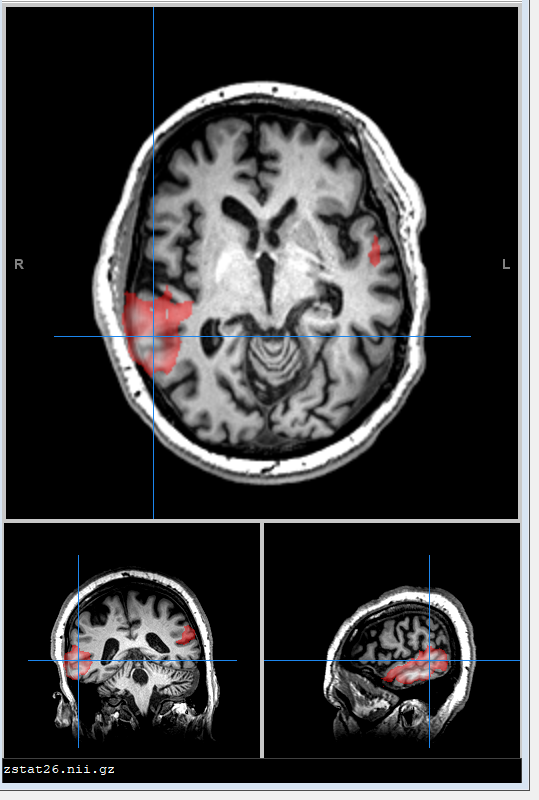  zstat 26 | 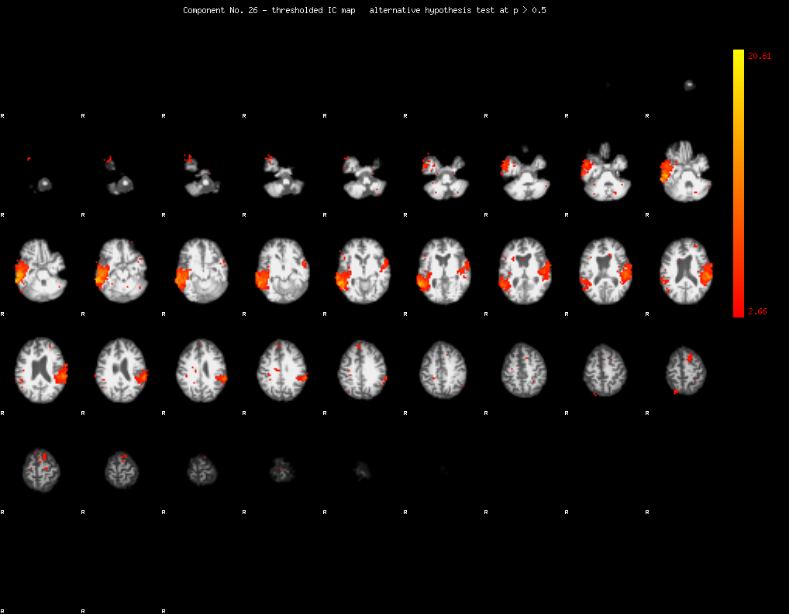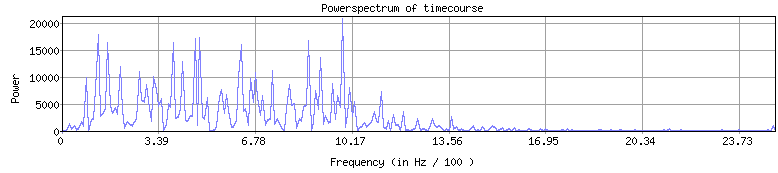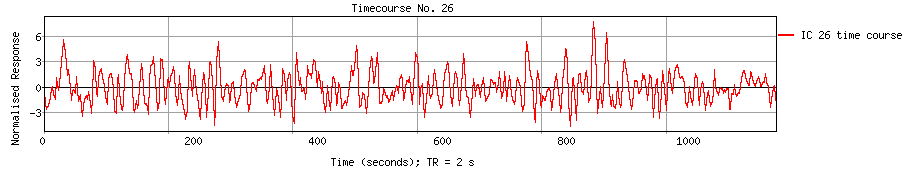 |
|  |  |

| 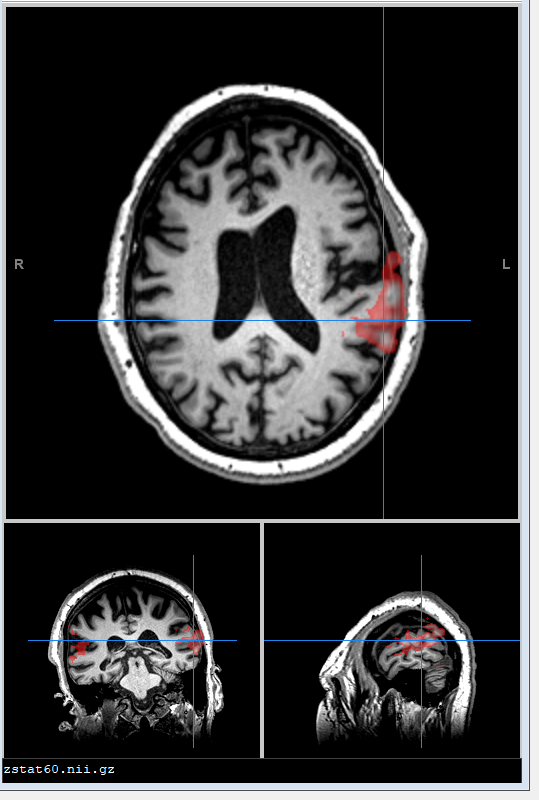  zstat 60 | 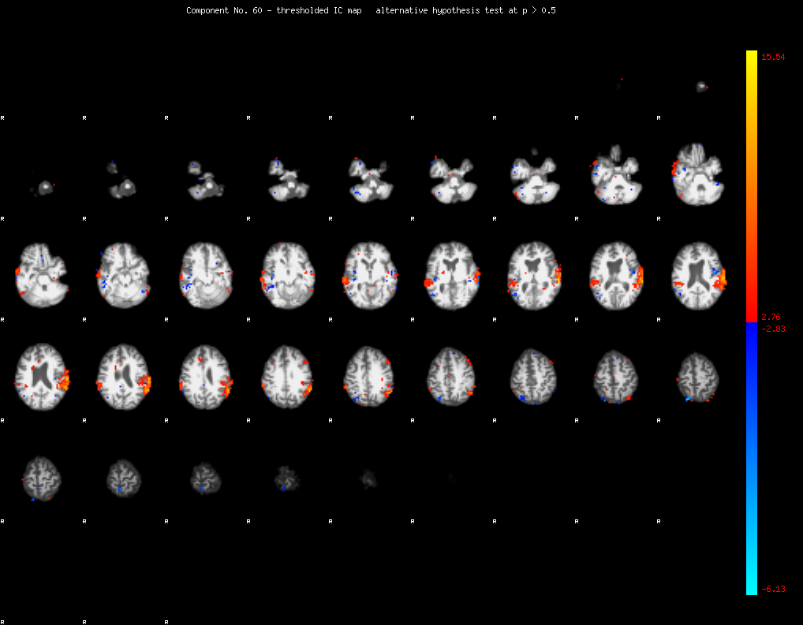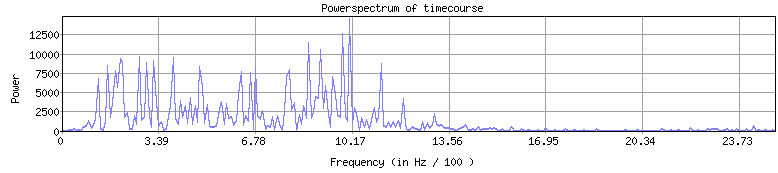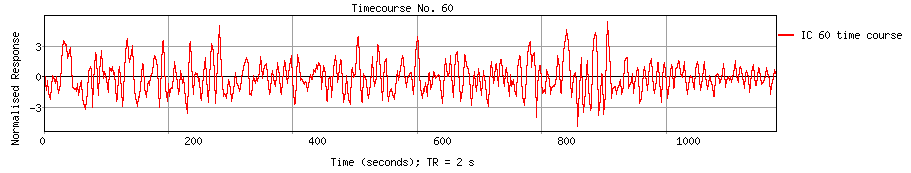 |
| --- | --- |
| 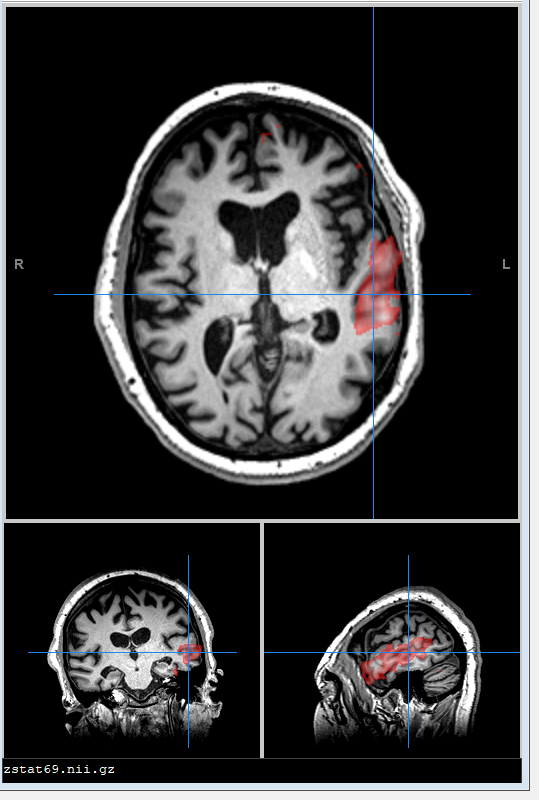  zstat 69 | 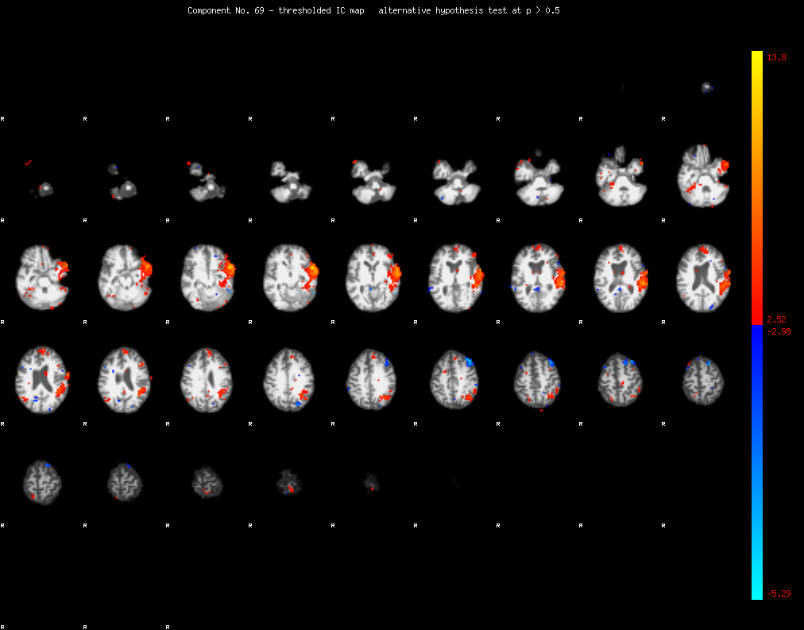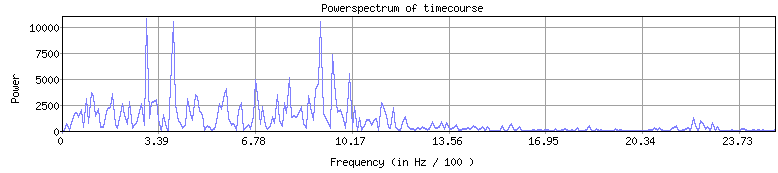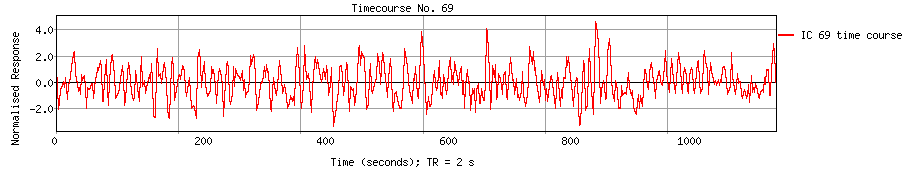 |

| **Parietal** | |
| --- | --- |
|  |  |
|  |  |
| 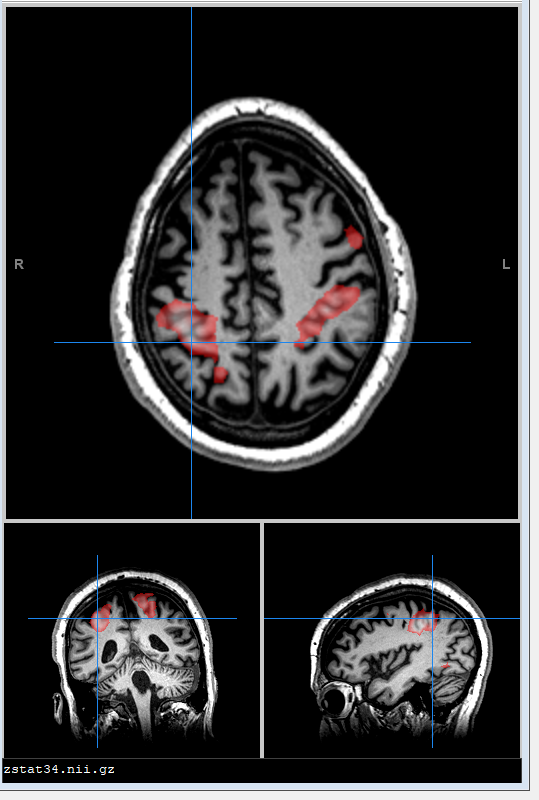  zstat 34 | 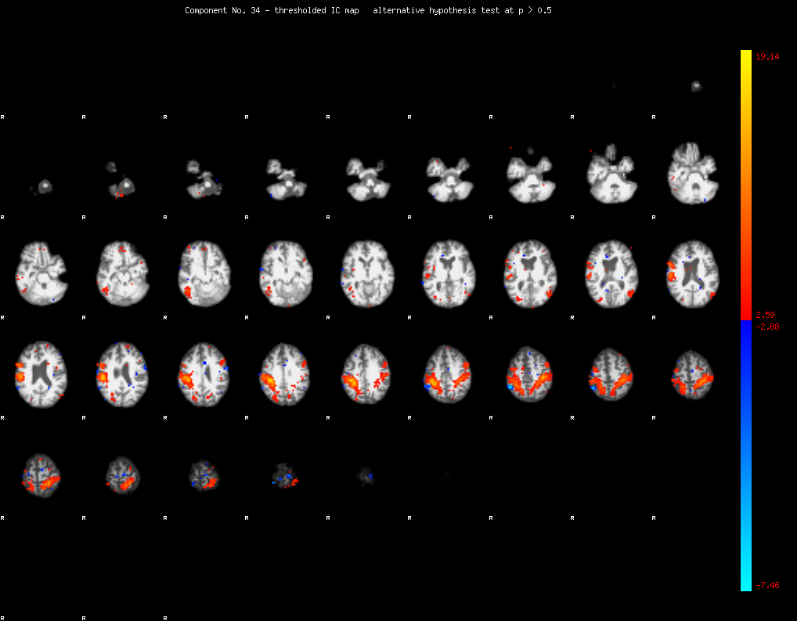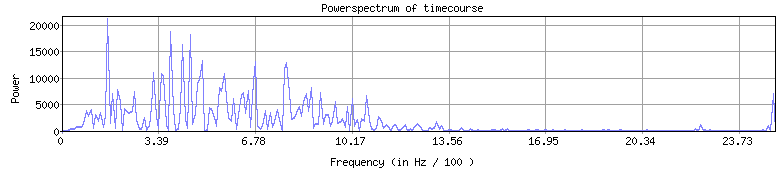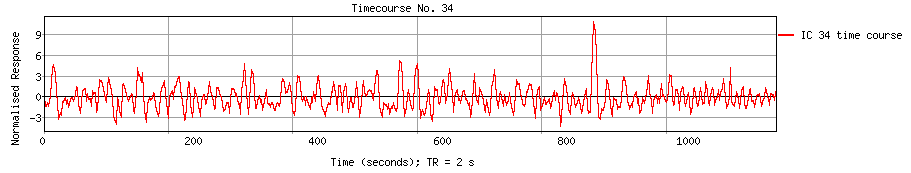 |
| 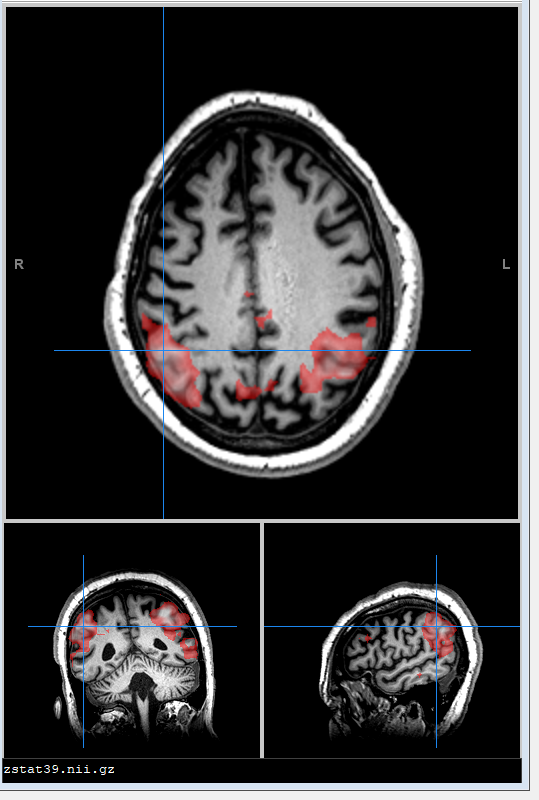  zstat 39 | 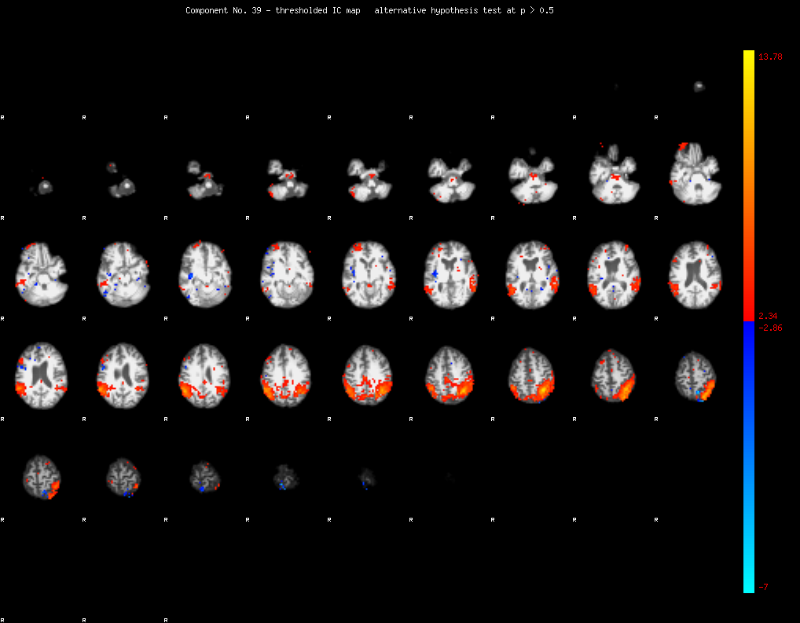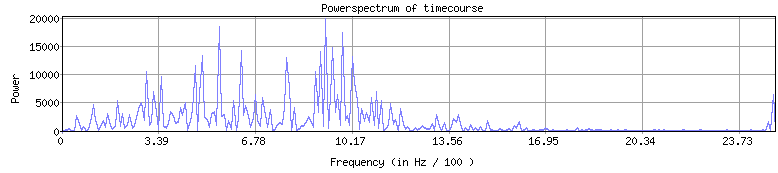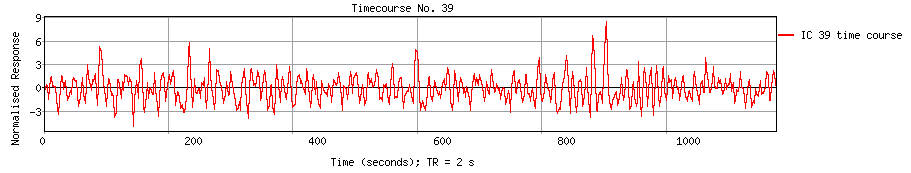 |
|  |  |
| 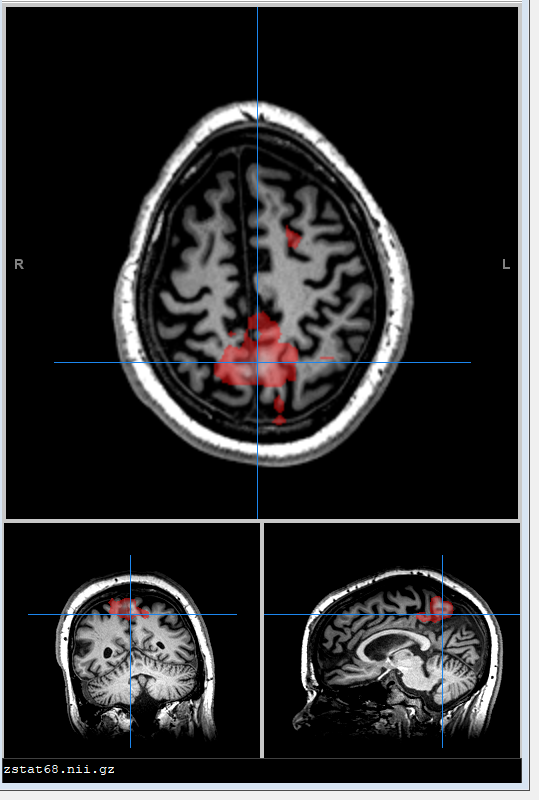  zstat 68 | 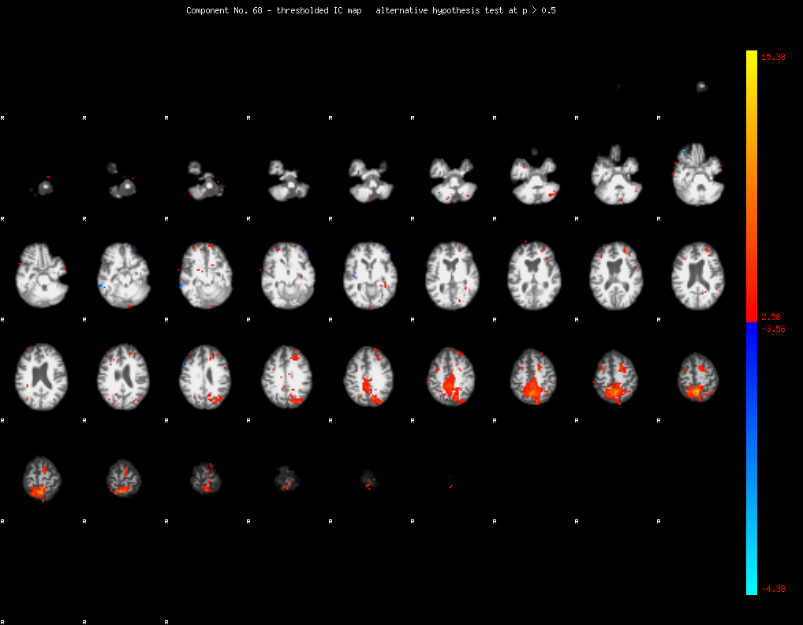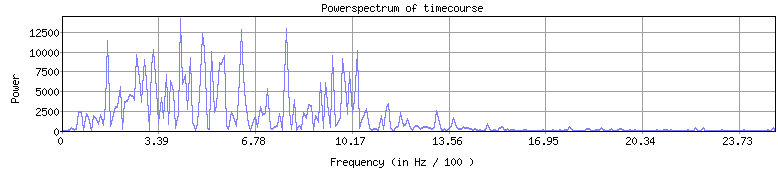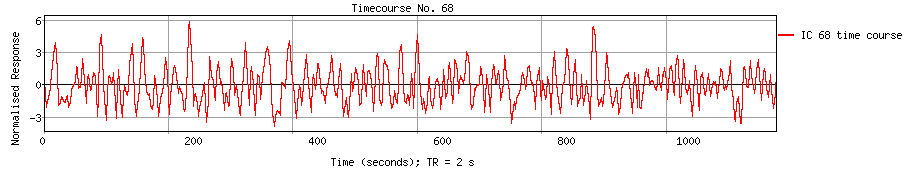 |
| 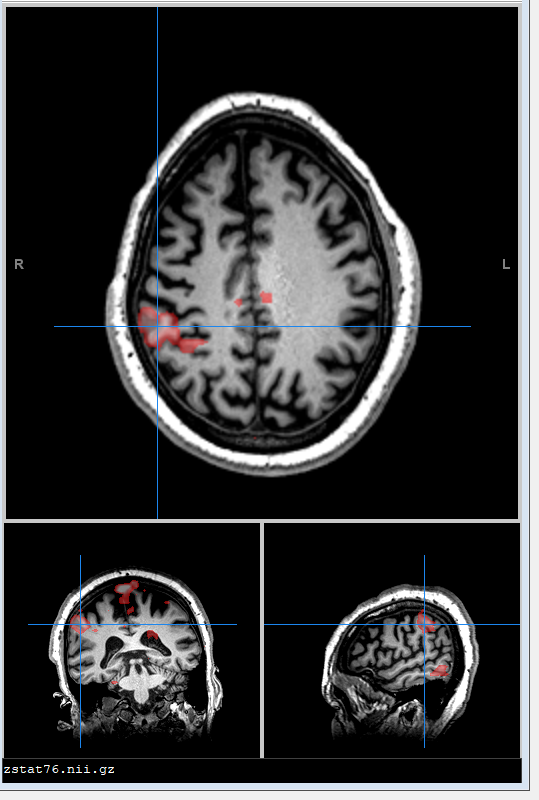  zstat 76 | 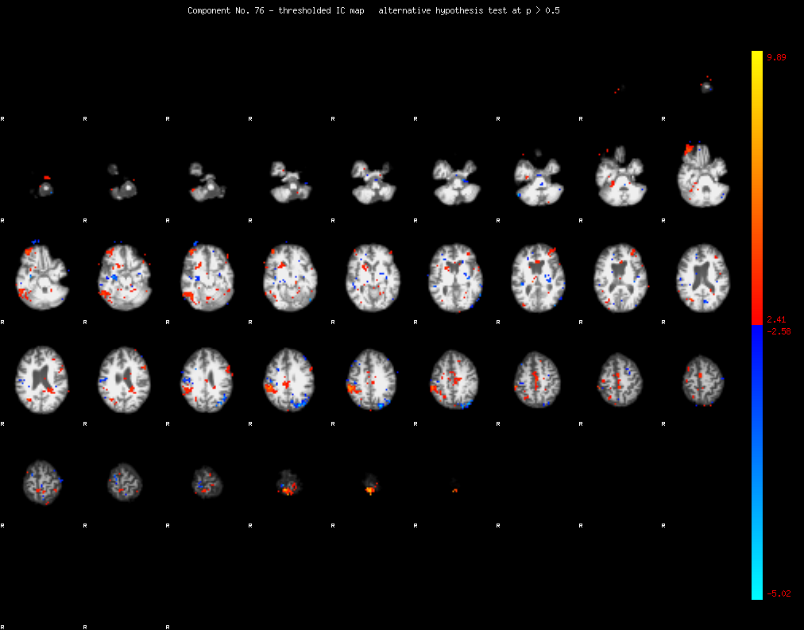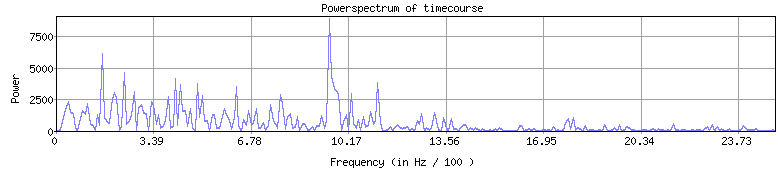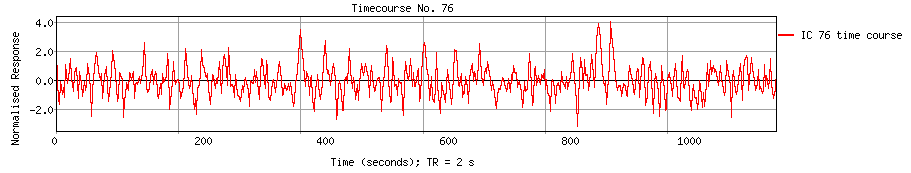 |
| 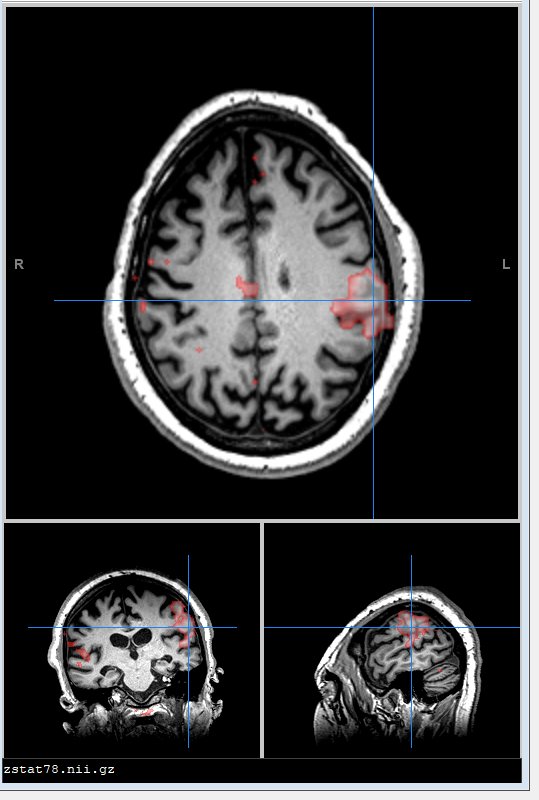  zstat 78 | 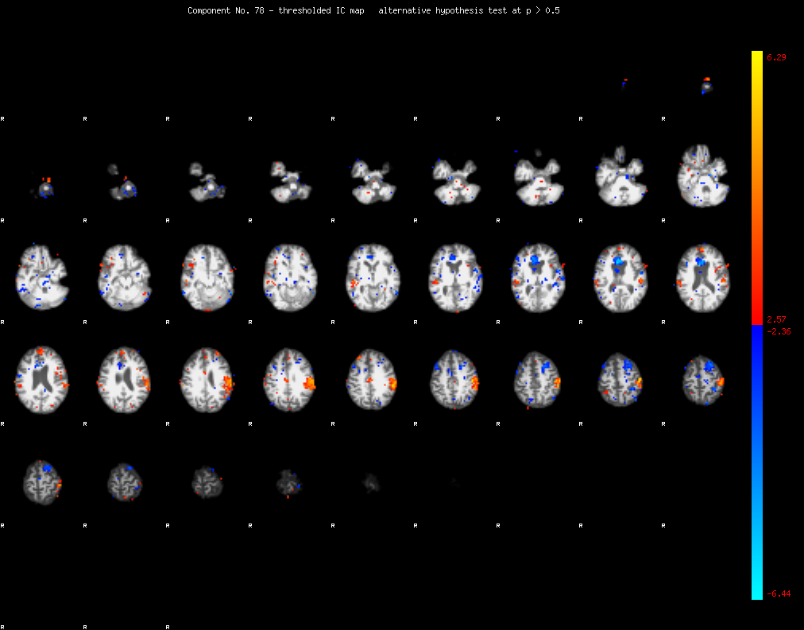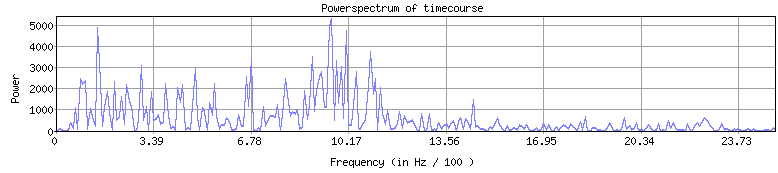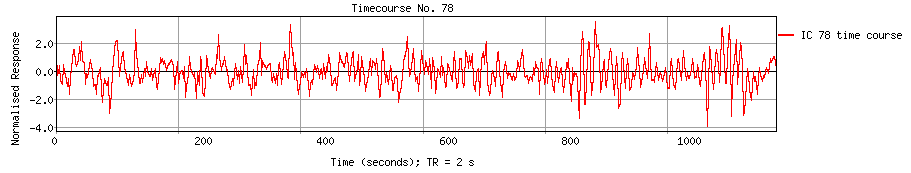 |
|  |  |

| **Frontal** | |
| --- | --- |
| 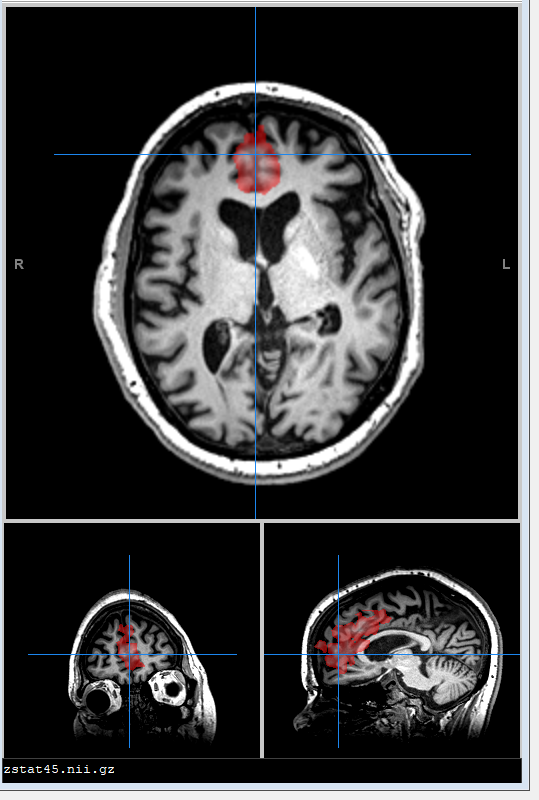  zstat 45 | 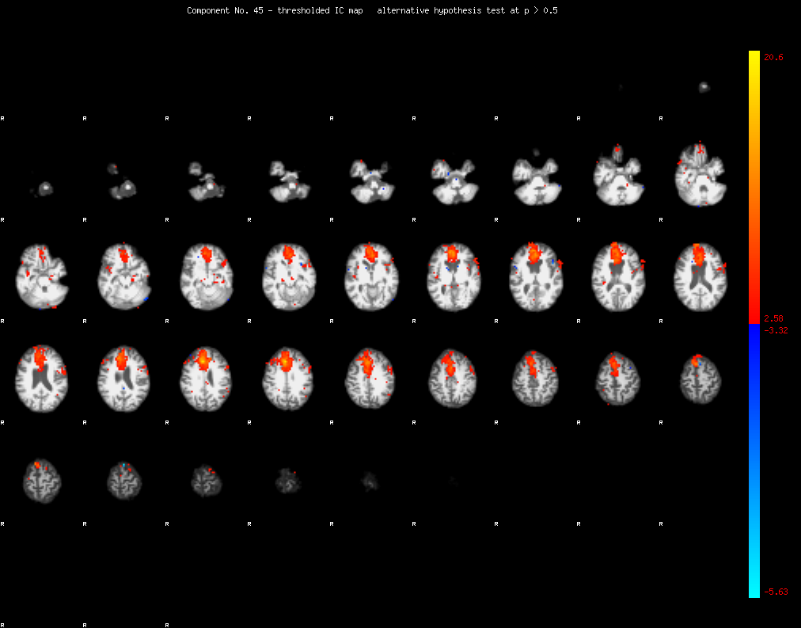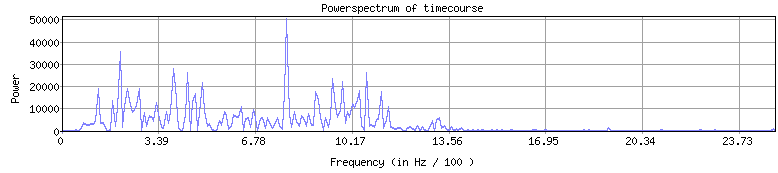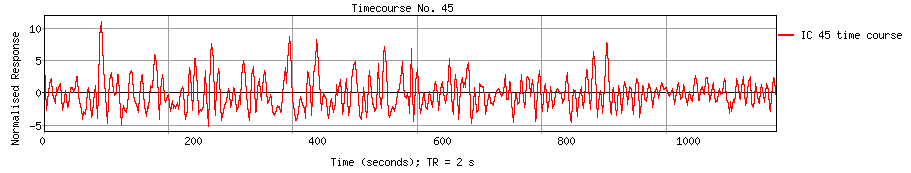 |
| 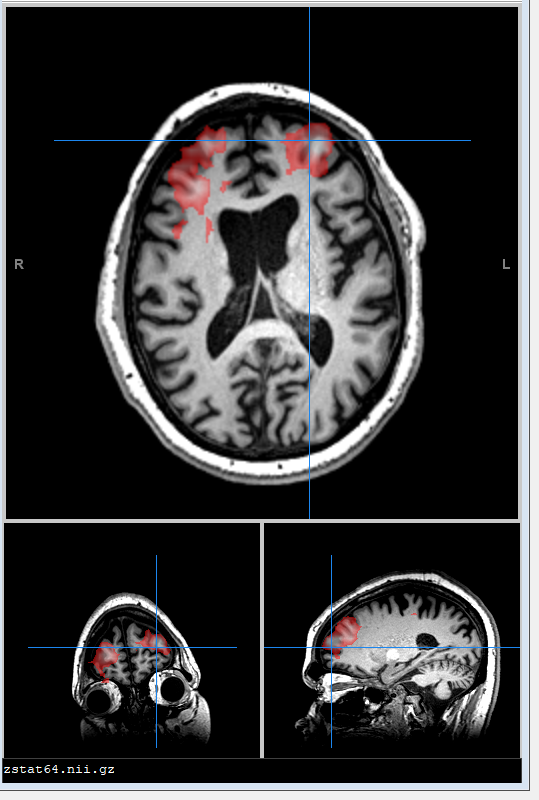  zstat 64 | 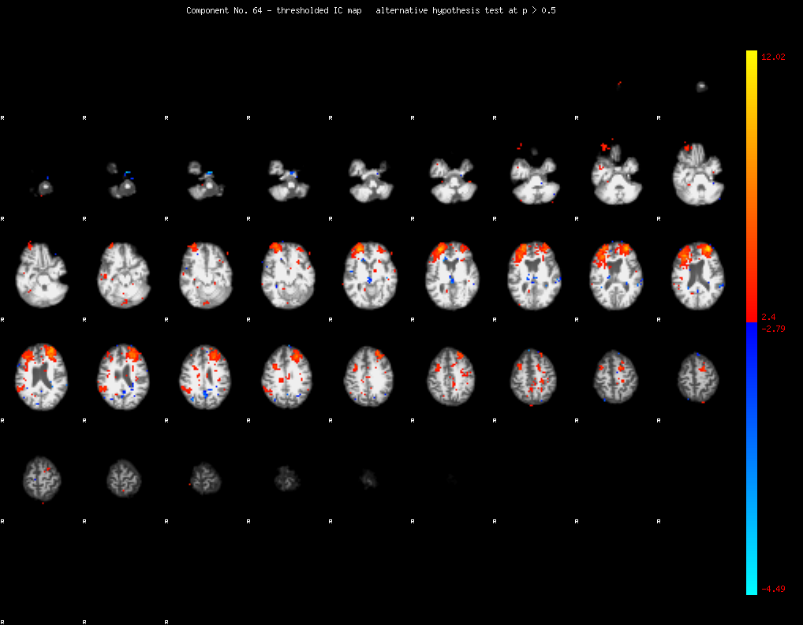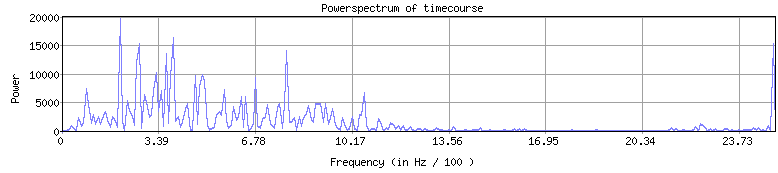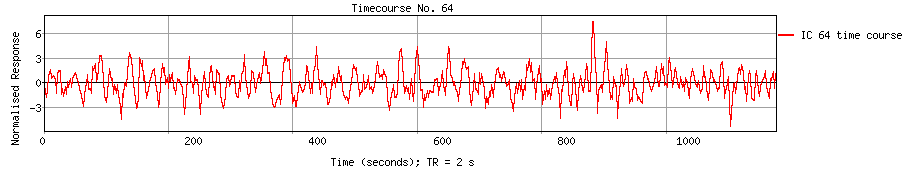 |
| 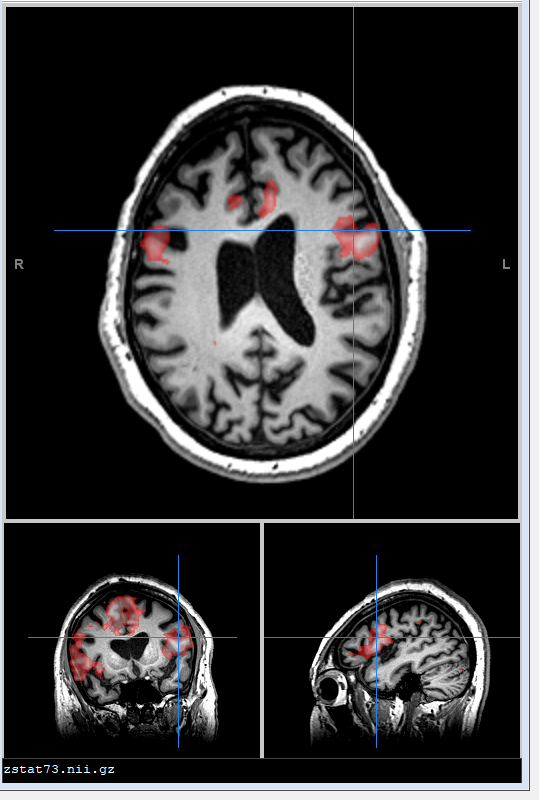  zstat 73 | 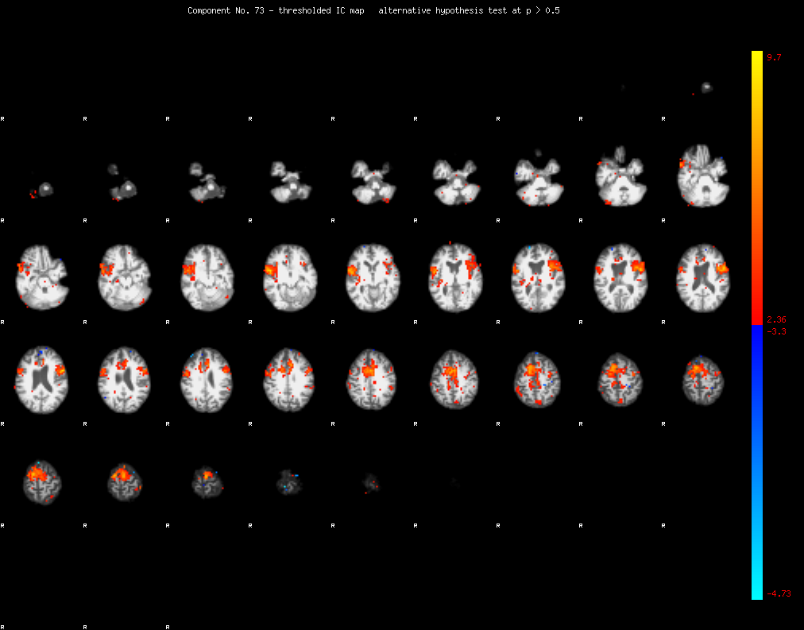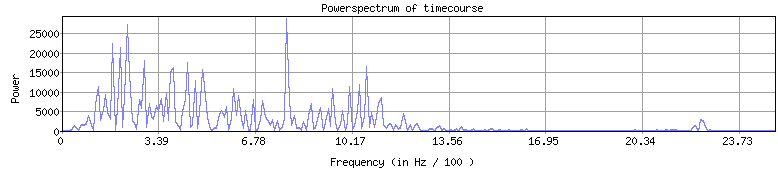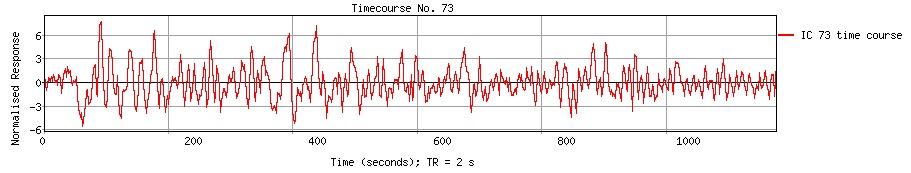 |

| 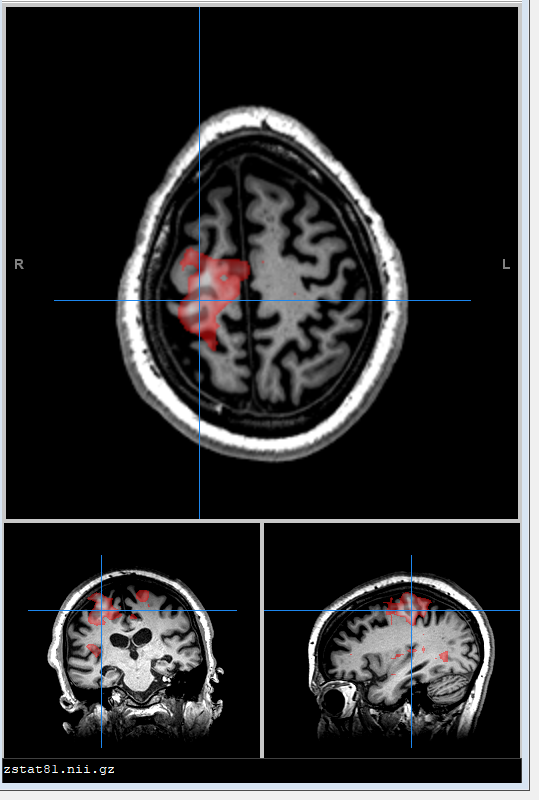  zstat 81 | 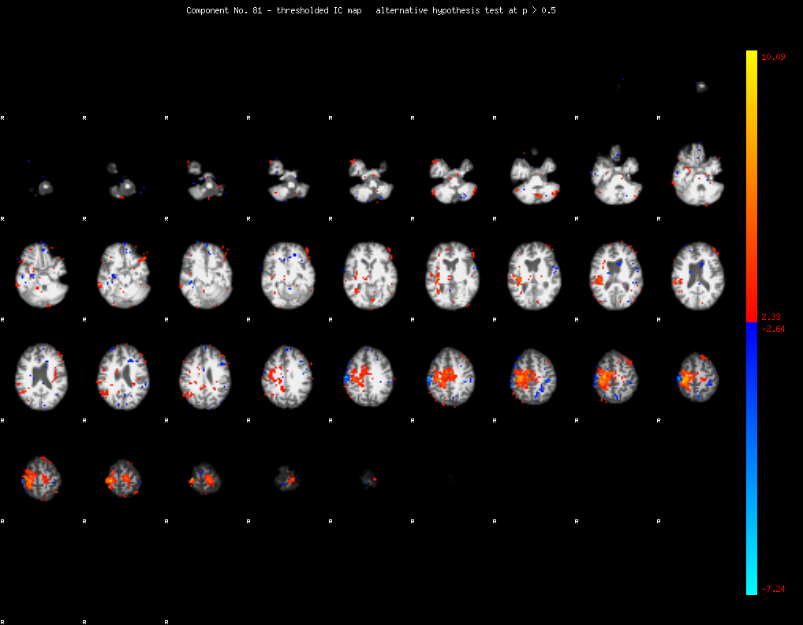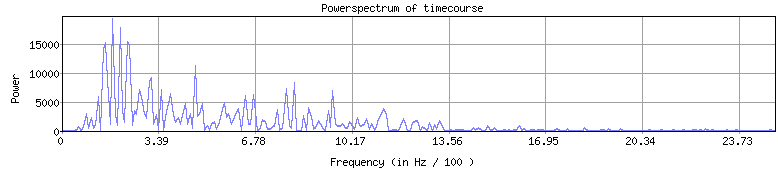 |
| --- | --- |
|  |  |

| zstat 51 |  |
| --- | --- |
|  |  |
| zstat 56 |  |

| **Temporal** | |
| --- | --- |
| zstat 18 |  |
| zstat 70 |  |
|  |  |

| **Vision** | |
| --- | --- |
| zstat 9 |  |
| zstat 12 |  |
|  |  |
| zstat 50 |  |
| zstat 55 |  |

| **Deep Grey** | |
| --- | --- |
| zstat 37 |  |
|  |  |
| zstat 66 |  |
|  |  |

| **Modulating** | |
| --- | --- |
| zstat 30 |  |
|  |  |
|  |  |

| **Association: Long-range Fronto-Parietal** |
| --- |

|  |  | |  |
| --- | --- | --- | --- |
|  |  | |  |
| zstat 80 | |  | |
|  | |  | |
| zstat 53 |  | |  |
| zstat 63 |  | |  |
| zstat 65 |  | |  |
|  |  | |  |

**Abbreviation Guide**

ACG Anterior Cingulate Gyrus

APF Anterior PreFrontal

AT anterior Temporal

B Bilateral

BG Basal Ganglia

DMN Default Mode Network

IFG Inferior Frontal Gyrus

IFS Inferior Frontal Sulcus

IPL Inferior Parietal Lobule

ITG Inferior Temporal Gyrus

L Left

LR-FTP Long Range Fronto-to-Parietal association network, anterior prefrontal IFG, MFG, posterior lateral parietal S2

LS2 – lateral Secondary Sensory area

MFG Middle Frontal Gyrus

MT mesial Temporal

MTG Middle Temporal Gyrus

mS2 – medial Secondary Sensory area

OPC- Operculum

OTG Occipito-temporal gyrus

PCG Posterior Cingulate Gyrus

PFC Prefrontal Cortex

PMC Premotor Cortex

PHG Parahippocampal Gyrus

POS Parietal Occipital Sulcus

R Right

Rs-fMRI Resting state functional MRI

RS Resting State

RSN Resting state network – an expected brain network

S1 Primary somatosensory cortex

S2 Secondary somatosensory cortex or secondary sensory association area

SFG Superior Frontal Gyrus

SFS Superior Frontal Sulcus

SMA Supplementary Motor Association area

SMG Supramarginal Gyrus

SOZ Seizure onset zone

SPL Superior Parietal Lobule

STG Superior Temporal Gyrus

STS Superior Temporal Sulcus

TOJ – Temporal Occipital Junction network

V1 Primary visual cortex associated network

V2 Secondary visual cortex associated network

vmPFC ventral medial prefrontal cortex
